# Supplementary material for: Metabolic rewiring in keratinocytes by miR‐31‐5p identifies therapeutic intervention for psoriasis
Source: EMBO Mol Med. 2023 Mar 1;15(4):e15674. doi: 10.15252/emmm.202215674 (PMC10086589; doi:10.15252/emmm.202215674)
Supplement: Supplementary file 1 — Expanded View Figures PDF [file EMMM-15-e15674-s004.pdf]

Expanded View Figures

Figure EV1. Identification of targets of miR-31 by stable isotope labeling by amino acids in cell culture proteomics.

A Quantitative RT–PCR detection of Has-miR-31-5p expression levels in skin biopsies of healthy individuals and psoriasis patients (*n* = 5 individuals), left panel; and mmu-miR-31-5p expression levels in skin biopsies of healthy mice or imiquimod-induced psoriatic mice (IMQ), *n* = 7 mice in each group, right panel.

B Violin plot of miR-31 host gene (miR-31 HG) expression in human skin biopsies. Data were extracted from an online microarray study (GSE13355). *n* = 64/58 in healthy control (HC)/psoriasis patients (PN, psoriatic non-lesional skin; PL, psoriatic lesional skin), respectively.

C Schematic representation of the workflow for SILAC proteomics.

D, E Volcano plots summarizing SILAC proteomics data in HEK 293 T cells (D) and HaCaT cells (E).

F Correlation analysis of SILAC proteomics data of HEK 293 T cells and HaCaT cells.

G, H Volcano plot representation of changes in validated targets of miR-31 in HEK 293 T cells (G) and HaCaT cells (H) proteomics.

I Principal component analysis (PCA) indicating a specific metabolism signature induced by miR-31 overexpression in HEK 293 T cells.

Data information: In (A) and (B), data are presented as truncated violin plot, and dash and solid lines represent quartiles and median, respectively (by unpaired Student's *t*-test for (A) and one-way ANOVA with Sidak test for (B)).
Source data are available online for this figure.

© 2023 The Authors

© 2023 The Authors

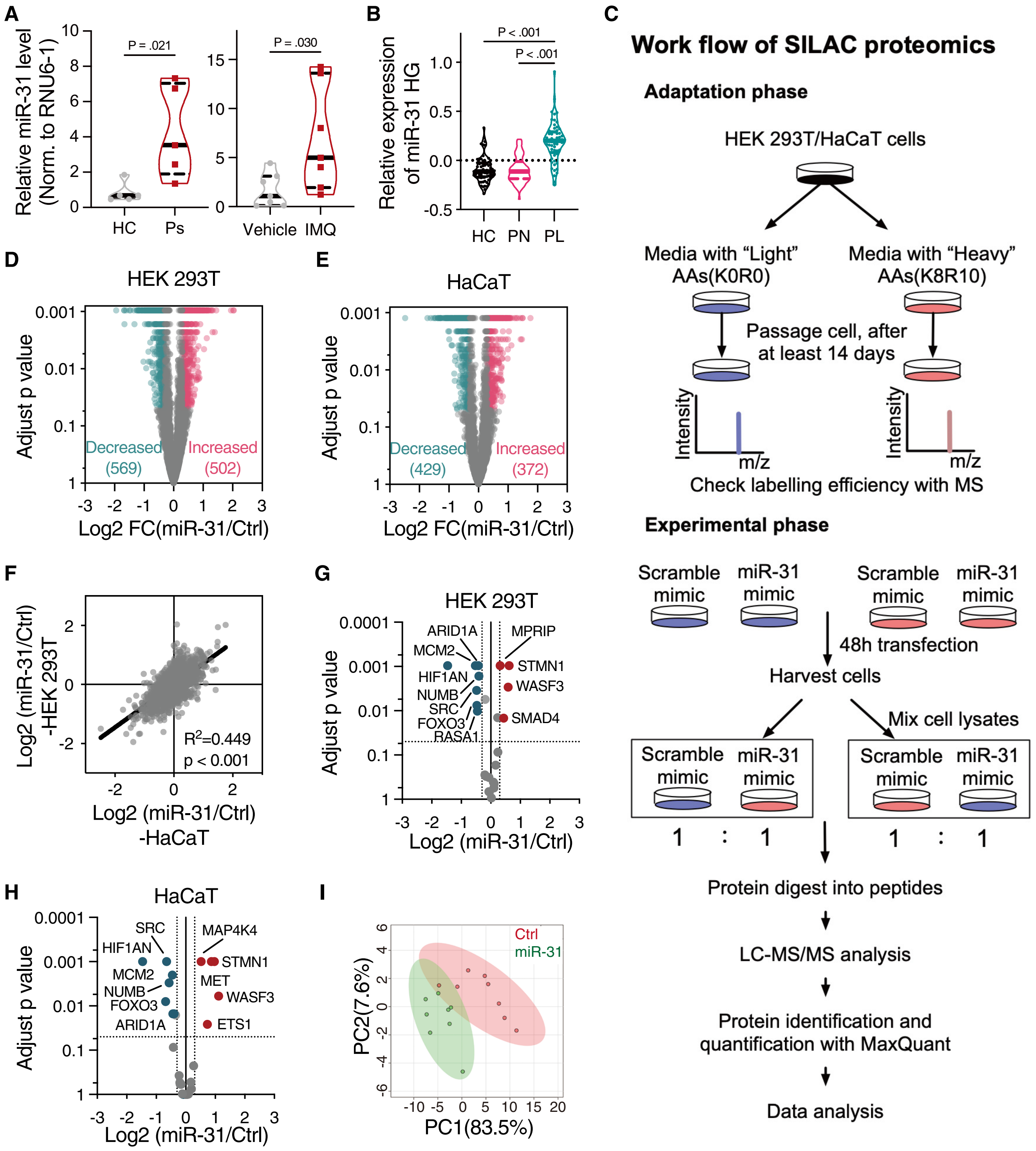
A
Relative miR-31 level (Norm. to RNU6-1)
P = .021
P = .030
HC
Ps
Vehicle
IMQ
B
Relative expression of miR-31 HG
P < .001
P < .001
HC
PN
PL
C
Work flow of SILAC proteomics
Adaptation phase
HEK 293T/HaCaT cells
Media with "Light" AAs(K0R0)
Media with "Heavy" AAs(K8R10)
Passage cell, after at least 14 days
Intensity
m/z
Check labelling efficiency with MS
Experimental phase
Scramble mimic
miR-31 mimic
48h transfection
Harvest cells
Mix cell lysates
1 : 1
Protein digest into peptides
LC-MS/MS analysis
Protein identification and quantification with MaxQuant
Data analysis
D
HEK 293T
Adjust p value
Decreased (569)
Increased (502)
Log2 FC(miR-31/Ctrl)
E
HaCaT
Decreased (429)
Increased (372)
F
Log2 (miR-31/Ctrl) -HEK 293T
R2=0.449
p < 0.001
Log2 (miR-31/Ctrl) -HaCaT
G
HEK 293T
ARID1A
MCM2
HIF1AN
NUMB
SRC
FOXO3
RASA1
MPRIP
STMN1
WASF3
SMAD4
Log2 (miR-31/Ctrl)
H
HaCaT
SRC
HIF1AN
MCM2
NUMB
FOXO3
ARID1A
MAP4K4
STMN1
MET
WASF3
ETS1
I
Ctrl
miR-31
PC2(7.6%)
PC1(83.5%)

Figure EV1.

Figure EV2. MiR-31 inhibits glycolysis and rewires glutamine metabolism.

- A 2-NBDG uptake of HaCaT cells in response to different doses of glucose (n = 3 biological replicates).
- B Immunostaining of GLUT1 in HaCaT cells of 48 h treatments of scramble, miR-31 mimic, and GLUT1 siRNA (si-GLUT1). DAPI is gray and GLUT1 is green. Similar results were obtained from three independent experiments. Scale bars: 20 μm.
- C Quantitative RT–PCR detection of GLUT1 expression levels in HaCaT cells (n = 3 biological replicates) and normal human epidermal keratinocytes (NHEK, n = 3 biological replicates) treated with or without 2 mM $CaCl_2$ upon miR-31 overexpression.
- D LDH activity assay was applied in HaCaT cells treated with 0 mM or 2 mM CaCl2 and HEK 293 T cells upon miR-31 overexpression and inhibition (n = 4 biological replicates).
- E Quantitative RT–PCR detection of PDHX expression levels in HaCaT cells (n = 3 biological replicates) and NHEK (n = 3 biological replicates) treated with or without 2 mM $CaCl_2$ upon miR-31 overexpression.
- F Western blot analysis of PDHX and GS in HaCaT and NHEK cells treated with or without 2 mM $CaCl_2$ upon miR-31 overexpression, and vinculin was used as loading control.
- G Quantitative RT–PCR detection of SLC7A11 and SLC3A2 in HaCaT cells upon miR-31 overexpression (n = 7 biological replicates).
- H Baselined extracellular acidification rates (ECAR) and oxygen consumption rates (OCR) of HaCaT cells in response to glutamine (Gln) or glutamate (Glu) addition in a long-term observation (n = 5 biological replicates).
- I A summary scheme showing how miR-31 rewires glutamine metabolism.
- J, K FACS analysis of 2-NBDG uptake of HaCaT cells upon treatments of GS siRNA (si-GS) and CB-839 (C, n = 4 biological replicates) or PC siRNA (si-PC, D, n = 3 biological replicates).
- L, M Glycolysis stress test (L) and mitochondrial stress test (M) of HaCaT cells upon GS siRNA treatment in 1 mM glucose condition (n = 5 biological replicates). ECAR and OCR were normalized to the amount of protein.

Data information: In (A), (C–E), (H), and (J–M), data are presented as mean ± SD (by One-way ANOVA with Sidak test for (J), unpaired Student's t-test for (K), and two-way ANOVA with Sidak test for (A), (C–E), (G), (L), and (M)).
Source data are available online for this figure.

© 2023 The Authors

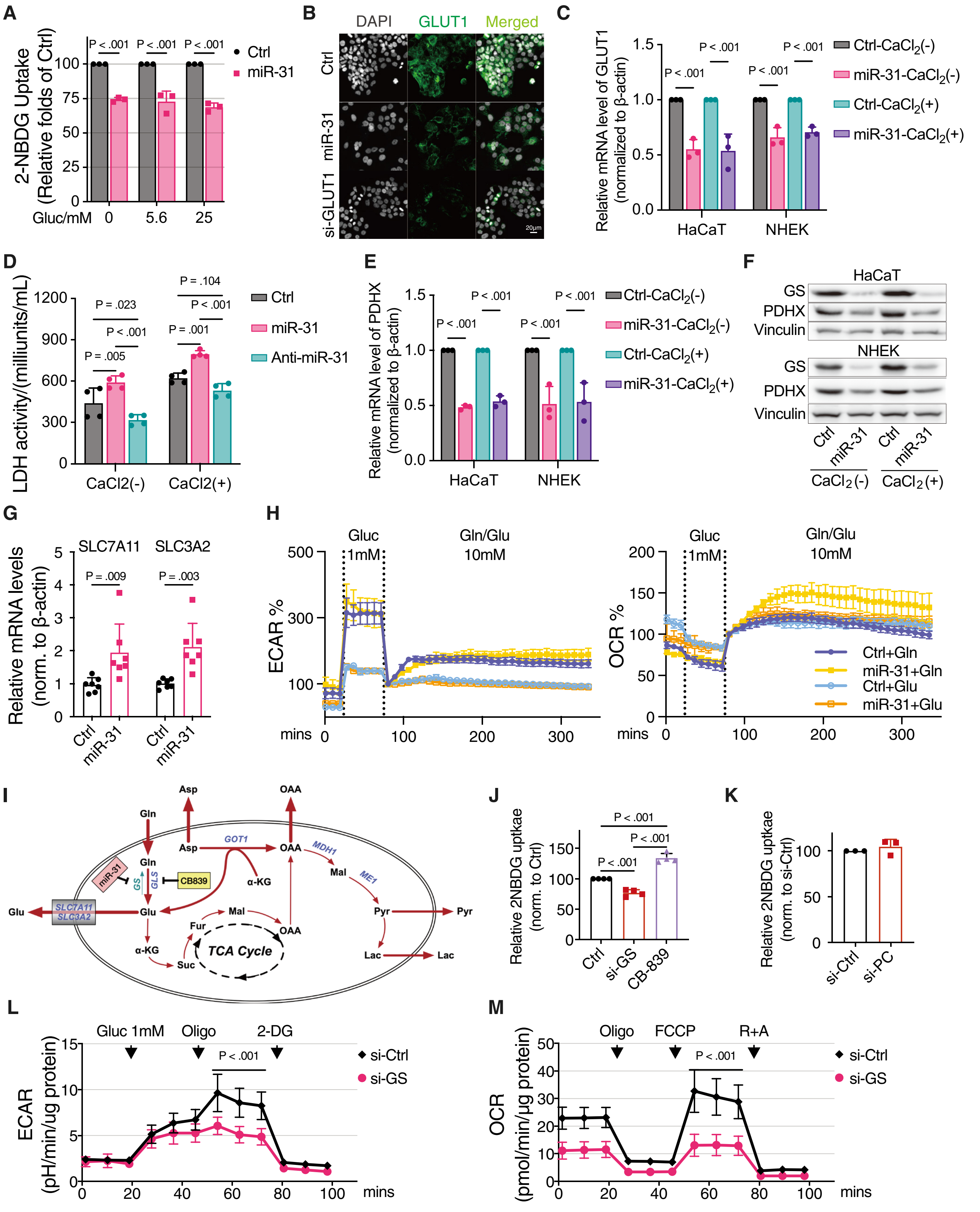
A
2-NBDG Uptake (Relative folds of Ctrl)
Ctrl
miR-31
P < .001
Gluc/mM 0 5.6 25
B
DAPI GLUT1 Merged
Ctrl
miR-31
si-GLUT1
20μm
C
Relative mRNA level of GLUT1 (normalized to β-actin)
Ctrl-CaCl2(-)
miR-31-CaCl2(-)
Ctrl-CaCl2(+)
miR-31-CaCl2(+)
HaCaT NHEK
D
LDH activity/(milliunits/mL)
Ctrl
miR-31
Anti-miR-31
P = .023
P < .001
P = .005
P = .104
P = .001
CaCl2(-) CaCl2(+)
E
Relative mRNA level of PDHX (normalized to β-actin)
F
HaCaT
NHEK
GS
PDHX
Vinculin
Ctrl miR-31 Ctrl miR-31
CaCl2(-) CaCl2(+)
G
Relative mRNA levels (norm. to β-actin)
SLC7A11 SLC3A2
P = .009
P = .003
H
Gluc 1mM
Gln/Glu 10mM
ECAR %
OCR %
mins
Ctrl+Gln
miR-31+Gln
Ctrl+Glu
miR-31+Glu
I
Asp
OAA
Gln
GOT1
MDH1
Mal
ME1
miR-31
GS
GLS
CB839
α-KG
Glu
SLC7A11
SLC3A2
Pyr
Fur
TCA Cycle
Suc
Lac
J
Relative 2NBDG uptkae (norm. to Ctrl)
P < .001
Ctrl si-GS CB-839
K
Relative 2NBDG uptkae (norm. to si-Ctrl)
si-Ctrl si-PC
L
Gluc 1mM Oligo 2-DG
ECAR (pH/min/ug protein)
si-Ctrl
si-GS
mins
M
Oligo FCCP R+A
OCR (pmol/min/μg protein)

Figure EV2.

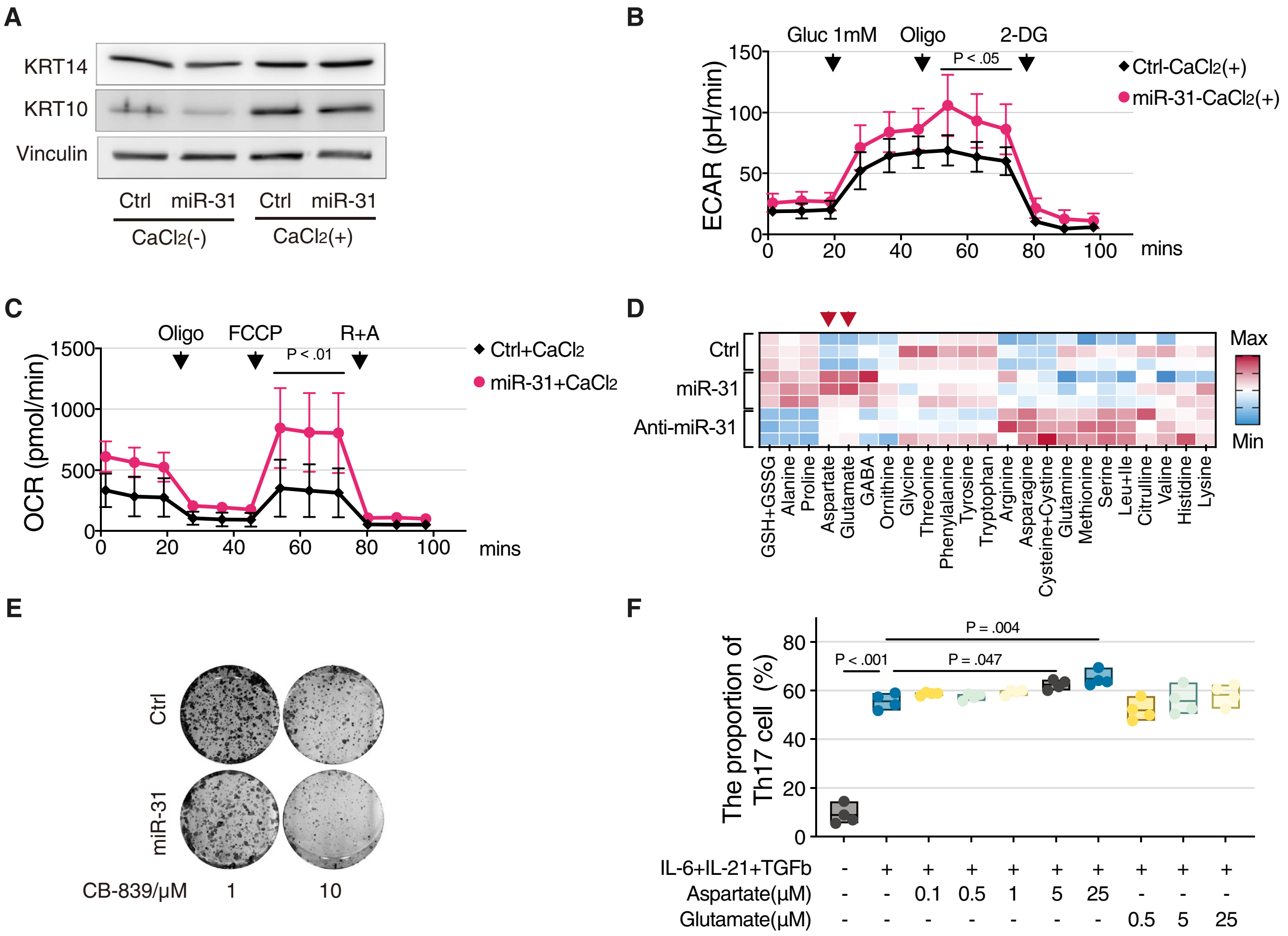

Figure EV3. Differentiated HaCaT cells show similar metabolism reprogramming upon miR-31 overexpression.

A Western blot analysis of KRT14 and KRT10 expressions in HaCaT cells cultured in medium supplemented with or without 2 mM CaCl2, and vinculin was used as loading control.
B Glycolysis stress test of differentiated HaCaT cells upon miR-31 overexpression in 1 mM glucose condition (n = 5 biological replicates).
C Mitochondrial stress test of differentiated HaCaT cells upon miR-31 overexpression in 1 mM glucose condition (n = 3 biological replicates).
D Heatmap representation of relative amino acid levels in cell culture medium of differentiated HaCaT cells upon miR-31 overexpression.
E Representative picture of cell colony-forming assay of HaCaT cells in response to CB839 treatment (n = 3 biological replicates).
F FACS analysis of Th17 differentiation upon glutamate or aspartate additions (n = 4 biological replicates).

Data information: In (B), (C), and (F), data are presented as mean ± SD (by two-way ANOVA with Sidak test for (B) and (C) and one-way ANOVA with Sidak test for (F)). Source data are available online for this figure.

 © 2023 The Authors

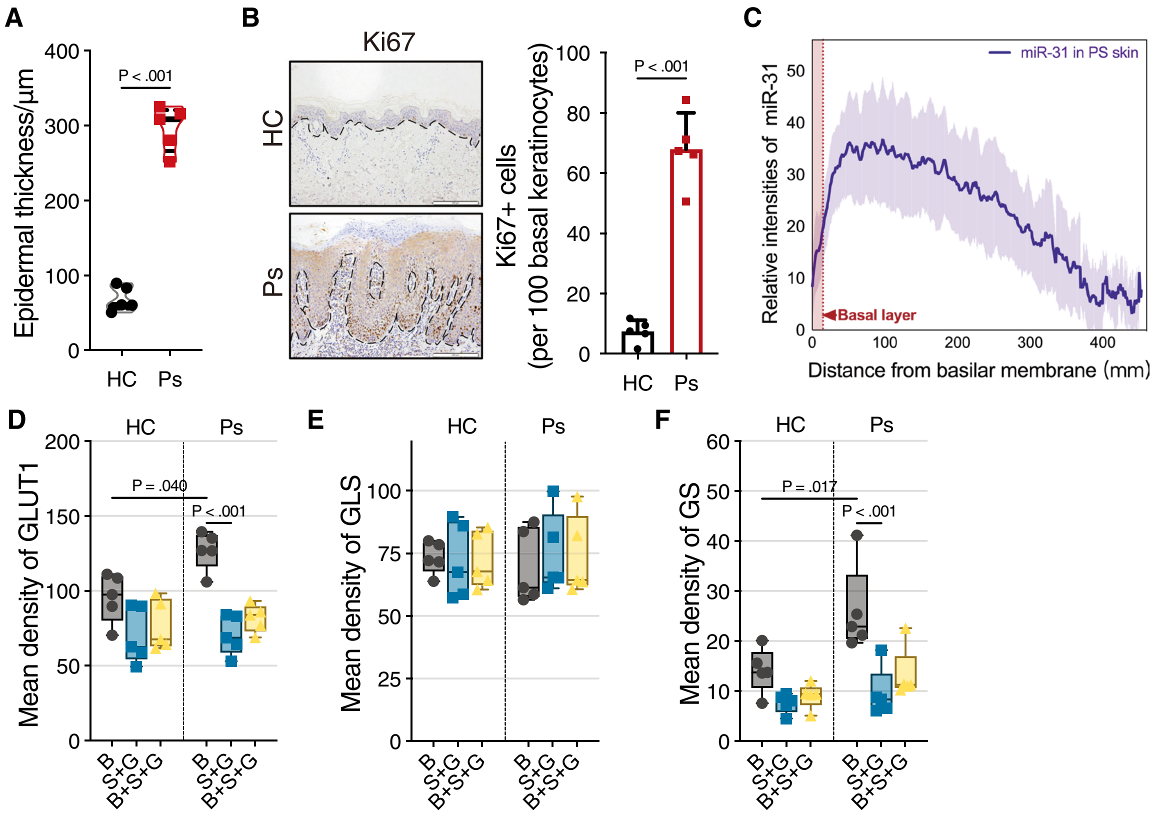

Figure EV4. Epidermal keratinocytes exhibit different metabolic characteristics *in vivo*.

A Violin plot of epidermal thickness of skin biopsies taken from healthy individuals and psoriasis patients ($n = 5$ individuals in each group).

B Representative pictures of immunohistochemical staining of Ki-67 in skin biopsies of HC and Ps. Scale bars: 200 µm. Quantification of result is shown on right panel, $n = 5$ biological replicates.

C Quantification of result of miR-31 ISH data of skin biopsies from psoriasis patients ($n = 5$ biological replicates). The continuous line represents the mean intensity of miR-31, and the shaded area means the standard deviation.

D–F Quantification of results of GLUT1(D), GLS(E), and GS(F) staining of human skin biopsies ($n = 5$ biological replicates). S + G layers: spinous layer and granular layer of epidermis; B + S + G layers: basal layer, spinous layer, and granular layer.

Data information: Data are presented as truncated violin plot in (A), mean ± SD in (B) and (C), and box and whiskers (central band, median; whiskers, min to max and show all points) in (D–F) (by unpaired Student's *t*-test for (A) and (B), One-way ANOVA with Sidak test for (D–F)).
Source data are available online for this figure.

Figure EV5. Blocking the miR-31 induced metabolic changes relieves psoriatic disease in a mouse model.

A Representations of *in situ* hybridization staining (ISH) of miR-31 in skin biopsies from control mice (Ctrl, $n = 3$ mice) and imiquimod-induced psoriatic mice (IMQ, $n = 6$ mice). Quantification result showing the average level of miR-31 in epidermis (right panel). B, basal layer; S + G, spinous and granular layers. Scale bars: 100 µm.

B–D Disease activity was evaluated by epidermal thickness (B), Baker's score (C), and Ki-67-positive cell count (D). $n = 4$ mice in Ctrl and CBG groups and $n = 5$ mice in IMQ and CB-839 groups.

E–G Representative pictures of immunohistochemistry staining of CD4 (upper panel) and MPO (bottom panel) in skin biopsies from mice ($n = 4$ mice in Ctrl and CBG groups and $n = 5$ mice in IMQ and CB-839 groups). Scale bars: 100 µm. Quantification results showing the average number of CD4-positive or MPO-positive cells in skin sections (F and G, respectively).

H–K IHC staining of GLUT1, GLS, and GS in skin from mice with different treatments (H, scale bars: 25 µm). I–K present the quantification results of GLUT1, GLS, and GS, respectively. $n = 4$ mice in Ctrl and CBG groups and $n = 5$ mice in IMQ and CB-839 groups.

L Enzyme-linked immunosorbent assay for the determination of IL-10 levels in serum of mice ($n = 5$ mice in vehicle group and $n = 6$ mice in other groups).

Data information: In (A), (F), and (G), data are presented as mean ± SD. In (B–D) and (I–L), data are presented as box and whiskers (min to max; by unpaired Student's *t*-test for (A) and one-way ANOVA with Sidak test for (B–D) and (I–L)).
Source data are available online for this figure.

© 2023 The Authors

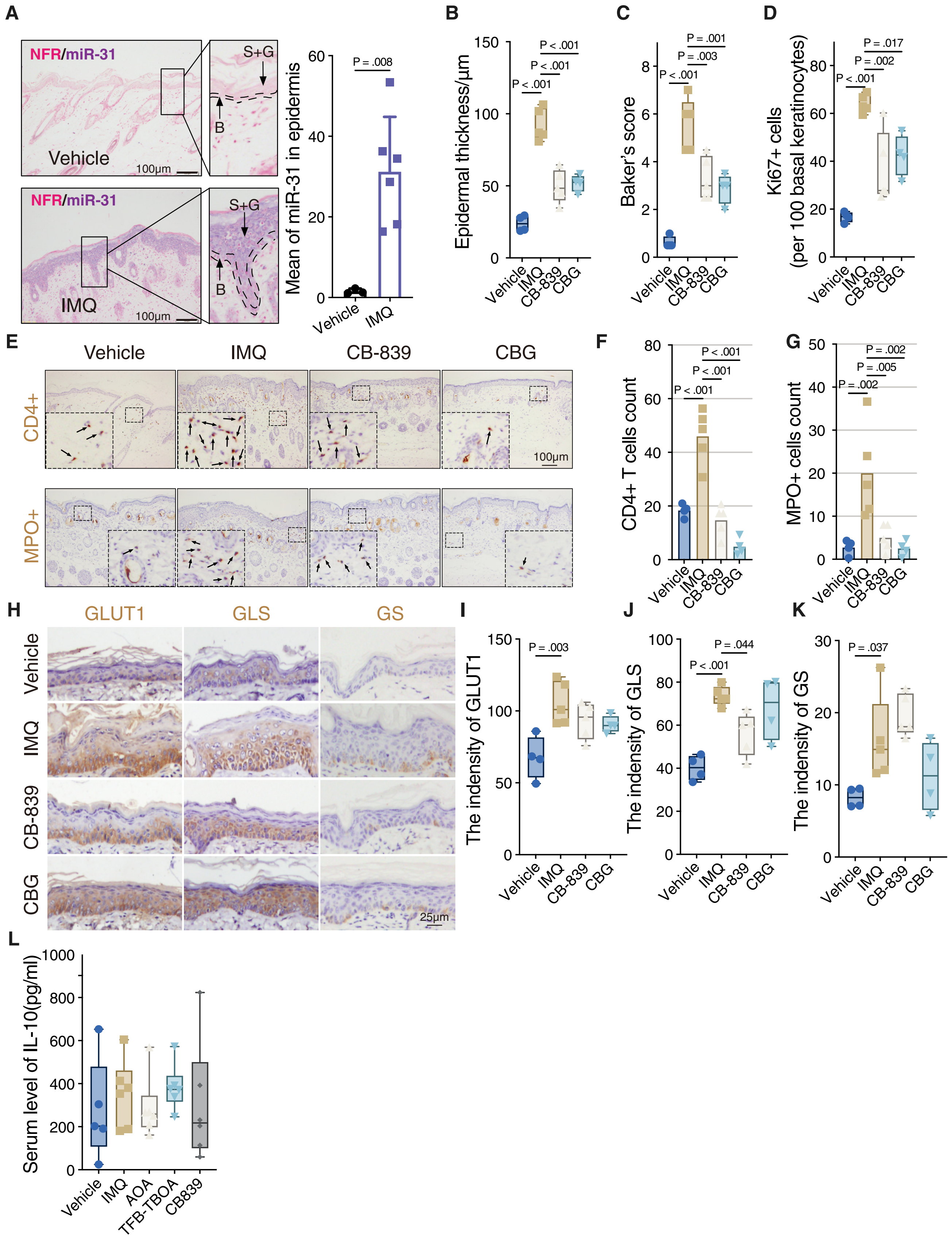

Figure EV5.

 © 2023 The Authors
