# Supplementary figures and images for: Metabolic rewiring in keratinocytes by miR‐31‐5p identifies therapeutic intervention for psoriasis
Source: EMBO Mol Med. 2023 Mar 1;15(4):e15674. doi: 10.15252/emmm.202215674 (PMC10086589; doi:10.15252/emmm.202215674)

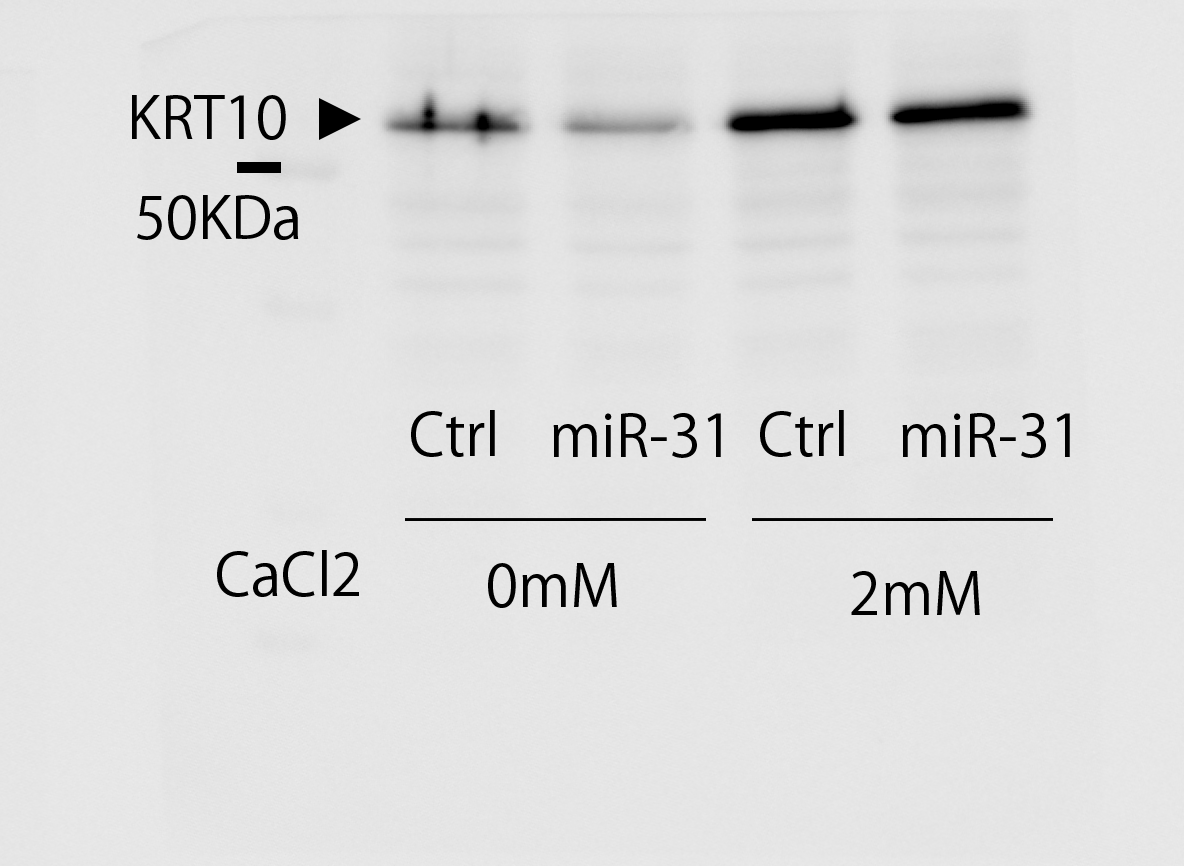

Supplement: Supplementary file 10 — Source Data for Expanded View [file EMMM-15-e15674-s014.zip › Figure Source Data File of Figure EV/Figure EV 3/3A/3A- western blot of KRT10 .tif]

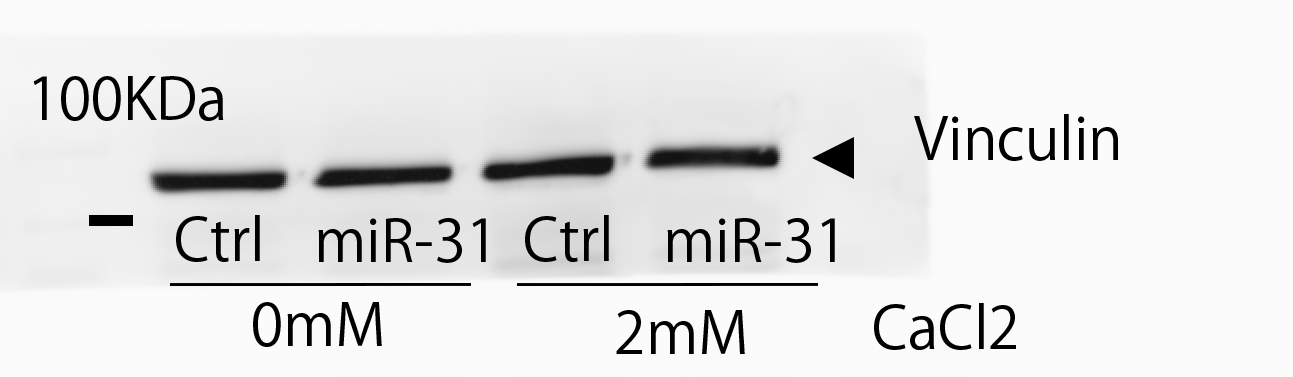

Supplement: Supplementary file 10 — Source Data for Expanded View [file EMMM-15-e15674-s014.zip › Figure Source Data File of Figure EV/Figure EV 3/3A/3A- western blot of Vinculin .tif]

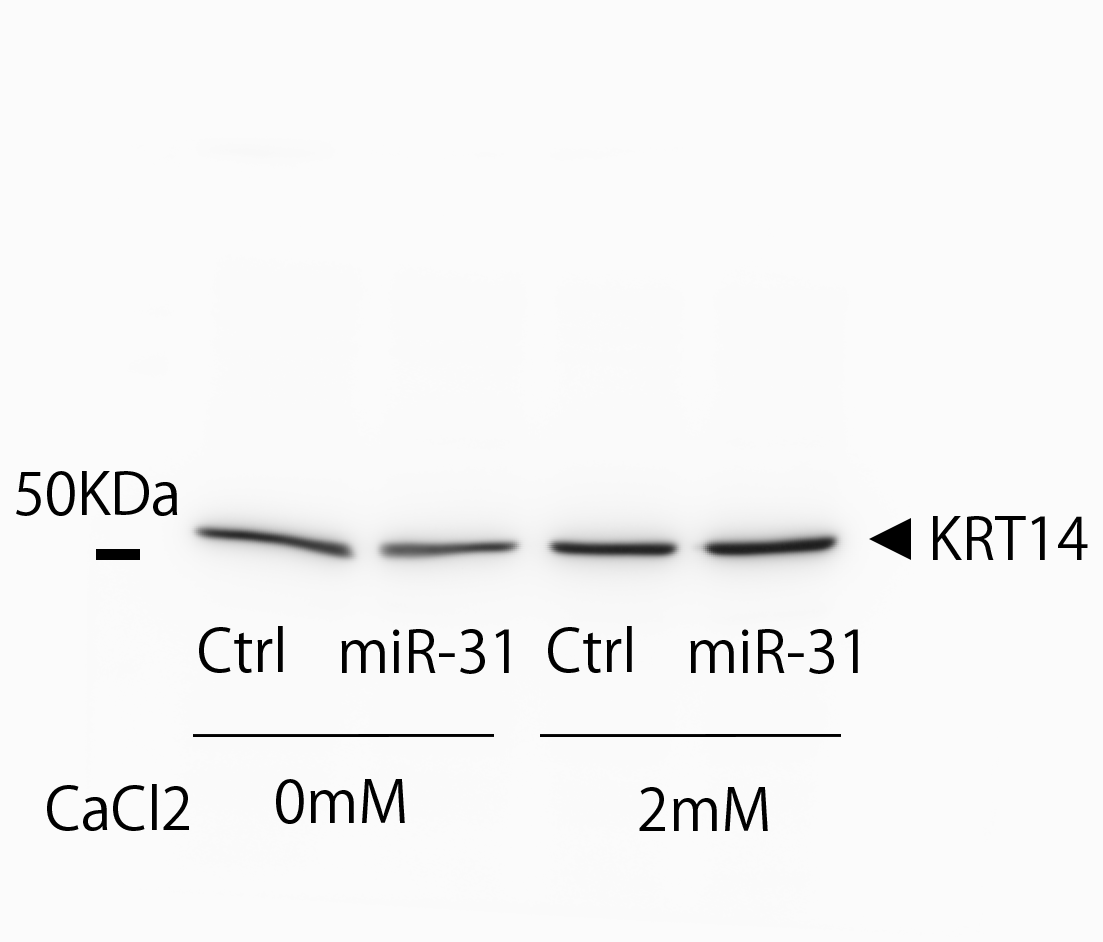

Supplement: Supplementary file 10 — Source Data for Expanded View [file EMMM-15-e15674-s014.zip › Figure Source Data File of Figure EV/Figure EV 3/3A/3A- western blot of KRT14 .tif]

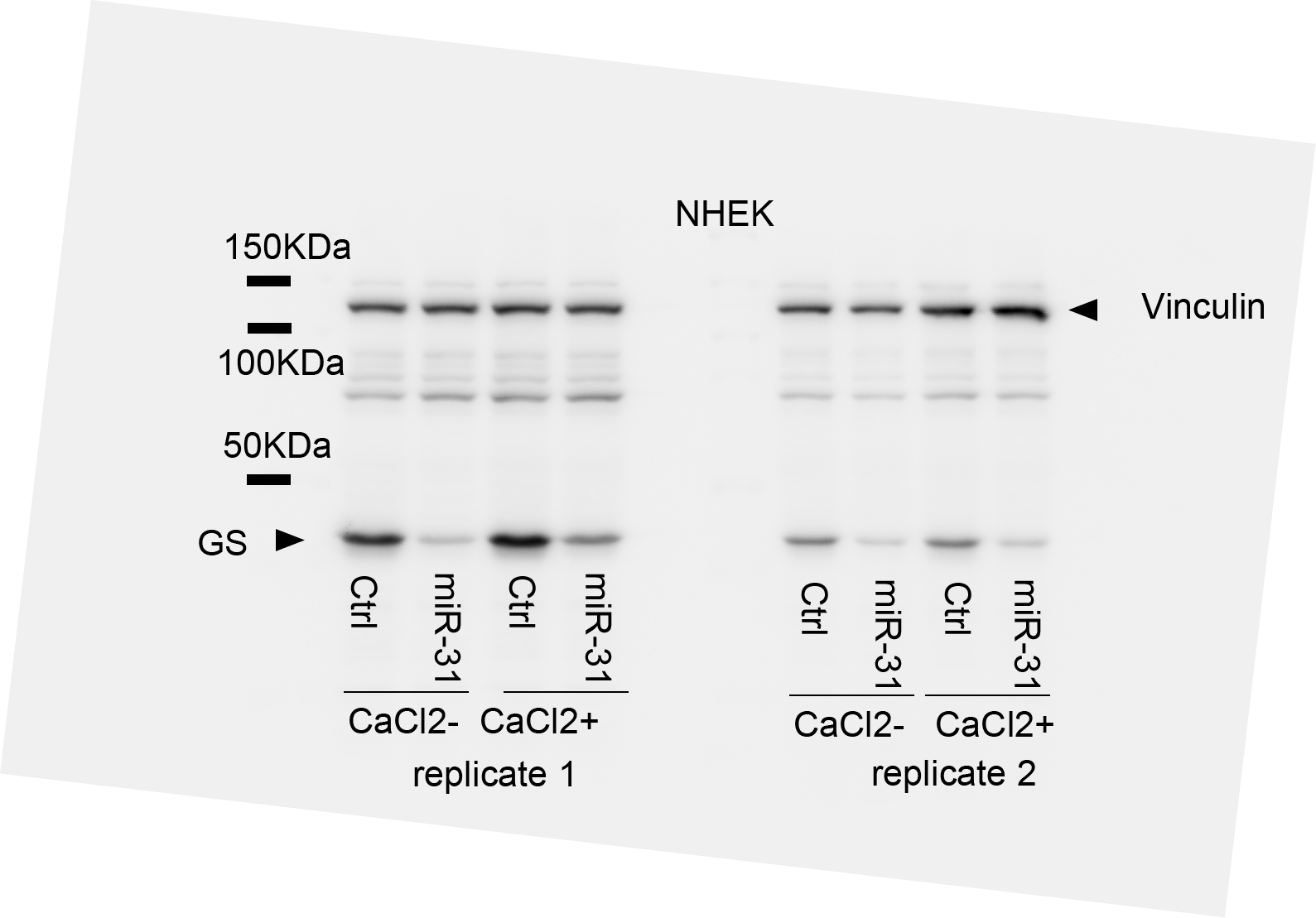

Supplement: Supplementary file 10 — Source Data for Expanded View [file EMMM-15-e15674-s014.zip › Figure Source Data File of Figure EV/Figure EV 2/2F/2F-western blot of vinculin for GS of NHEK-replicate 1 and 2.tif]

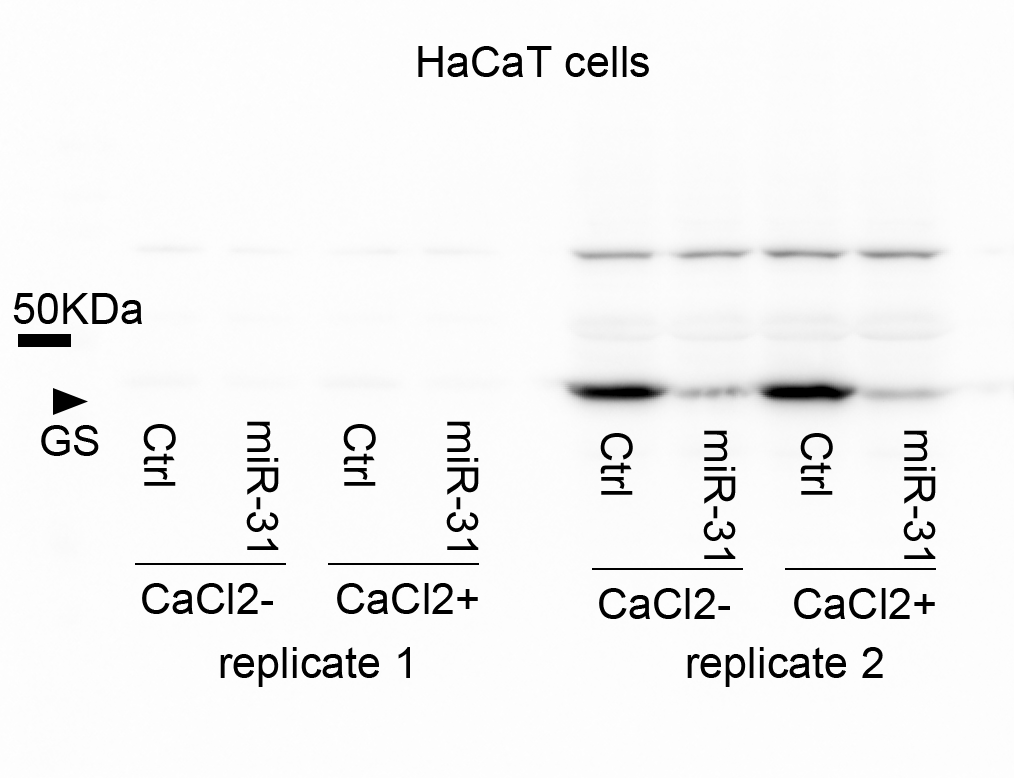

Supplement: Supplementary file 10 — Source Data for Expanded View [file EMMM-15-e15674-s014.zip › Figure Source Data File of Figure EV/Figure EV 2/2F/2F-western blot of GS of HaCaT-replicate 1 and 2.tif]

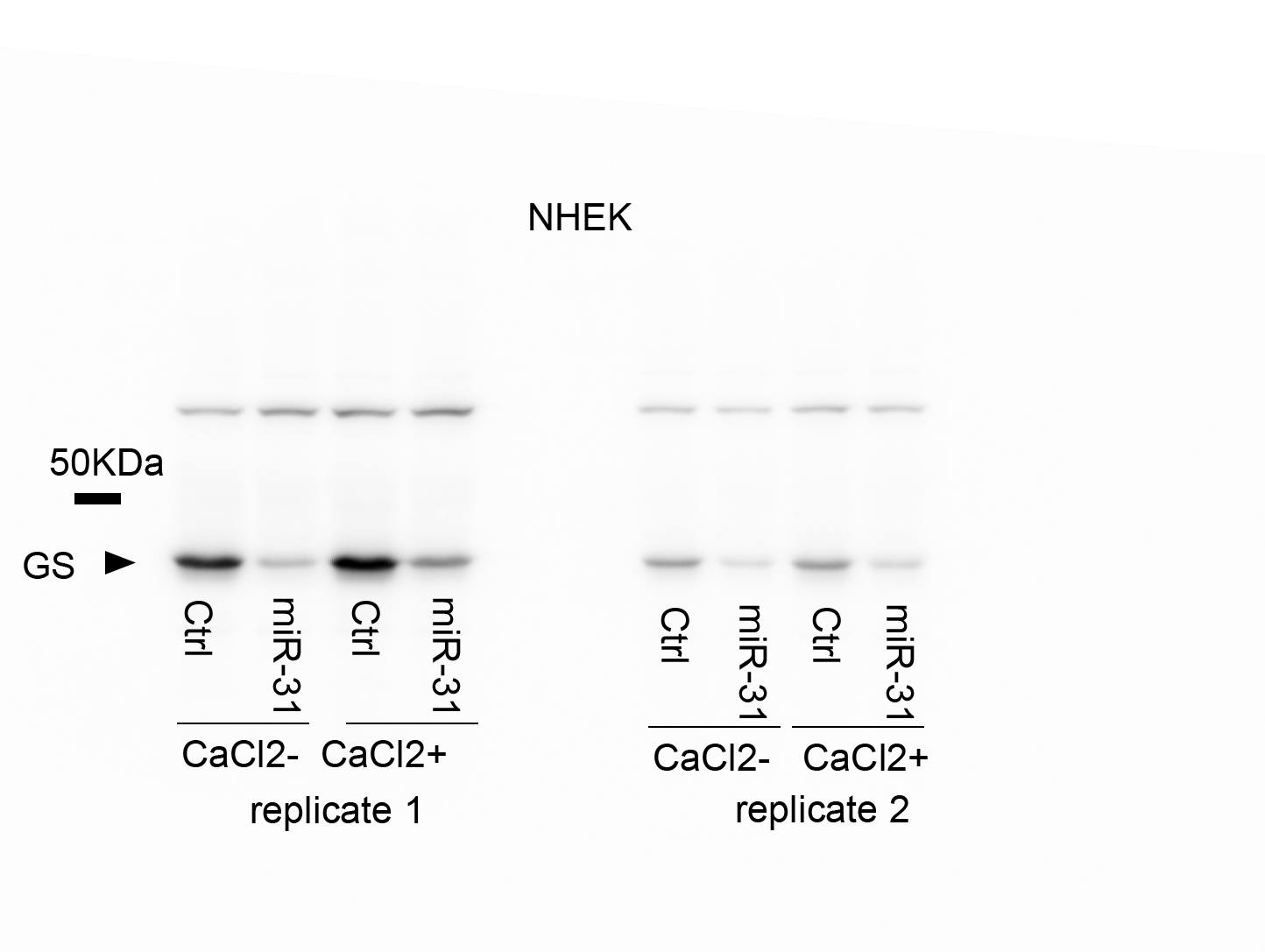

Supplement: Supplementary file 10 — Source Data for Expanded View [file EMMM-15-e15674-s014.zip › Figure Source Data File of Figure EV/Figure EV 2/2F/2F-western blot of GS of NHEK-replicate 1 and 2.tif]

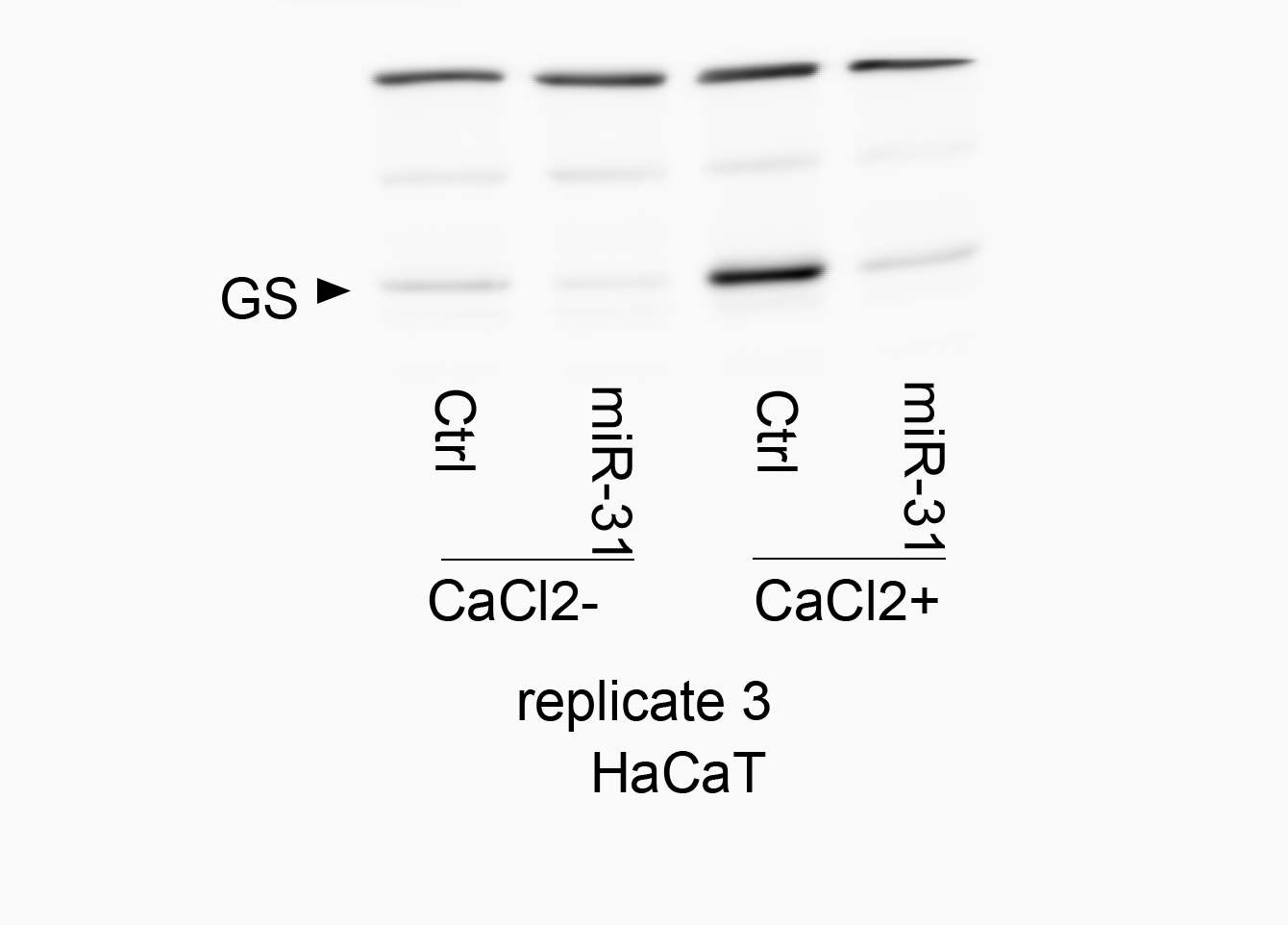

Supplement: Supplementary file 10 — Source Data for Expanded View [file EMMM-15-e15674-s014.zip › Figure Source Data File of Figure EV/Figure EV 2/2F/2F-western blot of GS of HaCaT-replicate 3.tif]

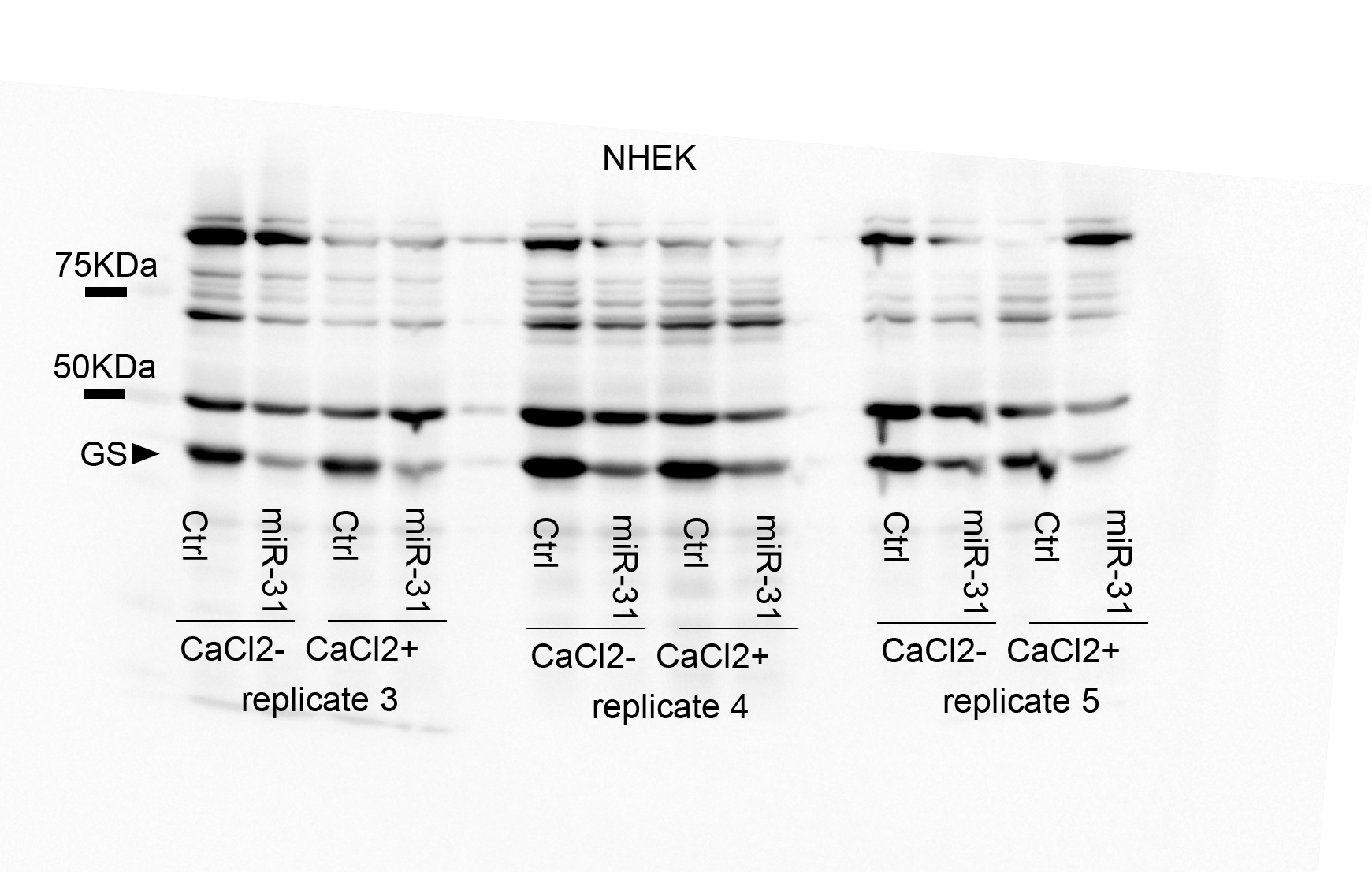

Supplement: Supplementary file 10 — Source Data for Expanded View [file EMMM-15-e15674-s014.zip › Figure Source Data File of Figure EV/Figure EV 2/2F/2F-western blot of GS of NHEK-replicate 3-5.tif]

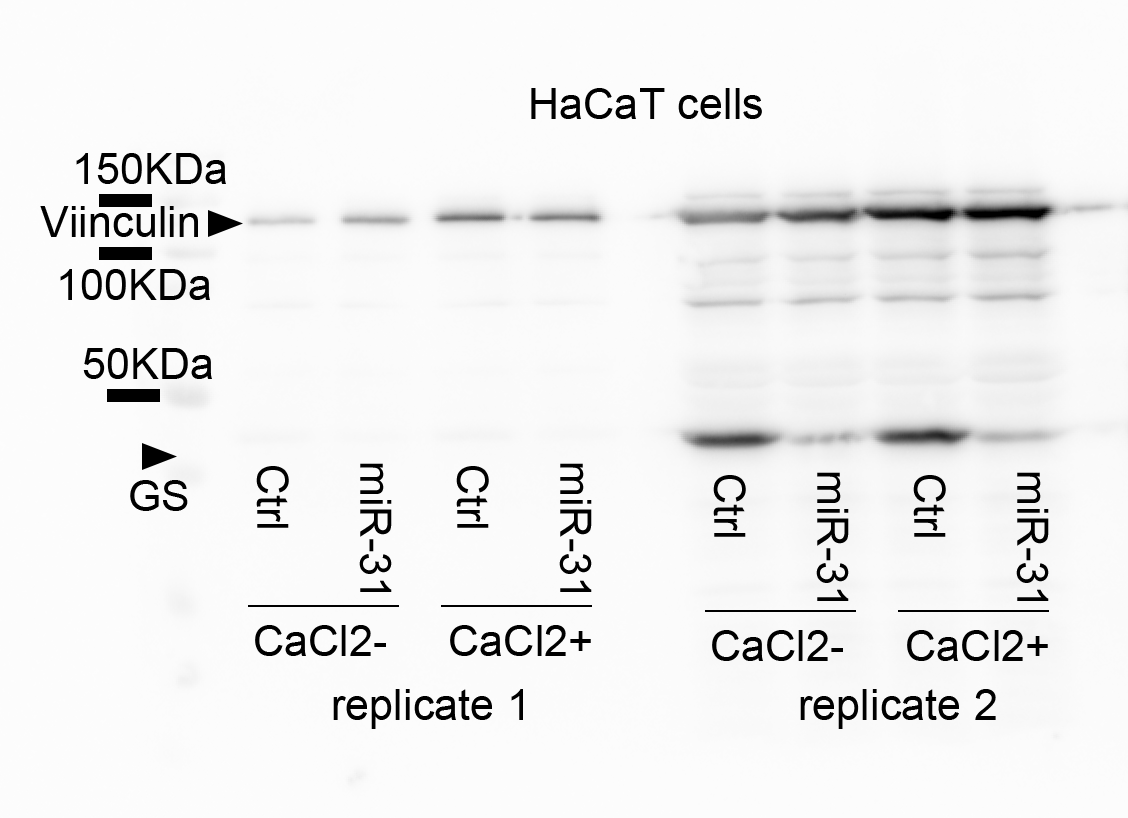

Supplement: Supplementary file 10 — Source Data for Expanded View [file EMMM-15-e15674-s014.zip › Figure Source Data File of Figure EV/Figure EV 2/2F/2F-western blot of vinculin for GS of HaCaT-replicate 1 and 2.tif]

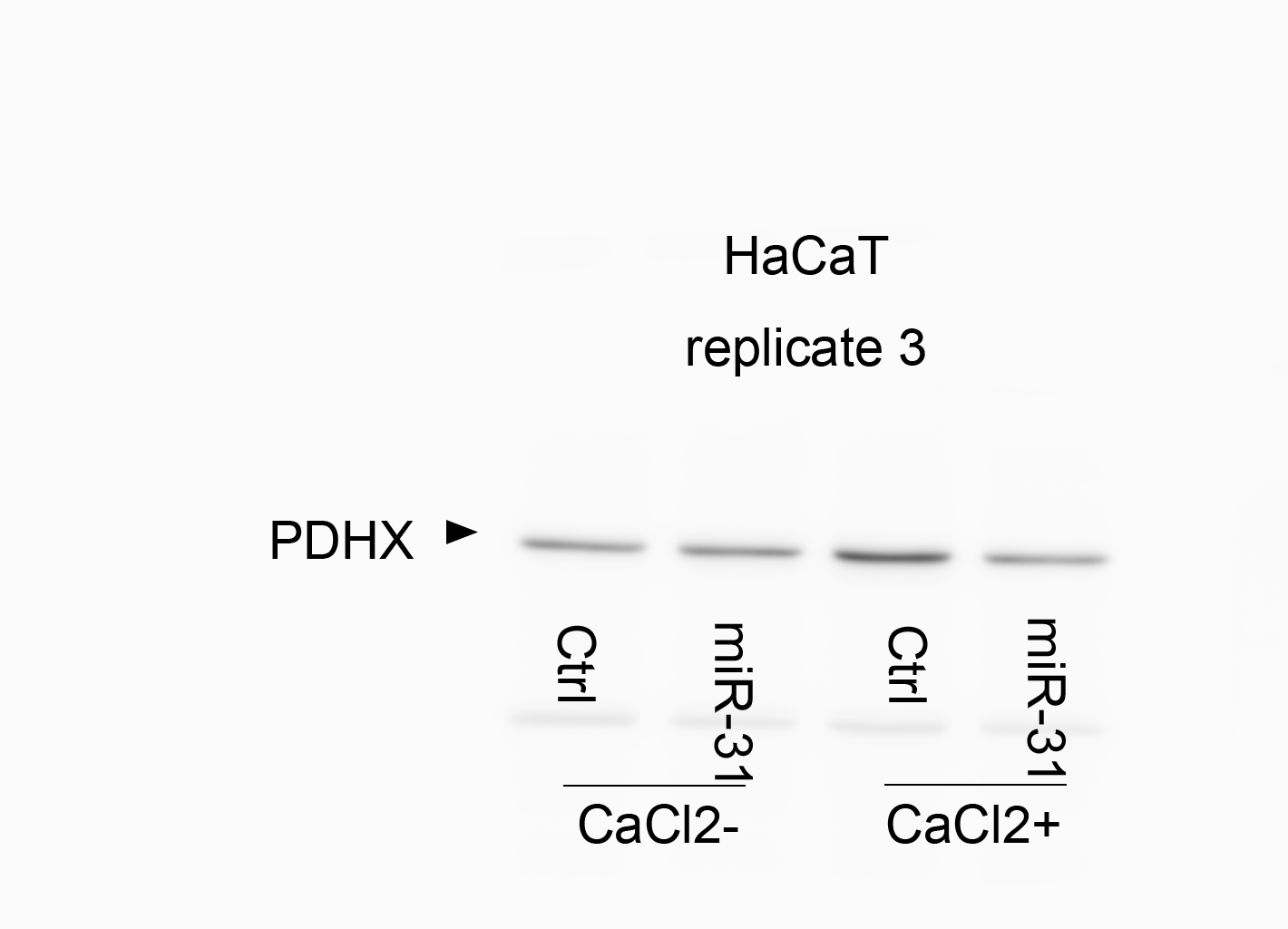

Supplement: Supplementary file 10 — Source Data for Expanded View [file EMMM-15-e15674-s014.zip › Figure Source Data File of Figure EV/Figure EV 2/2F/2F-western blot of PDHX of HaCaT-replicate 3.tif]

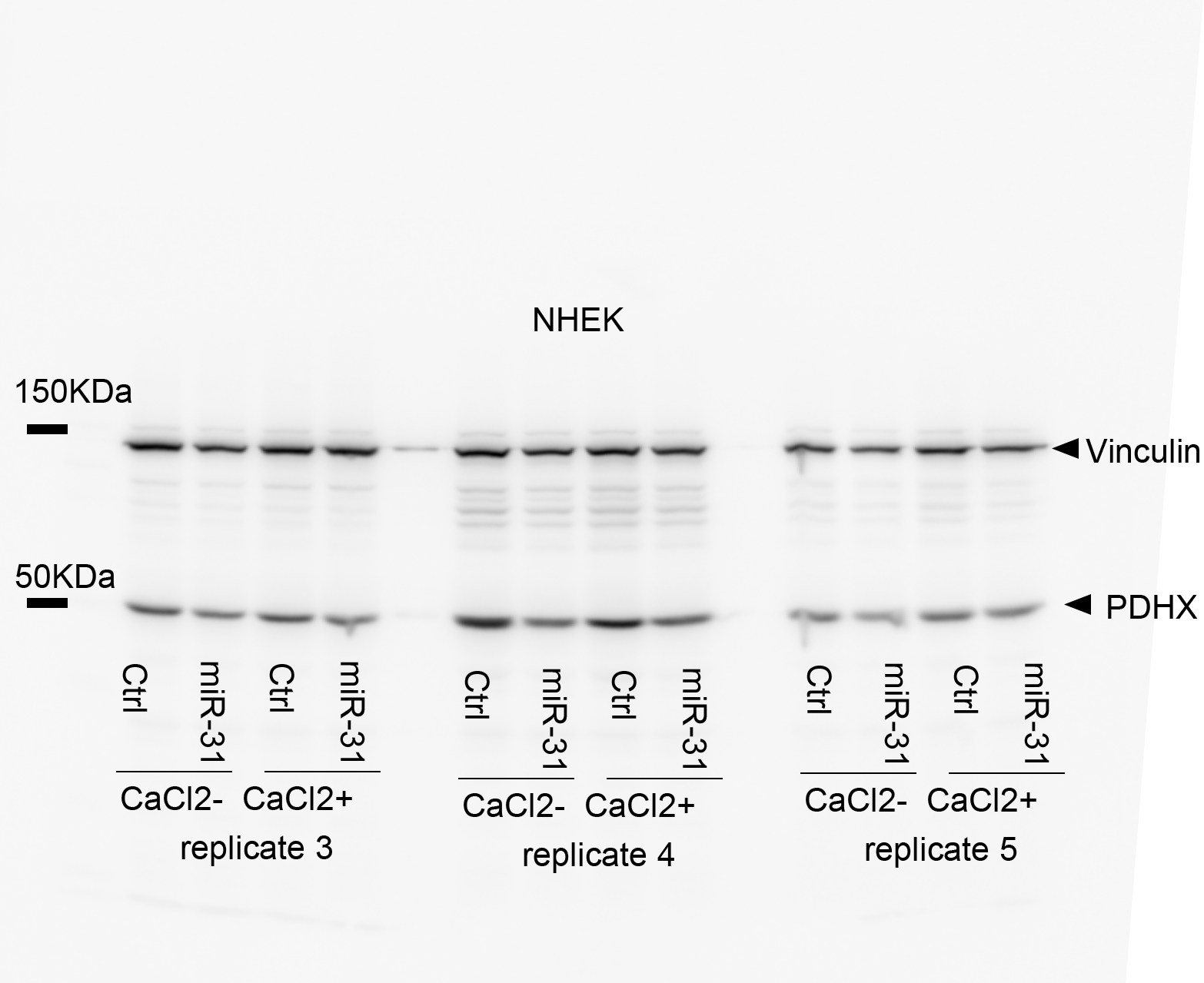

Supplement: Supplementary file 10 — Source Data for Expanded View [file EMMM-15-e15674-s014.zip › Figure Source Data File of Figure EV/Figure EV 2/2F/2F-western blot of vinculin and PDHX of NHEK-replicate 3-5.tif]

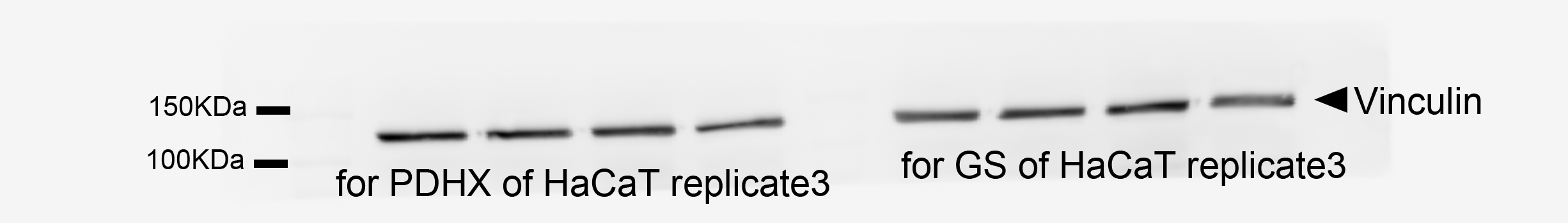

Supplement: Supplementary file 10 — Source Data for Expanded View [file EMMM-15-e15674-s014.zip › Figure Source Data File of Figure EV/Figure EV 2/2F/2F-western blot of Vinculin of HaCaT-replicate 3.tif]

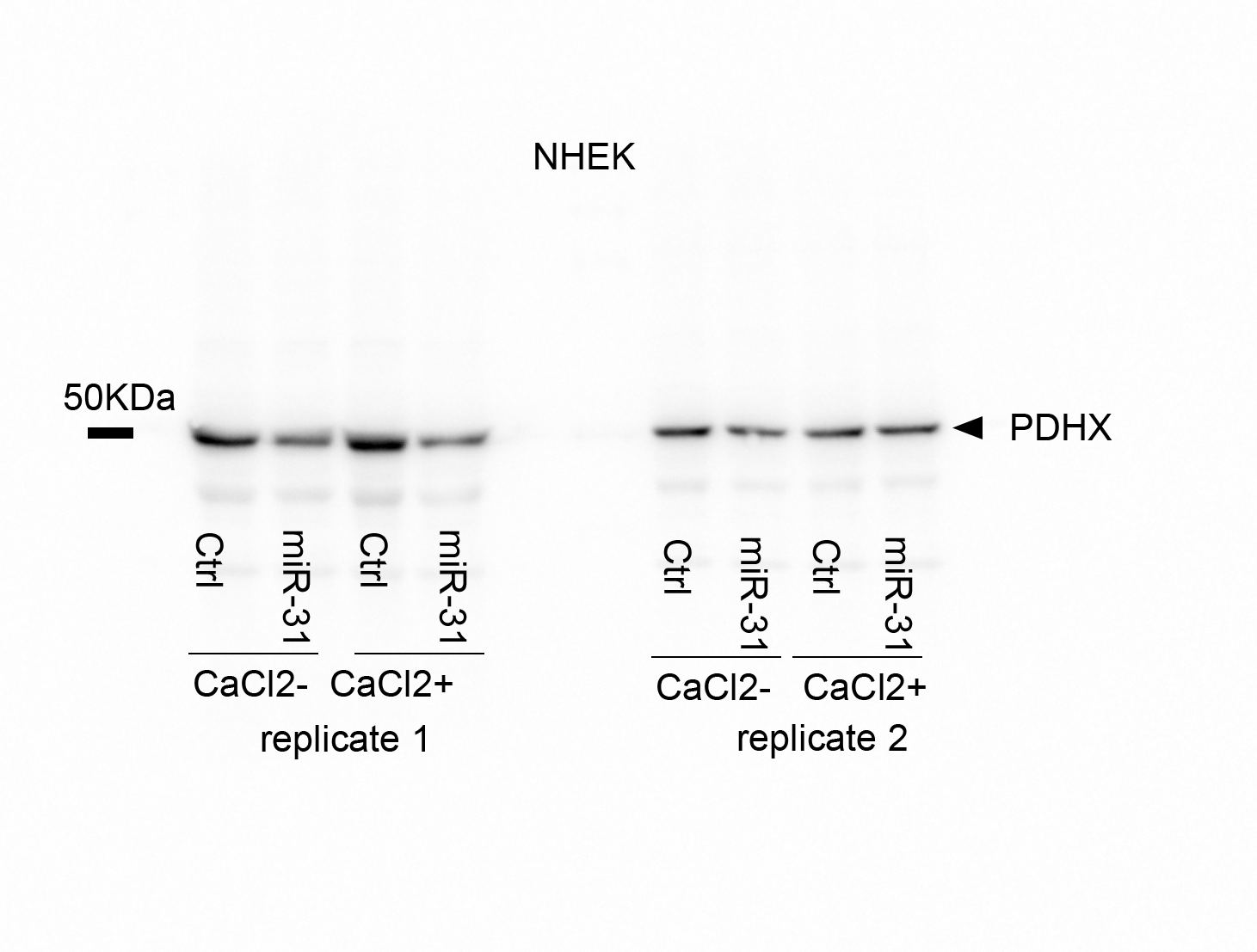

Supplement: Supplementary file 10 — Source Data for Expanded View [file EMMM-15-e15674-s014.zip › Figure Source Data File of Figure EV/Figure EV 2/2F/2F-western blot of PDHX of NHEK-replicate 1 and 2.tif]

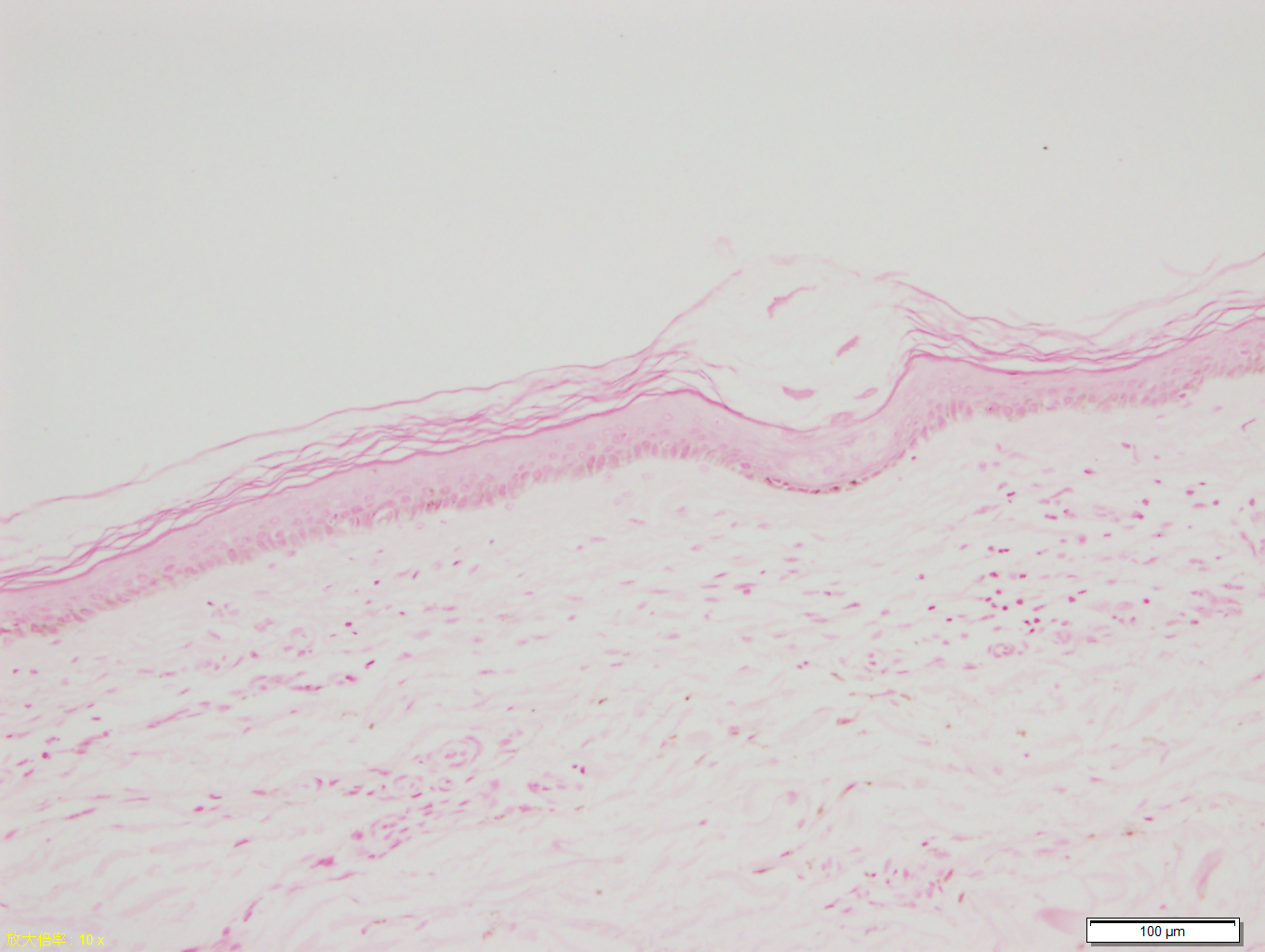

Supplement: Supplementary file 10 — Source Data for Expanded View [file EMMM-15-e15674-s014.zip › Figure Source Data File of Figure EV/Figure EV 5/5A/Ctrl-1.tif]

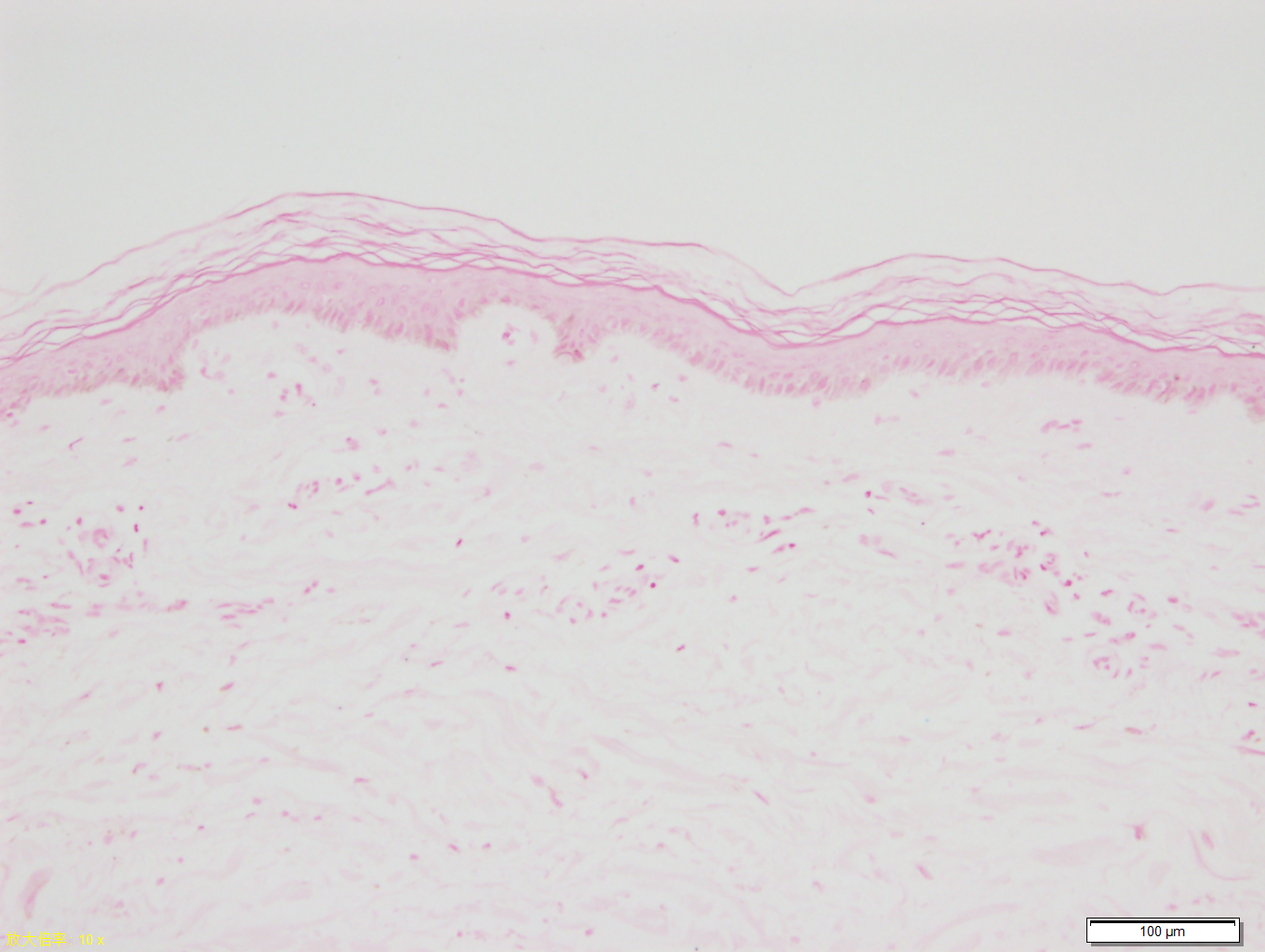

Supplement: Supplementary file 10 — Source Data for Expanded View [file EMMM-15-e15674-s014.zip › Figure Source Data File of Figure EV/Figure EV 5/5A/Ctrl-3.tif]

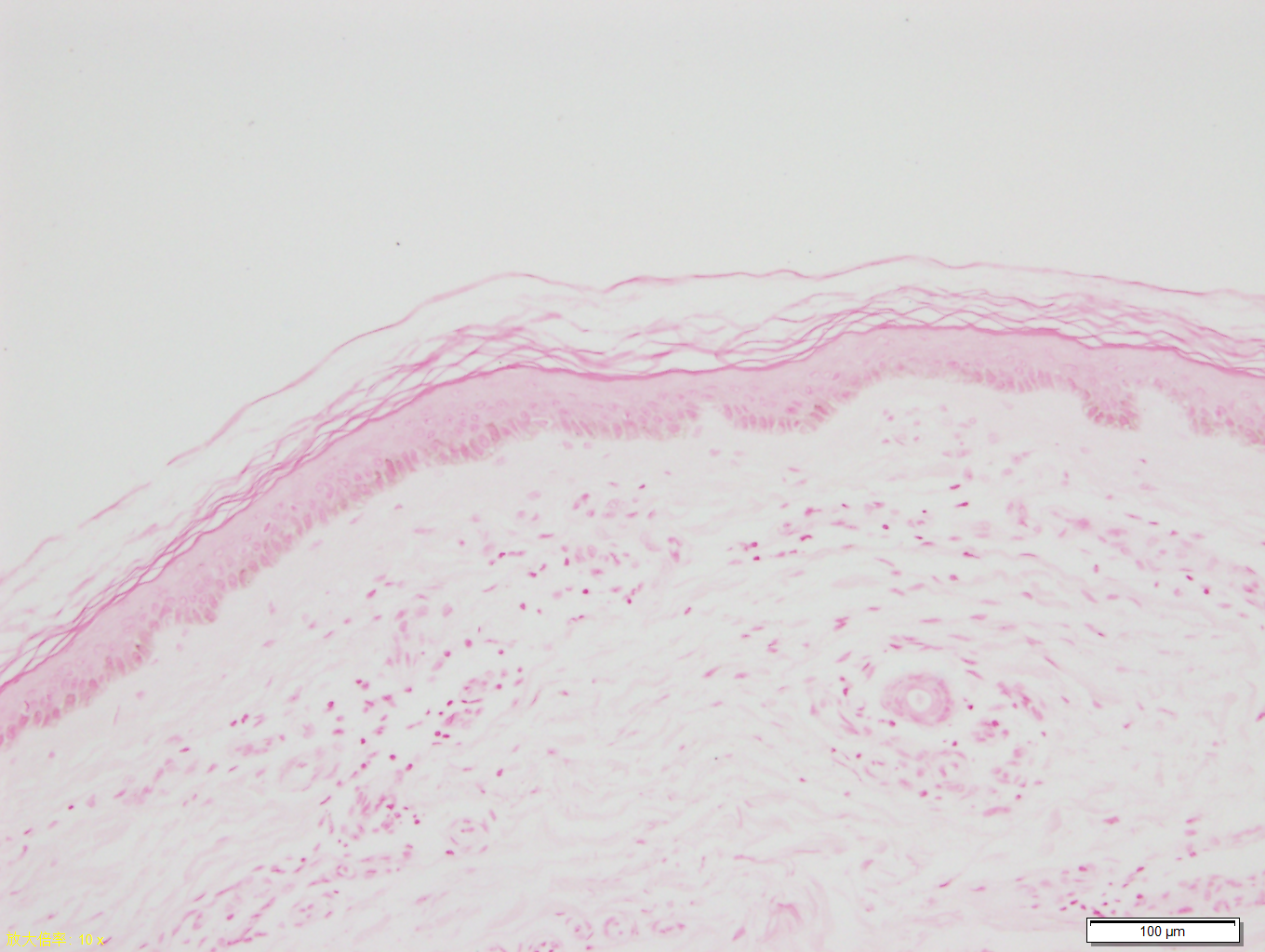

Supplement: Supplementary file 10 — Source Data for Expanded View [file EMMM-15-e15674-s014.zip › Figure Source Data File of Figure EV/Figure EV 5/5A/Ctrl-2.tif]

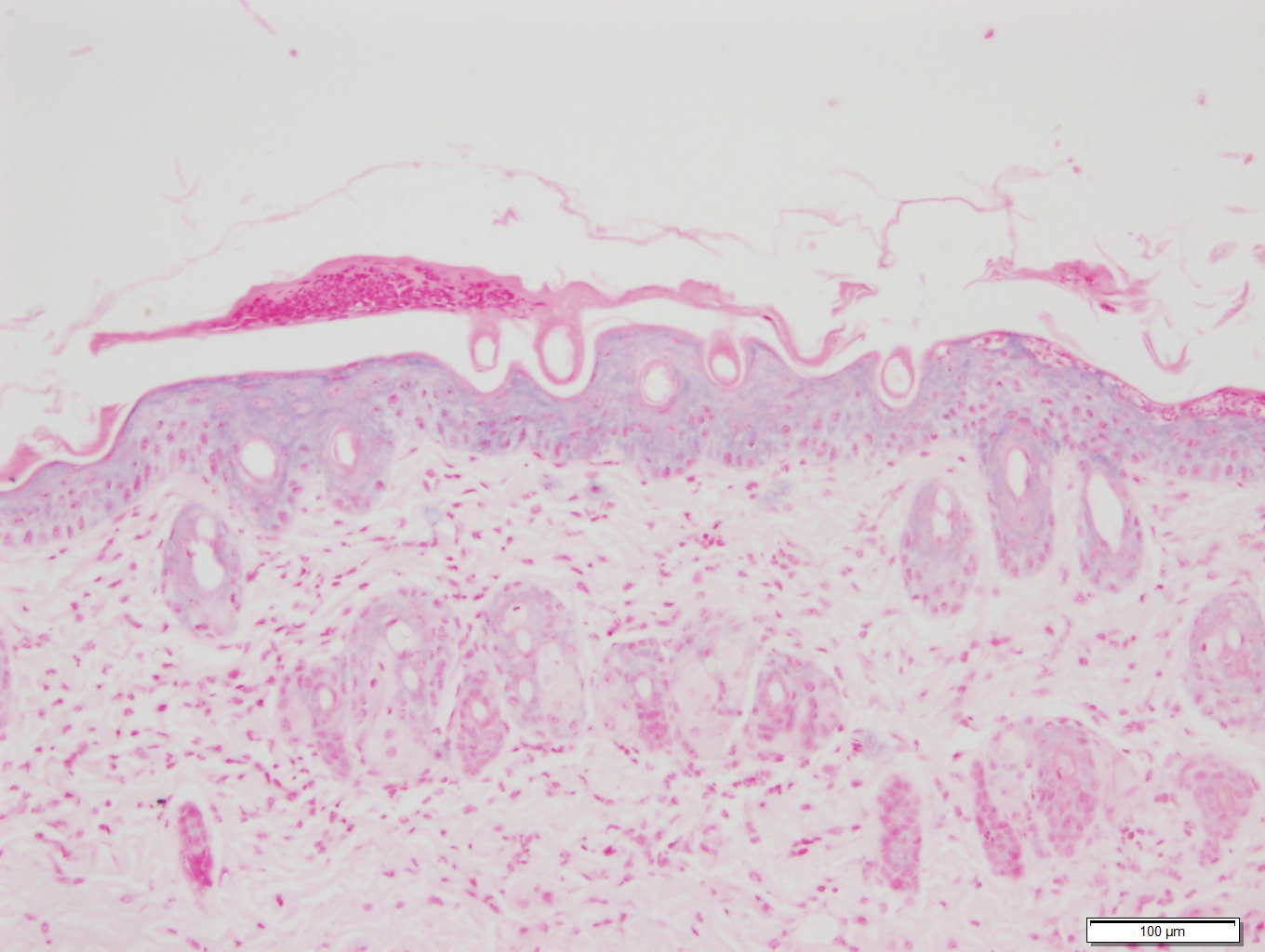

Supplement: Supplementary file 10 — Source Data for Expanded View [file EMMM-15-e15674-s014.zip › Figure Source Data File of Figure EV/Figure EV 5/5A/IMQ-3.tif]

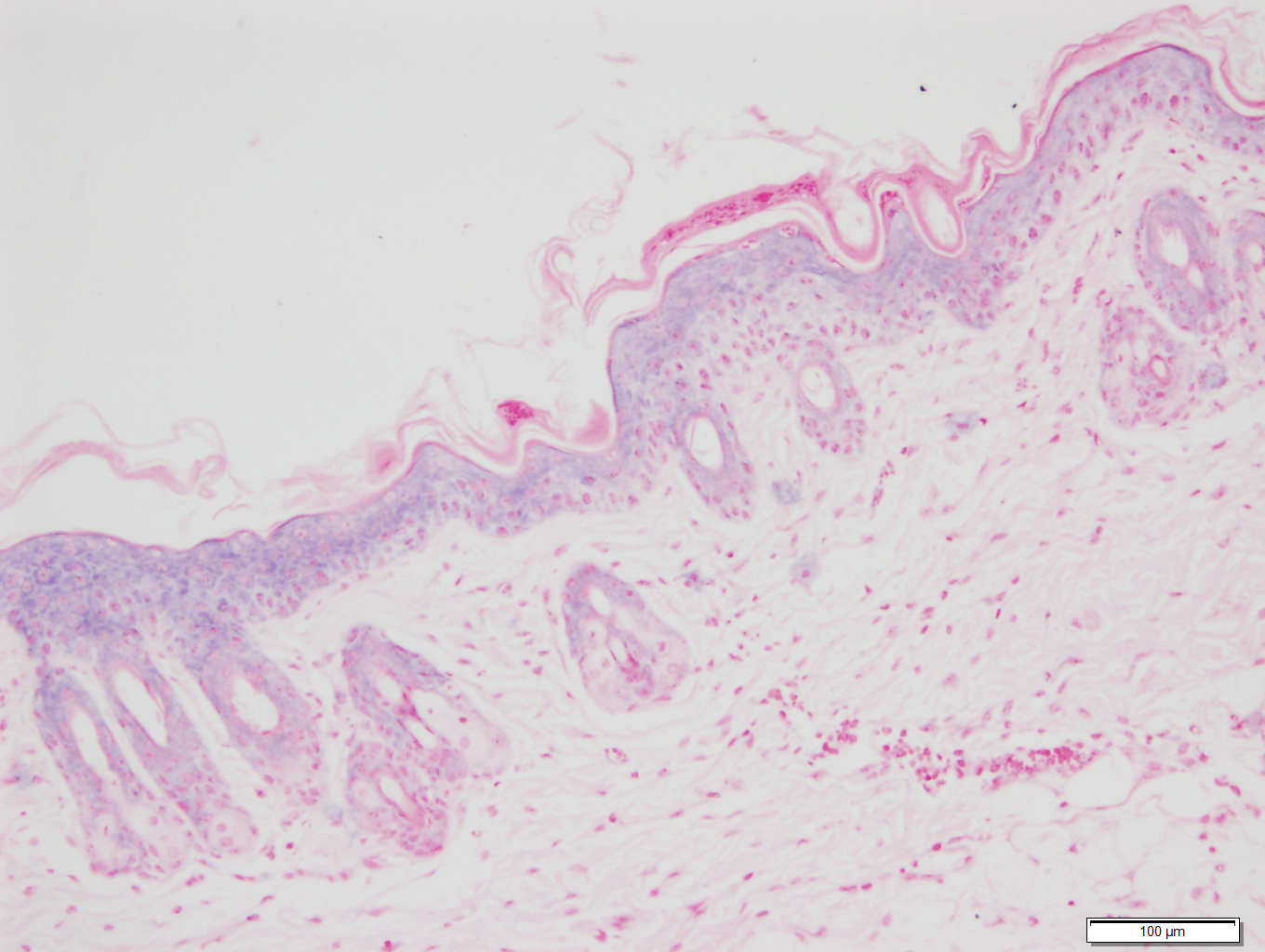

Supplement: Supplementary file 10 — Source Data for Expanded View [file EMMM-15-e15674-s014.zip › Figure Source Data File of Figure EV/Figure EV 5/5A/IMQ-2.tif]

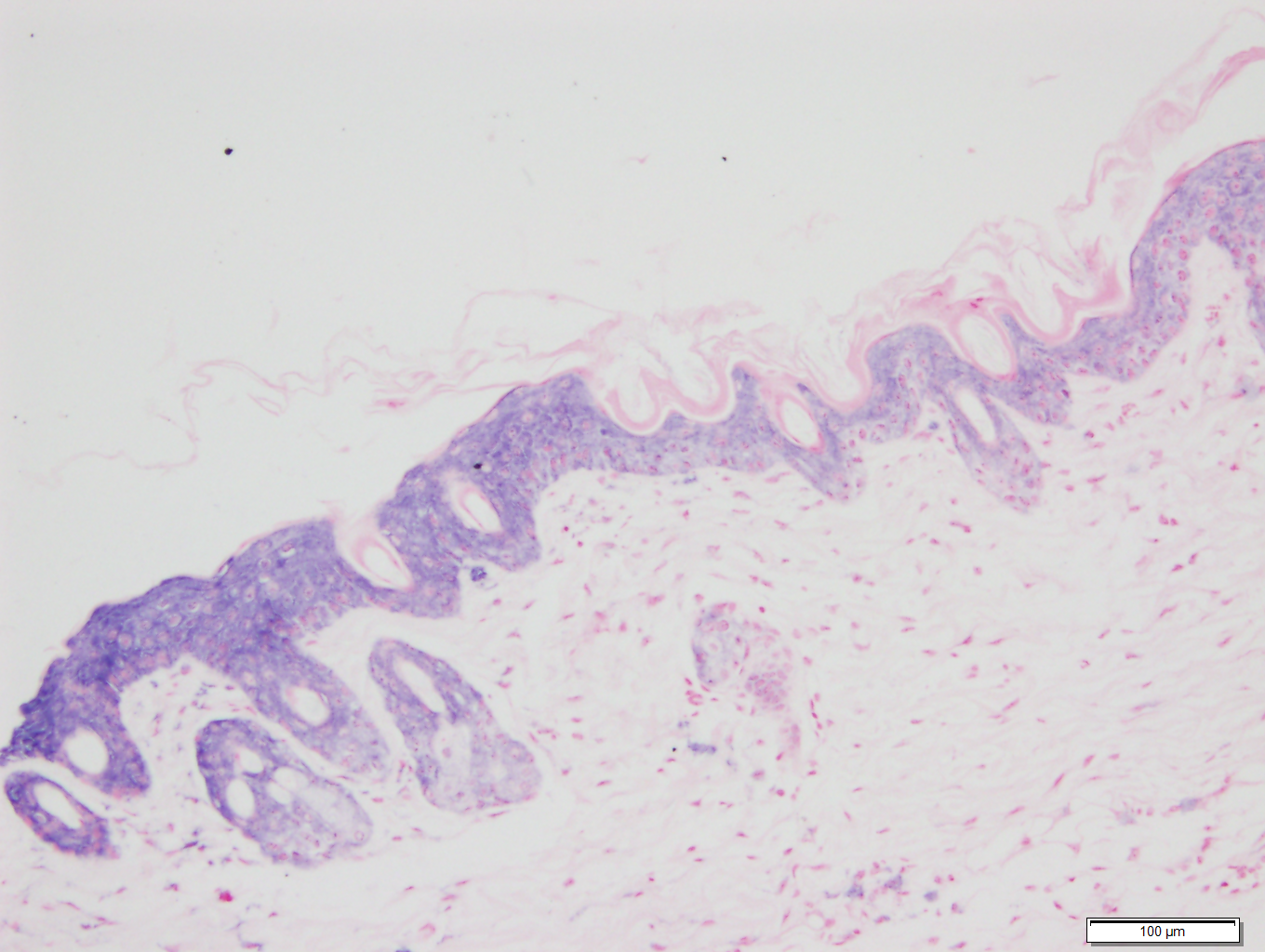

Supplement: Supplementary file 10 — Source Data for Expanded View [file EMMM-15-e15674-s014.zip › Figure Source Data File of Figure EV/Figure EV 5/5A/IMQ-1.tif]

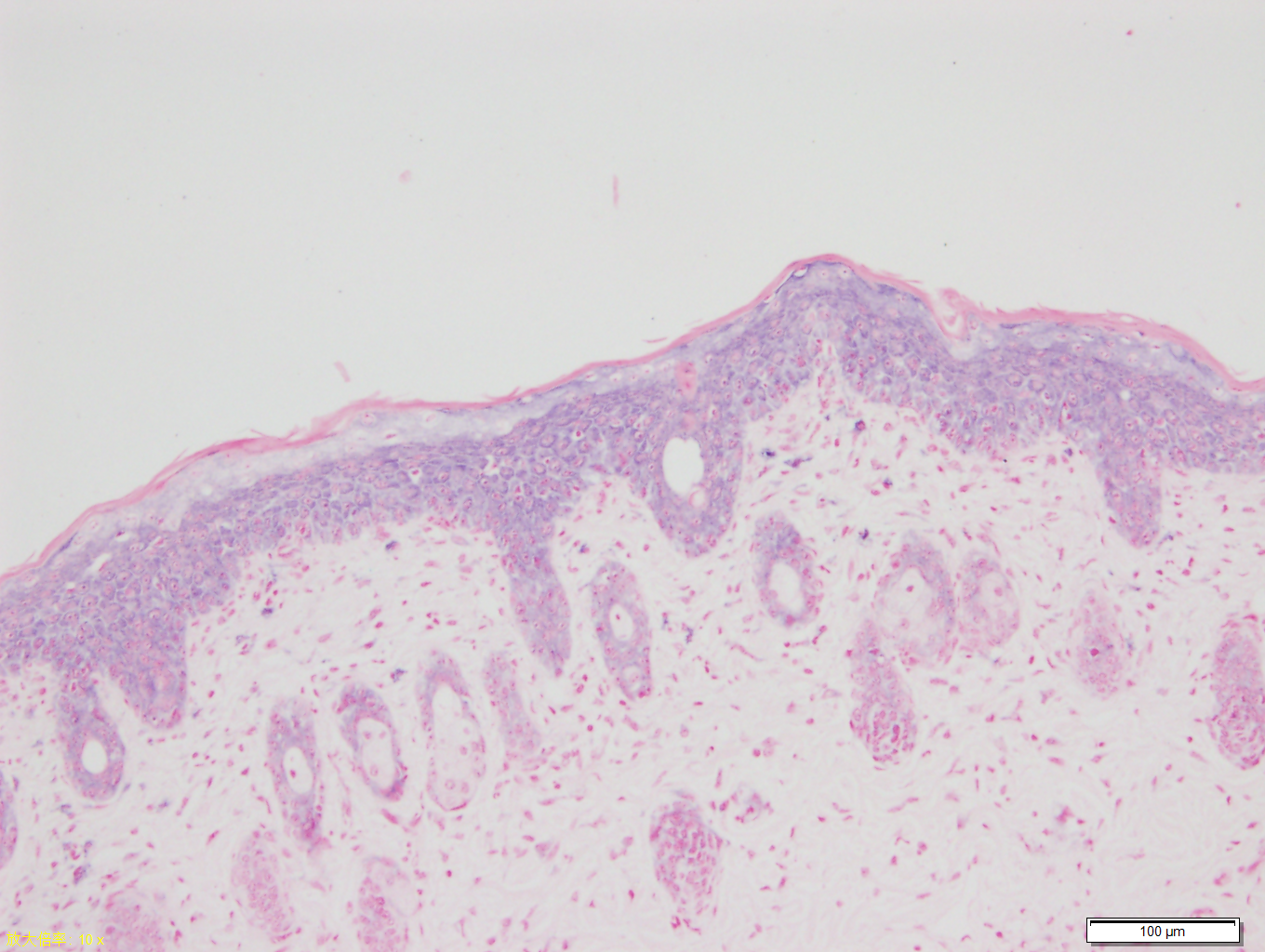

Supplement: Supplementary file 10 — Source Data for Expanded View [file EMMM-15-e15674-s014.zip › Figure Source Data File of Figure EV/Figure EV 5/5A/IMQ-5.tif]

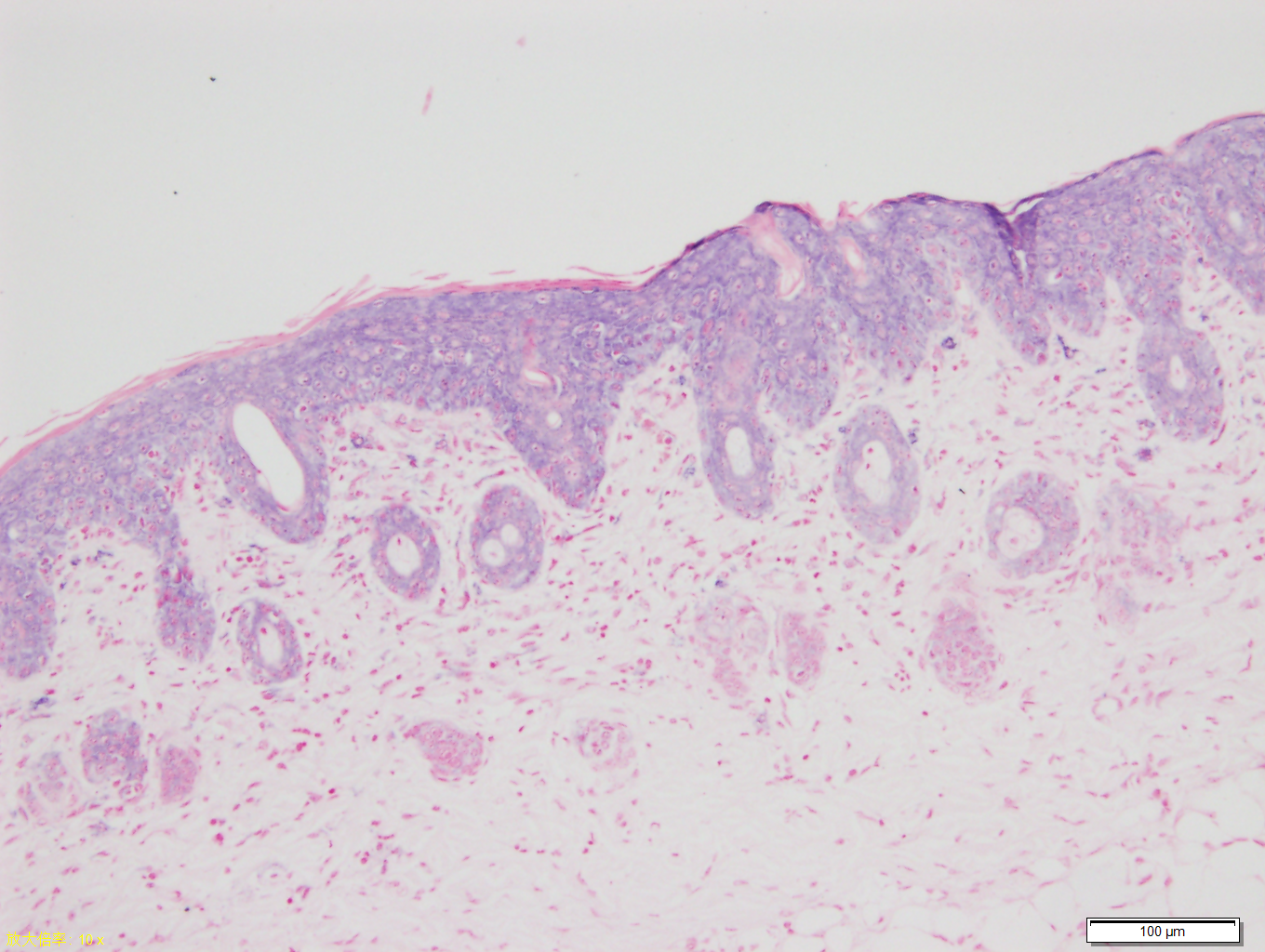

Supplement: Supplementary file 10 — Source Data for Expanded View [file EMMM-15-e15674-s014.zip › Figure Source Data File of Figure EV/Figure EV 5/5A/IMQ-4.tif]

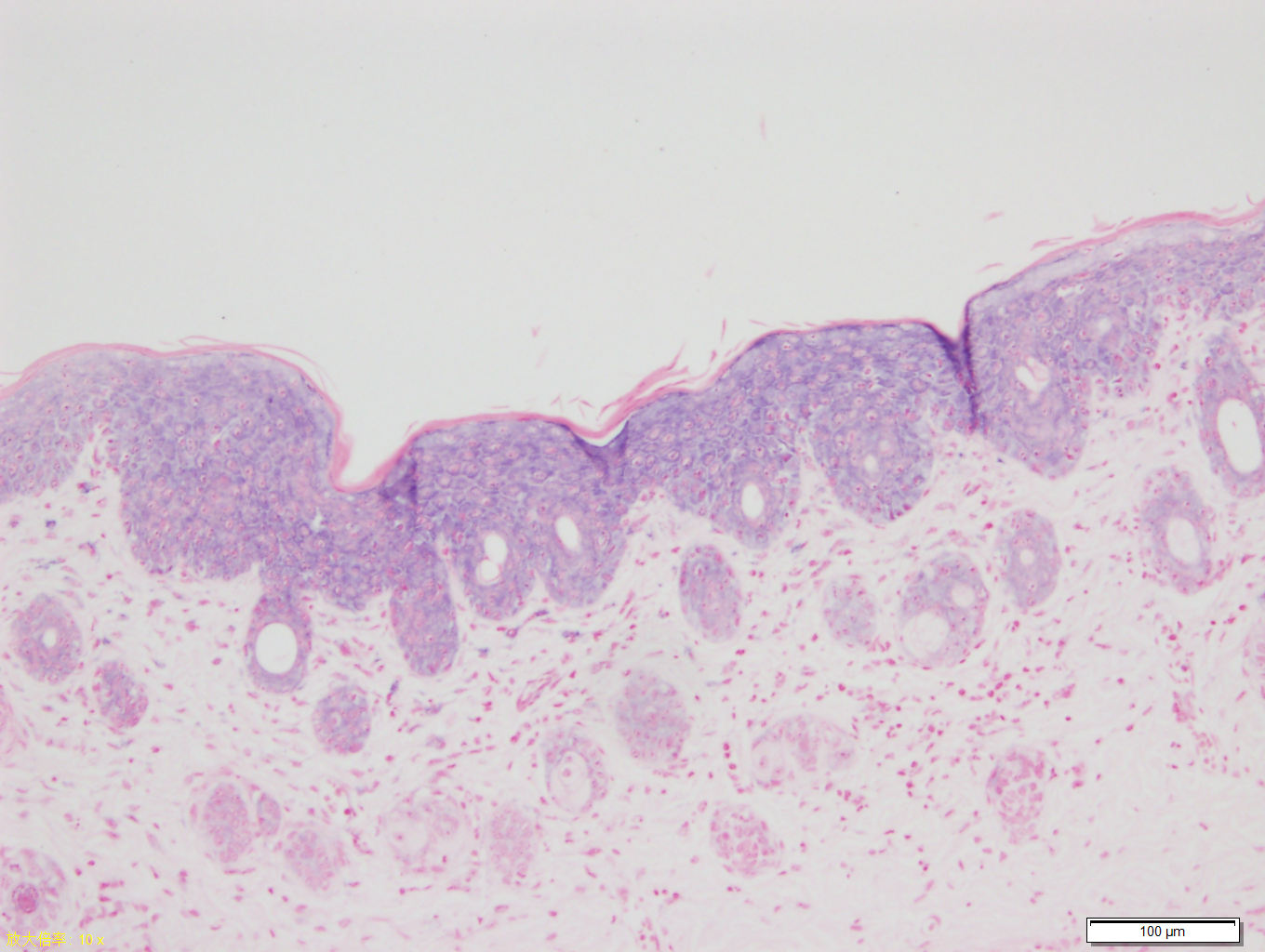

Supplement: Supplementary file 10 — Source Data for Expanded View [file EMMM-15-e15674-s014.zip › Figure Source Data File of Figure EV/Figure EV 5/5A/IMQ-6.tif]

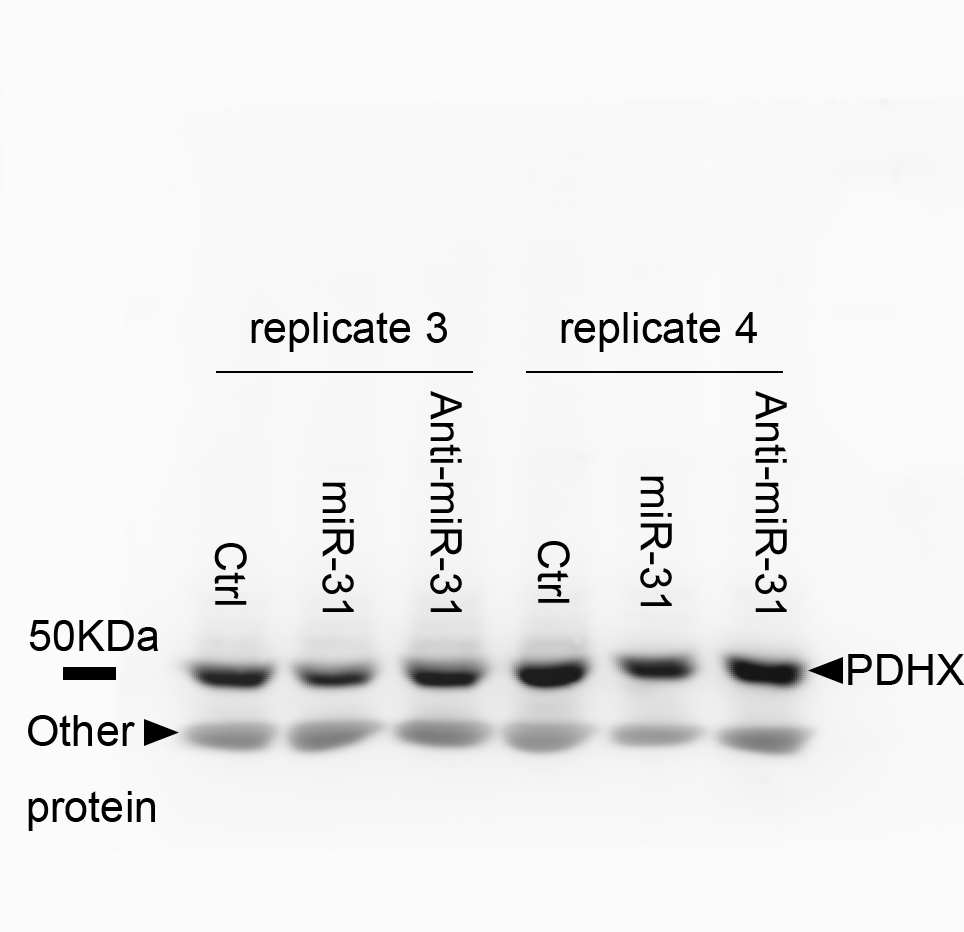

Supplement: Supplementary file 12 — Source Data for Figure 2 [file EMMM-15-e15674-s006.zip › Figure 2/2F/2F-western blot of PDHX-replicate 3 and 4.tif]

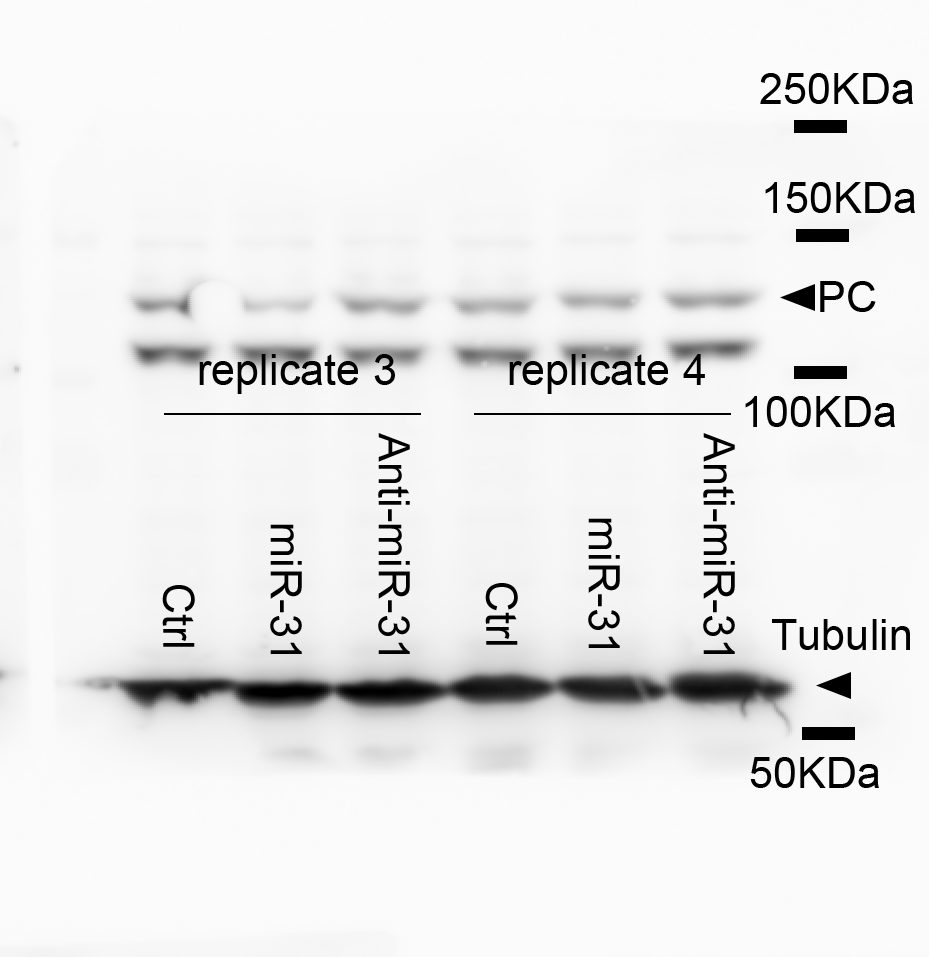

Supplement: Supplementary file 12 — Source Data for Figure 2 [file EMMM-15-e15674-s006.zip › Figure 2/2F/2F-western blot of tubulina-replicate 3 and 4.tif]

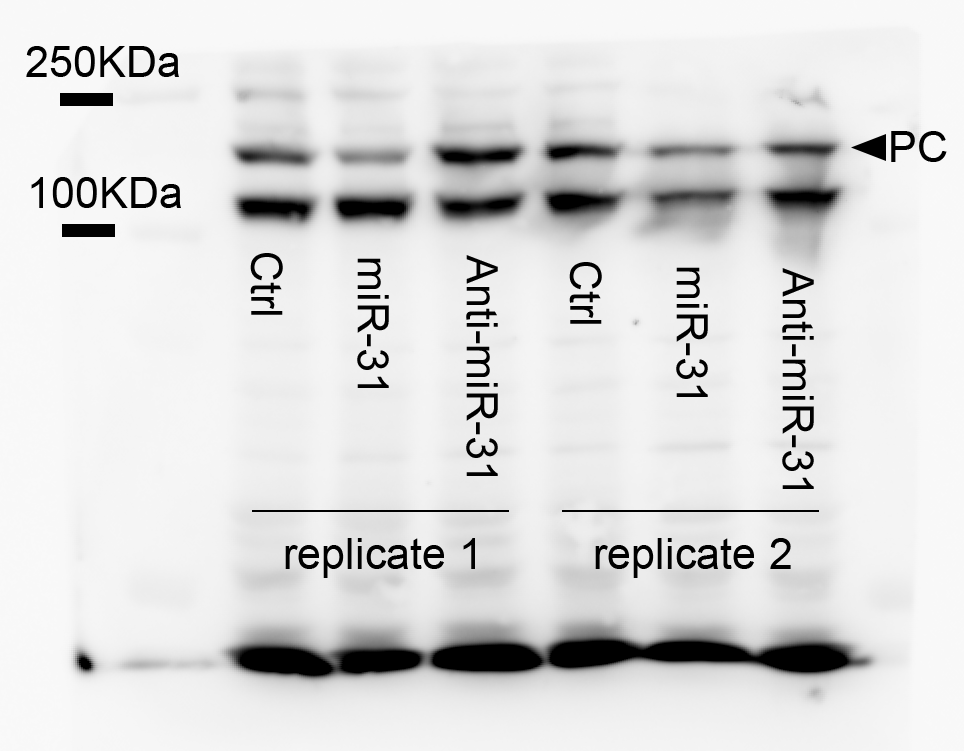

Supplement: Supplementary file 12 — Source Data for Figure 2 [file EMMM-15-e15674-s006.zip › Figure 2/2F/2F-western blot of PC-replicate 1 and 2.tif]

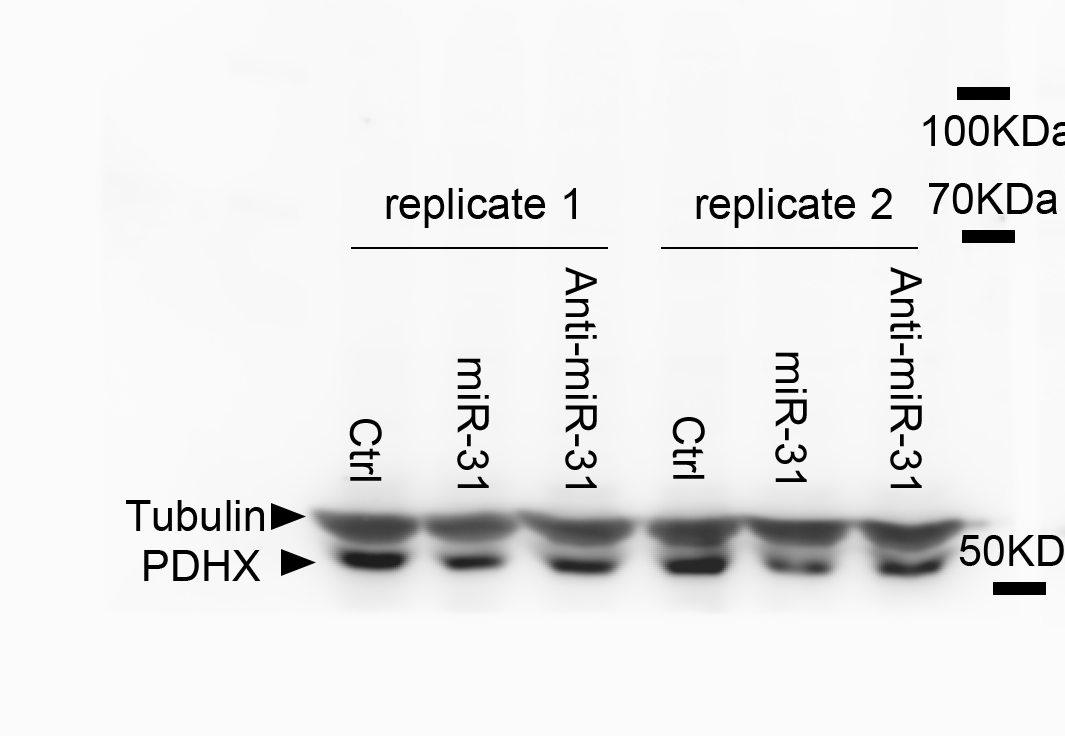

Supplement: Supplementary file 12 — Source Data for Figure 2 [file EMMM-15-e15674-s006.zip › Figure 2/2F/2F-western blot of tubulina-replicate 1 and 2.tif]

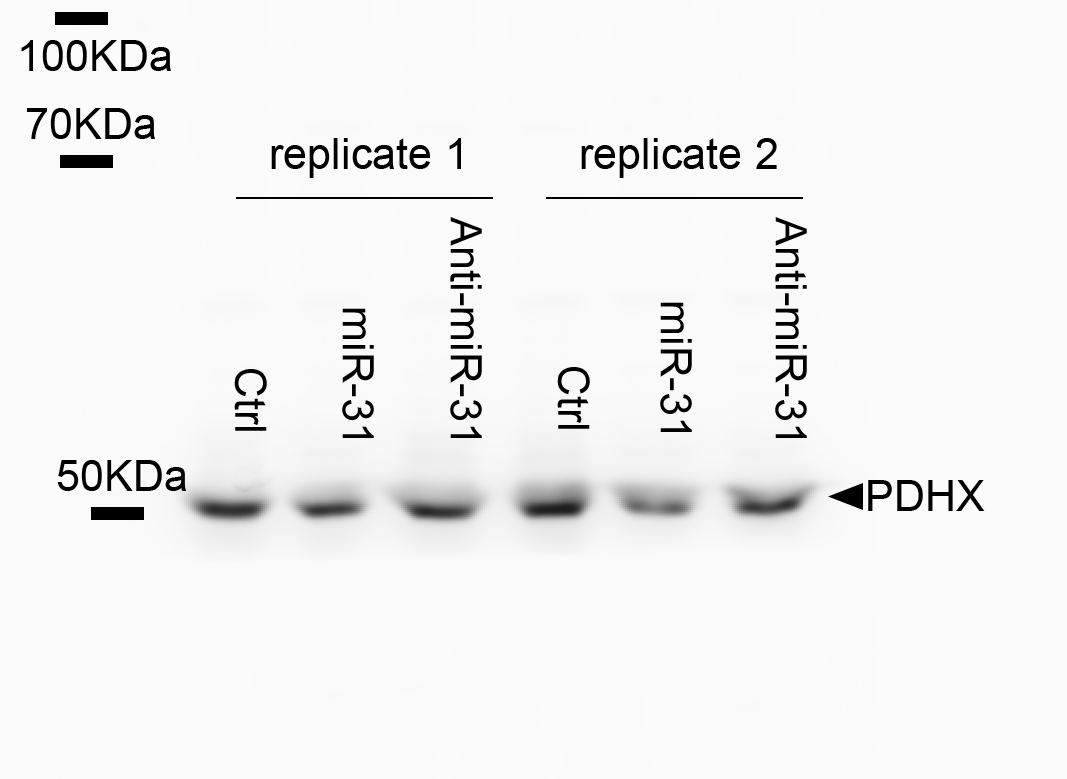

Supplement: Supplementary file 12 — Source Data for Figure 2 [file EMMM-15-e15674-s006.zip › Figure 2/2F/2F-western blot of PDHX-replicate 1 and 2.tif]

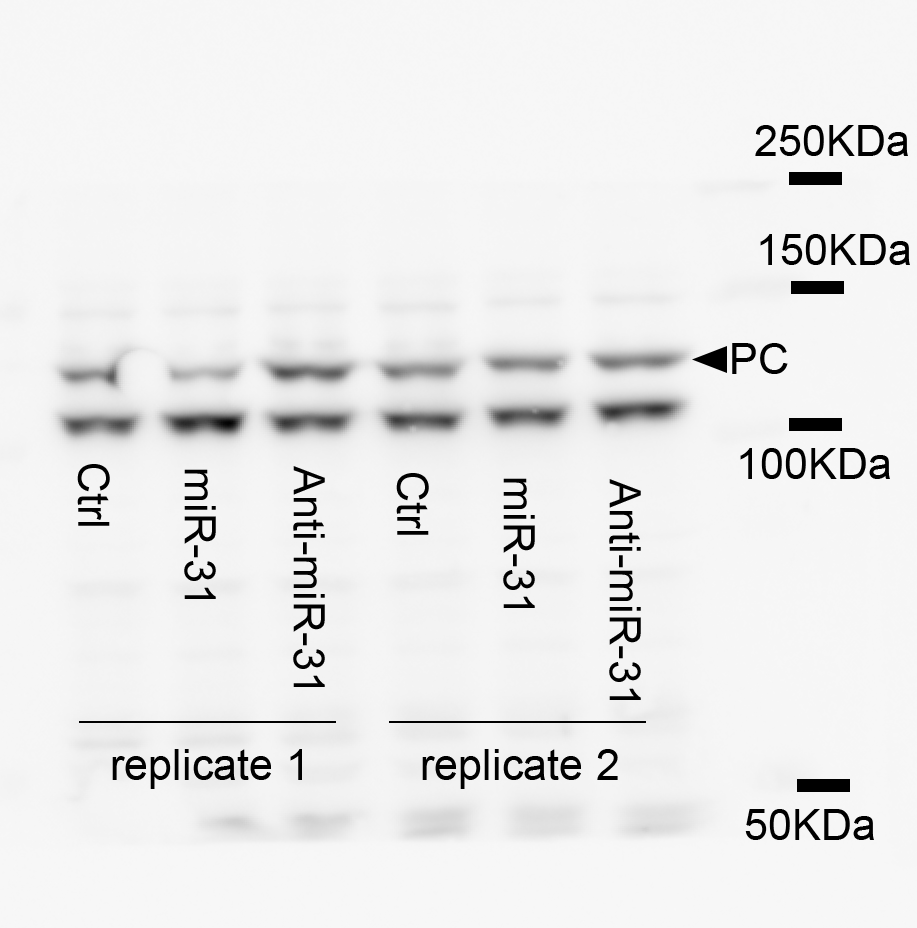

Supplement: Supplementary file 12 — Source Data for Figure 2 [file EMMM-15-e15674-s006.zip › Figure 2/2F/2F-western blot of PC-replicate 3 and 4.tif]

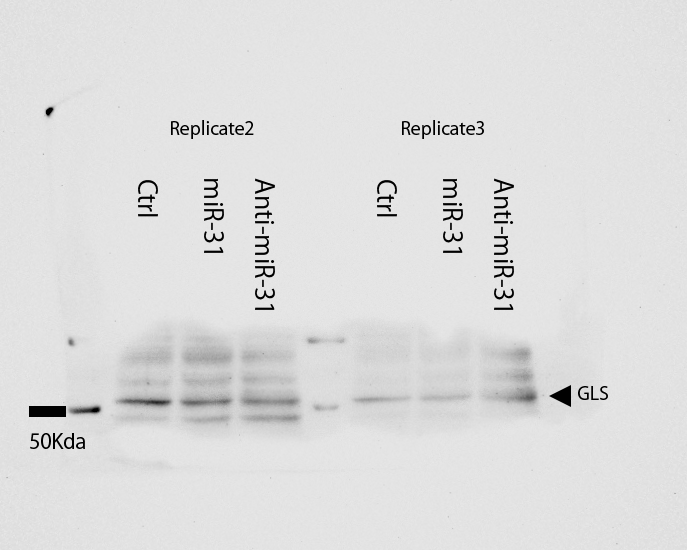

Supplement: Supplementary file 13 — Source Data for Figure 3 [file EMMM-15-e15674-s008.zip › Figure 3/3B/3B-western blot of GLS - replicate 2 and 3.tif]

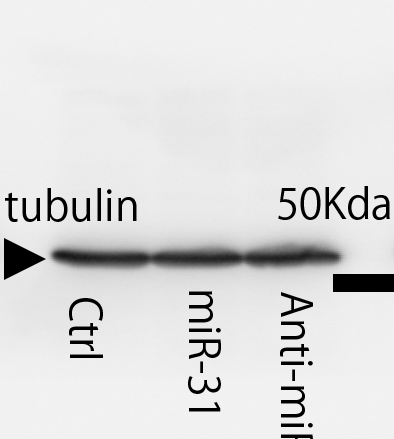

Supplement: Supplementary file 13 — Source Data for Figure 3 [file EMMM-15-e15674-s008.zip › Figure 3/3B/3B-western blot of tubulin-a - replicate 3.tif]

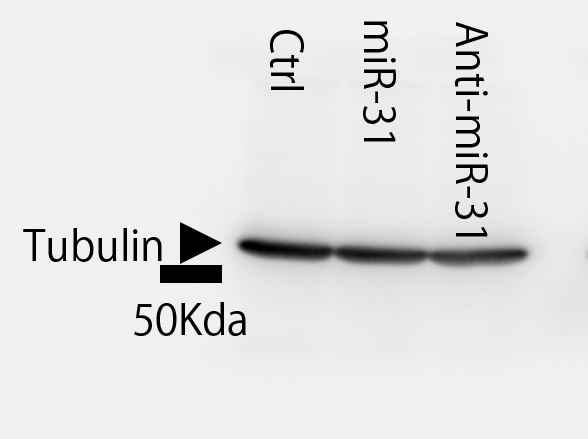

Supplement: Supplementary file 13 — Source Data for Figure 3 [file EMMM-15-e15674-s008.zip › Figure 3/3B/3B-western blot of tubulin-a - replicate 2.tif]

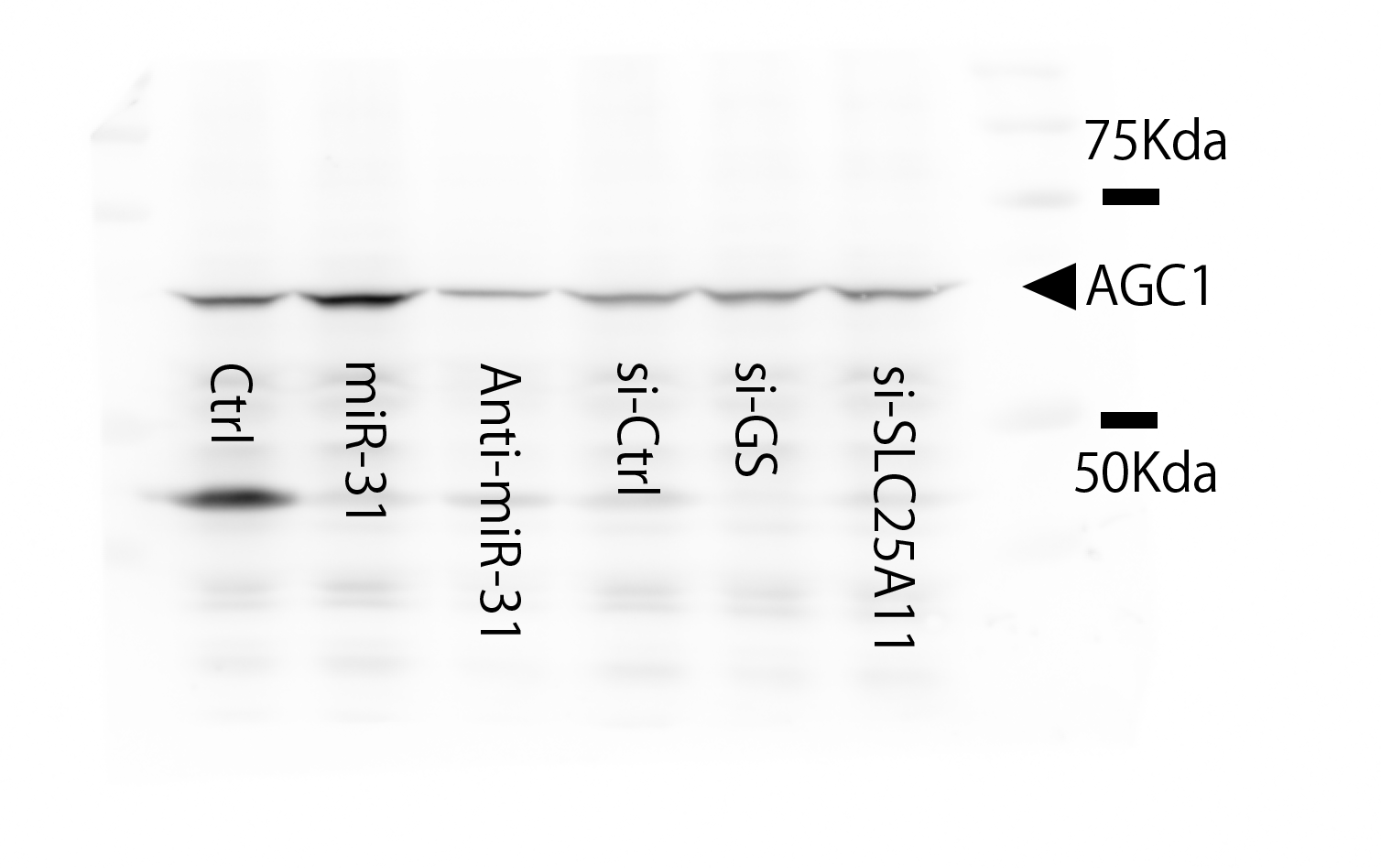

Supplement: Supplementary file 13 — Source Data for Figure 3 [file EMMM-15-e15674-s008.zip › Figure 3/3B/3B-western blot of AGC1 - replicate 3.tif]

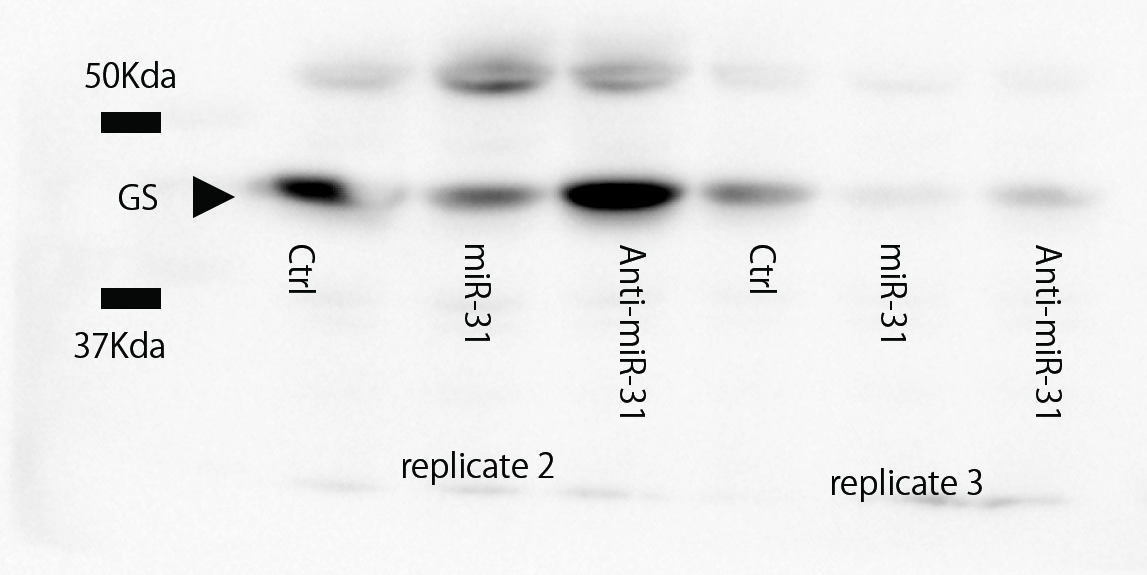

Supplement: Supplementary file 13 — Source Data for Figure 3 [file EMMM-15-e15674-s008.zip › Figure 3/3B/3B-western blot of GS - replicate 2 and 3.tif]

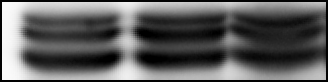

Supplement: Supplementary file 13 — Source Data for Figure 3 [file EMMM-15-e15674-s008.zip › Figure 3/3B/3B-western blot of GLS - replicate 1.tif]

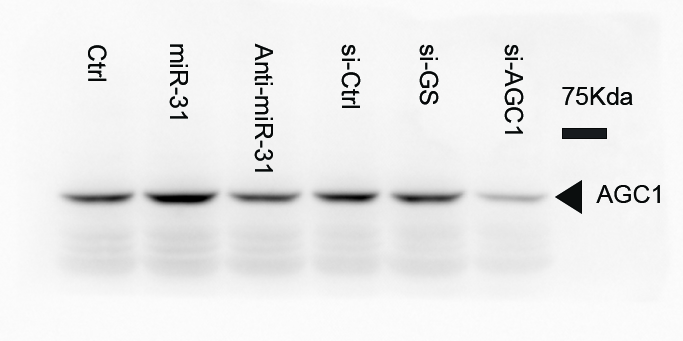

Supplement: Supplementary file 13 — Source Data for Figure 3 [file EMMM-15-e15674-s008.zip › Figure 3/3B/3B-western blot of AGC1 - replicate 1.tif]

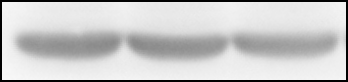

Supplement: Supplementary file 13 — Source Data for Figure 3 [file EMMM-15-e15674-s008.zip › Figure 3/3B/3B-western blot of tubulin-a - replicate 1.tif]

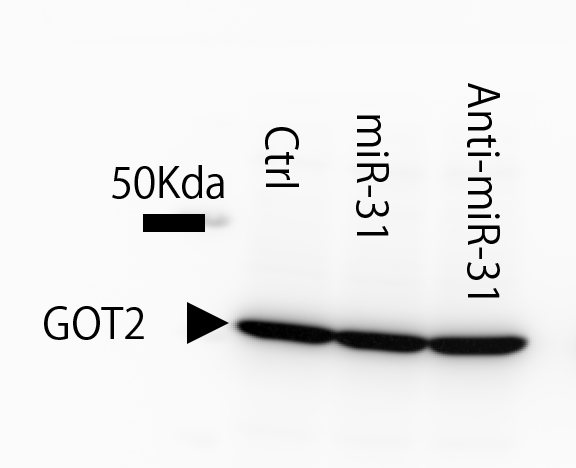

Supplement: Supplementary file 13 — Source Data for Figure 3 [file EMMM-15-e15674-s008.zip › Figure 3/3B/3B-western blot of GOT2 - replicate 3.tif]

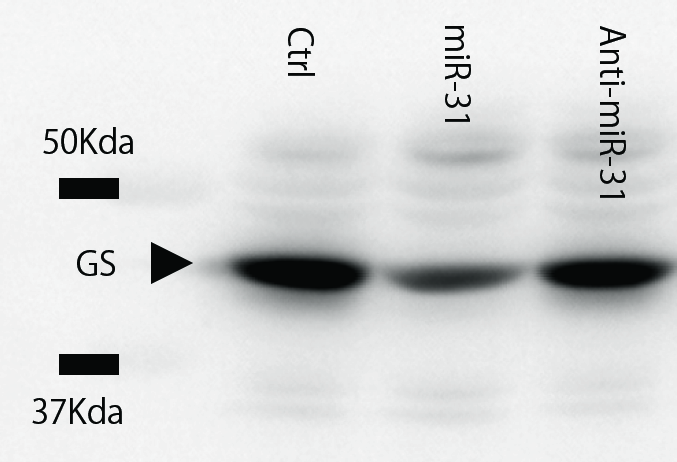

Supplement: Supplementary file 13 — Source Data for Figure 3 [file EMMM-15-e15674-s008.zip › Figure 3/3B/3B-western blot of GS - replicate 1.tif]

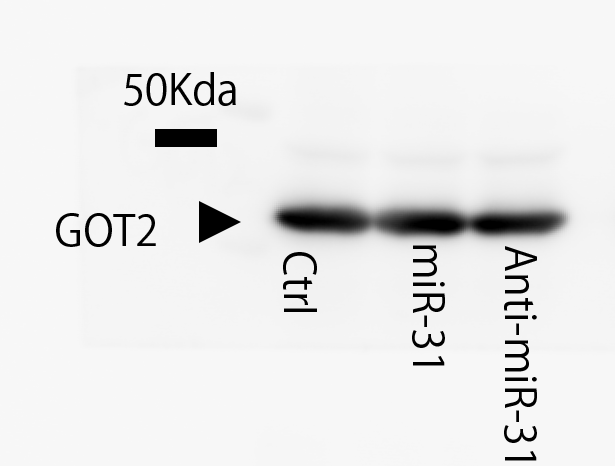

Supplement: Supplementary file 13 — Source Data for Figure 3 [file EMMM-15-e15674-s008.zip › Figure 3/3B/3B-western blot of GOT2 - replicate 2.tif]

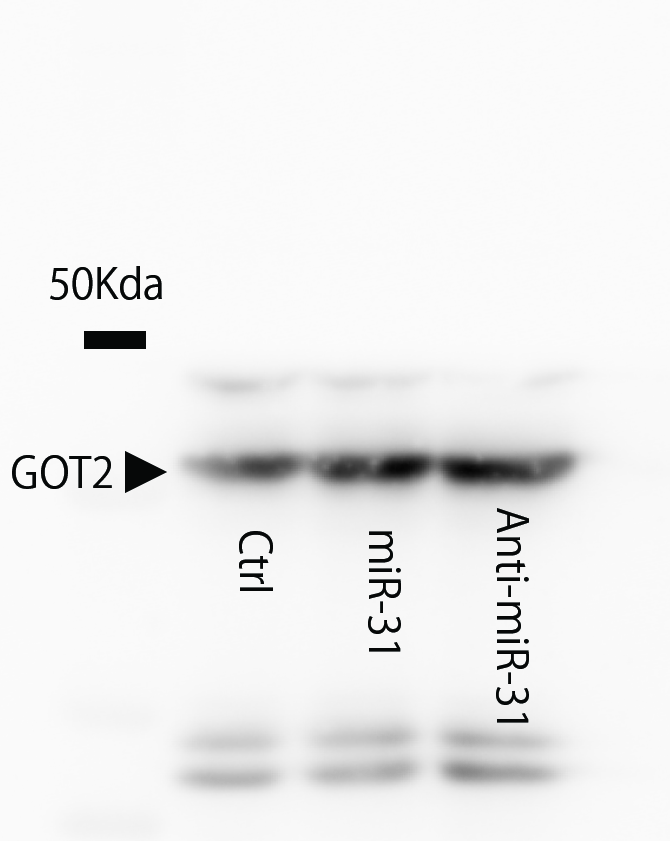

Supplement: Supplementary file 13 — Source Data for Figure 3 [file EMMM-15-e15674-s008.zip › Figure 3/3B/3B-western blot of GOT2 - replicate 1.tif]

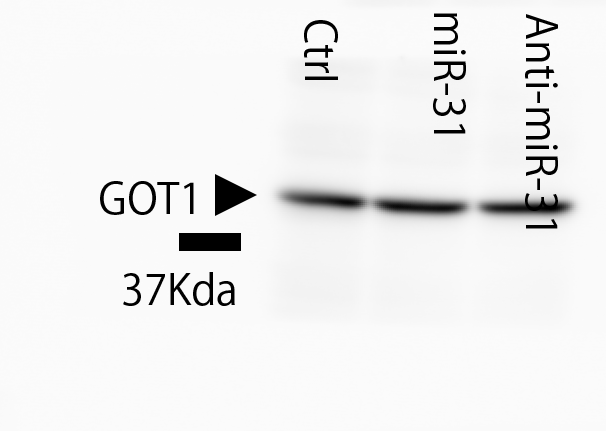

Supplement: Supplementary file 13 — Source Data for Figure 3 [file EMMM-15-e15674-s008.zip › Figure 3/3B/3B-western blot of GOT1 - replicate 3.tif]

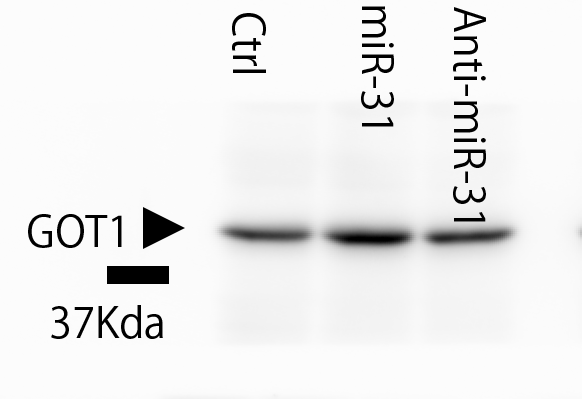

Supplement: Supplementary file 13 — Source Data for Figure 3 [file EMMM-15-e15674-s008.zip › Figure 3/3B/3B-western blot of GOT1 - replicate 2.tif]

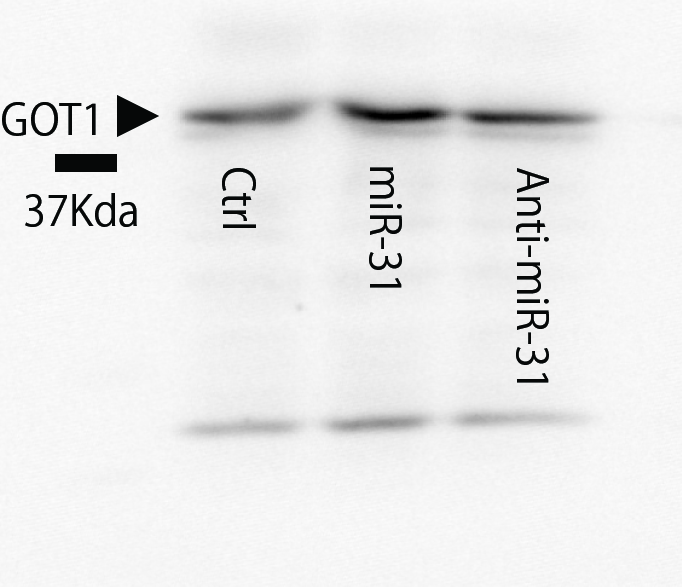

Supplement: Supplementary file 13 — Source Data for Figure 3 [file EMMM-15-e15674-s008.zip › Figure 3/3B/3B-western blot of GOT1 - replicate 1.tif]

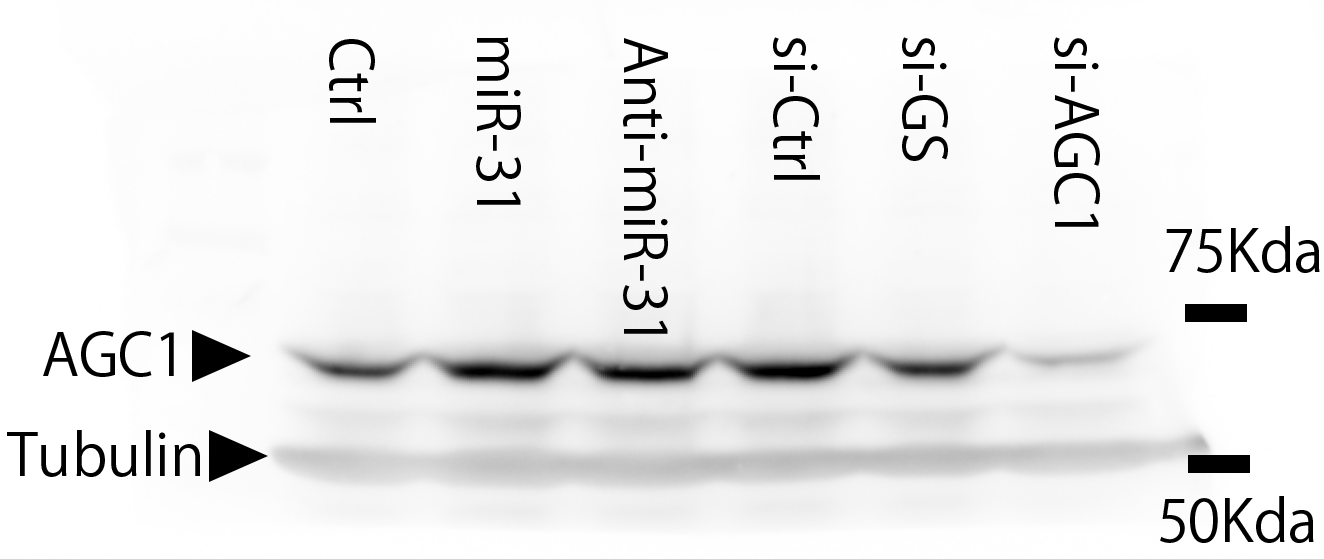

Supplement: Supplementary file 13 — Source Data for Figure 3 [file EMMM-15-e15674-s008.zip › Figure 3/3B/3B-western blot of AGC1 and tubulin- replicate 2.tif]

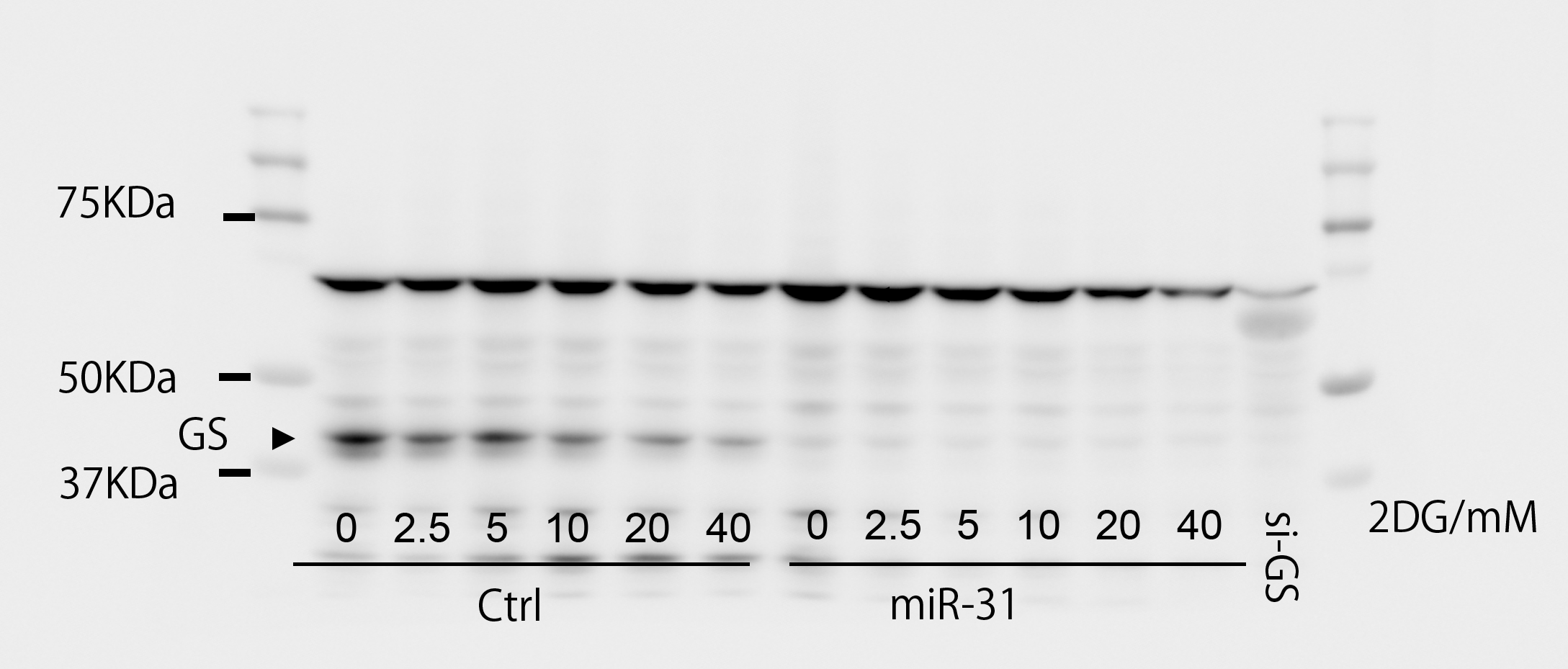

Supplement: Supplementary file 14 — Source Data for Figure 4 [file EMMM-15-e15674-s009.zip › Figure 4/4D/4D-western blot GS-2DG-replicate 1.tif]

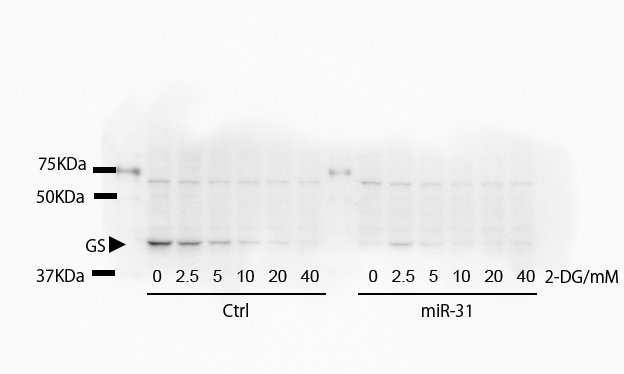

Supplement: Supplementary file 14 — Source Data for Figure 4 [file EMMM-15-e15674-s009.zip › Figure 4/4D/4D-western blot GS-2DG-replicate 3.tif]

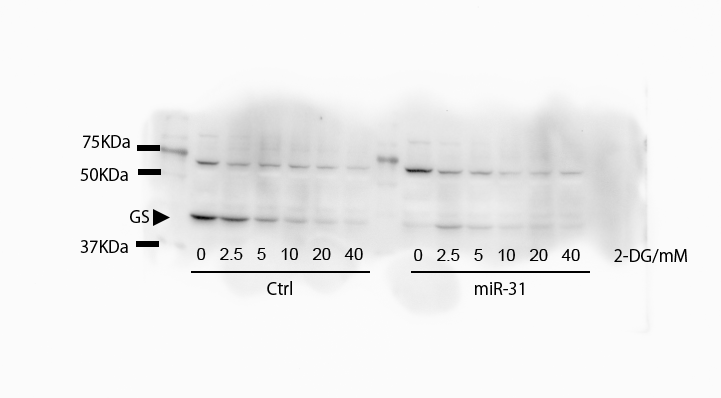

Supplement: Supplementary file 14 — Source Data for Figure 4 [file EMMM-15-e15674-s009.zip › Figure 4/4D/4D-western blot GS-2DG-replicate 2.tif]

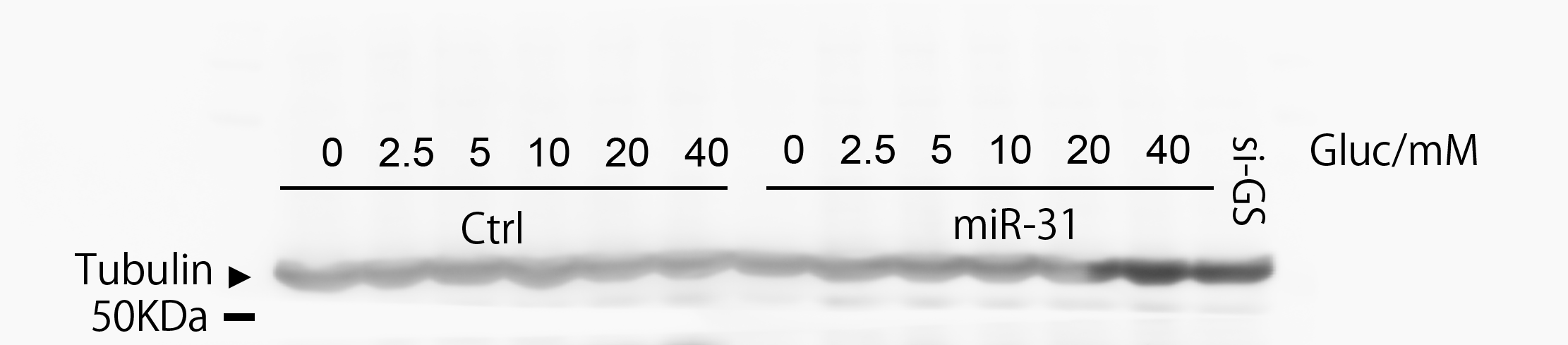

Supplement: Supplementary file 14 — Source Data for Figure 4 [file EMMM-15-e15674-s009.zip › Figure 4/4D/4D-western blot tubulin-glucose-replicate 1.tif]

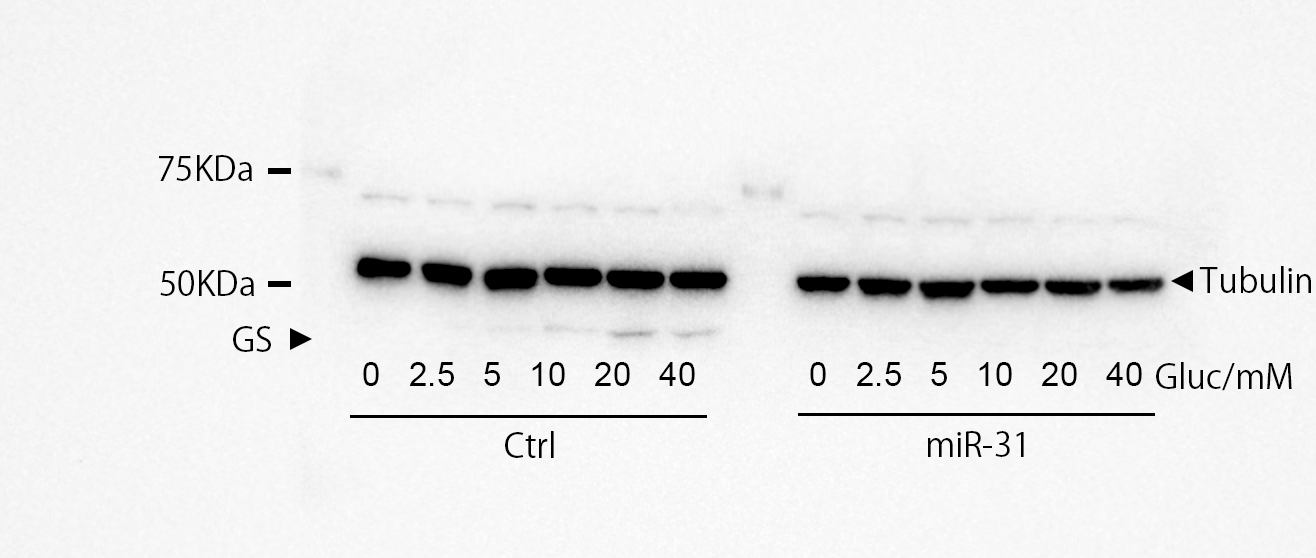

Supplement: Supplementary file 14 — Source Data for Figure 4 [file EMMM-15-e15674-s009.zip › Figure 4/4D/4D-western blot tubulin-glucose-replicate 3.tif]

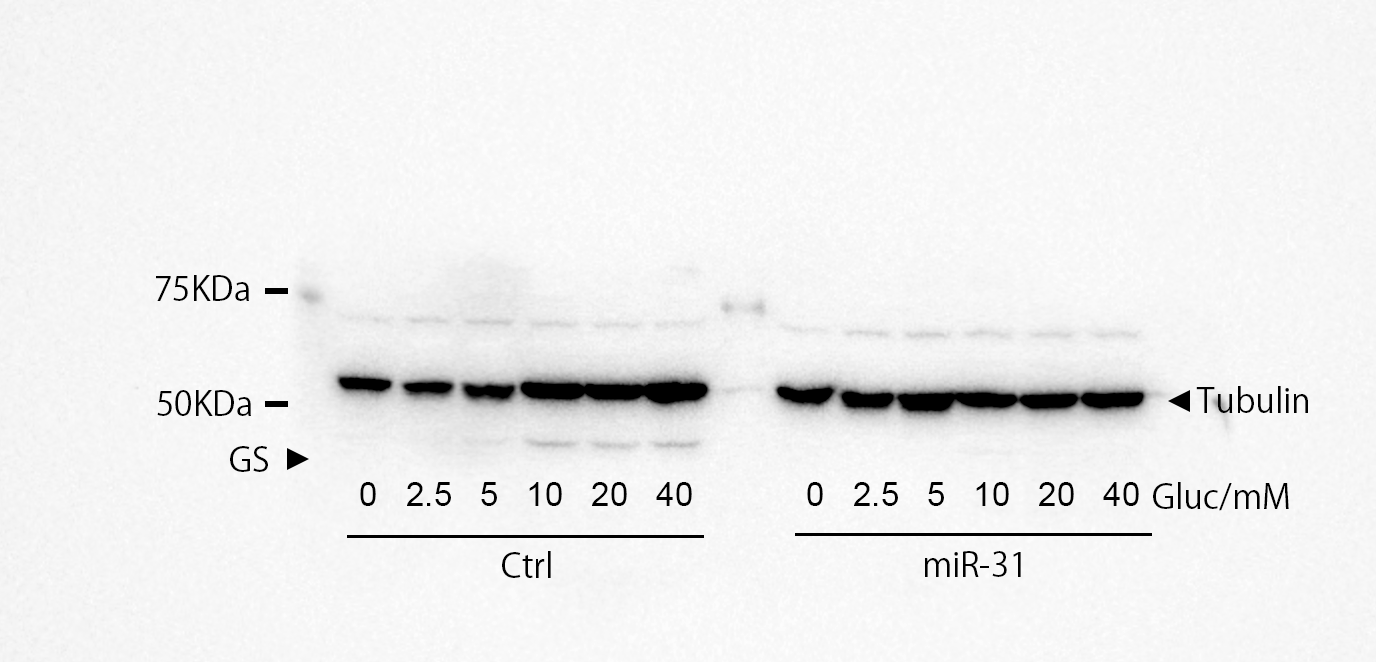

Supplement: Supplementary file 14 — Source Data for Figure 4 [file EMMM-15-e15674-s009.zip › Figure 4/4D/4D-western blot tubulin-glucose-replicate 2.tif]

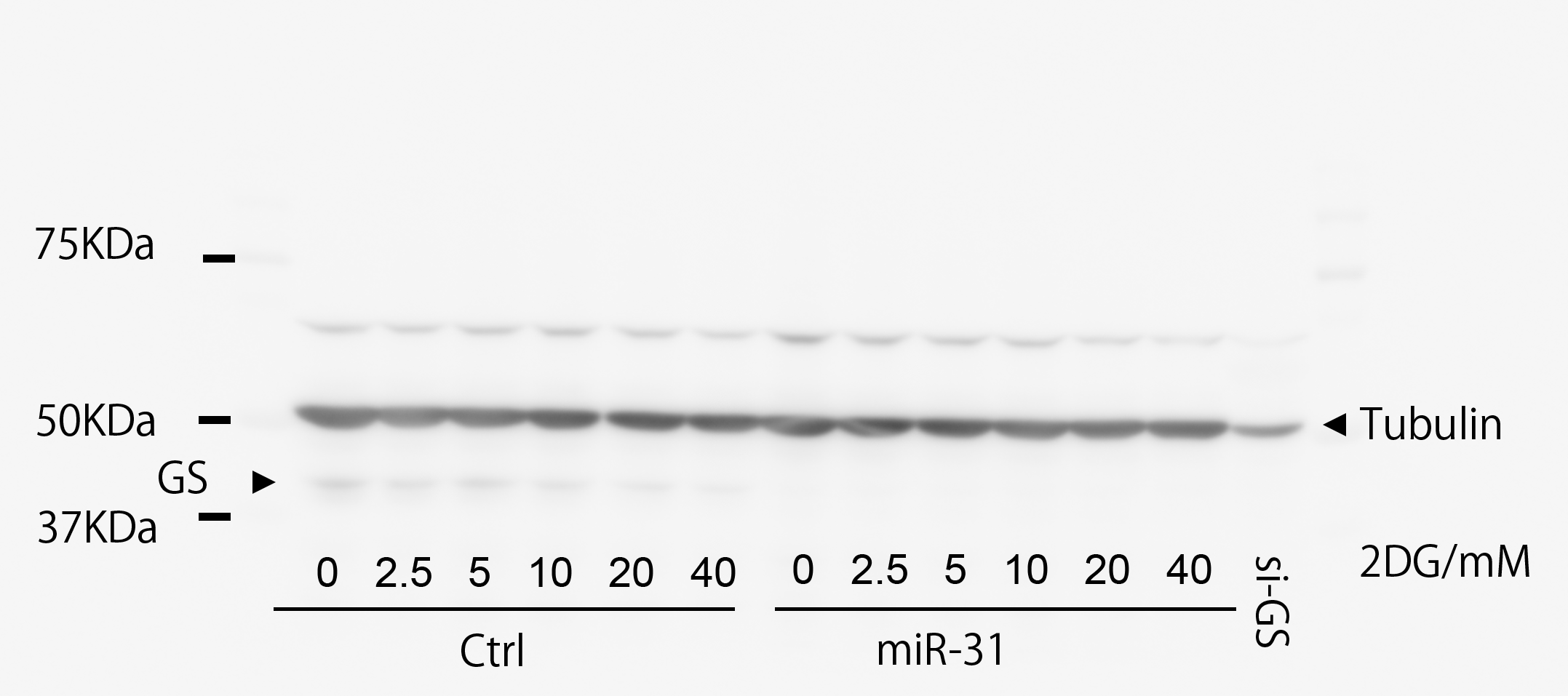

Supplement: Supplementary file 14 — Source Data for Figure 4 [file EMMM-15-e15674-s009.zip › Figure 4/4D/4D-western blot tubulin-2DG-replicate 1.tif]

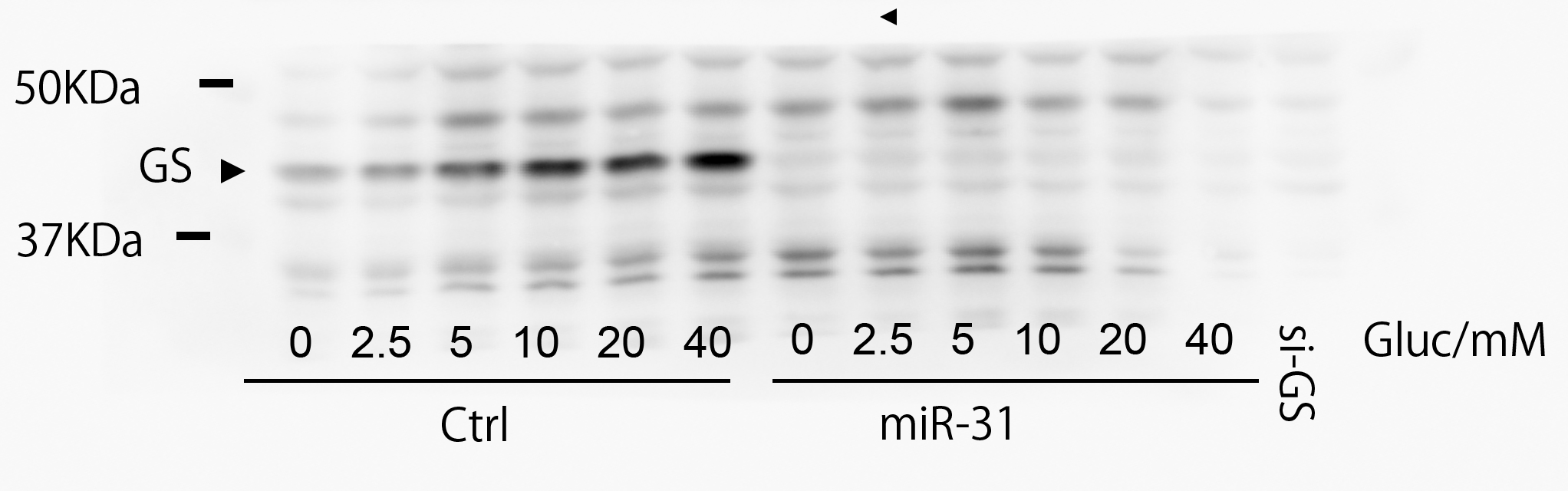

Supplement: Supplementary file 14 — Source Data for Figure 4 [file EMMM-15-e15674-s009.zip › Figure 4/4D/4D-western blot GS-glucose-replicate 1.tif]

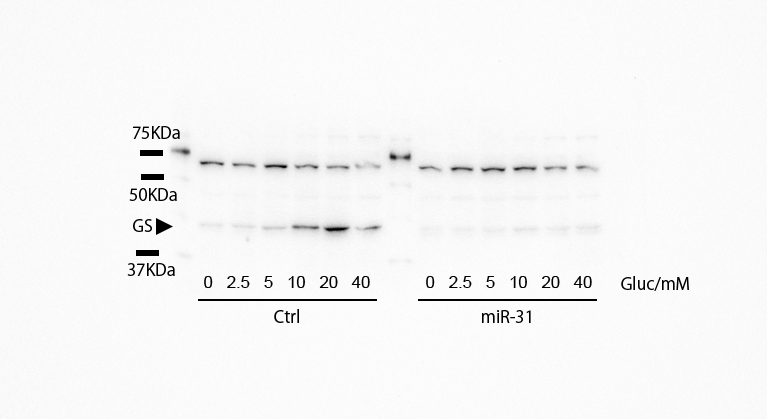

Supplement: Supplementary file 14 — Source Data for Figure 4 [file EMMM-15-e15674-s009.zip › Figure 4/4D/4D-western blot GS-glucose-replicate 3.tif]

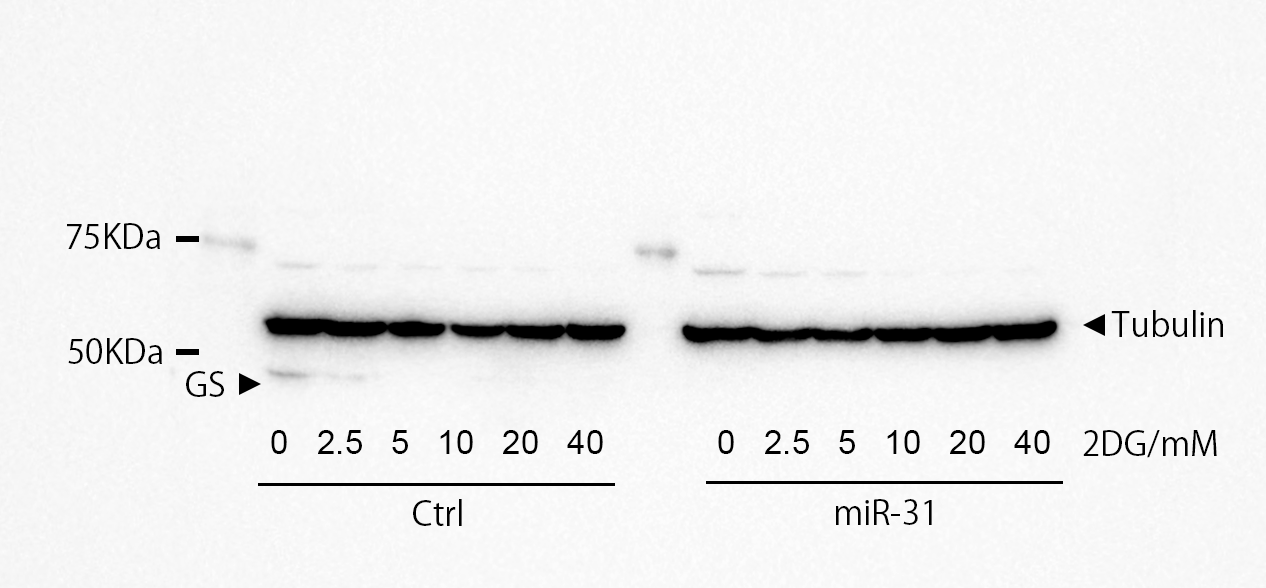

Supplement: Supplementary file 14 — Source Data for Figure 4 [file EMMM-15-e15674-s009.zip › Figure 4/4D/4D-western blot Tubulin-2DG-replicate 2.tif]

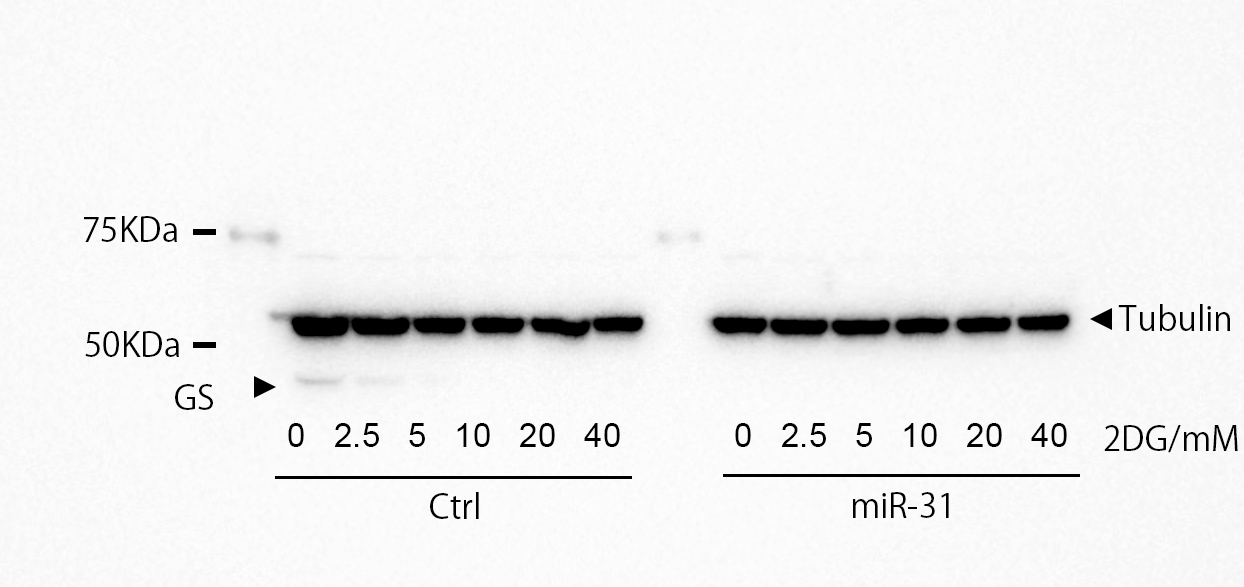

Supplement: Supplementary file 14 — Source Data for Figure 4 [file EMMM-15-e15674-s009.zip › Figure 4/4D/4D-western blot tubulin-2DG-replicate 3.tif]

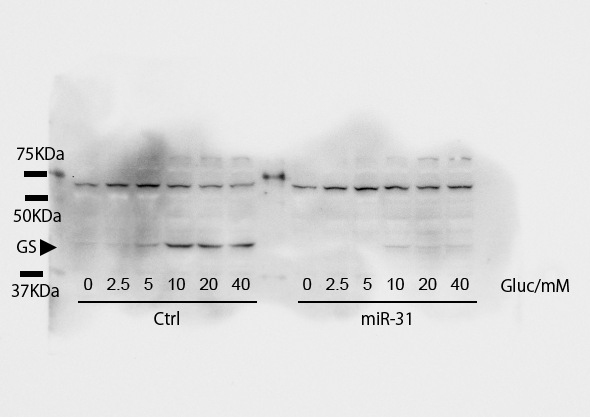

Supplement: Supplementary file 14 — Source Data for Figure 4 [file EMMM-15-e15674-s009.zip › Figure 4/4D/4D-western blot GS-glucose-replicate 2.tif]

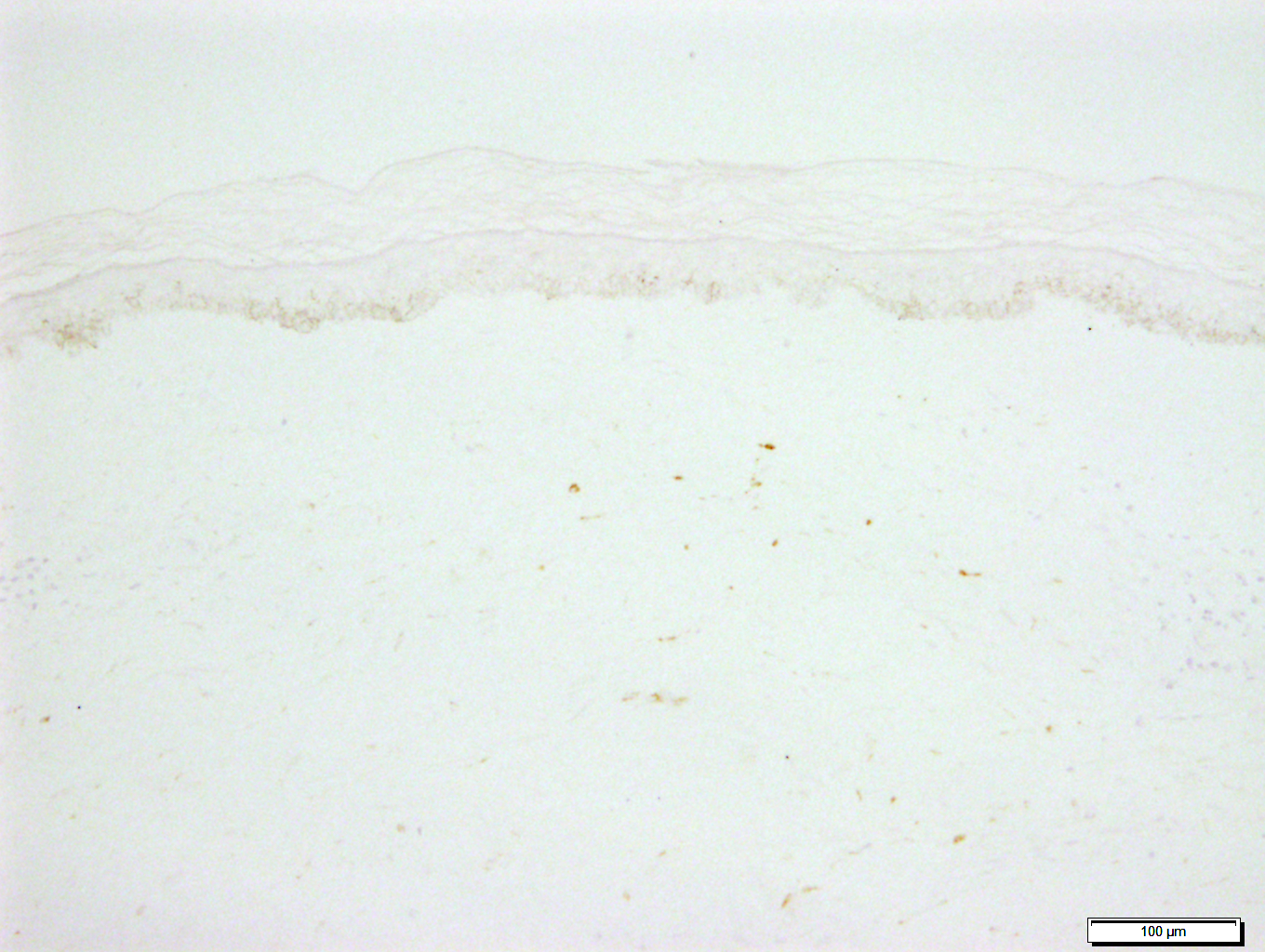

Supplement: Supplementary file 15 — Source Data for Figure 5 [file EMMM-15-e15674-s002.zip › Figure 5/5A/HC-5.tif]

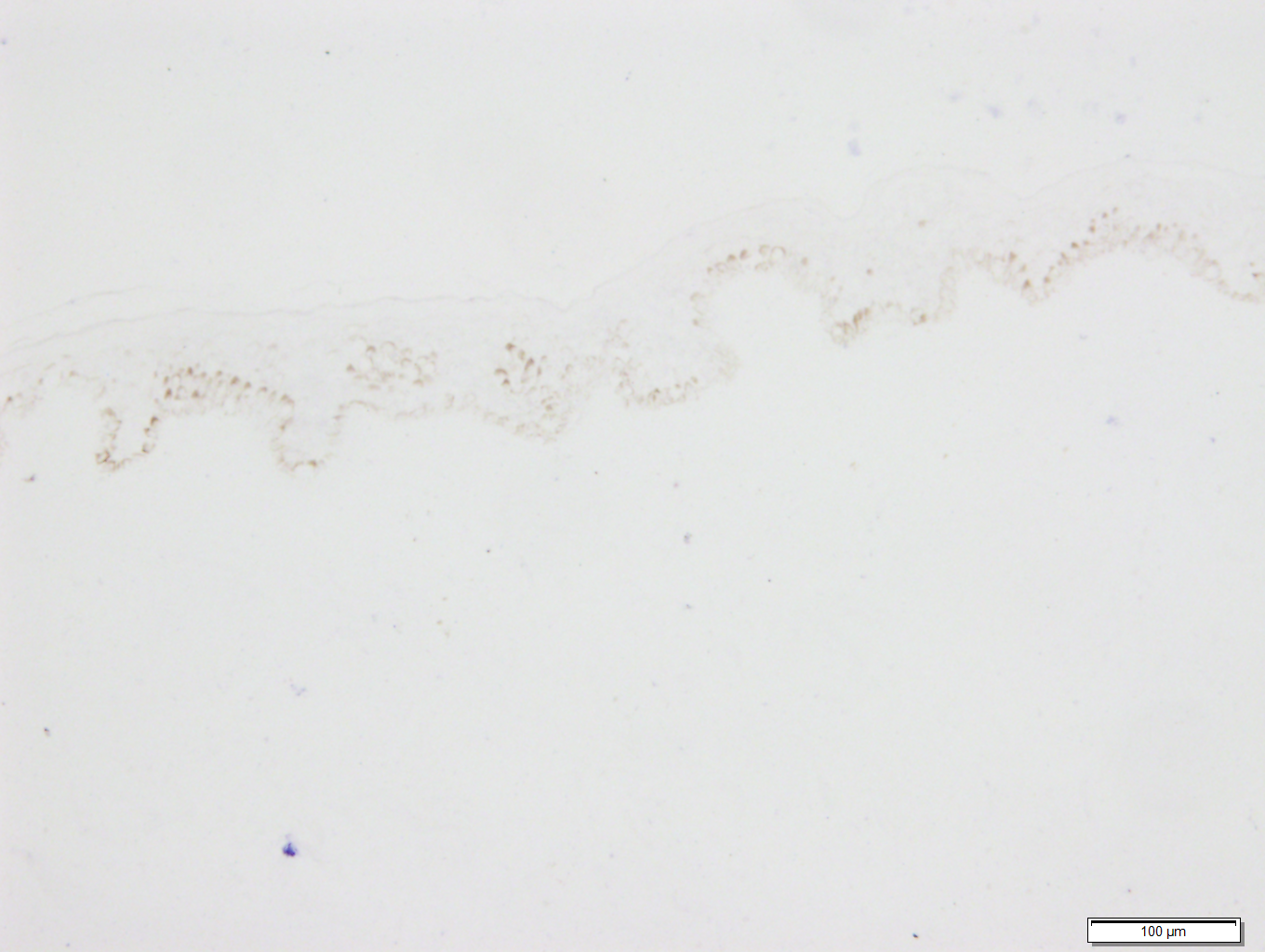

Supplement: Supplementary file 15 — Source Data for Figure 5 [file EMMM-15-e15674-s002.zip › Figure 5/5A/HC-4.tif]

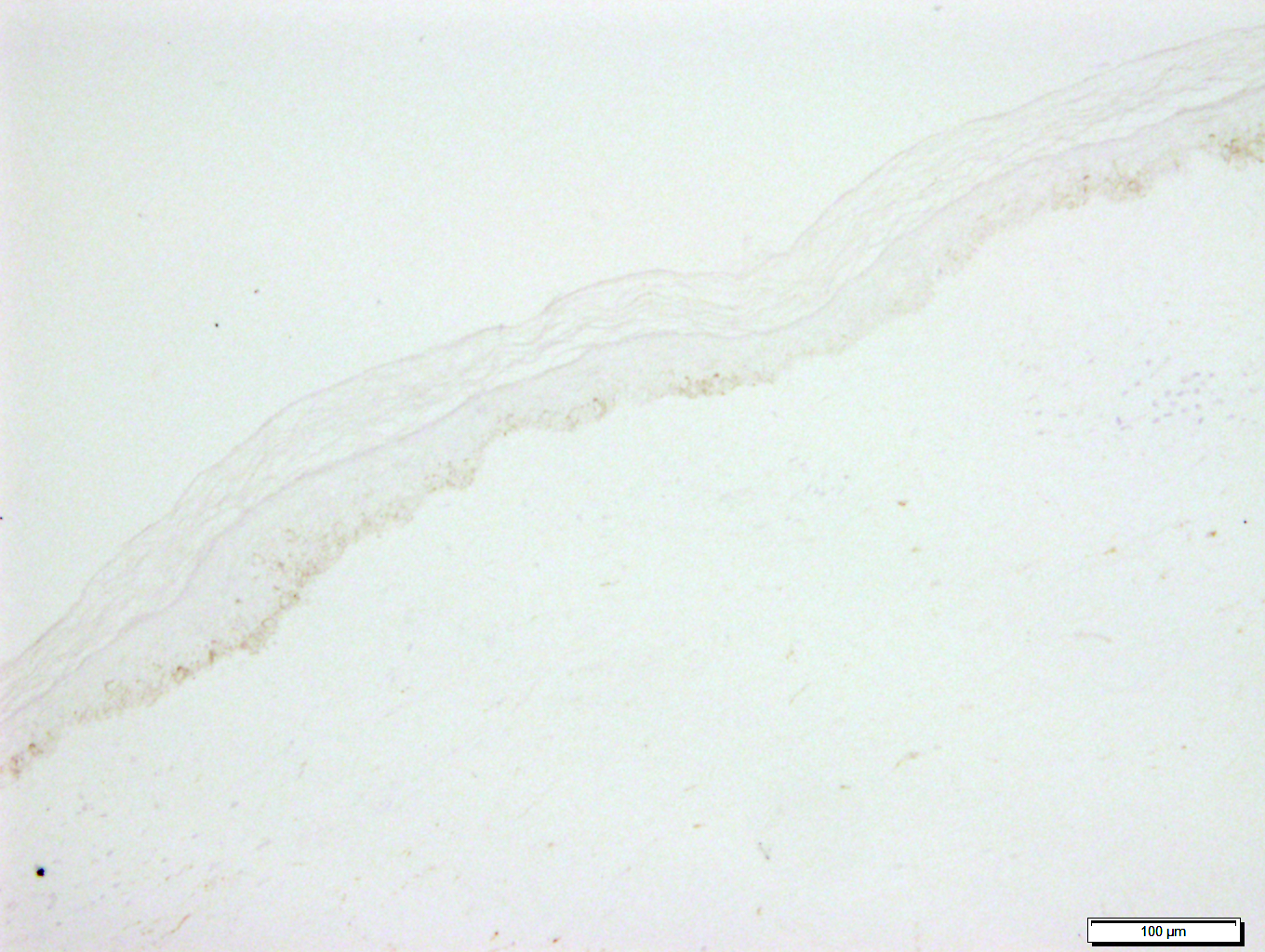

Supplement: Supplementary file 15 — Source Data for Figure 5 [file EMMM-15-e15674-s002.zip › Figure 5/5A/HC-3.tif]

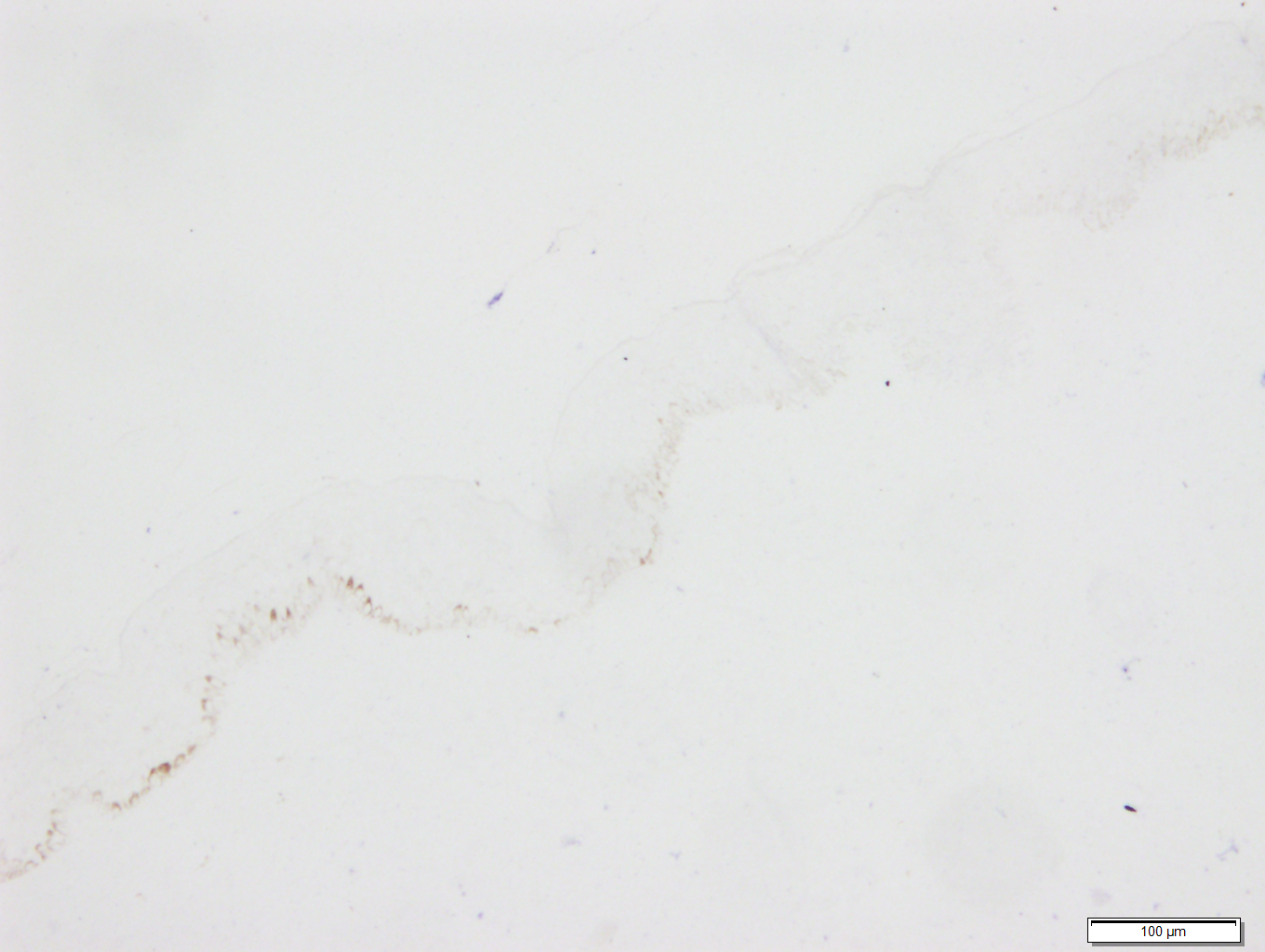

Supplement: Supplementary file 15 — Source Data for Figure 5 [file EMMM-15-e15674-s002.zip › Figure 5/5A/HC-2.tif]

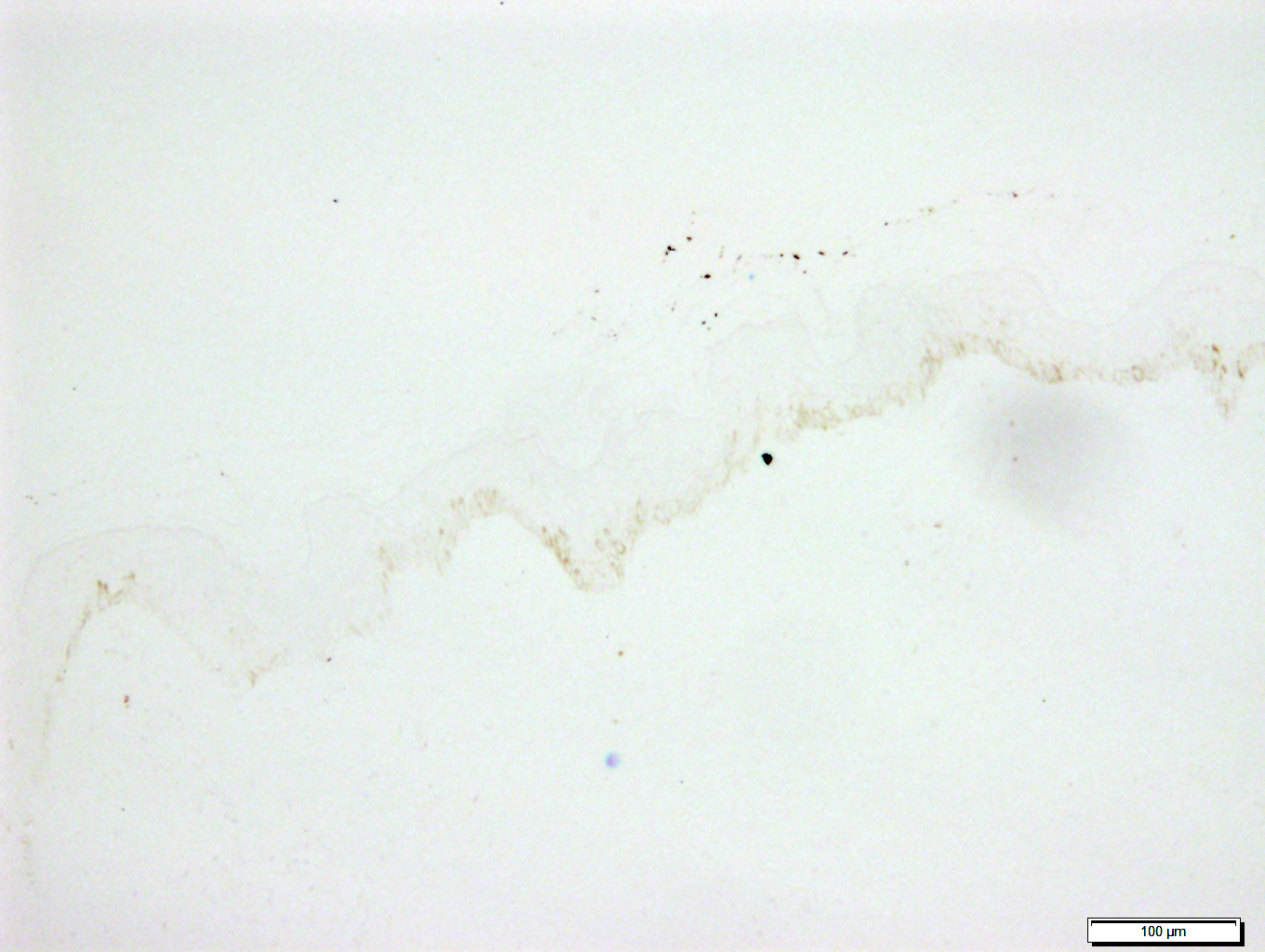

Supplement: Supplementary file 15 — Source Data for Figure 5 [file EMMM-15-e15674-s002.zip › Figure 5/5A/HC-1.tif]

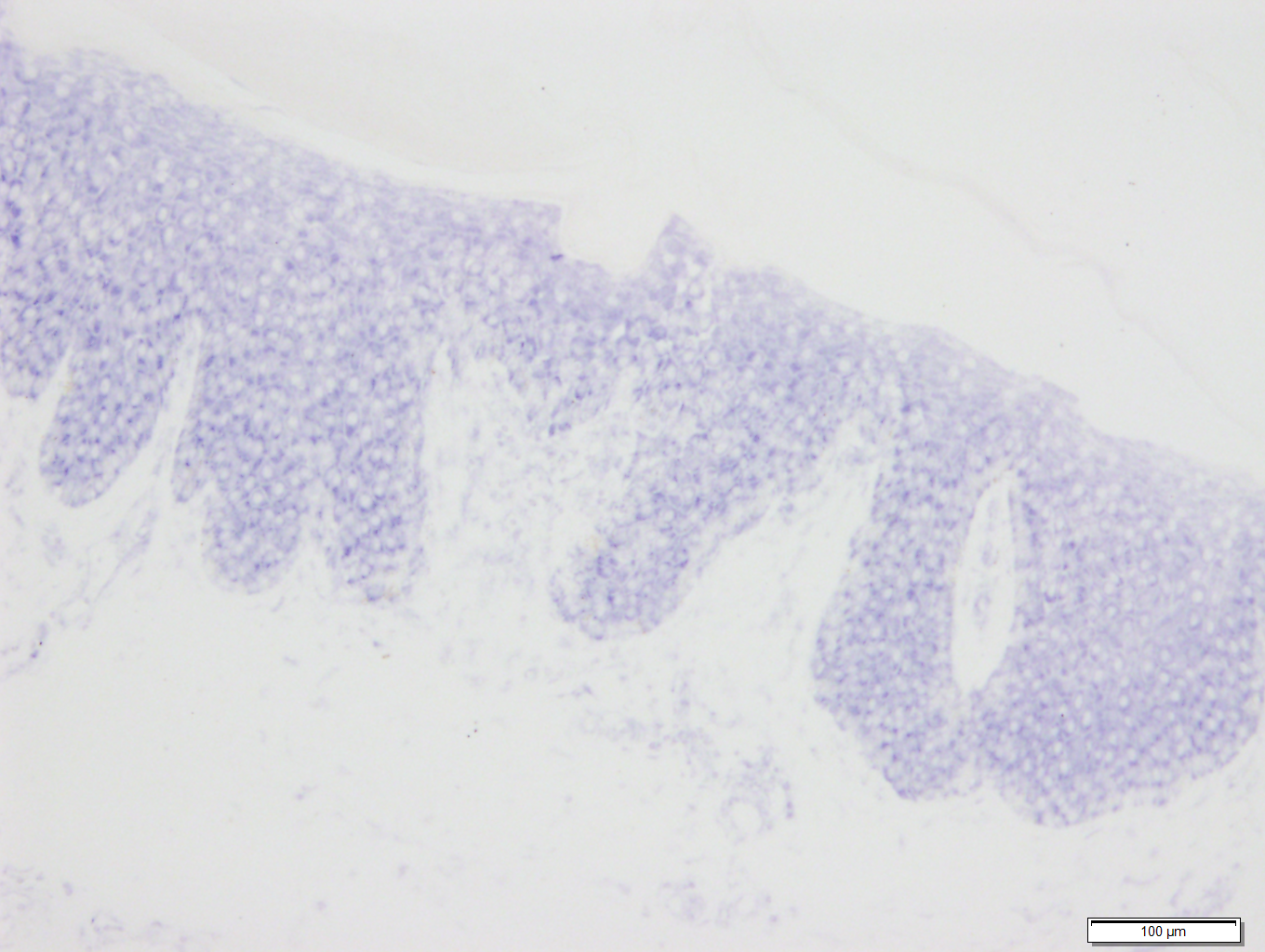

Supplement: Supplementary file 15 — Source Data for Figure 5 [file EMMM-15-e15674-s002.zip › Figure 5/5A/Ps-5.tif]

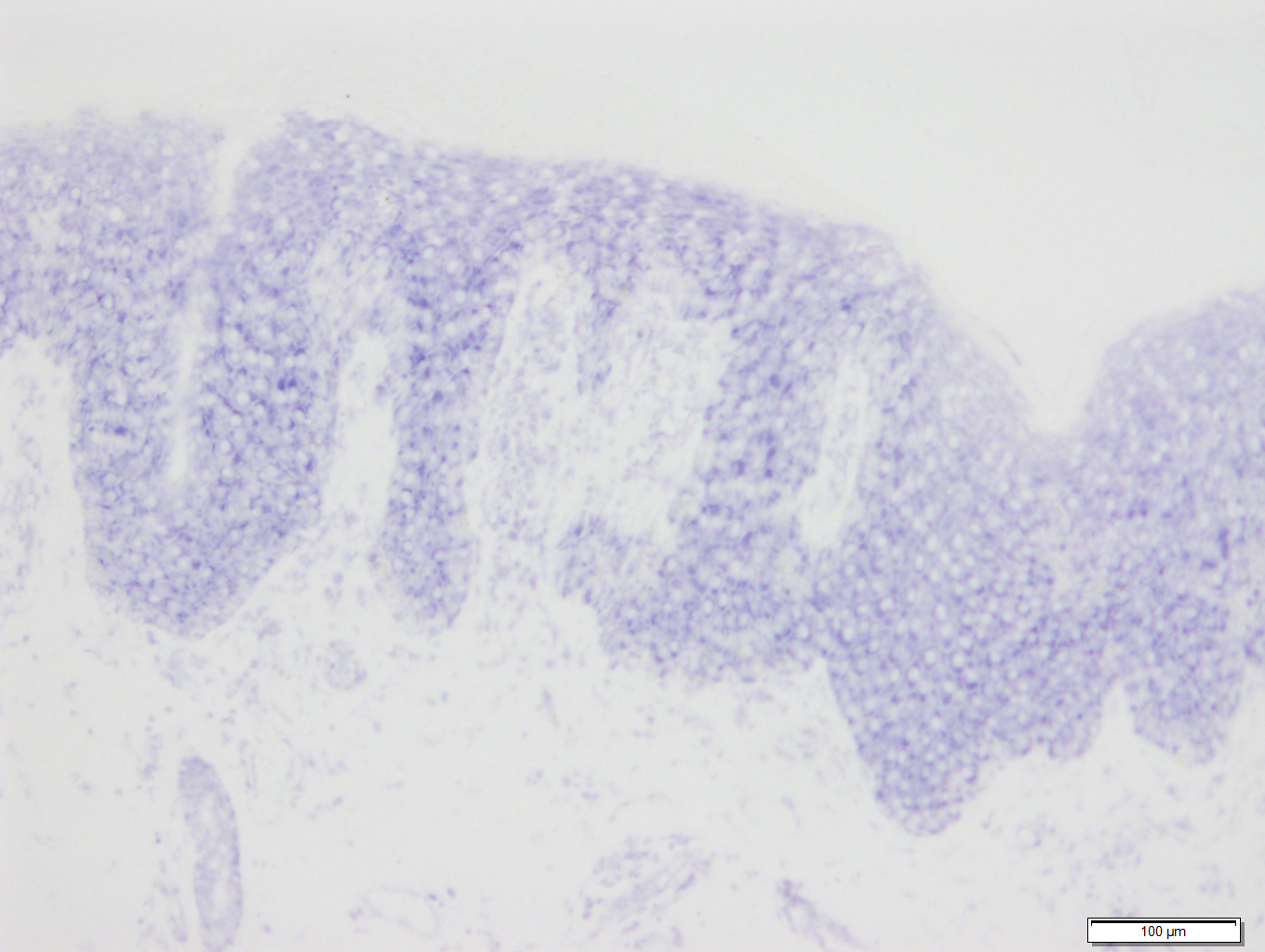

Supplement: Supplementary file 15 — Source Data for Figure 5 [file EMMM-15-e15674-s002.zip › Figure 5/5A/Ps-4.tif]

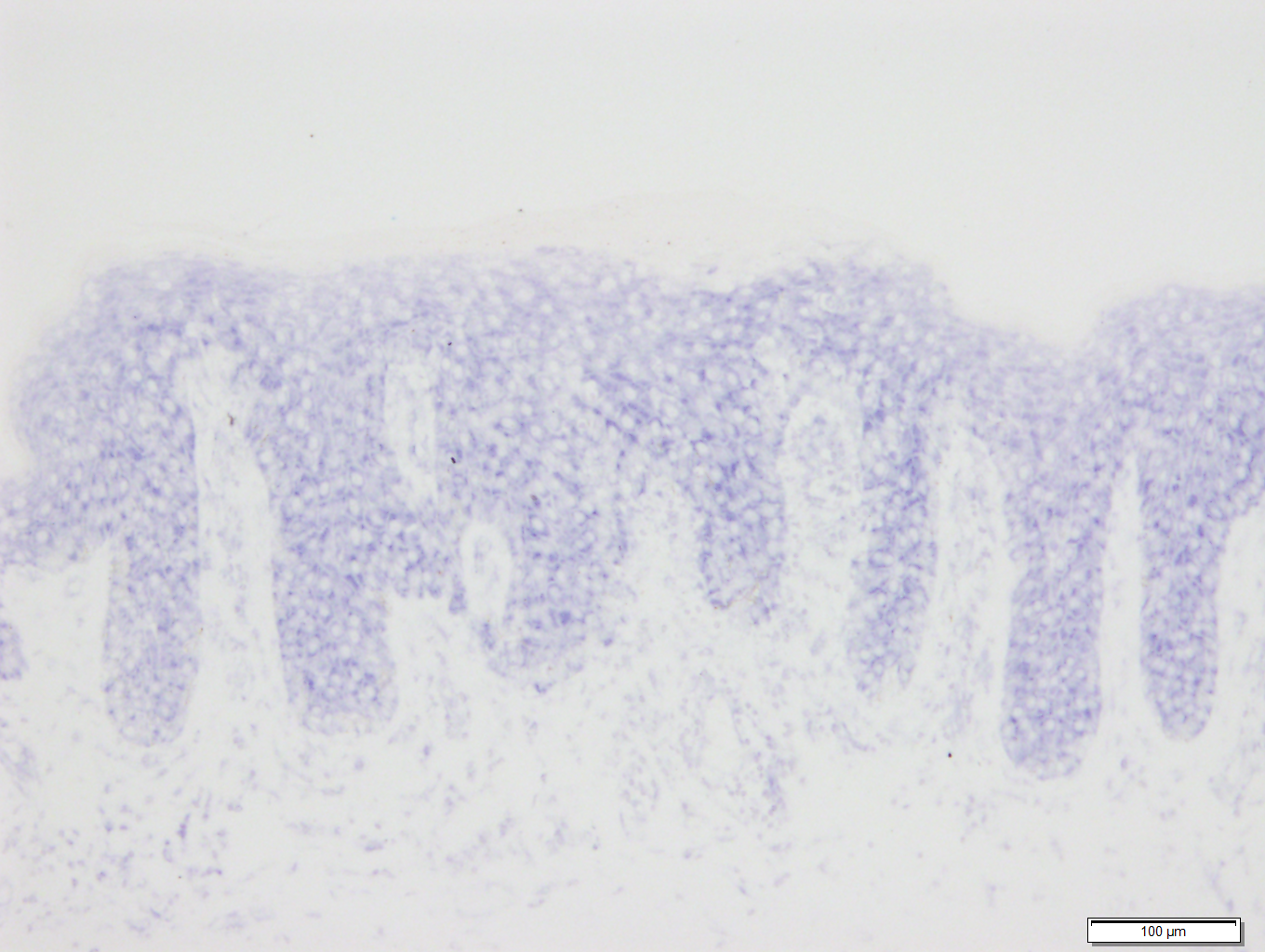

Supplement: Supplementary file 15 — Source Data for Figure 5 [file EMMM-15-e15674-s002.zip › Figure 5/5A/Ps-3.tif]

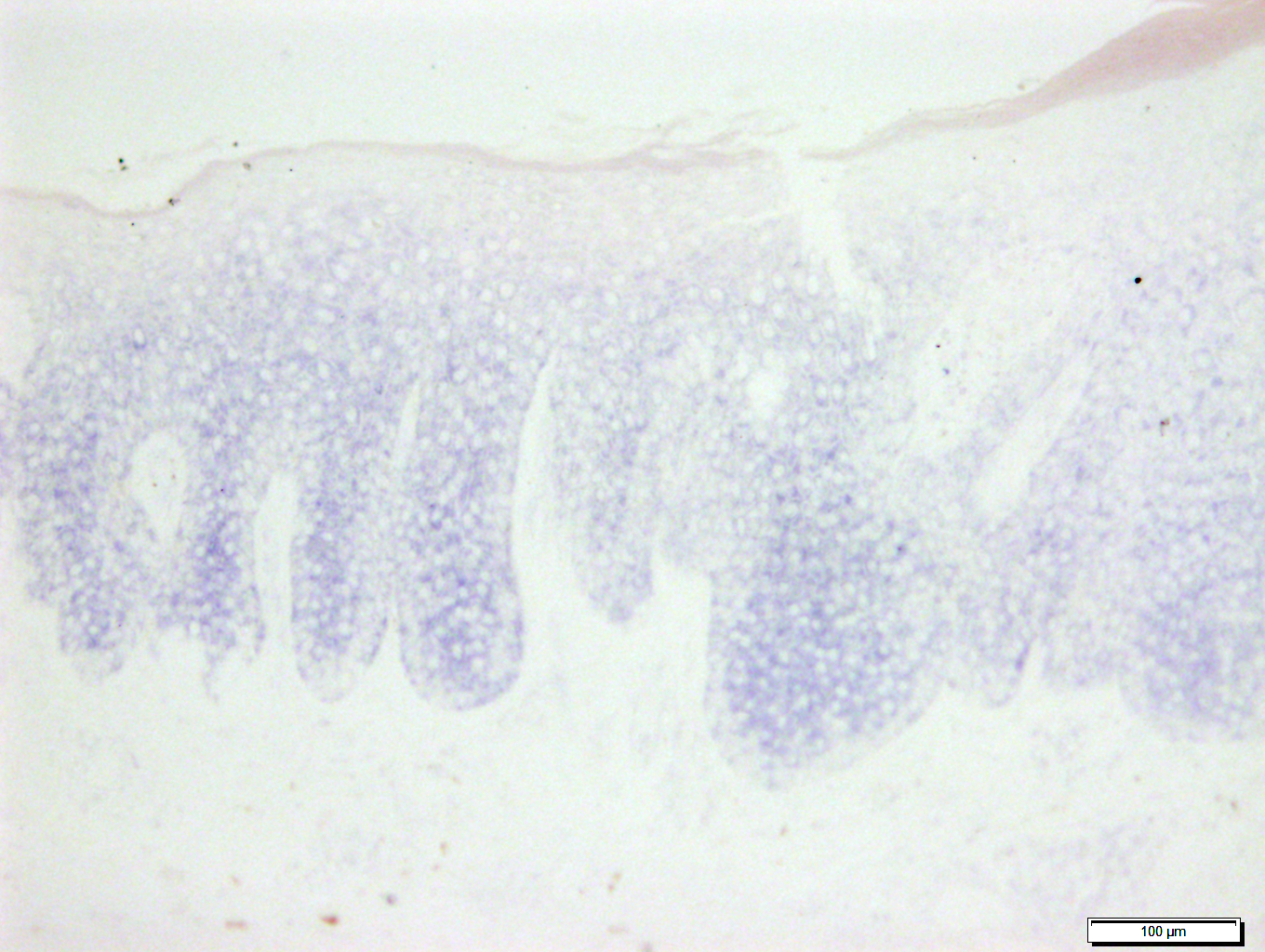

Supplement: Supplementary file 15 — Source Data for Figure 5 [file EMMM-15-e15674-s002.zip › Figure 5/5A/Ps-2.tif]

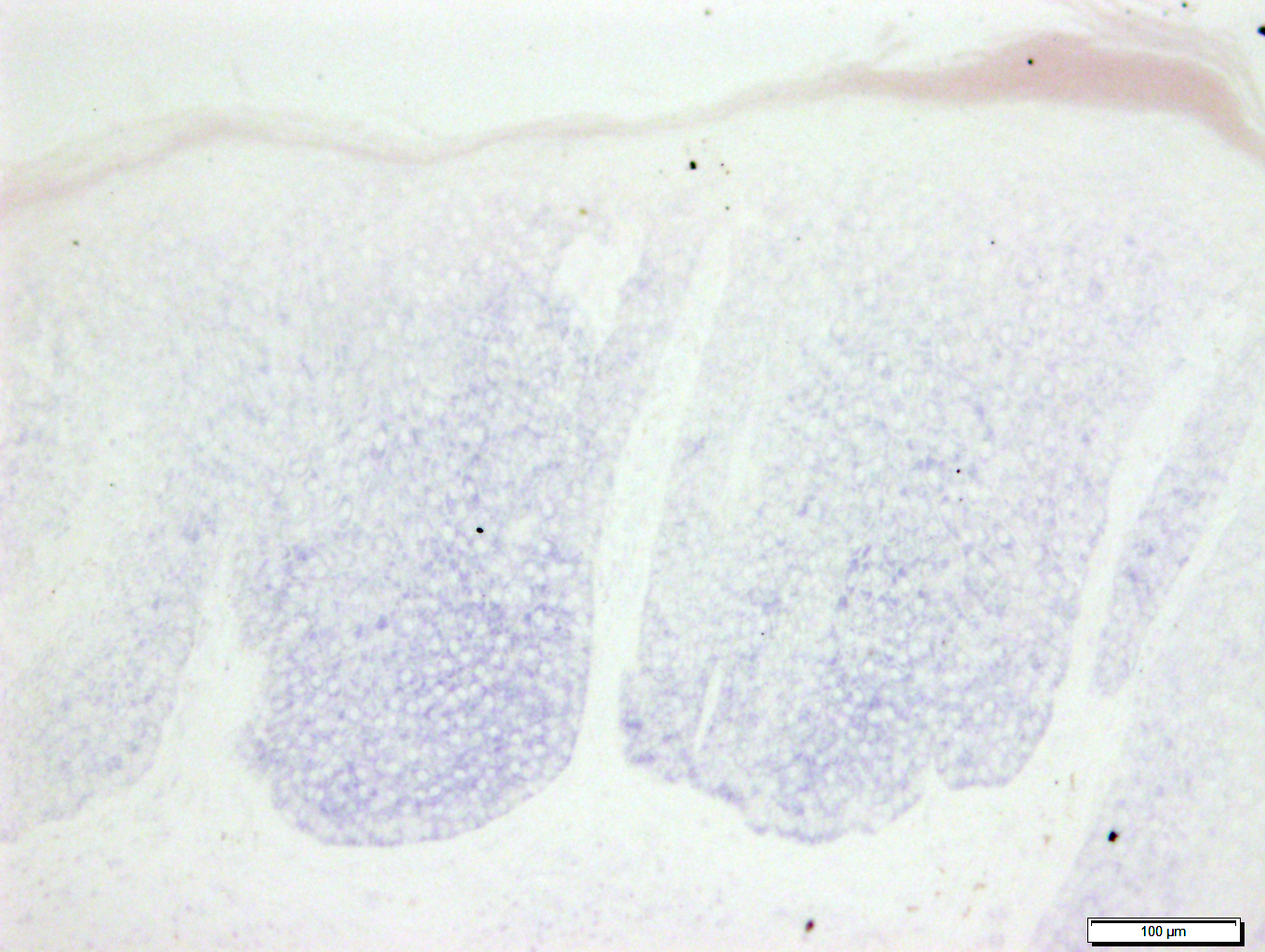

Supplement: Supplementary file 15 — Source Data for Figure 5 [file EMMM-15-e15674-s002.zip › Figure 5/5A/Ps-1.tif]
