# Supplementary material for: Metabolic rewiring in keratinocytes by miR‐31‐5p identifies therapeutic intervention for psoriasis
Source: EMBO Mol Med. 2023 Mar 1;15(4):e15674. doi: 10.15252/emmm.202215674 (PMC10086589; doi:10.15252/emmm.202215674)
Supplement: Supplementary file 11 — PDF+ [file EMMM-15-e15674-s001.pdf]

Article

SOURCE DATA | TRANSPARENT PROCESS | OPEN ACCESS

EMBO Molecular Medicine

Metabolic rewiring in keratinocytes by miR-31-5p identifies therapeutic intervention for psoriasis

Mao-Jie Wang[1,2,3,4], Huan-Jie Huang[1,2], Yong-Yue Xu[1], Harmjan Vos[2], Can Gulersonmez[2], Edwin Stigter[2], Johan Gerritsen[5], Marc Pages Gallego[6], Robert van Es[2], Li Li[1], Hao Deng[1], Lin Han[1], Run-Yue Huang[1,3,4,*], Chuan-Jian Lu[1,3,4,**] & Boudewijn MT Burgering[2,***]

Abstract

Besides genetic alterations, the cellular environment also determines disease onset and progression. When different cell types contribute to disease outcome, this imposes environmental challenges as different cell types likely differ in their extracellular dependencies. Hsa-microRNA-31-5p (miR-31) is highly expressed in keratinocytes of psoriatic skin, and we show that expression in keratinocytes is induced by limited glucose availability and enables increased survival under limiting glucose conditions by increasing glutamine metabolism. In addition, miR-31 expression results in not only secretion of specific metabolites (aspartate and glutamate) but also secretion of immunomodulatory factors. We show that this miR-31-induced secretory phenotype is sufficient to induce Th17 cell differentiation, a hallmark of psoriasis. Inhibitors of miR31-induced metabolic rewiring and metabolic crosstalk with immune cells alleviate psoriasis pathology in a mouse model of psoriasis. Together our data illustrate an emerging concept of metabolic interaction across cell compartments that characterizes disease development, which can be employed to design effective treatment options for disease, as shown here for psoriasis.

Keywords glutaminolysis; metabolism reprogramming; miR-31; psoriasis; T helper 17 cells
Subject Categories Immunology; Metabolism; Skin
DOI 10.15252/emmm.202215674 | Received 21 January 2022 | Revised 6 February 2023 | Accepted 7 February 2023 | Published online 1 March 2023
EMBO Mol Med (2023) 15: e15674

Introduction

Psoriasis is a chronic immune-mediated disorder of the skin that affects 0.1–0.4% of the total world population and is typically characterized by scaly, erythematous plaques on the extensor surfaces (Armstrong & Read, 2020; Parisi *et al*, 2020). The disease causes a negative impact on quality of life and is associated with additional pathologies, such as arthritis, cardiovascular disease, and metabolic syndrome (Parisi *et al*, 2020). The pathogenesis is characterized by a combination of keratinocyte hyperproliferation and inflammation mediated by activated immune cells, including neutrophils, dendritic cells, and Th17 cells (Greb *et al*, 2016).

Human disease, like psoriasis, is prevented by intrinsic and extrinsic control mechanisms that will remove aberrant cells before these can survive and establish a stable altered cellular compartment. To evade these control mechanisms, aberrant cells may adopt various strategies. A well-studied example is the various mechanisms by which cancer cells prevent clearance by the immune system. Immune cell activation oftentimes requires a shift in T cell metabolism toward glycolysis and hence increased glucose consumption. Cancer cells are similarly dependent on increased glucose consumption to sustain proliferation (Warburg effect) and cancer cells have been suggested to outcompete immune cells for glucose availability and thereby establish immune suppression (Garcia-Bermudez *et al*, 2018).

Strikingly, inflammatory skin disease, like psoriasis, is characterized by an increase in proliferation of skin cells, mostly keratinocytes combined with activation of glycolytic T cells. Efficient disease progression likely requires keratinocytes to adopt a metabolic switch that enables them to proliferate without conflicting with, or even supporting immune cell differentiation and activation, resulting in inflammation. Cellular adaptation oftentimes requires changes in gene expression programs and these can be brought about through not only epigenetic changes but also by expression of microRNAs (miRNAs), non-coding RNAs of 21–25 nucleotides that can regulate mRNA, and consequent protein expression of hundreds of genes simultaneously.

A number of miRNAs have been implicated in psoriasis pathology (Joyce *et al*, 2011), including microRNA-31-5p (miR-31;

1 The Second Affiliated Hospital, Guangzhou University of Chinese Medicine (Guangdong Provincial Hospital of Chinese Medicine), Guangzhou, China
2 Oncode Institute and Molecular Cancer Research, Center for Molecular Medicine, University Medical Center Utrecht, Utrecht, The Netherlands
3 Guangdong Provincial Key Laboratory of Clinical Research on Traditional Chinese Medicine Syndrome, Guangzhou, China
4 Guangdong-Hong Kong-Macau Joint Lab on Chinese Medicine and Immune Disease Research, Guangzhou University of Chinese Medicine, Guangzhou, China
5 Metabolic Diagnostics, Department of Biomedical Genetics, Centre for Molecular Medicine, University Medical Centre Utrecht, Utrecht, The Netherlands
6 Oncode Institute and Department of Genetics, Center for Molecular Medicine, University Medical Center Utrecht, Utrecht, The Netherlands
*Corresponding author. Tel: +86 15889932190; E-mail: ryhuang@gzucm.edu.cn
**Corresponding author. Tel: +86 13822250116; E-mail: lcj@gzucm.edu.cn
***Corresponding author. Tel: +31 887568918; E-mail: m.t.burgering@umcutrecht.nl

© 2023 The Authors. Published under the terms of the CC BY 4.0 license.

Xu *et al*, 2013; Wang *et al*, 2019). Interestingly, miR-31 expression is also implicated in various types of cancer (reviewed in Valastyan & Weinberg, 2010; Laurila & Kallioniemi, 2013) and appears therefore associated in general with increased proliferation. Therefore, we chose to study the role of miR-31 in psoriasis pathology, and in order to obtain a more integrated view of miR-31 function and regulation of keratinocyte proliferation, we used a combination of proteomics and metabolomics and focused on the regulation of metabolic processes by miR-31.

Interestingly, we find that miR-31 expression impacts glutamine metabolism at several levels. Glutamine through reductive or oxidative metabolism can provide cells with the majority of building blocks and efficiently maintain cell viability even in the absence of glucose (Altman *et al*, 2016). In agreement, miR-31 expression in keratinocytes enables survival and proliferation of keratinocytes under limiting glucose conditions by switching to glutamine-dependent metabolism. Interestingly, miR-31 expression itself is induced by limiting glucose availability. In psoriasis miR-31, expression is restricted to the keratinocytes in the spinous layer, whereas the majority of proliferating keratinocytes are located within the basal layer and require increased GLUT1-dependent glycolysis to sustain proliferation (Zhang *et al*, 2018). As nutrients including glucose have to pass the basal layer to reach the spinous layer, the observation of increased miR-31 expression can be explained by reduced glucose in the spinous layer due to excessive proliferation in the basal layer, which may even be amplified further by increased glucose consumption required for T cell activation. This reciprocal interaction between keratinocytes and immune cells in psoriasis is further enforced by our observation that miR-31 expression results in glutamine-dependent secretion of metabolites and cytokines that enable Th17 differentiation. Consequently, breaking the cross-talk between T cells and keratinocytes by inhibiting glutaminase (GLS), a key enzyme in glutamine metabolism, or SLC1A3, a key transporter of aspartate uptake, alleviates psoriasis in a mouse model. Taken together, our results illustrate how the extracellular environment can be involved in disease progression and how this knowledge may be employed to tailor treatment.

Results

miR-31 expression induces extensive changes in the cellular proteome and metabolome

We confirmed increased miR-31 expression in psoriasis by comparing skin biopsies from healthy control and psoriatic patients (Fig EV1A). We observed similar increase in miR-31 host gene expression in publicly available data (Fig EV1B) and in the imiquimod (IMQ)-induced mouse model for psoriasiform hyperplasia (van der Fits *et al*, 2009; Fig EV1A). Potential target mRNAs have been identified for miR-31 (reviewed in Laurila & Kallioniemi, 2013), yet mostly these proposed targets have been studied on an individual basis, whereas a specific miRNA can target simultaneously up to a few hundred of different mRNA species, and thus a simultaneous change in the expression of many proteins. Importantly, this indicates that miRNAs regulate biology in a systemic rather than singular manner. To study the full spectrum of miRNA-31-induced protein deregulation, we made use of a quantitative proteomic strategy (stable isotope labeling by amino acids in cell culture, SILAC, Fig EV1C). Ectopic miR-31 expression in two different human cell lines, HEK 293 T, and the keratinocyte cell line HaCaT, induced extensive changes in protein levels (Fig EV1D and E). Pearson correlation indicated a high concordance between SILAC proteome results obtained with these cell lines ($R^2 = 0.449$, $P < 0.001$, Fig EV1F) and a significant overlap in both cell lines of downregulated (279) and upregulated (187) proteins (Fig 1A; Dataset EV1). To further evaluate the quality of our proteomics results, we selected a set of validated miR-31 targets (meaning a target that at least is validated by reporter assay, western blot, or qPCR according to miRTarBase database [http://mirtarbase.mbc.nctu.edu.tw/; Table EV1]). Comparison with our results confirmed the regulation of several of these validated miR-31 targets in both cell lines (e.g., hypoxia-inducing factor1 alpha inhibitor [HIFAN], forkhead box-O transcription factor 3 [FOXO3], NUMB-endocytic adaptor protein [NUMB], and AT-rich interaction domain 1A [ARID1A; Fig EV1G and H]). Next, we checked our data for expression of 301 metabolic genes and 137 of these were detected in all replicates. When compiling these data, we identified 15 high-confidence hits for metabolic proteins downregulated following miR-31 expression. Importantly, a majority of downregulated metabolic genes (13/15) harbor at least one miR-31 seed sequence (Fig 1B; Dataset EV2).

As miR-31 expression resulted in significant metabolic protein expression changes, we next performed LC–MS metabolomics to obtain an unbiased view of metabolic changes induced by miR-31 expression. Principal component analysis (PCA, Fig EV1I) and heatmap clustering (Fig 1C; Table EV2) showed a clear separation in metabolite expression profiles between HEK293aCaT cells expressing miR-31 or a scrambled miRNA mimic. Importantly, we observed metabolite changes to corroborate protein expression changes. For example, in agreement with downregulation of ribose 5-phosphate isomerase A (RPIA) at the protein level and its role in the pentose phosphate pathway (PPP), we observed a reduced level of PPP intermediates (ribulose-5P, glucose-6P, and seduheptulose-7P). Also, changes in glutamine/glutamate levels are consistent with the observed downregulation of glutamine synthetase (GS) in the proteome analysis. Pathway GO-term analysis using metabolite levels as input also indicated extensive miR-31-induced dysregulation of glucose and glutamine metabolisms (Fig 1D).

Combination of proteomic and metabolic data indicates that miR-31 expression, through simultaneous deregulation of the expression of multiple metabolic genes, may impact glycolytic, lipid, and amino acid metabolism (summarized in Fig 1E), and deregulated gene expression appears to correlate by and large with changes in metabolite levels.

Regulation of glucose metabolism by miR-31

We next explored in detail how miR-31 expression regulates the above-identified metabolic pathways. To analyze the role of miR-31 expression in glucose metabolism (Fig 2A), we first measured uptake of the fluorescent glucose compound 2-[N-(7-nitrobenz-2-oxa-1,3-diazol-4-yl) amino]-2-deoxyglucose (2-NBDG) as a proxy for glucose uptake and observed on average a reduction in 2-NBDG uptake of 20–30% after ectopic miR-31 expression (Fig 2B). This reduction was observed for several cell lines including two additional keratinocyte cell lines (CCD-1106 (Hsieh *et al*, 2014) and CCD-1102KERTr). Expressing an anti-miR for miR-31 did not, or only mildly, increase

 © 2023 The Authors

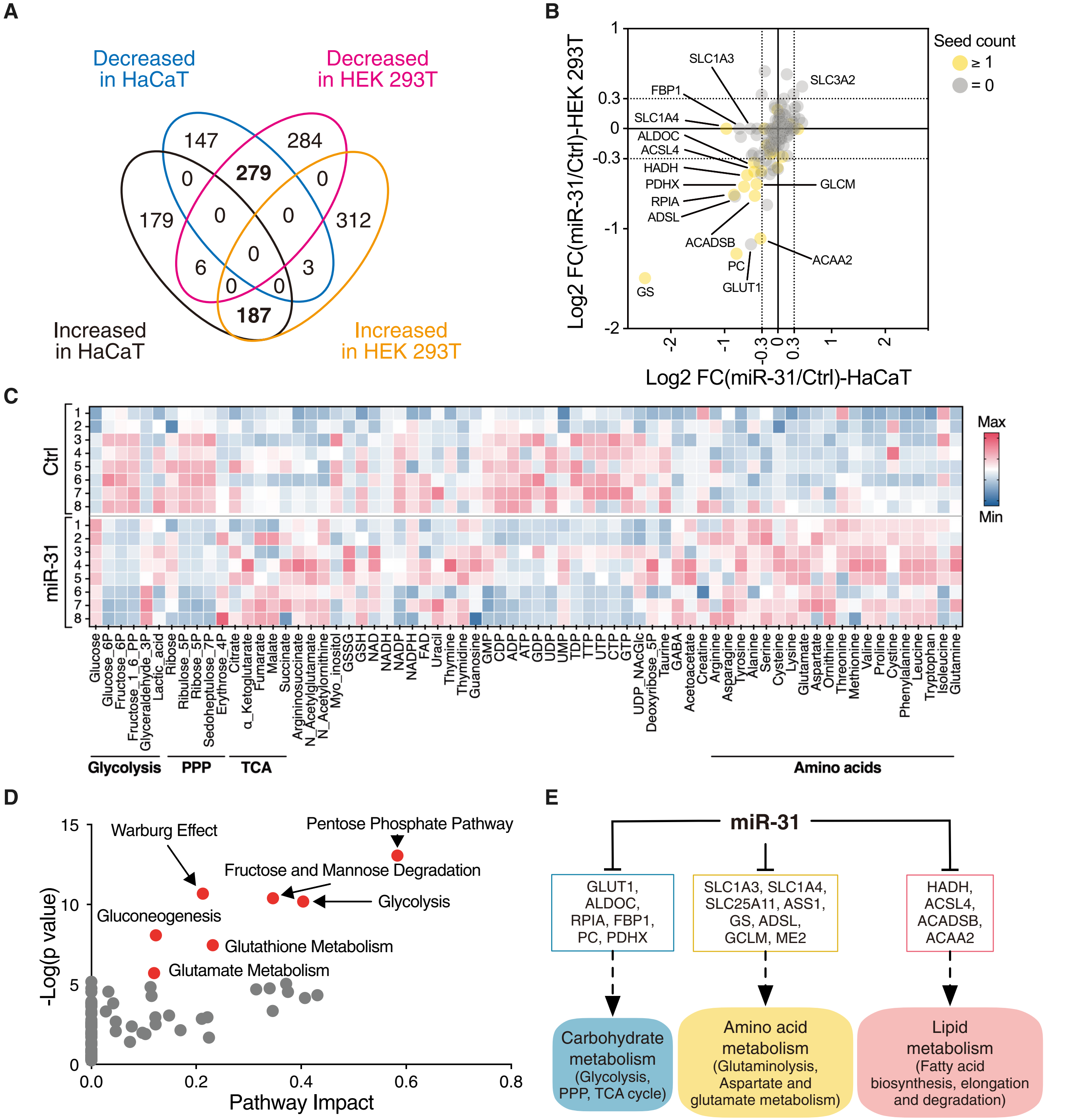

Figure 1. MiR-31 induces redirection of cell metabolism.

A Venn diagram depicting the number of proteins increased or decreased in HaCaT cells and HEK 293 T cells upon miR-31 overexpression.

B Dot plots of logarithmic fold changes in metabolic gene expression in HEK 293 T cells (y-axis) and HaCaT cells (x-axis) are indicated. Yellow-labeled genes contain at least one binding seed of miR-31-5p in the 3′UTR.

C Heatmap representation of the metabolite profile of HEK 293 T cells upon miR-31 overexpression. PPP, pentose phosphate pathway; TCA, tricarboxylic acid cycle.

D Metabolic pathway enrichment analysis using miR-31-induced significant changed metabolites as input. Those most predicted pathways are highlighted in red.

E Summary of the impact of miR-31 on cell metabolism.

© 2023 The Authors

2-NBDG fluorescence (Fig 2B), possibly reflecting a low level of endogenous miR-31 expression. Furthermore, miR-31-induced reduction in 2-NBDG fluorescence was independent of glucose concentration (Fig EV2A). These findings are consistent with miR-31-repressing glucose uptake. In keratinocytes, GLUT1 acts as the major glucose transporter (Zhang *et al*, 2018) and our proteomics data showed reduced expression of GLUT1 despite lacking an obvious miR-31 seed sequence in the 3′UTR (Fig 1B). We confirmed reduced GLUT1 expression following miR-31 expression by immune fluorescence microscopy and RT–qPCR (Fig EV2B and C). GLUT1 is a low Km and ATP-independent glucose transporter, and hexokinase (HK)-mediated glucose-6-phosphate formation is the rate-limiting step for glucose utilization (Ishihara *et al*, 1994). We observed miR-31-induced reduction in mRNA expression of HK1 and HK2 (Fig 2C) and this corroborates the observed reduction in glucose-6-phosphate (Fig 1C). In addition, expression of all three phospho-fructose kinase isozymes is reduced upon miR-31 expression (PFKM, PFKL, and PFKP; Fig 2C). Combined with the reduced protein and mRNA expression of aldolase (ALDOC, Figs 1B and 2C), this suggests that miR-31 expression reduced glucose uptake, which also results in decreased levels of glycolytic intermediates and possibly reduced glycolysis. However, when measuring extracellular acidification rate under normal culture conditions using Seahorse technology, we did not observe a significant miR-31-induced change in lactate production (Fig 2D). Importantly, under limiting glucose conditions, miR-31 expression significantly enhanced lactate production upon glucose addition (Fig 2E left panel and Table EV3). This is accompanied by increased lactate dehydrogenase (LDH) activity (Fig EV2D). Our SILAC data indicate that miR-31 expression also reduced pyruvate dehydrogenase complex member X (PDHX) and pyruvate carboxylase (PC) expression (Fig 1B) and this was confirmed by RT–qPCR (Fig EV2E) and western blotting in either HaCaT or normal human epidermal keratinocytes (NHEK; Figs 2F and EV2F). Both enzymes regulate entry of pyruvate into the mitochondria, and therefore, decreased expression may divert pyruvate flux toward lactate. In agreement, under low-glucose conditions, similar to miR-31 expression, pharmacological inhibition of pyruvate entry by UK-5099, a potent inhibitor of the mitochondrial pyruvate carrier (MPC1, Fig 2G; Table EV3), as well as siRNA-mediated knockdown of PC increased glycolytic flux toward lactate after glucose addition (Fig 2H; Table EV3). This suggests that miR-31, despite reducing glucose uptake, maintains glycolytic flux toward lactate by reducing pyruvate entry into mitochondria.

Alongside increasing lactate production following glucose stimulation, miR-31 expression increased basal mitochondrial O_2 consumption in the absence of glucose, yet this was not further increased by glucose addition. Importantly, this result is identical to UK-5099-mediated MPC inhibition, and siRNA-mediated knockdown of PC showed a similar trend (data no showed). The latter is in agreement with the study of Cheng *et al* (2011) showing that PC levels and activity regulate the choice between glucose and glutamine-mediated anaplerosis (Cheng *et al*, 2011), whereby reduced PC increases glutamine-dependent anaplerosis. Taken together, miR-31 expression reduced glycolytic flux toward mitochondrial metabolism while upholding lactate production, and miR-31 induced an increase in mitochondrial O_2 consumption. These results thus suggest that miR-31 induces mitochondrial activity through alternative sources, like glutamine metabolism.

Regulation of glutamine metabolism by miR-31

Our proteomic analysis (Fig 1B) showed a strong downregulation of glutamine synthase (GS) following miR-31 expression making GS a prime target in mediating possible miR-31-induced glutamine-dependent anaplerosis (schematic representation Fig 3A). Reduced GS expression induced by miR-31 was confirmed by immunoblotting (Figs 3B and EV2F). In addition, to GS regulation, we also observed a small increase in expression of glutamate oxaloacetic transaminase (GOT1) and the mitochondrial aspartate/glutamate transporter (AGC1/SLC25A12; Fig 3B). Consequently, miR-31 expression increased glutamine uptake, resulting in an apparent surplus of glutamate production as we observed profound glutamate secretion (Fig 3C). Glutamate can be exported through various transporters including the cystine–glutamate antiporter (xCT), a heterodimer consisting of SLC7A11 and SLC3A2. miR-31 enhances mRNA expression of both components (Fig EV2G). However, cystine uptake is only slightly increased by miR-31 (Fig 3C). This may suggest that additional glutamate transporters are being used (e.g., SLC1A3 or SLC1A4) and that usage of the xCT antiporter is indirectly determined by intracellular conversion of glutamate toward glutathione and that the observed miR-31 induced reduction in glutamate–cysteine ligase modifier (GCLM, Fig 1B), which therefore constrains cystine uptake. Glutamine-to-glutamate conversion requires glutaminase (GLS), and CB-839 is a specific pharmacological inhibitor of GLS. Inhibition of GLS reduced basal glutamine uptake, but miR-31 expression still induced an increase in glutamine uptake albeit to a lower level compared to the untreated condition. CB-839 inhibited miR-31-induced glutamate secretion (Fig 3C), suggesting that the low level of glutamine uptake induced by miR-31 is indeed not, or only minimally, converted to glutamate. Next, we tested a role for miR-31 in glutamine-dependent anaplerosis under low-glucose condition. As shown, miR-31 expression increased mitochondrial oxygen consumption under low-glucose condition, and this was dependent on extracellular glutamine (Fig 3D; Table EV3). Glutamate cannot replace glutamine in supporting miR-31-dependent mitochondrial metabolism or miR-31-induced lactate production (Fig EV2H). Furthermore, the miR-31-induced increase in both mitochondrial metabolism and lactate production was inhibited by CB-839 treatment (Fig 3E; Table EV3). Substrate supply for oxidative phosphorylation (OXPHOS) is controlled in many ways and the malate/aspartate shuttle (MAS) plays a role by regulating pyruvate supply and the NAD+/NADH balance between cytosol and mitochondria. Increased expression of AGC1 and GOT1 following miR-31 expression suggests also a key role for MAS in miR-31-induced mitochondrial metabolism and lactate production. Indeed, AGC1 knockdown reduced miR-31-driven mitochondrial metabolism and lactate production (Fig 3F; Table EV3). Combined, these results indicate that miR-31 stimulates anaplerosis through oxidative glutaminolysis and stimulates aspartate transport to the cytosol to generate pyruvate.

To further corroborate our observations, we performed 15N,13C-glutamine tracing (Fig 3G). As we predominantly observed miR-31 to stimulate glutaminolysis under low-glucose conditions (Fig 3D), we performed tracing under glucose-free conditions and added a low amount of unlabeled glutamine to prevent cell death before adding tracer glutamine. This resulted in rapid (within 30 min) labeling of most metabolites. In agreement with lactate production

 © 2023 The Authors

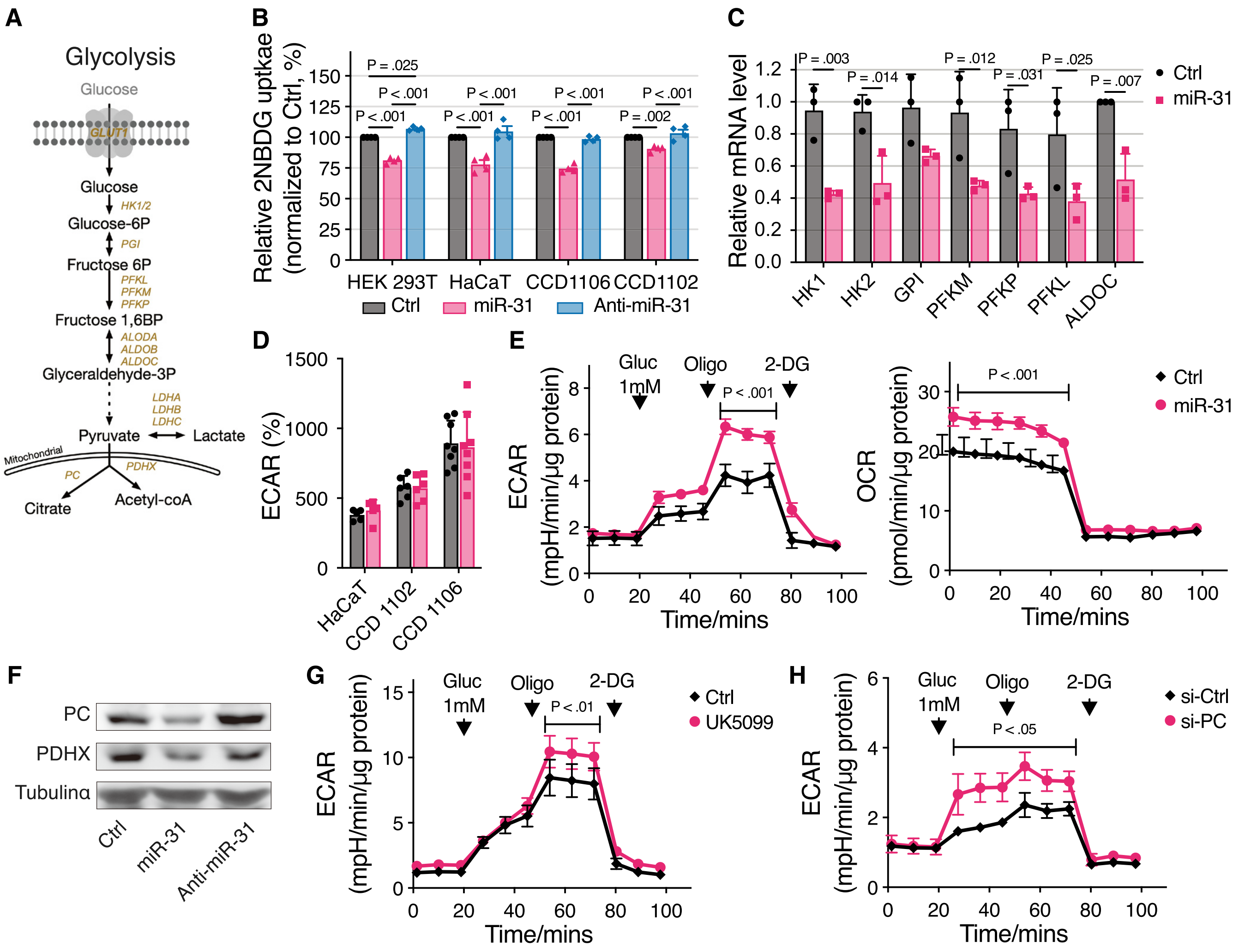

Figure 2. MiR-31 inhibits glycolysis but not affecting lactate production.

A Schematic picture of the glycolysis pathway.

B Measurement of 2-NBDG uptake in different cell lines by flow cytometry (*n* = 4 biological replicates).

C Quantitative RT–PCR detection of glycolytic gene expression levels in HaCaT cells upon miR-31 overexpression. β-actin was used as a housekeeping control gene (*n* = 3 biological replicates).

D Glycolysis stress test of three different cell lines (HaCaT, *n* = 5 biological replicates; CCD1102, *n* = 6 biological replicates; and CCD1106, *n* = 8 biological replicates) upon miR-31 overexpression. ECAR, extracellular acidification rate was baselined to third measurement. Gluc, glucose, 10 mM was applied; Oligo, oligomycin; 2-DG, 2-deoxy-D-glucose.

E Glycolysis stress test of HaCaT cells upon miR-31 overexpression in 1 mM glucose condition (*n* = 5 biological replicates). ECAR (left panel) and oxygen consumption rate (OCR, right panel) were normalized to the amount of protein.

F Western blot analysis of PC and PDHX expressions in HaCaT cells, and tubulin α was used as loading control. PC, pyruvate carboxylase; PDHX, pyruvate dehydrogenase complex component X.

G, H Glycolysis stress test of HaCaT cells upon UK5099 (G, *n* = 4 biological replicates) or PC siRNA (si-PC, H, *n* = 5 biological replicates) in 1 mM glucose condition. ECAR and OCR were normalized to the amount of protein.

Data information: In (B–E), (G), and (H), data are presented as mean ± SD (Two-way ANOVA with Turkey test for (B) and (D) and Sidak test for (C), (E), (G), and (H)). Source data are available online for this figure.

being maintained by miR-31 (Fig 2E), we observed that miR-31 increased lactate labeling derived from glutamine (Fig 3H) after 24 h culture, indicating that lactate production is the consequence of a combination of increased glutaminolysis and reduced pyruvate entry into mitochondria. We observed an additional miR-31-induced increase in cellular malate and fumarate labeling, showing miR-31-induced anaplerosis of the TCA cycle. More strikingly, we observed a strong increase in miR-31-induced secretion of not only glutamate but also aspartate, oxaloacetate, and to a lesser extent, malate and fumarate (Fig 3G). Glutamate and aspartate secretion following miR-31 expression was independently confirmed using LC–MS platform for analysis of biogenic amines (Fig 3I). These results combined suggest that miR-31 expression results in increased glutamine-to-glutamate conversion by inhibiting GS, however, the fate of

© 2023 The Authors

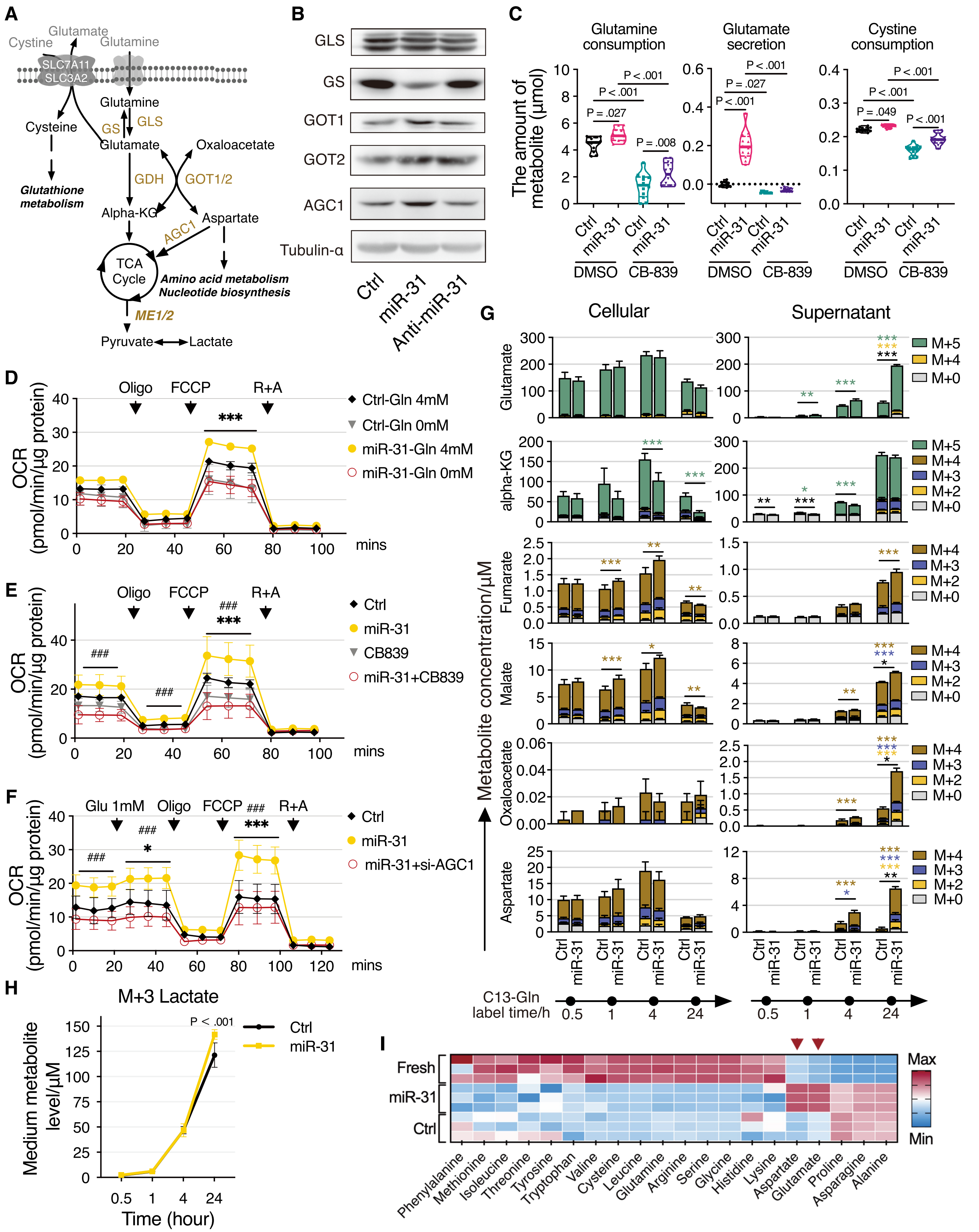

Figure 3.

 © 2023 The Authors

Figure 3. MiR-31 enhances glutaminolysis of HaCaT cells to produce glutamate and aspartate.

A Schematic diagram of glutaminolysis pathway. TCA cycle, tricarboxylic acid cycle; Alpha-KG, alpha-ketoglutarate; GS, glutamine synthetase; GLS, glutaminase; GDH, glutamate dehydrogenase; GOT1/2, glutamic-oxaloacetic transaminase 1/2; AGC1, aspartate/glutamate transporter; ME1/2, malic enzyme 1/2.

B Western blot analysis of GLS, GS, GOT1, GOT2, and AGC1 in HaCaT cells upon miR-31 overexpression, and tubulin α was used as loading control.

C Violin plot of glutamine consumption, glutamate secretion, and cystine consumption of HaCaT cells upon different treatments ($n = 12$ biological replicates).

D Mitochondrial stress test of HaCaT cells upon miR-31 overexpression ($n = 3$ biological replicates). Cells were pre-cultured in medium with or without glutamine overnight. OCR was normalized to the amount of protein. FCCP, carbonyl cyanide 4-(trifluoromethoxy) phenylhydrazone; R + A, antimycin and rotenone.

E, F Mitochondrial stress tests of HaCaT cells upon CB839 (E, $n \geq 4$ biological replicates) and AGC1 siRNA (si-AGC1, F, $n \geq 3$ biological replicates) treatments under 1 mM glucose supplementation.

G Cellular and extracellular levels of metabolites were detected by targeted metabolomics using C13 glutamine tracing protocol in a time course ($n = 3$ biological replicates).

H Line graph showing the extracellular expression levels of M + 3 lactate in HaCaT cells after overexpression of miR-31 over time ($n = 3$ biological replicates).

I Heatmap representation of relative amino acid concentrations in cell culture medium of HaCaT cells upon miR-31 overexpression.

Data information: In (C–H), data are presented as mean ± SD. Compared with ctrl, *$P < 0.05$; **$P < 0.01$; ***$P < 0.001$ (by one-way ANOVA with Sidak test for (C), two-way ANOVA with Sidak test for (D–F) and (H), and two-way ANOVA with Turkey test for (G)); MiR-31 compared to miR-31 + CB-839 in (E) or miR-31 + si-AGC in (F), $^{###}P < 0.001$ (two-way ANOVA with Sidak test).
Source data are available online for this figure.

glutamate is determined by miR-31-dependent regulation of other metabolic enzymes like PC/PDHX and GCLM/AGC1. Together, this results in not only miR-31-facilitated glutamine-dependent anaplerosis under glucose-limiting conditions, and this produces aspartate, but also a significant part of the metabolites (glutamate and oxaloacetate) is secreted (schematic representation Fig EV2I).

To determine the relevance of GS regulation compared to the other miR-31-induced changes in metabolic enzyme expression, we addressed whether reduced GS expression only fully phenocopies miR-31 expression in regulating glutaminolysis. Interestingly, siRNA-mediated reduction in GS expression, similar to miR-31 expression, also decreased glucose uptake (Fig EV2J), whereas reducing expression of PC did not affect glucose uptake (Fig EV2K). In contrast to miR-31 expression, siRNA-mediated knockdown of GS resulted in decreased glycolysis toward lactate (Fig EV2L; Table EV3) and also decreased mitochondrial metabolism (Fig EV2M; Table EV3). As individual regulation of these miR-31 targets does not phenocopy miR-31-induced metabolism, these results strongly imply that the mechanism whereby miR-31 couples glucose and glutamine metabolism results from miR-31-dependent regulation of GS combined with PC/PDHX and GCLM/AGC1 regulation.

In psoriasis, miR-31 is expressed in the spinous layer that consists predominantly of differentiated cells, proliferating cells being mostly restricted to the basal layer. To study whether our results are specific to proliferating cells or can also be observed in differentiated cells *in vitro*, we made use of $CaCl_2$ treatment to induce differentiation of HaCaT cells *in vitro* as illustrated by increased expression of keratin-10 (KRT14) marking the cells of the spinous layer (Wang *et al*, 2010; Fig EV3A). Ectopic expression of miR-31 in $CaCl_2$-treated HaCaT cells showed that similar to proliferating HaCaT cells, miR-31 induced downregulation of GS (Fig EV2F). Also, in the $CaCl_2$-induced differentiated cell model, miR-31 expression significantly enhanced lactate production upon 1 mM glucose re-addition (Fig EV3B) and miR-31 expression increased mitochondrial oxygen consumption under low-glucose conditions dependent on extracellular glutamine (Fig EV3C). Finally, differentiated HaCaT cells still secreted significant levels of aspartate and glutamate upon ectopic miR-31 expression (Fig EV3D). Combined, these data show that by and large, the metabolic consequences of miR-31 expression are similar in proliferating versus differentiated HaCaT cells.

Consequence of metabolic regulation on proliferation and inflammation

Psoriasis and comparable skin diseases emerge through a combination of hyperproliferative keratinocytes and local inflammation. To test consequences on disease parameters of metabolic redirection by miR-31 expression, we first tested cell growth of HaCaT cells under limiting glucose or glutamine conditions. Under normal culture conditions, miR-31 expression did not affect cell proliferation, but miR-31 expression reduced cell proliferation under glutamine-restricted conditions, and in contrast, enhanced cell proliferation under glucose-limiting conditions (Fig 4A). Mechanistically, these effects on cell proliferation can be explained not only by miR-31-induced reduction in GS, and consequently, enhanced glutaminolysis, but also by reduced PC and PDHX expressions following miR-31 expression, which renders cells more dependent on glutamine for anaplerosis (Cheng *et al*, 2011). In contrast, siRNA-mediated reduction of GLS expression or pharmacological inhibition by CB-839 makes cells dependent on glucose for anaplerosis (Cheng *et al*, 2011). In agreement, we observed synthetic lethality in HaCaT cells between miR-31 expression and CB-839 treatment, whereas anti-miR-31 expression increased survival after CB-839 treatment (Figs 4B and EV3E). This GLS dependence of miR-31-expressing cells further corroborates that upon miR-31 expression, cells move from endogenous glutamine production to exogenous glutamine uptake as a source of glutamine.

As the miR-31 effect on cell proliferation is dependent on the consequent metabolic changes, we addressed whether metabolic changes in reverse affect miR-31 expression, and interestingly, we observed that glucose restriction by itself induces expression of miR-31 (Fig 4C) and also reduced GS expression (Fig 4D). Together, this suggests that glucose availability can couple in an inverse manner to glutaminolysis by regulating expression of miR-31 and consequent expression of GS and other miR-31 targets.

Crosstalk between keratinocytes and immune cells mediated by cytokines is important in maintaining the diseased state (reviewed in Lowes *et al*, 2014). Previous studies (Xu *et al*, 2013; Yan *et al*, 2015) identified miR-31 as a pro-inflammatory factor in psoriasis, showing that miR-31 activated the NF-kB pathway and thereby increased secretion of a set of cytokines. Indeed, ectopic expression

© 2023 The Authors

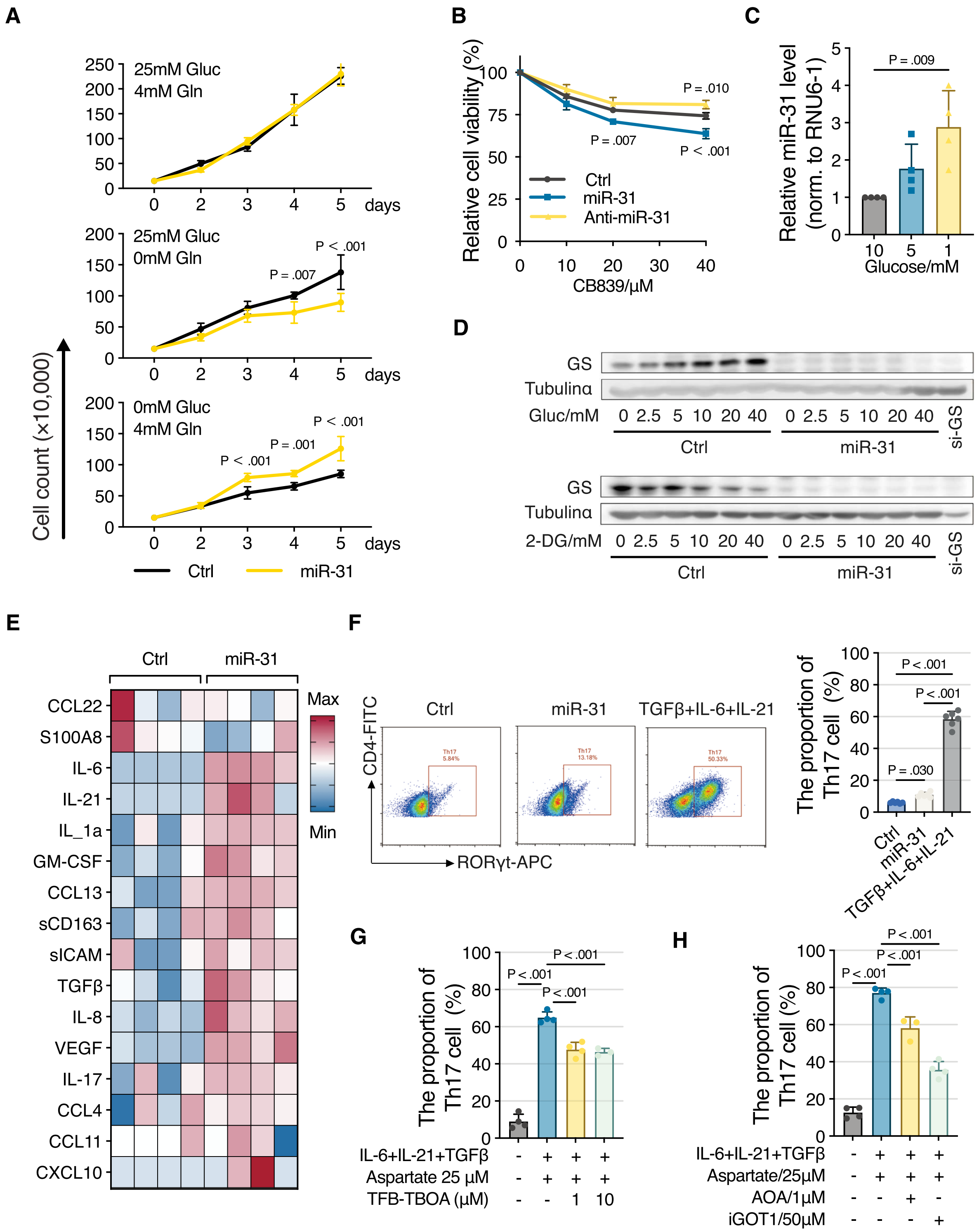

Figure 4.

of miR-31 in HaCaT cells induced expression and secretion of a variety of cytokines (Fig 4E; Table EV4). Importantly, these include IL-6, IL-21, and TGFb1 cytokines that have been shown to contribute to Th17 differentiation (reviewed in Guo & Zhang, 2021), a T cell subtype that marks psoriasis.

MiR-31-induced secretome induces Th17 differentiation

To test a functional consequence of the miR-31-induced secretome, we tested differentiation of naïve CD4+ T cells towards Th17 cells, as this T cell subtype specifically marks psoriasis. *In vitro* naïve

© 2023 The Authors

Figure 4. Consequences of metabolic regulation on proliferation and inflammation.

A Cell growth curves of HaCaT cells upon miR-31 overexpression in medium with or without glutamine or glucose as indicated (*n* = 5 biological replicates).
B Cell viability of HaCaT cell was measured by MTS assay (*n* = 4 biological replicates).
C Quantitative RT–PCR detection of Has-miR-31-5p expression levels in HaCaT cells exposed to a range of glucose (*n* = 4 biological replicates). RNU6-1 was used as house control gene.
D Western blot analysis of GS in HaCaT cells with miR-31 overexpression under different concentrations of glucose or 2-DG treatment, and tubulin α was used as a loading control.
E Heatmap representation of relative cytokine production in HaCaT cells upon miR-31 overexpression.
F MiR-31-induced secretome enhances Th17 differentiation and was analyzed by FACS (*n* = 6 biological replicates).
G, H FACS analysis of Th17 differentiation upon TFB-TBOA treatment (G, *n* = 4 biological replicates) or AOA and iGOT1 treatments (H), $n \geq 3$ biological replicates in each group.

Data information: In (A–C) and (F–H), data are presented as mean ± SD (by two-way ANOVA with Turkey test for (A) and (B), and one-way ANOVA with Sidak test for (C) and (F–H)).
Source data are available online for this figure.

CD4+ T cells can be differentiated to Th17 cells by treating with a cytokine cocktail, including IL-1β, IL-6, TGFβ, IL-23, IFN-γ, and IL-4 (Zhou *et al*, 2008 and review in van den Berg & McInnes, 2013). As these IL-6, IL-21, and TGFb1 are secreted by miR-31-expressing HaCaT cells, we first tested whether addition of a conditioned medium of miR-31-expressing HaCaT cells to naïve CD4+ T cells resulted in Th17 differentiation. Indeed, we observed a consistent two- to threefold induction of Th17 differentiation by addition of a conditioned medium (Fig 4F). Arguably, in this experimental setting, the concentrations of cytokines are not optimal for induction of Th17 differentiation as for comparison, the addition of a purified cytokine cocktail at likely high concentration resulted in considerably larger induction of Th17 differentiation (Fig 4F). Differentiation induced by this cytokine cocktail is not affected by co-treatment with succinate, fumarate, malate, citrate, or α-ketoglutarate (Xu *et al*, 2017). As we observed that miR-31 induced aspartate and glutamate secretion besides secretion of a subset of the cytokines involved *in vitro* Th17 differentiation, we treated naïve $CD4^+$ T cells with combinations of different miR-31-regulated cytokines and metabolites. We observed that Th17 differentiation induced by miR-31-induced cytokines TGFb1, IL-6, and IL-21 can be further increased in a concentration-dependent manner by aspartate but not glutamate (Fig EV3F). Recently, it has been shown that glutaminolysis in mice stimulates Th17 differentiation (Johnson *et al*, 2018; Kono *et al*, 2018), and therefore the observation that glutamate addition did not induce additional differentiation was unexpected. However, high extracellular glutamate inhibits cystine uptake by the cystine/glutamate antiporter (system xCT; Dixon *et al*, 2012) and consequently, results in increased levels of ROS that even impair Th17 formation (Johnson *et al*, 2018). In this respect, glutamate may not simply replace glutamine.

The SLC1A3 inhibitor, (2S, 3S)-3-[3-[4-(trifluoromethyl) benzoylamino]benzyloxy] aspartate (TFB-TBOA), inhibits cellular uptake of aspartate by cancer cells (Garcia-Bermudez *et al*, 2018), and in agreement, TFB-TBOA significantly inhibited *in vitro* Th17 differentiation in the presence of aspartate (Fig 4G). In addition, aspartate taken up by the naïve CD4+ T cells is metabolized to oxalacetate by GOT1, a process described to be essential for the differentiation of Th17 cells (Xu *et al*, 2017). Consequently, we observed inhibitors of GOT1, aminooxyacetate (AOA), and iGOT1 also significantly inhibit *in vitro* Th17 differentiation in the presence of aspartate (Fig 4H). Taken together, these results indicate that miR-31 expression in keratinocytes can result in the secretion of cytokines and metabolites that combined can support in part the inflammatory response by stimulating Th17 differentiation.

Roles for miR-31-induced metabolic regulation in psoriasis

To study whether the miR-31-mediated switch to glutaminolysis and its effect on cell proliferation under glucose-limiting conditions *in vitro* is relevant to psoriasis *in vivo*, we first characterized expression of relevant parameters using immunohistochemistry (IHC). The biopsies of psoriasis patients used for IHC show typical epidermal thickening (Fig EV4A), increased proliferation (Ki67 staining, Fig EV4B), and displayed increased miR-31 expression, specifically in the spinous layer (Figs 5A and EV4C). Next, we tested expression GLUT1, GLS, and GS (Fig 5B). In normal skin, GLUT1 expression appears mostly restricted to cells present in the basal layer, and expression is lost when cells are moving upward into the spinous and granular layer. Also, in psoriatic lesions from human patients, GLUT1 expression appears mostly restricted to cells in the basal layer and strongly reduced in the upper layers (Figs 5B and EV4D). In contrast to GLUT1, GLS staining is high in all cell layers both in normal skin as well as in psoriatic skin (Figs 5B and EV4E). GS expression was low in normal skin but increased in cells of the basal layer of the skin in psoriatic lesions. However, in psoriatic lesions, GS expression was decreased in the spinous and granular layer concordant with miR-31-regulating GS and expression of miR-31 being low in the basal layer as compared to an increase in the spinous and granular layer (Figs 5B and EV4F). Taken together these data suggest that the skin displays metabolic compartments whereby the basal layer is GLUT1 positive and the upper layers are GLUT1 negative, but GLS positive.

To address causality, we made use of the IMQ mouse model for psoriasis. The IMQ model employed, similar to human patients, showed increased scaling (Fig 5C), but also an increase in miR-31 expression in the spinous layer (Fig EV5A). As we observed in cell culture synthetic lethality between miR-31 expression and GLS inhibition by CB-839, we tested the effect of CB-839 treatment in this model for psoriasis. Cutaneous application of CB-839 was compared to treatment with calcipotriol and betamethasone dipropionate gel (CBG, commercial name Xamiol), a commonly used treatment option for psoriasis (Kuehl & Shear, 2018). CB-839 treatment decreased epidermal thickness (Fig EV5B), lowered the histopathological score based on Baker's system (Fig EV5C,) and decreased keratinocyte proliferation as measured by Ki67 staining (Fig EV5D).

© 2023 The Authors

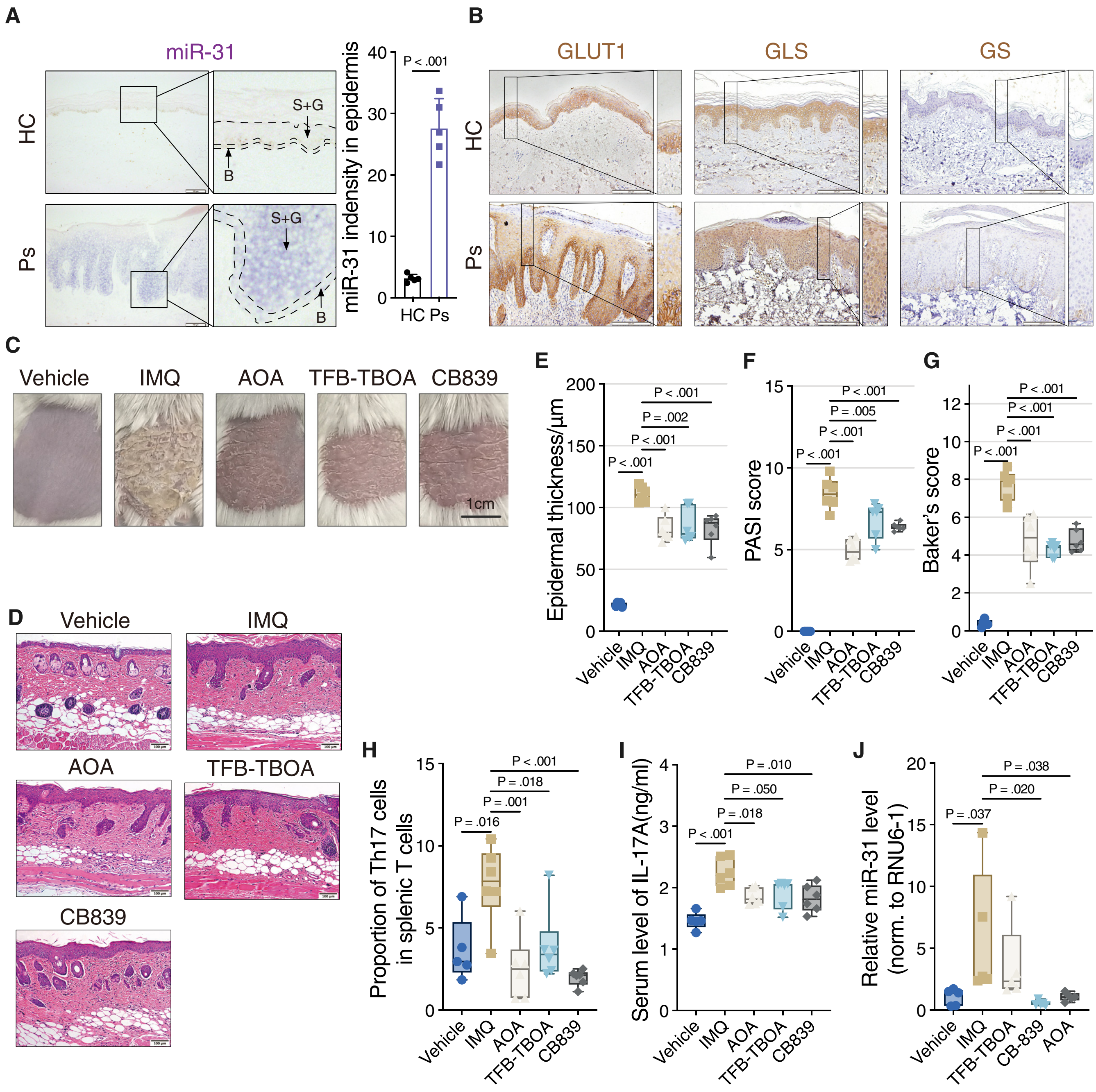

Figure 5. Pharmacological inhibition of miR-31-induced metabolic changes relieves psoriatic disease in an animal model.

A Representative picture of *in situ* hybridization (ISH) staining of miR-31 in skin biopsies of healthy individuals and psoriasis patients ($n = 5$ individuals in each group). Quantification of results showing the average level of miR-31 in skin epidermis (right panel). B, basal layer; S + G, spinous and granular layers. Scale bars: 100 μm.

B Representative pictures of immunohistochemical staining of GLUT1, GLS, and GS in skin biopsies of HC and Ps ($n = 5$ individuals in each group). Scale bars: 200 μm.

C Representative images of skin of mice ($n = 5$ mice in vehicle group and $n = 6$ mice in other groups). Scale bars: 1 cm.

D Representative pictures of hematoxylin–eosin staining of skin biopsies of mice treated with vehicle, IMQ, AOA, TFB-TBOA, and CB-839 ($n = 5$ mice in vehicle and $n = 6$ mice in other groups). Scale bars: 100 μm.

E–G Disease activity was evaluated by epidermal thickness (E), PASI score (F), and Baker's score (G). $n = 5$ mice in vehicle and $n = 6$ mice in other groups.

H FACS analysis of Th17 cell proportion in splenic lymphocytes of mice ($n = 5$ mice in vehicle and $n = 6$ mice in other groups).

I Enzyme-linked immunosorbent assay for the determination of IL-17A levels in serum of mice ($n = 5$ mice in Vehicle and $n = 6$ mice in other groups).

J Quantitative RT–PCR detection of mmu-miR-31-5p expression levels in skin of mice ($n = 5$ mice in each group).

Data information: Data of (A) are presented as mean ± SD and data of (E–J) are presented as box and whiskers (central band, median; whiskers, min to max; and show all points). Applied unpaired Student's *t*-test for (A), and one-way ANOVA with Sidak test for (E–J).
Source data are available online for this figure.

 © 2023 The Authors

In line with a role of miR-31 in regulating cytokine production by keratinocytes, we also observed a reduction in CD4+ T cells and myeloperoxidase-positive (MPO+ cells; neutrophil granulocytes), indicating reduced inflammatory response (Fig EV5E–G). In addition, CB-839 and CBG partially normalized the expression pattern of GLUT1, GLS, and GS (Fig EV5H–K).

As indicated, the presence of activated Th17 cells is characteristic of psoriasis, and we observed that the miR31-induced secretome harbors both cytokines and metabolites that can activate Th17 differentiation (Fig 4F). Therefore, we also tested the effect of the inhibitors for aspartate uptake (TFB-TOA) and conversion to oxaloacetate (AOA) in the IMQ model and compared these to CB-839. We observed that all these three inhibitors can alleviate psoriatic skin damage (Fig 5C) and induce consequent histopathological changes (Fig 5D). Most disease indexes, including PASI score, epidermal thickness, and baker score, were suppressed by AOA, TFB-TBOA, and comparable to CB-839 (Fig 5E–G). In addition, we collected the splenic lymphocytes from these mice and determined Th17 presence. In keeping with our *in vitro* data, AOA, TFB-TBOA, and CB-839 treatments induced a significant inhibitory effect on Th17 differentiation *in vivo* (Fig 5H).

Furthermore, mice treated with different inhibitors showed a decrease in the serum level of IL-17A induced by IMQ treatment of the mice (Fig 5I), whereas no significant difference was observed in the serum level of IL-10 (Fig EV5L). Importantly, RT–qPCR analysis showed that both CB-839 and AOA treatment in the IMQ model reduced miR-31 expression in the skin (Fig 5J).

Taken together targeting glutaminolysis by CB-839, or targeting the metabolic crosstalk between miR-31-expressing keratinocytes and naïve CD4+ T cells by TFB-TOA or AOA all showed similar or improved therapeutic effects compared to CBG/Xamiol in alleviating psoriatic symptoms in the IMQ model.

Discussion

Psoriasis is one of the most common human skin diseases characterized by excessive growth and aberrant differentiation of keratinocytes combined with a strong local inflammatory immune response. Many studies have addressed the genetic underpinning of psoriasis including the expression and potential role of aberrant miRNA expression. Here, we confirm high miR-31 expression in human biopsies of psoriasis and in the IMQ mouse model system for psoriasis. We present evidence that in cell lines, miR-31 expression induces a redirection of metabolism through combined deregulation of enzymes involved in glycolysis and glutaminolysis. We observed that miR-31 expression limits glucose flux toward mitochondrial metabolism, whereas at the same time, lactate production is not affected. Lactate production is maintained by miR-31 expression in part by reducing pyruvate entry into mitochondria through reduced PC and PDHX expression and in part through increased glutaminolysis, which also maintains mitochondrial respiration. miR-31 regulation of glutaminolysis occurs at multiple levels. Primarily, expression of miR-31 reduced GS expression resulting in increased glutamine uptake and excess glutamate and aspartate. Subsequently, miR-31 expression steers glutamate metabolism through the combined regulation of several metabolic enzymes/pathways. First, miR-31 expression increased glutamate secretion by regulation of the xCT antiporter (increased expression of SLC7A11 and SLC3A2), likely in combination with downregulation of GCLM. Furthermore, glutamate metabolism is determined by miR-31-dependent regulation of the mitochondrial malate–aspartate shuttle. Interestingly, we observed regulation of GOT1, MDH1, ME1, and AGC1 as compared to GOT2, MDH2, ME2, and SLC25A11, indicating that glutamine-directed anaplerosis of the TCA cycle additionally results in MAS-dependent secretion of aspartate and oxaloacetate into the cytosol and this is corroborated with the observed increase in these metabolites when measuring flux under glucose-limiting conditions (see for schematic overview Fig EV2I). Importantly, the mode of miR-31-dependent regulation of glutaminolysis allows cell survival under glucose-limiting conditions.

To test the metabolic consequences of miR-31 expression, we initially used a proliferating keratinocyte cell line, HaCaT cells, as a model, whereas in psoriasis, miR-31 is expressed in the spinous layer mostly consisting of non-proliferative or differentiated cells. Metabolism may differ between proliferating and non-proliferating cells albeit not necessarily. For example, glucose metabolism does not differ between proliferating and quiescent fibroblasts (Lemons *et al*, 2010). However, recently, proliferating mammary epithelial cells, especially in the context of cancer, have been shown to differ from quiescent mammary epithelial cells in glutamate metabolism (Coloff *et al*, 2016). Interestingly, quiescence reduced glutamine uptake and synthesis of non-essential amino acids derived from glutamate by reducing transaminase activity and increasing glutamate dehydrogenase (GLUD) expression. We used *in vitro* differentiation of HaCaT cells by $CaCl_2$ treatment (Pleguezuelos & Kapas, 2006) to test whether differentiation affects the redirection of metabolism by miR-31 compared to proliferative HaCaT cells. We observed equal downregulation of metabolic targets like GS by miR-31 and overall observed similar consequences in switching to glutaminolysis and importantly, also similar secretion of metabolites (aspartate and glutamate). Together suggesting that at least *in vitro* we do not observe major difference between proliferating and differentiated HaCaT cells with respect to the response toward ectopic miR-31 expression.

Inhibition of glutaminolysis by CB-839 treatment mitigates most of the effects of miR-31 on glutamine metabolism, suggesting that miR-31 redirects cells from endogenous glutamine production to exogenous glutamine uptake as a source of glutamine.

In keeping with our results showing differential expression of miR-31 and GLS/GS within the layers of healthy and psoriatic skin, we find that CB-839 treatment is effective in alleviating psoriatic disease in the IMQ mouse model. Furthermore, we provide evidence that miR-31-induced secretion of cytokines and aspartate is sufficient to induce Th17 differentiation *in vitro* and blocking SLC1A3-mediated aspartate uptake using TFB-TBOA, and further conversion to oxaloacetate by AOA reduces Th17 differentiation and importantly, also affect psoriasis pathology in the IMQ model similar to CB-839.

Many studies have reported a possible correlation among metabolic syndrome, diabetes, and the incidence of skin diseases such as psoriasis. However, a recent meta-analysis covering 26 clinical studies concluded that there is no clear support to suggest a link between defective glucose metabolism and psoriasis (Friis *et al*, 2019). In contrast, others (Zhang *et al*, 2018) and our results clearly provide support for the involvement of dysregulated metabolism in psoriasis, but not as a predisposition but rather as part of the mechanism that drives the disease when manifest.

© 2023 The Authors

Zhang *et al* (2018) demonstrated the involvement in psoriasis of increased GLUT1-mediated glycolysis in the proliferation of basal membrane cells, whereas our results indicate the importance of miR-31-mediated increase in glutaminolysis in the cells of the spinous layer. Interestingly, we observed that reducing glucose availability *in vitro* induces miR-31 expression. In addition, we observed that GLUT-1 expression is low or absent in the spinous layer of psoriatic skin of humans as well as in the IMQ model. Together, this may suggest that in psoriasis, glucose utilization by the keratinocyte cells in the basal layer and activated immune cells may limit glucose availability for cells in the spinous layer leading to consequent miR-31 expression in the spinous layer and survival under these conditions. This also results in secretion of cytokines and metabolites that increase Th17 cell differentiation. Zhang *et al* (2018) show that topical treatment with a GLUT1 inhibitor alleviates psoriasis pathology similar to the results we obtain by using inhibitors directed at glutaminolysis (CB839) and metabolic crosstalk (aspartate uptake [TFB-TBOA]). Importantly, whereas GLUT1 inhibition targets keratinocytes within the basal layer and glutaminolysis inhibition will target keratinocyte cells in the spinous layer, both approaches also target the immune cell compartment. In agreement, genetic deletion of GLUT1 in the skin rescued only epidermal acanthosis and proliferation, in contrast to the topical, chemical inhibition of GLUT1 which also reduced inflammation. Together, these results and ours demonstrate not only a complex interplay between the different cell compartments within the skin but also that inhibition of immune cell activation combined with targeting metabolic changes in either basal or spinous layer will have similar results in disease outcome.

Our results identify inhibition of glutaminolysis as a treatment opportunity for psoriasis. Currently, various psoriasis treatment regimens are available. Most recently, these are drugs targeting the IL-23/IL-17A pathway which are reasonably effective in relieving skin symptoms of psoriasis. However, more general treatment options, such as fumarate, are still clinically used (Wang & Tsai, 2017; Mrowietz *et al*, 2018). The mode of action of fumarate and its related compound dimethyl-fumarate in relieving psoriatic symptoms remains unclear (Mrowietz *et al*, 2018). Interestingly, recently, GAPDH has been suggested to be targeted by fumarate (Kornberg *et al*, 2018). Targeting glycolysis through GLUT1 inhibition as treatment option for psoriasis is suggested by Zhang *et al* (2018) and thus fumarate may have similar mode of action as GLUT1 inhibition in psoriasis treatment.

Anti-IL17 therapy is highly effective in psoriasis patients, indicating the importance of IL-17-dependent immune cells like Th17 cells. Glutaminolysis has been reported to control Th17 differentiation in mice (Xia *et al*, 2020; Miao *et al*, 2021) and in agreement herewith it has been shown that also the Th17 cells in psoriasis display increased glutaminolysis, likely through increased GLS1 expression (Johnson *et al*, 2018; Kono *et al*, 2018). Based on the requirement for glutaminolysis in Th17 differentiation, Xia *et al* (2020) also described the potential of GLS inhibition in the treatment of psoriasis. However, there it was suggested that the effect on psoriasis was due to GLS inhibition in T cells and consequently reduced Th17 differentiation. Here, we show that the involvement of glutamine metabolism in psoriasis acts beyond the differentiation of Th17 cells and is also part of the mechanism whereby keratinocytes contribute to this disease.

Limitation

We primarily used HaCaT cells for mechanistic studies, namely the consequences of miR-31 expression on protein expression and the metabolic consequences of deregulation of metabolic enzyme expression. However, despite being a keratinocyte cell line, HaCaT cells do not fully recapitulate the *in vivo* status of keratinocytes within the spinous layer of the psoriatic skin where miR-31 is expressed. However, we have observed that miRNA-mediated gene expression regulation is consistent between the cell lines we used and not dependent on cell type, the metabolic state of the different models may have impact on the consequences of the miR-31-induced metabolic reprogramming. Irrespective, we do observe that the miR-31 target genes are also responsive in primary keratinocytes, and importantly, that metabolic redirection induced by miR-31 expression translates as far as tested to the *in vivo* mouse model of psoriasis we employed.

We addressed metabolic rewiring in response to miR-31 using several approaches, with each technology having its intrinsic limitations. For example, Seahorse technology enables determination of mitochondrial activity by O_2 consumption, but this does by itself not inform what the underlying cause for a change in activity is. To mitigate in part these limitations, we used ^{13}C flux analysis that revealed that miR-31 mediates changes in the glutamine flux that affect mitochondrial metabolism rather than changes in activity of mitochondrial enzymes. This reinforces the importance of approaching (metabolic) questions from different angles for a comprehensive understanding of biological processes.

Materials and Methods

None of the described experiments were specifically blinded for analysis.

Cell culture

HEK293T and HaCaT cells were cultured in high-glucose DMEM (Gibco, 11960044) supplemented with 10% FBS and 1% penicillin–streptomycin. CCD 1102 KERTr (ATCC® CRL-2310™) and CCD 1106 KERTr (ATCC® CRL-2309™) were cultured in keratinocyte-serum free medium (Gibco, 17005-042) with keratinocytes supplements (Gibco, 37000-015) including bovine pituitary extract (BPE; Gibco, 13028-014) and human recombinant epidermal growth factor (EGF; Gibco, 10450-013). Normal human epidermal keratinocytes (NHEK) were obtained from Bio-connect (C-12003) and cultured in keratinocyte growth medium 2 (Bio-connect, C-20216) with supplement pack (Bio-connect, C-39011). All cells were cultured at 37°C and 5% CO2. Cell lines were monthly tested for mycoplasma infection using a PCR–ELISA method (Roche, 11663925910). Genotyping of cell lines is done on an irregular basis using an in-house SNP array profiling method.

Glucose uptake measurement

A non-radioactive assay was performed for the measurement of glucose uptake in different cell lines by using 2-NBDG (2-(N-(7-nitrobenz-2-oxa-1, 3-diazol-4-yl) amino)-2-deoxyglucose, Cayman chemicals, 11046–10), a fluorescent deoxyglucose analog. Briefly, 50 μM of 2-NBDG was added to the cell culture medium and then

 © 2023 The Authors

incubated in a cell incubator at 37°C for 1 h. The cells were then collected and washed twice with PBS. We stained with PI for about 5 min to identify live cells, and finally, loaded into Celesta flow cytometer (BD Biosciences) for FACS analysis.

Proliferation measurement

For cell growth curve experiments, 150,000 cells were first seeded in six-well plates and treated with scramble miRNA mimic (Ctrl, Merck, HMC0002-5NM) or miR-31 mimic (miR-31, Merck, HMI0466-5NM) overnight in complete medium. Next day, replaced the medium with the 10% FBS glucose and glutamine-free DMEM medium (Life Technologies, A1443001) supplemented with indicated metabolites (25 mM glucose + 4 mM glutamine; 25 mM glucose + 0 mM glutamine; and 0 mM glucose + 4 mM glutamine) for cell incubation. The corresponding medium was changed every 3 days. The cells were collected at the indicated times and stained and counted using Trypan blue (Bio-Rad, 145-0013). For colony-forming assay, HaCaT cells were seeded in six-well plates and were transfected with miR-31 mimic or inhibitor for 36 h, followed by 10 μM CB-839 (Bio-connect, HY-12248) addition. Cell culture medium and CB-839 were refreshed every 3 days for a total of 14 days of treatment. For staining, cells were washed with PBS twice, fixed with 100% methanol 20 min, and stained with 0.25% crystal violet (Sigma, C0775) in 6.25% ethanol for 30 min at room temperature, washed three times with ddH2O, dried at room temperature, and photographed.

MTS assay

Cells were plated in 96-well microtiter plate and received different treatments before MTS assay (Abcam, ab197010). For the MTS assay, 20 μl of MTS reagent was directly added into the culture medium and incubated at 37°C incubator for 3 h. Finally, the formazan dye is quantified by measuring the absorbance at 490 nm.

RNA extraction and quantitative real-time–PCR

Total mRNA was extracted following the manufacture of Qiagen RNeasy Kit (Qiagen, 28106), and reversed transcribed using the iScript cDNA Synthesis Kit (Bio-Rad, 1708891). qReal-time–PCR was performed using SYBR Green FastStart Master Mix (Merck Life Science, 4913914001) in the CFX Connect real-time–PCR detection system (Bio-Rad). Data were normalized to beta-actin and presented as a relative expression level that was calculated by using delta delta Ct analysis. Most of primer sequences of targeted genes were derived from primer bank (https://pga.mgh.harvard.edu/primerbank/) and described in Table EV5. For quantification of microRNA expression, mature miR-31 was quantified using MystiCq® microRNA–qPCR Assay (Sigma, MIRRM00-100RXN) according to the manufacturer's instructions. Primer of miR-31 was ordered from Sigma (MIRAP00090) and RNU6-1 was used as the internal control (Sigma, MIRCP00001).

Stable isotope labeling of amino acids in cell culture (SILAC) experiment setup

For SILAC labeling, HaCaT cells were cultured in SILAC-labeled DMEM (Thermo Fisher Scientific) with 10% dialyzed FBS and 1% of penicillin/streptomycin-containing L-arginine and L-lysine (light medium) or 13C6-L-arginine and 3C6-L-lysine (heavy medium) for at least 14 days to eliminate non-labeled arginine and lysine. The day before the transfection, 1.5×10^5 cells were seeded in a six-well plate in a final volume of 3 ml. On the next day, the medium was changed and the 60–70% confluent cells were transfected with a specific miRNA mimic either for miR-31 mimic or control miRNA mimic at a final concentration of 30 nM together with lipofectamine RNAiMAX (Fisher scientific, 10601435) and Opti-MEM. A 48 h post-transfection, cells were harvested by adding lysis buffer containing 8 M urea, 1 M ammonium bicarbonate, 10 nM tris (2-carboxyethyl) phosphine, and 40 nM chloroacetamide. Cell lysates were incubated at 95°C for 5 min, sonicated, and diluted to 2 M Urea with 1 M ABC. After protein quantification, using the BCA protein assay, cell lysates from miR-130a- or miR-708-transfected cells generated from heavy medium and SCR-transfected cells from light medium were mixed 1:1 (reverse mode) and vice versa (forward mode). Proteins were digested overnight with 2% (w/w) trypsin, next peptides were fractionated based on their molecular mass using ultra-high-performance liquid chromatography (UltiMate-3000 system, Thermo Fisher Scientific), and finally, desalted and acidified on a C-18 cartridge (3M, Saint Paul, Minnesota, USA). C18-stagetips were activated with methanol, washed with buffer containing 0.5% formic acid in 80% ACN (buffer B) and then with 0.5% formic acid (buffer A). After loading of the digested sample, stagetips were washed with buffer A and peptides were eluted with buffer B, dried in a SpeedVac, and dissolved in buffer A. Peptides were electro-sprayed directly into an Orbitrap Fusion Tribrid Mass Spectrometer (Thermo Fisher Scientific) and analyzed in Top Speed data-dependent mode. Raw files were analyzed using Maxquant software. For identification, the human Uniport 2017 was searched with both the peptide as well as the protein false discovery rate set to 1%. Proteins identified were filtered for reverse and decoy hits, standard contaminants, and selected to have more than one unique or razor peptide by using the Perseus software 1.5.1.6. Heavy/light normalized ratios (ratio reverse and ratio forward) were used to quantify protein expression and were further processed for comparative analysis of differential expression among the conditions.

Western blot analysis and immunofluorescence

Cells were lysed in sample buffer (0.2% m/v SDS, 10% v/v glycerol, 0.2% v/v β-mercaptoethanol, and 60 mmol/L Tris pH 6.8) for protein extraction. Proteins were detected using 7.5–12.5% SDS–PAGE gels and subsequent western blot analysis with primary antibodies detected by HRP-conjugated secondary antibody. Unless stated otherwise, alpha-tubulin or vinculin was used as loading control. For immunofluorescence, cells were fixed with 3.7% paraformaldehyde in PBS, permeabilized with 0.1% v/v Triton in PBS, and blocked with 2% m/v BSA (Sigma). Cells were subsequently incubated with primary antibody (see Table EV6 for the information of antibodies) and visualized with Alexa secondary antibody (Sigma).

LDH activity assay

LDH activity was measured using a Lactate Dehydrogenase Activity Assay Kit (Sigma, MAK066) according to manufacturer's instruction.

© 2023 The Authors

Bioenergetics

Seahorse Bioscience XFe24 Analyzer was used to measure extracellular acidification rates (ECAR) in mpH/min and oxygen consumption rates (OCR) in pmol O2/min. Cells were seeded in six-well plates and transfected with indicated miRNA mimic (scramble or miR-31-5p) or indicated si-RNAs (si-PC, si-GS, and si-AGC1) for 36 h, or treated with pharmaceutical drugs (CB-839; 10 mM final concentration) or UK 5099 (25 mM final concentration, Merck life science, PZ0160) for 24 h. Thereafter, cells were seeded in XF24 polystyrene cell culture microplates (Agilent, 100882) at a density of 10,000 cells per well. One hour before the measurements, culture medium was replaced, and the plate was incubated for 60 min at 37°C. For the mitochondrial stress test, culture medium was replaced for Seahorse XF Base medium (Agilent, 102353), supplemented with 20 mM glucose (Sigma-Aldrich), 2 mM L-glutamine (Sigma-Aldrich), 5 mM pyruvate (Sigma-Aldrich), and 0.56 μl NaOH (1 M). During the test, 5 μM oligomycin, 2 μM FCCP, and 1 μM of rotenone and antimycin A (all Sigma-Aldrich) were injected to each well after 18, 45, and 63 min, respectively. For the glycolysis stress test, culture medium was replaced for Seahorse XF Base medium supplemented with 2 mM L-glutamine and 0.52 μl/ml NaOH (1 M). Sensor cartridges (pre-hydrated in XF calibrant solution overnight in a CO_2-free incubator) were loaded with glucose (Port A), oligomycin (Port B), and 2-deoxyglucose (2-DG, Port C) to achieve concentrations of 1 mM, 2 μM, and 50 mM, respectively, after injection. After injections, measurements of 2 min were performed in triple, preceded with 4 min of mixture time. The first measurements after oligomycin injections were preceded with 5 min mixture time, followed by 8 min waiting time for the mitochondrial stress test and 5 min mixture time followed by 10 min waiting time for the glycolysis stress test. Both ECAR and OCR were normalized to individual protein amount, and data were analyzed using the XF Mito Stress Test Report Generator.

Clinical specimens

Skin specimens were obtained from the Guangdong Provincial Hospital of Chinese Medicine (China) with Institutional Review Board approval (BF2019-108-01), and written informed consent was obtained from all patients. A total of 10 cases, including 5 cases of psoriasis patients and 5 cases of healthy people with an age ranging from 23 to 46 years (mean, 37 years) and 31 to 49 years (median, 38 years), respectively, were enrolled in this study. Samples were formalin-fixed paraffin embedded and were furtherly used for *in situ* hybridization detection of miR-31 and immunohistochemistry staining of specific proteins. The experiments conformed to the principles set out in the WMA Declaration of Helsinki and the Department of Health and Human Services Belmont Report.

Animal experiments

An imiquimod-induced mouse model (IMQ) of psoriasis was introduced in this study. Eight-week-old BALB/c mice were obtained from Guangdong Experimental Animal Center (Guangzhou, China) and were raised in 12 h light/dark cycles under a specific pathogen-free (SPF) environment. The mice were fed a standard diet and had free access to water. To establish an IMQ model, 50 mg of imiquimod cream (5%; Med-Shine Pharmaceutical, China) was applied topically onto the dorsal skin of mice continuously for 6 days after dorsal hair was shaved and chemically depilated. In this study, two independent animal experiments were processed. Mouse experiments were performed in parallel to study the therapeutic effect of CB-839, TFB-TBOA, and AOA in psoriasis. Starting from the second day of imiquimod induction, BALB/c mice were randomly assigned to receive DMSO (Vehicle), CB839 (0.6 mg/mouse/day), TFB-TBOA (0.2 mg/mouse/day, Tocris, 2532), AOA (0.5 mg/mouse/day, Selleck, S4989), or calcipotriol and betamethasone dipropionate gel (50 μg/mouse/day, commercial name: Xamiol, LEO laboratories Ltd., USA) topically prior to imiquimod treatment in a total of 6 days. The mouse experiment was approved by the Animal Ethics Committee of Guangdong Provincial Hospital of Chinese Medicine (China, No. 2018068). No animals were excluded from analysis.

Immunohistochemistry (IHC) and Hematoxylin & Eosin Y staining (HE)

Biopsies were harvested from dorsal skin of mice and fixed with 10% neutral buffered formalin (NBF) about 48 h. After paraffin embedding, all samples were cut into 4 μm sections and attached to microslide. Heat-induced antigen repair was performed by using a pressure cooker and naturally cooled down about 10 min. Sections were deparaffinized, heat retrieved (buffer with 1 citrate buffer, pH 6, cooked for 2 min in a pressure cooker, kept in 94–96°C for 10 min, and cooled naturally), perforated (0.2% TBST, 10 min), blocked in 2% BSA (Sigma, A9418), and then incubated with antibodies listed in Table EV6. The immunostaining was performed using an EliVision System-HRP DAB (MXB, DAB-2031). In the end, sections were counterstained with hematoxylin, dehydrated, and cover slipped. HE was performed under the manufacturer's instructions. The staining was quantified using Image J. All IHC and HE staining shown are representative of three or more sections.

In situ hybridization

For miR-31 *in situ* hybridizations, digoxigenin (DIG)-labeled probes were used following the manufacturer's protocol (Qiagen, 339459). Both DIG-labeled miR-31 and scrambled probes (Qiagen, YD00610651) were hybridized at 61°C. U6 probe was used as the positive control. *In situ* signals were detected by staining with Anti-DIG-AP antibody (Sigma, 11093274910) and developed using NBT/CBIP purple substrate (Sigma, 11697471001). Sections were counterstained with nuclear fast red (VECTOR, H3403) for 1 min and washed by flowing water. The quantification was performed by using Image J.

LC–MS amino acid assay

A volume of 15 μl medium was transferred to a labeled 1.5 ml Eppendorf vial. A volume of 285 μl 80% acetonitrile also containing internal standards (final concentration of 10 μM) was added and the sample was thoroughly mixed. The sample was centrifuged for 10 min at 17,000 *g* in an Eppendorf centrifuge. A volume of 250 μl was transferred to a new, labeled Eppendorf vial

© 2023 The Authors

and evaporated to dryness. Cell samples harvested in 300 μl ice-cold methanol were subjected to the same protocol. The residue was dissolved in 70 μl of borate buffer (pH 8.2) by thorough mixing and the derivatization was started by adding 20 μl of AccQ-Tag reagent solution prepared according to the suppliers' protocol (Waters, Etten-Leur, The Netherlands) after which the samples were vortex mixed and incubated at 55°C for 10 min. The sample was evaporated to dryness and the residue was dissolved in 120 μl 10% acetonitrile containing 0.1 mM formic acid and transferred to an LC sample vial.

Analysis was performed on a system consisting of an Ultimate 3000 LC and an LTQ-Orbitrap XL (Thermo Scientific, Breda, The Netherlands). As a column, a Waters HSS T3 (2.1 × 100 mm, 1.8 μm) was used, kept at a temperature of 40°C in the column oven. Eluent A used for analysis was milliQ water containing 0.1% formic acid and eluent B consisted of acetonitrile containing 0.1% formic acid. The LC gradient used for separation commenced by injecting 1 μl of sample and started at 0% B for 5 min, followed by a 10 min linear gradient to 75% B. In 0.5 min, the gradient increase to 100% B and kept there for 1 min before returning to 0% B. The column was allowed to regenerate for 2.5 min prior to a next analysis. Total runtime was 18 min and flow rate was 400 μl/min. The mass spectrometer was operated in ESI-positive mode, full scan 100–1,000 m/z, capillary temperature 300°C, sheath gas: 35, aux gas: 2, resolution 30,000, and capillary voltage 3 kV.

Metabolite extraction and LC–MS analysis

Cells were collected directly in ice-cold methanol in a 2 ml safe-lock Eppendorf vial (end concentration 80%). To the 400 μl volume, two spoons (~ 100 μl) of 0.9-2 mm steel beads were added and the samples were homogenized using a bullet blender (Next Advance, NY) for 2 min in a cold room (4°C). After homogenization, the samples were extracted by adding 240 μl ice-cold Milli-Q and 320 μl ice-cold chloroform. After vortex mixing for 30 s, the samples were left for 10 min at room temperature. Phases were separated after centrifugation (10 min, 17,000 *g* at 10°C) and the aqueous phase was transferred to a clean 1.5 ml Eppendorf vial and evaporated to dryness in a vacuum concentrator (Labconco, MO, USA). The residue was dissolved in 128 μl methanol–water (50/50, v/v) and transferred to an injection vial for analysis. Analysis was performed on a system consisting of a Waters M-Class LC system and a Waters Vion Quadrupole–Time-of-Flight mass spectrometer (Milford MA, USA). The analytical column was a SeQuant ZIC cHILIC, 150 × 1 mm, 3 μM (Merck KGaA, Darmstadt, Germany), and was kept at 30°C during analysis. Eluent A was 90% acetonitrile containing 5 mM ammonium acetate, pH 6.8, and eluent B consisted of 10% acetonitrile also containing 5 mM ammonium acetate, pH 6.8. The LC gradient used for separation commenced by the injection of 1 μl sample starting at 0% B for 3 min, followed by a 17 min linear increase to 64% B and kept at 64% B for 8 min before returning to 100% A in 0.5 min. The column was allowed to re-equilibrate for 12 min prior to a next analysis. Total runtime was 40.5 min and flow rate was 70 μl/min. The mass spectrometer was operated in negative mode, full-scan range between 70 and 1,000 m/z, source and desolvation temperatures were 120 and 350°C, cone and desolvation gas flow were 50 and 600 l/h, respectively, and capillary voltage −2 kV; ion source ESI.

LC–MS ^{13}C-glutamine tracing

HaCaT cells were transfected with control scrambled miRNA or miR-31. After 32 h, culture medium was replaced with 0.5% FBS DMEM with 1 mM glucose, without glutamine for 16 h. To start glutamine labeling, the medium was replaced with DMEM, which contains 0.5% FBS, 1 mM glucose, and 2 mM [U-$^{13}C_5$]-glutamine. Samples were harvested at the indicated time points after start of glutamine labeling. Medium was collected and snap frozen and cell was immediately harvested in 100% ice-cold methanol.

Analysis was performed as described (Ciapaite *et al*, 2020), with following modifications. Prior to analysis, calibration samples were prepared by dilution and addition of internal standards. Calibration samples were prepared in the concentration range from 0.08 to 80 μM. Twenty microliter of internal standard mix (100 μM) was added to e500 μl methanol sample extract or 50 μl standard and evaporated under a gentle flow of nitrogen at 40°C. To the dried extract, 25 μl 0.1% NaOH and 25 μl 10 mg/ml O-(2,3,4,5,6-pentafluorobenzyl) hydroxylamine (PFBHA) in Milli-Q water were added, vortexed for homogenization, and for derivatization incubated for 30 min at 20°C. For determination of extracellular metabolites, to 50 μl cell medium, 20 μl of Internal standard mix (100 μM), 25 μl 0.1% NaOH, and 25 μl 10 mg/ml PFBHA in Milli-Q water were added, vortexed, and derivatized 30 min at 20°C.

The derivatized metabolites were separated on a Sunshell RP-Aqua column (150 mm × 3 mm i.d., 2.6 μm; ChromaNik Technologies Inc., Osaka, Japan). The following eluents were used: solvent A: 0.1% formic acid in H_2O (v/v); and solvent B: 0.1% formic acid in ACN (v/v). Gradient elution was as follows: 0–2.75 min isocratic 0% B, 2.75–3.5 min linear from 0 to 70% B, 3.5–6.0 min isocratic 70% B, 6.0–6.2 min linear from 70 to 100% B, 6.2–9.0 min isocratic 100% B, and 9.0–9.2 min linear from 100 to 0% B, with 9.2–12 min for initial conditions of 0% B for column equilibration. Settings used were as follows: flow rate: 0.6 ml/min; column temperature: 40°C; autosampler temperature: 15°C; and injection volume: 5 μl.

The eluent was analyzed on a Q-Exactive HF mass spectrometer with the following settings: detection mode: full scan in negative ionization mode; scan range: 70–900 m/z; resolution: 240,000; AGC target: 1e6; maximum IT: 200 ms; capillary voltage: 4 kV; capillary temperature: 300°C; sheath gas: 50; aux gas: 2; spare gas: 0; S-lens RF level: 65; and ion source: ESI.

Analysis of cytokine production by multiplexed particle-based flow cytometric assay (Luminex)

Cell culture supernatants were collected, stored at −80°C, and processed as described (Scholman *et al*, 2018). Cytokine concentrations were measured with the Bio-Plex system in combination with the Bio-Plex Manager software, version 4.0 (Bio-Rad Laboratories, Hercules, CA, USA), which employs the Luminex xMAP technology (Scholman *et al*, 2018).

Induction of *in vitro* Th17 differentiation

Spleens were harvested from C57BL/6 mice for lymphocyte isolation following the protocol of Bedoya *et al* (2013). Naïve CD4$^+$ T cells were subsequently isolated by negative selection using magnetic beads (Miltenyi Biotech, 130-104-453). Naïve CD4$^+$ T cells were

The paper explained

Problem
Psoriasis is a chronic immune-mediated disorder of the skin that affects 0.1–0.4% of the total world population, and for which current treatment is still not optimal. Several skin diseases, including psoriasis, are characterized by a combination of keratinocyte hyperproliferation, consequent aberrant keratinocyte differentiation, and immune cell activation. These diseases thus require metabolic adaptations that enable co-existence between hyperproliferative keratinocytes and activated immune cells.

Results
Here, we found that miR-31 expression in the spiny layer of psoriatic skin induced a metabolic shift toward glutamine utilization, leading to massive production of aspartate. Aspartate in turn became a key mediator of metabolic crosstalk between keratinocytes and naïve $CD4^+$ T cells, promoting their differentiation toward Th17 cells. Importantly, targeting miR-31-induced metabolic changes and blocking intercellular metabolic crosstalk could alleviate psoriatic pathology.

Impact
Collectively, our data illustrate an emerging concept of metabolic interaction across cell compartments that characterizes disease development. This can be exploited to design effective treatment options for disease, as shown here for psoriasis.

stimulated with plate-bound anti-CD3 (eBioscience, 4310627) and 1.5 μg/ml soluble CD28 monoclonal antibodies (BD Biosciences Pharmingen, 557393) in DMEM medium and cytokines (IL-6, 20 ng/ml; TGF-b1, 5 ng/ml; IL-21, 25 ng/ml) or metabolites (glutamate 25 μM; aspartate 25 μM) for a period of 3 days, at which point cells were collected and were stained with the fluorescent-coupled antibodies (see Table EV6 for the information of antibodies).

Enzyme-linked immunosorbent assay

Serum samples from mice were collected and stored in −80°C freezer. Prior to the start of the experiment, serum samples were thawed and then assayed for IL-17A (RayBiotech, ELM-IL17-1,) and IL-10 (Cat: ELM-IL10-1, RayBiotech), respectively, according to the product instructions for the ELISA.

Data availability

Proteomics data are deposited at the PRIDE database with project accession PXD029030 (http://www.ebi.ac.uk/pride/archive/projects/PXD029030). Metabolomics data at MetaboLights (https://www.ebi.ac.uk/metabolights/index) with identifier MTBLS3608. Source microscopy images (Figs 5B and D, EV2B, EV4B, EV5E, and H) were deposited at Biostudies with accession number S-BSST999 (https://www.ebi.ac.uk/biostudies/studies/S-BSST999).

Expanded View for this article is available online.

Acknowledgements
We wish to thank Prof Matthew vander Heiden (MIT, Boston), Dr. Emmerik Leijten (Rheumatology, UMCU), Dr. Prof Nanda Verhoeven-Duif, Dr. Judith Jans (Metabolic Genetics, UMCU), Maria Rodriguez-Colman, Yvonne Vercoulen, Fried Zwartkruis, and other members of the Burgering Lab for comments, editing the manuscript, and discussion. Research in the Burgering Lab is financially supported by the Oncode Institute. Research at the Second Affiliated Hospital, Guangzhou University of Chinese Medicine, is supported by a grant from the National Natural Science Foundation of China (No. 81873302), a grant from the 2020 Guangdong Provincial Science and Technology Innovation Strategy Special Fund (No. 2020B1212030006), and four grants from Guangdong Provincial Hospital of Chinese Medicine (YN2019HL01, YN2019HL02, YN2019HL03, and YN2019QJ09).

Author contributions
Mao-Jie Wang: Conceptualization; data curation; software; formal analysis; validation; writing – original draft; project administration; writing – review and editing. **Huan-Jie Huang:** Data curation; formal analysis; validation; investigation; writing – review and editing. **Yong-Yue Xu:** Data curation; software; formal analysis; methodology. **Harmjan Vos:** Software; formal analysis; investigation; methodology. **Can Gulersonmez:** Formal analysis; investigation; methodology. **Edwin Stigter:** Formal analysis; investigation; methodology. **Johan Gerritsen:** Formal analysis. **Marc Pages Gallego:** Investigation; methodology. **Robert van Es:** Formal analysis; investigation; methodology. **Li Li:** Software; formal analysis. **Hao Deng:** Software; investigation. **Run-Yue Huang:** Conceptualization; supervision; writing – original draft. **Chuan-Jian Lu:** Conceptualization; supervision; writing – original draft; writing – review and editing. **Boudewijn MT Burgering:** Conceptualization; supervision; validation; investigation; writing – original draft; writing – review and editing.

Disclosure and competing interests statement
The authors declare that they have no conflict of interest.

References

Altman BJ, Stine ZE, Dang CV (2016) From Krebs to clinic: glutamine metabolism to cancer therapy. *Nat Rev Cancer* 16: 619 – 634

Armstrong AW, Read C (2020) Pathophysiology, clinical presentation, and treatment of psoriasis: a review. *JAMA* 323: 1945 – 1960

Bedoya SK, Wilson TD, Collins EL, Lau K, Larkin J 3rd (2013) Isolation and th17 differentiation of naive CD4 T lymphocytes. *J Vis Exp* https://doi.org/10.3791/50765

van den Berg WB, McInnes IB (2013) Th17 cells and IL-17 A—focus on immunopathogenesis and immunotherapeutics. *Semin Arthritis Rheum* 43: 158 – 170

Cheng T, Sudderth J, Yang C, Mullen AR, Jin ES, Mates JM, DeBerardinis RJ (2011) Pyruvate carboxylase is required for glutamine-independent growth of tumor cells. *Proc Natl Acad Sci USA* 108: 8674 – 8679

Ciapaite J, Albersen M, Savelberg SMC, Bosma M, Tessadori F, Gerrits J, Lansu N, Zwakenberg S, Bakkers JPW, Zwartkruis FJT *et al* (2020) Pyridox(am)ine 5′-phosphate oxidase (PNPO) deficiency in zebrafish results in fatal seizures and metabolic aberrations. *Biochim Biophys Acta Mol Basis Dis* 1866: 165607

Coloff JL, Murphy JP, Braun CR, Harris IS, Shelton LM, Kami K, Gygi SP, Selfors LM, Brugge JS (2016) Differential glutamate metabolism in proliferating and quiescent mammary epithelial cells. *Cell Metab* 23: 867 – 880

Dixon SJ, Lemberg KM, Lamprecht MR, Skouta R, Zaitsev EM, Gleason CE, Patel DN, Bauer AJ, Cantley AM, Yang WS *et al* (2012) Ferroptosis: an iron-dependent form of nonapoptotic cell death. *Cell* 149: 1060 – 1072

van der Fits L, Mourits S, Voerman JS, Kant M, Boon L, Laman JD, Cornelissen F, Mus AM, Florencia E, Prens EP *et al* (2009) Imiquimod-induced

 © 2023 The Authors

psoriasis-like skin inflammation in mice is mediated via the IL-23/IL-17 axis. *J Immunol* 182: 5836 – 5845

Friis NU, Hoffmann N, Gyldenlove M, Skov L, Vilsboll T, Knop FK, Storgaard H (2019) Glucose metabolism in patients with psoriasis. *Br J Dermatol* 180: 264 – 271

Garcia-Bermudez J, Baudrier L, La K, Zhu XG, Fidelin J, Sviderskiy VO, Papagiannakopoulos T, Molina H, Snuderl M, Lewis CA *et al* (2018) Aspartate is a limiting metabolite for cancer cell proliferation under hypoxia and in tumours. *Nat Cell Biol* 20: 775 – 781

Greb JE, Goldminz AM, Elder JT, Lebwohl MG, Gladman DD, Wu JJ, Mehta NN, Finlay AY, Gottlieb AB (2016) Psoriasis. *Nat Rev Dis Primers* 2: 16082

Guo K, Zhang X (2021) Cytokines that modulate the differentiation of Th17 cells in autoimmune uveitis. *J Immunol Res* 19: 6693542

Hsieh JC, Estess RC, Kaneko I, Whitfield GK, Jurutka PW, Haussler MR (2014) Vitamin D receptor-mediated control of soggy, wise, and hairless gene expression in keratinocytes. *J Endocrinol* 220: 165 – 178

Ishihara H, Asano T, Tsukuda K, Katagiri H, Inukai K, Anai M, Kikuchi M, Yazaki Y, Miyazaki J, Oka Y (1994) Overexpression of hexokinase I but not GLUT1 glucose transporter alters concentration dependence of glucose-stimulated insulin secretion in pancreatic beta-cell line MIN6. *J Biol Chem* 269: 3081 – 3087

Johnson MO, Wolf MM, Madden MZ, Andrejeva G, Sugiura A, Contreras DC, Maseda D, Liberti MV, Paz K, Kishton RJ *et al* (2018) Distinct regulation of Th17 and Th1 cell differentiation by Glutaminase-dependent metabolism. *Cell* 175: 1780 – 1795.e19

Joyce CE, Zhou X, Xia J, Ryan C, Thrash B, Menter A, Zhang W, Bowcock AM (2011) Deep sequencing of small RNAs from human skin reveals major alterations in the psoriasis miRNAome. *Hum Mol Genet* 20: 4025 – 4040

Kono M, Yoshida N, Maeda K, Tsokos GC (2018) Transcriptional factor ICER promotes glutaminolysis and the generation of Th17 cells. *Proc Natl Acad Sci USA* 115: 2478 – 2483

Kornberg MD, Bhargava P, Kim PM, Putluri V, Snowman AM, Putluri N, Calabresi PA, Snyder SH (2018) Dimethyl fumarate targets GAPDH and aerobic glycolysis to modulate immunity. *Science* 360: 449 – 453

Kuehl B, Shear NH (2018) The evolution of topical formulations in psoriasis. *Skin Therapy Lett* 23: 5 – 9

Laurila EM, Kallioniemi A (2013) The diverse role of miR-31 in regulating cancer associated phenotypes. *Genes Chromosomes Cancer* 52: 1103 – 1113

Lemons JM, Feng XJ, Bennett BD, Legesse-Miller A, Johnson EL, Raitman I, Pollina EA, Rabitz HA, Rabinowitz JD, Coller HA (2010) Quiescent fibroblasts exhibit high metabolic activity. *PLoS Biol* 8: e1000514

Lowes MA, Suarez-Farinas M, Krueger JG (2014) Immunology of psoriasis. *Annu Rev Immunol* 32: 227 – 255

Miao Y, Zheng Y, Geng Y, Yang L, Cao N, Dai Y, Wei Z (2021) The role of GLS1-mediated glutaminolysis/2-HG/H3K4me3 and GSH/ROS signals in Th17 responses counteracted by PPARgamma agonists. *Theranostics* 11: 4531 – 4548

Mrowietz U, Barker J, Boehncke WH, Iversen L, Kirby B, Naldi L, Reich K, Tanew A, van de Kerkhof PCM, Warren RB (2018) Clinical use of dimethyl fumarate in moderate-to-severe plaque-type psoriasis: a European expert consensus. *J Eur Acad Dermatol Venereol* 32: 3 – 14

Parisi R, Iskandar IYK, Kontopantelis E, Augustin M, Griffiths CEM, Ashcroft DM, Global Psoriasis Atlas (2020) National, regional, and worldwide epidemiology of psoriasis: systematic analysis and modelling study. *BMJ* 369: m1590

Pleguezuelos O, Kapas S (2006) Differentiation of the HaCaT keratinocyte cell line: modulation by adrenomedullin. *Br J Dermatol* 154: 602 – 608

Scholman RC, Giovannone B, Hiddingh S, Meerding JM, Malvar Fernandez B, van Dijk MEA, Tempelman MJ, Prakken BJ, de Jager W (2018) Effect of anticoagulants on 162 circulating immune related proteins in healthy subjects. *Cytokine* 106: 114 – 124

Valastyan S, Weinberg RA (2010) miR-31: a crucial overseer of tumor metastasis and other emerging roles. *Cell Cycle* 9: 2124 – 2129

Wang TS, Tsai TF (2017) Managing scalp psoriasis: an evidence-based review. *Am J Clin Dermatol* 18: 17 – 43

Wang WH, Song SJ, Li LF, Zhang L, Yang SM, Zhang Q, Wang YY, Sun TT (2010) Disturbed keratin expression and distinct genotype of ichthyosis hystrix Lambert type. *Eur J Dermatol* 20: 567 – 572

Wang Q, Chang W, Yang X, Cheng Y, Zhao X, Zhou L, Li J, Li J, Zhang K (2019) Levels of miR-31 and its target genes in dermal mesenchymal cells of patients with psoriasis. *Int J Dermatol* 58: 198 – 204

Xia X, Cao G, Sun G, Zhu L, Tian Y, Song Y, Guo C, Wang X, Zhong J, Zhou W *et al* (2020) GLS1-mediated glutaminolysis unbridled by MALT1 protease promotes psoriasis pathogenesis. *J Clin Invest* 130: 5180 – 5196

Xu N, Meisgen F, Butler LM, Han G, Wang XJ, Soderberg-Naucler C, Stahle M, Pivarcsi A, Sonkoly E (2013) MicroRNA-31 is overexpressed in psoriasis and modulates inflammatory cytokine and chemokine production in keratinocytes via targeting serine/threonine kinase 40. *J Immunol* 190: 678 – 688

Xu T, Stewart KM, Wang X, Liu K, Xie M, Ryu JK, Li K, Ma T, Wang H, Ni L *et al* (2017) Metabolic control of TH17 and induced Treg cell balance by an epigenetic mechanism. *Nature* 548: 228 – 233

Yan S, Xu Z, Lou F, Zhang L, Ke F, Bai J, Liu Z, Liu J, Wang H, Zhu H *et al* (2015) NF-kappaB-induced microRNA-31 promotes epidermal hyperplasia by repressing protein phosphatase 6 in psoriasis. *Nat Commun* 6: 7652

Zhang Z, Zi Z, Lee EE, Zhao J, Contreras DC, South AP, Abel ED, Chong BF, Vandergriff T, Hosler GA *et al* (2018) Differential glucose requirement in skin homeostasis and injury identifies a therapeutic target for psoriasis. *Nat Med* 24: 617 – 627

Zhou L, Lopes JE, Chong MM, Ivanov II, Min R, Victora GD, Shen Y, Du J, Rubtsov YP, Rudensky AY *et al* (2008) TGF-beta-induced Foxp3 inhibits T (H)17 cell differentiation by antagonizing RORgammat function. *Nature* 453: 236 – 240

License: This is an open access article under the terms of the Creative Commons Attribution License, which permits use, distribution and reproduction in any medium, provided the original work is properly cited.

Expanded View Figures

Figure EV1. Identification of targets of miR-31 by stable isotope labeling by amino acids in cell culture proteomics.

A Quantitative RT–PCR detection of Has-miR-31-5p expression levels in skin biopsies of healthy individuals and psoriasis patients (n = 5 individuals), left panel; and mmu-miR-31-5p expression levels in skin biopsies of healthy mice or imiquimod-induced psoriatic mice (IMQ), n = 7 mice in each group, right panel.

B Violin plot of miR-31 host gene (miR-31 HG) expression in human skin biopsies. Data were extracted from an online microarray study (GSE13355). n = 64/58 in healthy control (HC)/psoriasis patients (PN, psoriatic non-lesional skin; PL, psoriatic lesional skin), respectively.

C Schematic representation of the workflow for SILAC proteomics.

D, E Volcano plots summarizing SILAC proteomics data in HEK 293 T cells (D) and HaCaT cells (E).

F Correlation analysis of SILAC proteomics data of HEK 293 T cells and HaCaT cells.

G, H Volcano plot representation of changes in validated targets of miR-31 in HEK 293 T cells (G) and HaCaT cells (H) proteomics.

I Principal component analysis (PCA) indicating a specific metabolism signature induced by miR-31 overexpression in HEK 293 T cells.

Data information: In (A) and (B), data are presented as truncated violin plot, and dash and solid lines represent quartiles and median, respectively (by unpaired Student's t-test for (A) and one-way ANOVA with Sidak test for (B)).
Source data are available online for this figure.

 © 2023 The Authors

18 of 17 EMBO Molecular Medicine 15: e15674 | 2023 © 2023 The Authors

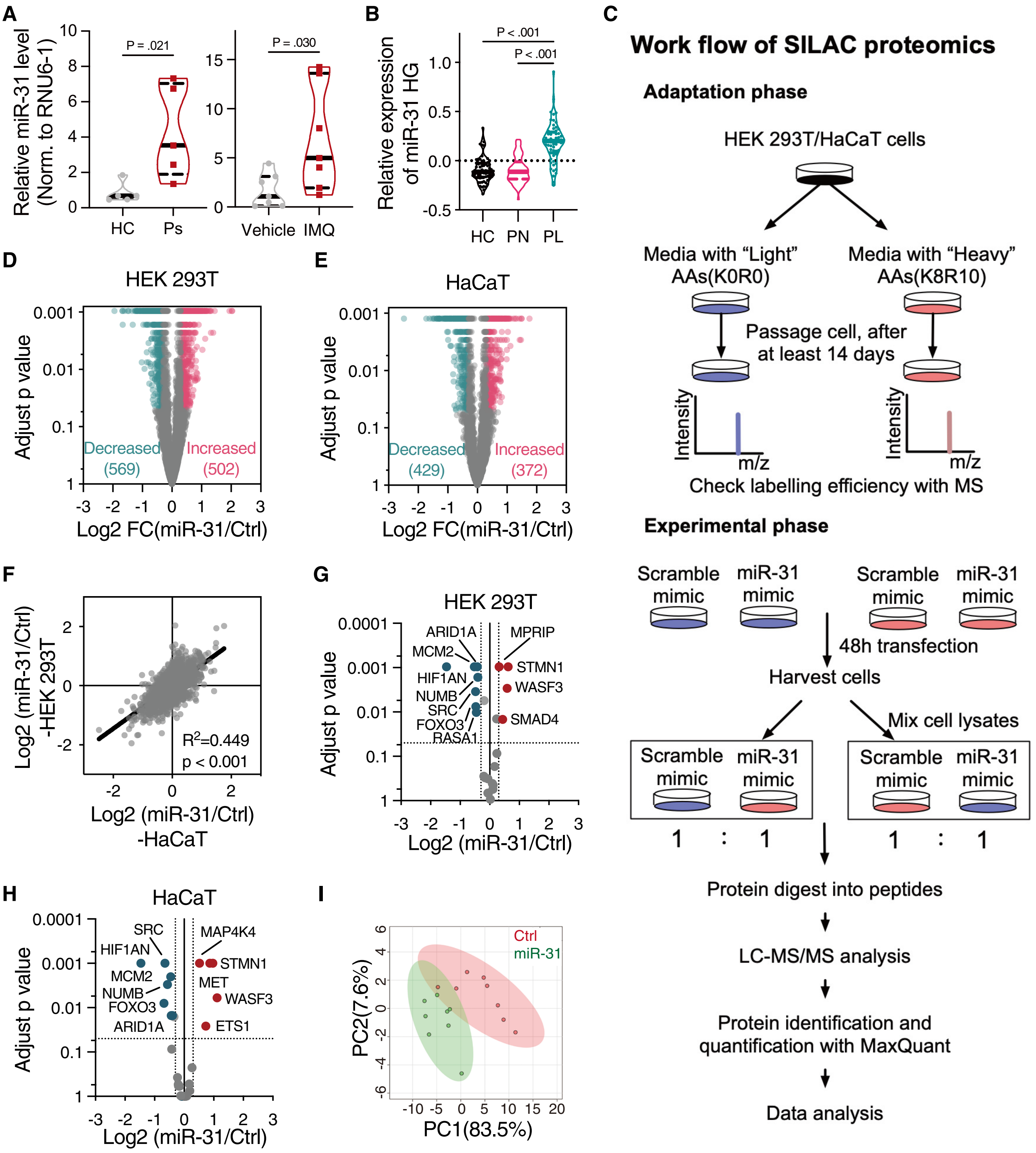
A
Relative miR-31 level (Norm. to RNU6-1)
P = .021
P = .030
HC Ps
Vehicle IMQ
B
Relative expression of miR-31 HG
P < .001
P < .001
HC PN PL
C
Work flow of SILAC proteomics
Adaptation phase
HEK 293T/HaCaT cells
Media with "Light" AAs(K0R0)
Media with "Heavy" AAs(K8R10)
Passage cell, after at least 14 days
Intensity
m/z
Check labelling efficiency with MS
Experimental phase
Scramble mimic
miR-31 mimic
48h transfection
Harvest cells
Mix cell lysates
1 : 1
Protein digest into peptides
LC-MS/MS analysis
Protein identification and quantification with MaxQuant
Data analysis
D
HEK 293T
Adjust p value
Decreased (569)
Increased (502)
Log2 FC(miR-31/Ctrl)
E
HaCaT
Decreased (429)
Increased (372)
F
Log2 (miR-31/Ctrl) -HEK 293T
R2=0.449
p < 0.001
Log2 (miR-31/Ctrl) -HaCaT
G
HEK 293T
ARID1A
MCM2
HIF1AN
NUMB
SRC
FOXO3
RASA1
MPRIP
STMN1
WASF3
SMAD4
Log2 (miR-31/Ctrl)
H
HaCaT
SRC
HIF1AN
MCM2
NUMB
FOXO3
ARID1A
MAP4K4
STMN1
MET
WASF3
ETS1
I
Ctrl
miR-31
PC2(7.6%)
PC1(83.5%)

Figure EV1.

Figure EV2. MiR-31 inhibits glycolysis and rewires glutamine metabolism.

A 2-NBDG uptake of HaCaT cells in response to different doses of glucose (n = 3 biological replicates).
B Immunostaining of GLUT1 in HaCaT cells of 48 h treatments of scramble, miR-31 mimic, and GLUT1 siRNA (si-GLUT1). DAPI is gray and GLUT1 is green. Similar results were obtained from three independent experiments. Scale bars: 20 μm.
C Quantitative RT–PCR detection of GLUT1 expression levels in HaCaT cells (n = 3 biological replicates) and normal human epidermal keratinocytes (NHEK, n = 3 biological replicates) treated with or without 2 mM $CaCl_2$ upon miR-31 overexpression.
D LDH activity assay was applied in HaCaT cells treated with 0 mM or 2 mM CaCl2 and HEK 293 T cells upon miR-31 overexpression and inhibition (n = 4 biological replicates).
E Quantitative RT–PCR detection of PDHX expression levels in HaCaT cells (n = 3 biological replicates) and NHEK (n = 3 biological replicates) treated with or without 2 mM $CaCl_2$ upon miR-31 overexpression.
F Western blot analysis of PDHX and GS in HaCaT and NHEK cells treated with or without 2 mM $CaCl_2$ upon miR-31 overexpression, and vinculin was used as loading control.
G Quantitative RT–PCR detection of SLC7A11 and SLC3A2 in HaCaT cells upon miR-31 overexpression (n = 7 biological replicates).
H Baselined extracellular acidification rates (ECAR) and oxygen consumption rates (OCR) of HaCaT cells in response to glutamine (Gln) or glutamate (Glu) addition in a long-term observation (n = 5 biological replicates).
I A summary scheme showing how miR-31 rewires glutamine metabolism.
J, K FACS analysis of 2-NBDG uptake of HaCaT cells upon treatments of GS siRNA (si-GS) and CB-839 (C, n = 4 biological replicates) or PC siRNA (si-PC, D, n = 3 biological replicates).
L, M Glycolysis stress test (L) and mitochondrial stress test (M) of HaCaT cells upon GS siRNA treatment in 1 mM glucose condition (n = 5 biological replicates). ECAR and OCR were normalized to the amount of protein.

Data information: In (A), (C–E), (H), and (J–M), data are presented as mean ± SD (by One-way ANOVA with Sidak test for (J), unpaired Student's t-test for (K), and two-way ANOVA with Sidak test for (A), (C–E), (G), (L), and (M)).
Source data are available online for this figure.

© 2023 The Authors

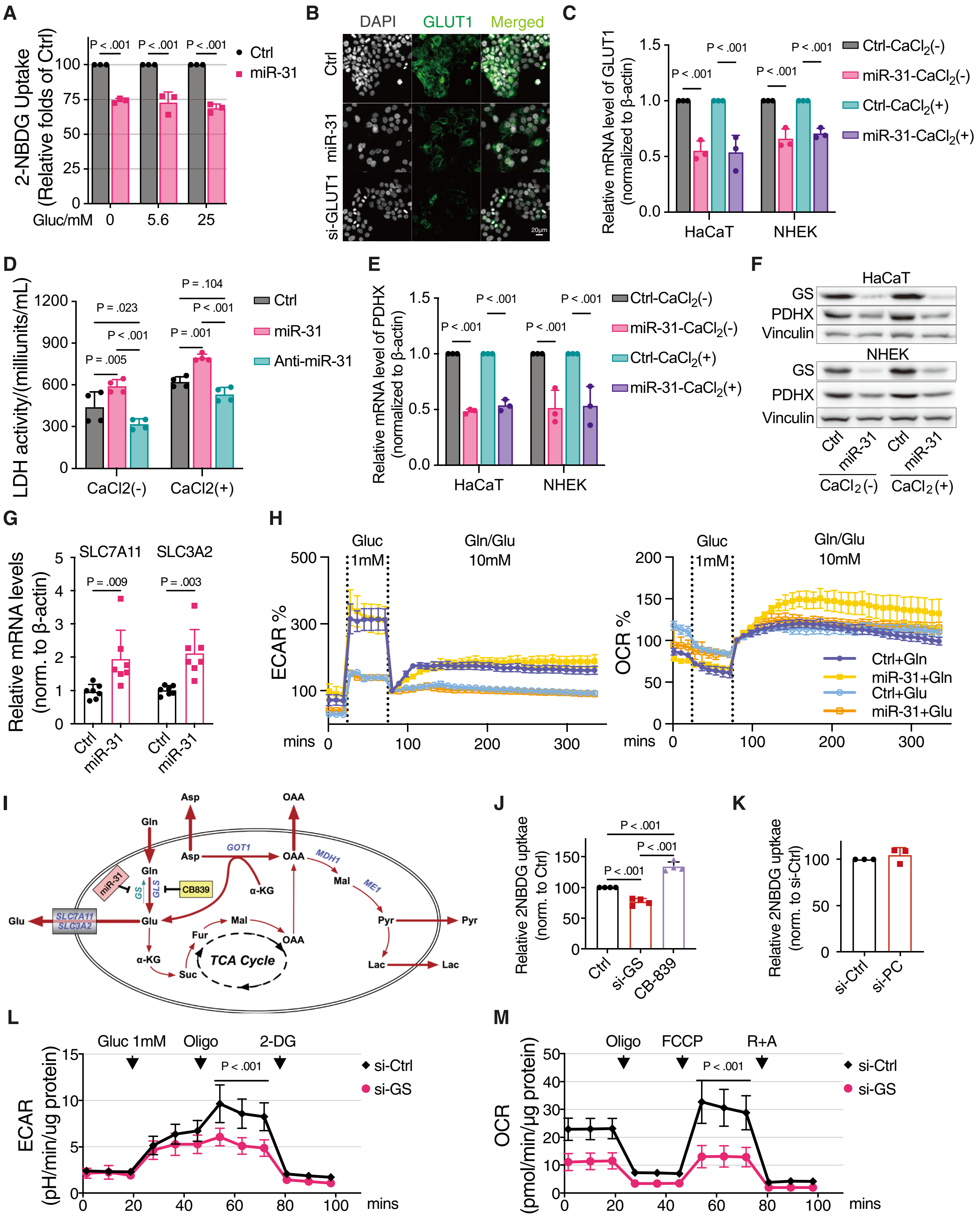
A
2-NBDG Uptake (Relative folds of Ctrl)
Ctrl
miR-31
Gluc/mM 0 5.6 25
B
DAPI GLUT1 Merged
Ctrl
miR-31
si-GLUT1
C
Relative mRNA level of GLUT1 (normalized to β-actin)
Ctrl-CaCl2(-)
miR-31-CaCl2(-)
Ctrl-CaCl2(+)
miR-31-CaCl2(+)
HaCaT NHEK
D
LDH activity/(milliunits/mL)
Ctrl
miR-31
Anti-miR-31
CaCl2(-) CaCl2(+)
E
Relative mRNA level of PDHX (normalized to β-actin)
F
HaCaT
GS
PDHX
Vinculin
NHEK
G
SLC7A11 SLC3A2
Relative mRNA levels (norm. to β-actin)
H
Gluc 1mM
Gln/Glu 10mM
ECAR %
OCR %
Ctrl+Gln
miR-31+Gln
Ctrl+Glu
miR-31+Glu
mins
I
TCA Cycle
J
Relative 2NBDG uptkae (norm. to Ctrl)
Ctrl si-GS CB-839
K
Relative 2NBDG uptkae (norm. to si-Ctrl)
si-Ctrl si-PC
L
Gluc 1mM Oligo 2-DG
ECAR (pH/min/ug protein)
si-Ctrl
si-GS
M
Oligo FCCP R+A
OCR (pmol/min/μg protein)

Figure EV2.

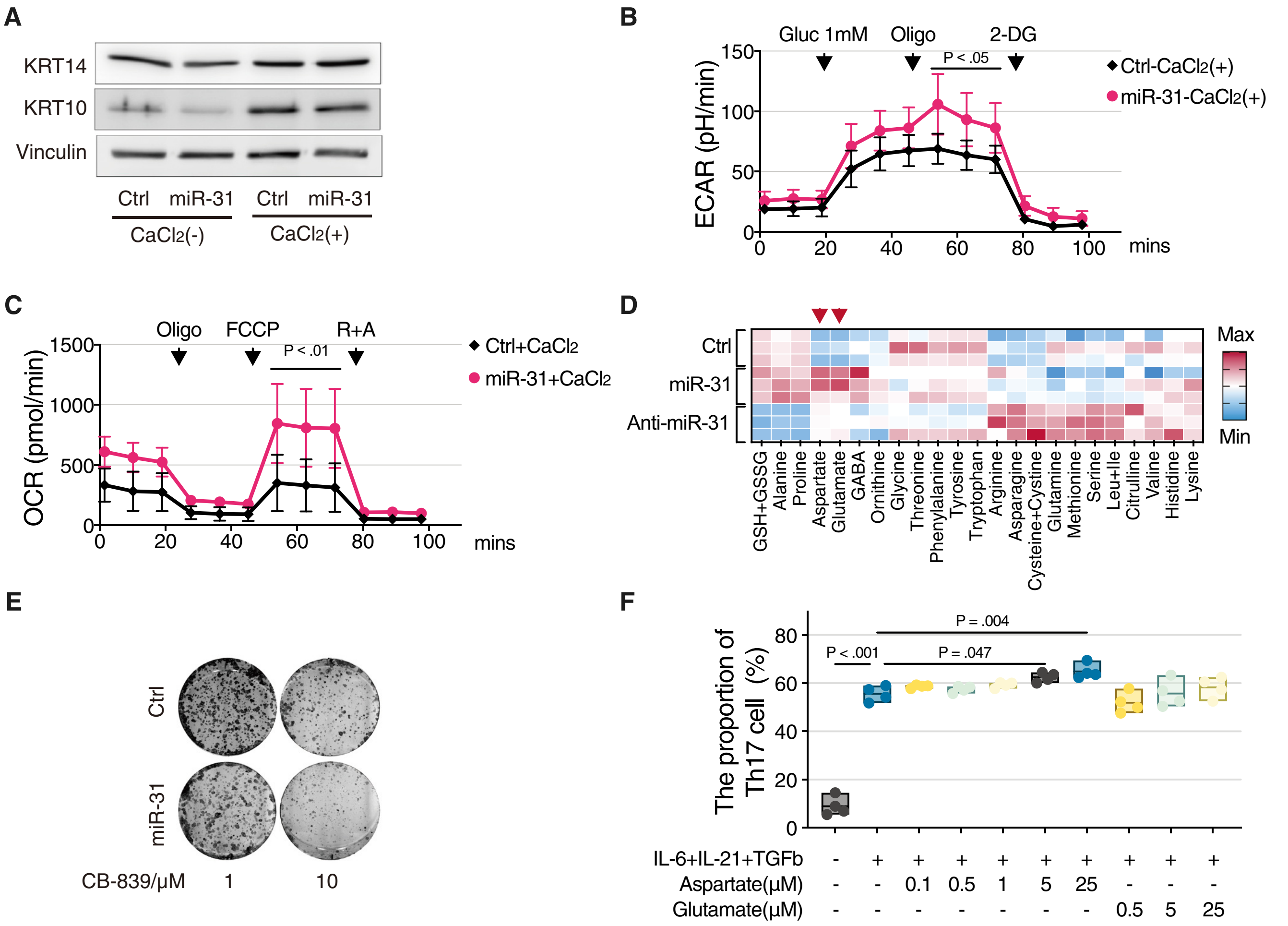

Figure EV3. Differentiated HaCaT cells show similar metabolism reprogramming upon miR-31 overexpression.

A Western blot analysis of KRT14 and KRT10 expressions in HaCaT cells cultured in medium supplemented with or without 2 mM CaCl2, and vinculin was used as loading control.

B Glycolysis stress test of differentiated HaCaT cells upon miR-31 overexpression in 1 mM glucose condition ($n = 5$ biological replicates).

C Mitochondrial stress test of differentiated HaCaT cells upon miR-31 overexpression in 1 mM glucose condition ($n = 3$ biological replicates).

D Heatmap representation of relative amino acid levels in cell culture medium of differentiated HaCaT cells upon miR-31 overexpression.

E Representative picture of cell colony-forming assay of HaCaT cells in response to CB839 treatment ($n = 3$ biological replicates).

F FACS analysis of Th17 differentiation upon glutamate or aspartate additions ($n = 4$ biological replicates).

Data information: In (B), (C), and (F), data are presented as mean ± SD (by two-way ANOVA with Sidak test for (B) and (C) and one-way ANOVA with Sidak test for (F)). Source data are available online for this figure.

 © 2023 The Authors

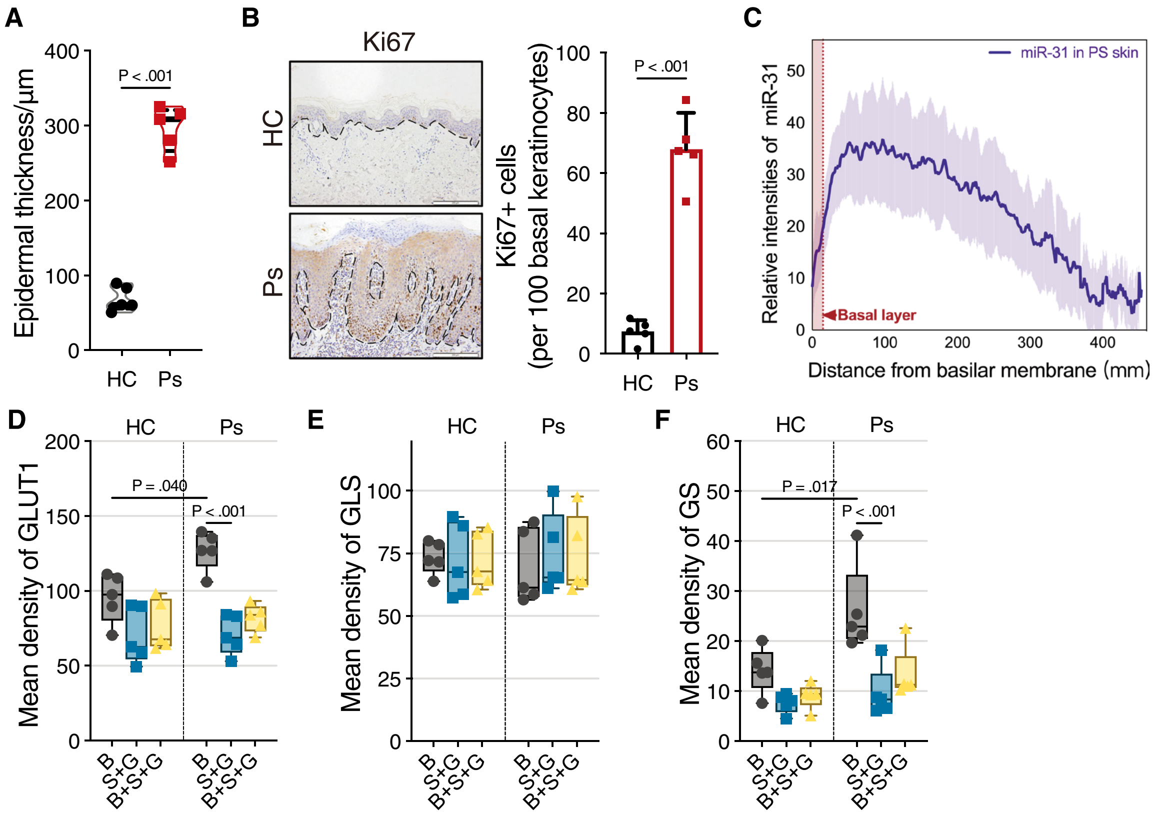

Figure EV4. Epidermal keratinocytes exhibit different metabolic characteristics *in vivo*.

- A Violin plot of epidermal thickness of skin biopsies taken from healthy individuals and psoriasis patients ($n = 5$ individuals in each group).
- B Representative pictures of immunohistochemical staining of Ki-67 in skin biopsies of HC and Ps. Scale bars: 200 μm. Quantification of result is shown on right panel, $n = 5$ biological replicates.
- C Quantification of result of miR-31 ISH data of skin biopsies from psoriasis patients ($n = 5$ biological replicates). The continuous line represents the mean intensity of miR-31, and the shaded area means the standard deviation.
- D–F Quantification of results of GLUT1(D), GLS(E), and GS(F) staining of human skin biopsies ($n = 5$ biological replicates). S + G layers: spinous layer and granular layer of epidermis; B + S + G layers: basal layer, spinous layer, and granular layer.

Data information: Data are presented as truncated violin plot in (A), mean ± SD in (B) and (C), and box and whiskers (central band, median; whiskers, min to max and show all points) in (D–F) (by unpaired Student's *t*-test for (A) and (B), One-way ANOVA with Sidak test for (D–F)).
Source data are available online for this figure.

Figure EV5. Blocking the miR-31 induced metabolic changes relieves psoriatic disease in a mouse model.

- A Representations of *in situ* hybridization staining (ISH) of miR-31 in skin biopsies from control mice (Ctrl, $n = 3$ mice) and imiquimod-induced psoriatic mice (IMQ, $n = 6$ mice). Quantification result showing the average level of miR-31 in epidermis (right panel). B, basal layer; S + G, spinous and granular layers. Scale bars: 100 μm.
- B–D Disease activity was evaluated by epidermal thickness (B), Baker's score (C), and Ki-67-positive cell count (D). $n = 4$ mice in Ctrl and CBG groups and $n = 5$ mice in IMQ and CB-839 groups.
- E–G Representative pictures of immunohistochemistry staining of CD4 (upper panel) and MPO (bottom panel) in skin biopsies from mice ($n = 4$ mice in Ctrl and CBG groups and $n = 5$ mice in IMQ and CB-839 groups). Scale bars: 100 μm. Quantification results showing the average number of CD4-positive or MPO-positive cells in skin sections (F and G, respectively).
- H–K IHC staining of GLUT1, GLS, and GS in skin from mice with different treatments (H, scale bars: 25 μm). I–K present the quantification results of GLUT1, GLS, and GS, respectively. $n = 4$ mice in Ctrl and CBG groups and $n = 5$ mice in IMQ and CB-839 groups.
- L Enzyme-linked immunosorbent assay for the determination of IL-10 levels in serum of mice ($n = 5$ mice in vehicle group and $n = 6$ mice in other groups).

Data information: In (A), (F), and (G), data are presented as mean ± SD. In (B–D) and (I–L), data are presented as box and whiskers (min to max; by unpaired Student's *t*-test for (A) and one-way ANOVA with Sidak test for (B–D) and (I–L)).
Source data are available online for this figure.

© 2023 The Authors

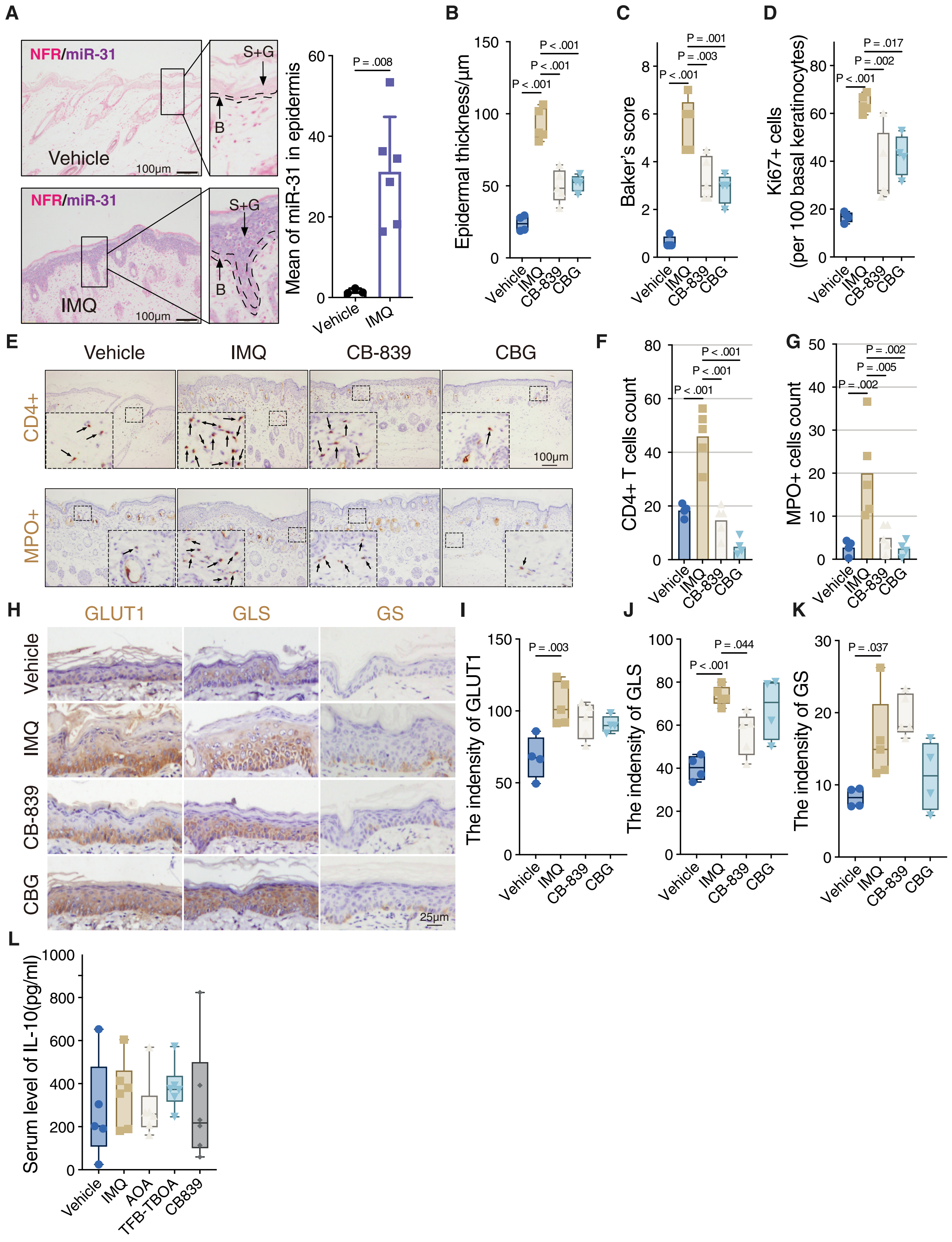

Figure EV5.

© 2023 The Authors
