# Supplementary material for: Metabolic rewiring in keratinocytes by miR‐31‐5p identifies therapeutic intervention for psoriasis
Source: EMBO Mol Med. 2023 Mar 1;15(4):e15674. doi: 10.15252/emmm.202215674 (PMC10086589; doi:10.15252/emmm.202215674)
Supplement: Supplementary file 16 [file EMMM-15-e15674-s005.pdf]

EMBO Molecular Medicine

Metabolic rewiring in keratinocytes by miR-31-5p identifies therapeutic intervention for psoriasis

Maojie Wang, Huanjie Huang, Yue Yong, Harmjan Vos, M. Can Gulersonmez, Edwin Stigter, Johan Gerrits, Marc Pages Gallego, Robert van Es, Li Li, Hao Deng, Ling Han, Runyue Huang, Chuanjian Lu, and Boudewijn Burgering
DOI: 10.15252/emmm.202215674

Corresponding author(s): Boudewijn Burgering (b.m.t.burgering@umcutrecht.nl) , Chuanjian Lu (lcj@gzucm.edu.cn), Runyue Huang (ryhuang@gzucm.edu.cn)

Review Timeline:

Submission Date:	21st Jan 22
Editorial Decision:	15th Feb 22
Revision Received:	15th Sep 22
Editorial Decision:	4th Oct 22
Revision Received:	17th Jan 23
Editorial Decision:	2nd Feb 23
Revision Received:	6th Feb 23
Accepted:	7th Feb 23

Editor: Lise Roth

Transaction Report:

(Note: With the exception of the correction of typographical or spelling errors that could be a source of ambiguity, letters and reports are not edited. Depending on transfer agreements, referee reports obtained elsewhere may or may not be included in this compilation. Referee reports are anonymous unless the Referee chooses to sign their reports.)

15th Feb 2022

Dear Prof. Burgering,

Thank you for the submission of your manuscript to EMBO Molecular Medicine. We have now heard back from the referees who agreed to evaluate your manuscript. As you will see below, the reviewers raise substantial concerns on your work, which unfortunately preclude its publication in EMBO Molecular Medicine in its current form.

The reviewers find that the question addressed by the study is of potential interest, however they remain unconvinced that some of the major conclusions are sufficiently supported by the data. They thus raise the following major issues:
● in vitro studies performed in proliferating keratinocytes instead of differentiated cells
● absence of solid experimental evidence to support the concept of competition for glucose between keratinocytes and immune cells in psoriasis.

Addressing the reviewers concerns in full (above points as well as other reviewers' comments) will be necessary for further considering the manuscript in our journal. As revising the manuscript according to the referees' recommendations appears to require a lot of additional work and experimentation, and given the potential interest of your findings, we are ready to extend the deadline to 6 months with the understanding that acceptance of the manuscript would entail a second round of review.

EMBO Molecular Medicine encourages a single round of revision only and therefore, acceptance or rejection of the manuscript will depend on the completeness of your responses included in the next, final version of the manuscript. For this reason, and to save you from any frustrations in the end, I would strongly advise against returning an incomplete revision. Should you find that the requested revisions are not feasible within the constraints outlined here and prefer, therefore, to submit your paper elsewhere, we would welcome a message to this effect.

When submitting your revised manuscript, please carefully review the instructions that follow below. We perform an initial quality control of all revised manuscripts before re-review; failure to include requested items will delay the evaluation of your revision.

We require:

1) A .docx formatted version of the manuscript text (including legends for main figures, EV figures and tables). Please make sure that the changes are highlighted to be clearly visible.

2) Individual production quality figure files as .eps, .tif, .jpg (one file per figure). For guidance, download the 'Figure Guide PDF' (https://www.embopress.org/page/journal/17574684/authorguide#figureformat).

3) A .docx formatted letter INCLUDING the reviewers' reports and your detailed point-by-point responses to their comments. As part of the EMBO Press transparent editorial process, the point-by-point response is part of the Review Process File (RPF), which will be published alongside your paper.

4) A complete author checklist, which you can download from our author guidelines (https://www.embopress.org/page/journal/17574684/authorguide#submissionofrevisions). Please insert information in the checklist that is also reflected in the manuscript. The completed author checklist will also be part of the RPF.

5) Please note that all corresponding authors are required to supply an ORCID ID for their name upon submission of a revised manuscript.

6) It is mandatory to include a 'Data Availability' section after the Materials and Methods. Before submitting your revision, primary datasets produced in this study need to be deposited in an appropriate public database, and the accession numbers and database listed under 'Data Availability'. Please remember to provide a reviewer password if the datasets are not yet public (see https://www.embopress.org/page/journal/17574684/authorguide#dataavailability).
In case you have no data that requires deposition in a public database, please state so in this section. Note that the Data Availability Section is restricted to new primary data that are part of this study.

7) For data quantification: please specify the name of the statistical test used to generate error bars and P values, the number (n) of independent experiments (specify technical or biological replicates) underlying each data point and the test used to calculate p-values in each figure legend. The figure legends should contain a basic description of n, P and the test applied. Graphs must include a description of the bars and the error bars (s.d., s.e.m.).

8) We would also encourage you to include the source data for figure panels that show essential data. Numerical data should be provided as individual .xls or .csv files (including a tab describing the data). For blots or microscopy, uncropped images should

be submitted (using a zip archive if multiple images need to be supplied for one panel). Additional information on source data and instruction on how to label the files are available at
.

9) Our journal encourages inclusion of *data citations in the reference list* to directly cite datasets that were re-used and obtained from public databases. Data citations in the article text are distinct from normal bibliographical citations and should directly link to the database records from which the data can be accessed. In the main text, data citations are formatted as follows: "Data ref: Smith et al, 2001" or "Data ref: NCBI Sequence Read Archive PRJNA342805, 2017". In the Reference list, data citations must be labeled with "[DATASET]". A data reference must provide the database name, accession number/identifiers and a resolvable link to the landing page from which the data can be accessed at the end of the reference. Further instructions are available at .

10) We replaced Supplementary Information with Expanded View (EV) Figures and Tables that are collapsible/expandable online. A maximum of 5 EV Figures can be typeset. EV Figures should be cited as 'Figure EV1, Figure EV2" etc... in the text and their respective legends should be included in the main text after the legends of regular figures.
- For the figures that you do NOT wish to display as Expanded View figures, they should be bundled together with their legends in a single PDF file called *Appendix*, which should start with a short Table of Content. Appendix figures should be referred to in the main text as: "Appendix Figure S1, Appendix Figure S2" etc.
- Additional Tables/Datasets should be labeled and referred to as Table EV1, Dataset EV1, etc. Legends have to be provided in a separate tab in case of .xls files. Alternatively, the legend can be supplied as a separate text file (README) and zipped together with the Table/Dataset file.
See detailed instructions here:
.

11) The paper explained: EMBO Molecular Medicine articles are accompanied by a summary of the articles to emphasize the major findings in the paper and their medical implications for the non-specialist reader. Please provide a draft summary of your article highlighting
- the medical issue you are addressing,
- the results obtained and
- their clinical impact.

This may be edited to ensure that readers understand the significance and context of the research. Please refer to any of our published articles for an example.

12) For more information: There is space at the end of each article to list relevant web links for further consultation by our readers. Could you identify some relevant ones and provide such information as well? Some examples are patient associations, relevant databases, OMIM/proteins/genes links, author's websites, etc...

13) Author contributions: the contribution of every author must be detailed in a separate section (before the acknowledgments).

14) Conflict of interest: We updated our journal's competing interests policy in January 2022 and request authors to consider both actual and perceived competing interests. Please review the policy https://www.embopress.org/competing-interests and update your competing interests if necessary.

15) Every published paper now includes a 'Synopsis' to further enhance discoverability. Synopses are displayed on the journal webpage and are freely accessible to all readers. They include a short stand first (maximum of 300 characters, including space) as well as 2-5 one-sentences bullet points that summarizes the paper. Please write the bullet points to summarize the key NEW findings. They should be designed to be complementary to the abstract - i.e. not repeat the same text. We encourage inclusion of key acronyms and quantitative information (maximum of 30 words / bullet point). Please use the passive voice. Please attach these in a separate file or send them by email, we will incorporate them accordingly.

Please also suggest a striking image or visual abstract to illustrate your article as a PNG file 550 px wide x 300-600 px high.

16) As part of the EMBO Publications transparent editorial process initiative (see our Editorial at http://embomolmed.embopress.org/content/2/9/329), EMBO Molecular Medicine will publish online a Review Process File (RPF) to accompany accepted manuscripts.
In the event of acceptance, this file will be published in conjunction with your paper and will include the anonymous referee reports, your point-by-point response and all pertinent correspondence relating to the manuscript. Let us know whether you agree with the publication of the RPF and as here, if you want to remove or not any figures from it prior to publication.
Please note that the Authors checklist will be published at the end of the RPF.

EMBO Molecular Medicine has a "scooping protection" policy, whereby similar findings that are published by others during review or revision are not a criterion for rejection. Should you decide to submit a revised version, I do ask that you get in touch

after three months if you have not completed it, to update us on the status.

I look forward to receiving your revised manuscript.

Yours sincerely,

Lise Roth

Lise Roth, PhD
Editor
EMBO Molecular Medicine

***** Reviewer's comments *****

Referee #1 (Comments on Novelty/Model System for Author):

This study uses a comination of elegant techniques, including metabolomics and SILEC, to demonstrate metabolic rewiring and crosstalk between keratinocytes and T cells. My main concerns is that most experimenst were performed in vitro in human keratinocyte cell line (HACAT) and the in vivo experiments (mouse model of psoriasis) are scarce and should provide more mechanistic information.

Referee #1 (Remarks for Author):

This study elegantly demostrates that miR-31 increases glutamin metabolism in keratinocytes (HACAT cells) under limited glucose availability conditions. Increased glutamine metabolism results in secretion of aspartate, glutamate, malate, oxalacetate and fumarate. In vitro studies also show that aspartate, but not glutamate, facilitates Th17 differentiation. Finally, pharmacological inhibition of glutaminase (GLS) alleviates pathology in a mouse model of psoriasis. The experiments are well performed and the manuscript very well written. The figures are especially good. I have several minor suggestions and 2 major requests, since the in vivo experiments failed to demostrate the miR-31-mediated metabolic rewiring and keratinocytes/Th17 cells crosstalk in vivo.

Major points:
1. Fig 4g: this results of Th17 polarization is important, as the relevance of miR-31-induced secretome in the crosstalk between keratinocytes and T cells in psoriasis is one of the main conclusion of the study. I think much more work is required here:
1a: Which concentration of Asp and Glu were used? They are not indicated in the legend. More importnatly, how were they established? Several concentrations should be used.
1b:In Fig 3g. it is shown that miR-321 promotes release of Glu, Asp and oxalacetate, but also at lower levels of fumarate and malate. Therefore, oxalacetate, fumarate and malate need to be tested and their combinations (this is the expected situation in vivo). Fumarate through succination is a good candidate to promote Th17 polarization.
1c: Any idea about the mechanism of Asp-induced Th17 polarization?
2. Most of the experiments were performed in vitro. Only data presented in Fig 5 and Extended Data Fig 5 are in vivo. I think more work is required in the in vivo models:
2a: Only T cells (CD4+) and neutrophil (MPO+) infiltration is analyzed upon GLS inhibition with CB-839. At least Th17 infiltration need to be tested to confim the in vitro results. A cytokine profiling may also strenghten the conclusions.
2b: In this model, the crosstalk between keratinocytes and T cells promoted by miR-31-induced secretome is not investigated. In addition, the ability of GSL inhibition with CB-839 to alleviate psoriasis in an IMQ mouse model was previously reported (Xia et al., 2020). Therefore, additional experiments to validate the in vitro results are required. For example, inhibition of glutamate/aspartate transporter, such SLC1A3 or SLC1A4, in the mouse model would demostrate the miR-31 regulated the crosstalk between keratinocytes and T cells in vivo via Glu/Asp release. Alternatively, Asp, or the best combination of metabolite able to promote Th17 differntiation in vitro (see my previous concern), could be topically applied and their effects in Th17 polarization and disease pathology evaluated.
Minor points:
1. Extended data Fig 2C: miR-31 reduced glucose uptake under basal condition and after insulin stimulation. Do you have evidences of the regulation of GLUT4 by miR-31?
2. Fig. 1C: ALDOC levels should be confirmed by RT-qPCR or WB
3. Fig. 2e & 2G: a clear impact of miR-31 and inhbition of MPC1 in lactate production is only observed after oligomycin addition. Any explanation?
4. Fig. 2F: the reduction of PDHX by miR-31 is not concincing. Better WB or quantification are required.

5. Fig. 3C: "Inhibition of GLS with CB-839 reduced glutamine uptake but did not inhibit miR-31 induced increase, but fully inhibited glutamate secretion in the presence or absence of miR-31". This sentence is confusing and should be rewritten. In any case, how can this result be explained?
6. Extended data Fig. 3a. The involvement of the cysteine-glutamate antiporter in glutamate release may be clarified by by measuring GSH levels.
7. Fig. 3d: miR-31 expression increased mitochondrial oxygen consumption under low glucose conditions and this was dependent on extracellular glutamine. However, this effect was only observed for maximal, but no basal,respiration. Any explanation?
8. Fig 3f: not cited in the text.
9. Fig 3h: the effect of miR-312 in lacttae derived from glutamin is very weak.10. Fig. 4e: Why il21, il8, il6, vegf and S100A8 are induced by CB-839? This is not obvious and no explanantion is given.
10. Fig 4f: has this experiment performed in the absence of glucose? If not, would you expect different results or TNFa favors glutamine use over glucose?
11: Fig 5 and extended data Fig 5: GLS is incued in the IMQ-induced psoriasis model but not in human samples. Why GS is not analyzed by IHC in the mouse model?
12: Extended Dta Fig. 2: GLUT1 may be also quantified by FC.
13: Fig. 5i: this model is confusign and does not include most results.

Referee #2 (Comments on Novelty/Model System for Author):

The work from Wang et al. studied the role of miR 31 in energy metabolism in keratinocytes and with regard to psoriasis. The topic of the study is new, interesting and relevant. Moreover, the team has generated a lot of data and this merits acclaim. Data are well presented. However, the manuscript is too dense that renders its reading unease and contains unnecessary results. Moreover, I have several concerns (please see my answer to authors).
Beside some technical flaws, my major concern is conceptual. Most experiments were carried out with keratinocyte cell lines overexpressing miR31 to mimic increased expression of miR31 in psoriatic epidermis. However, miR31 is increased in the spinous layer of the epidermis, so in differentiated keratinocytes. Authors carry out experiments in proliferating control cells, whose metabolism is totally different from than in differentiated (psoriatic) cells. Moreover, authors show that miR31 might decrease glycolysis and increase mitochondrial function in keratinocytes. However, in the discussion, they state that in suprabasal epidermal layers low PO2 decreases mitochondrial function. How do suprabasal epidermal cell survive in psoriasis if they are not relying on glycolysis due to high miR31 or on OXPHOS due to low PO2. ATP has to be produced via one of this pathway. Increased anaplerosis is just providing the TCA with substrates to produce NADH for OXPHOS. It is an ancillary pathway for TCA.
The abundance and quality of experiments are not able to cover the weakness of the concept. It is difficult to conceptually reconcile in vitro and in vivo data. This work might require another approach to demonstrate authors´ hypothesis.

Referee #2 (Remarks for Author):

The work from Wang et al. studied the role of miR 31 in energy metabolism in keratinocytes and with regard to psoriasis.
The topic of the study is new, interesting and relevant. Moreover, the team has generated a lot of data and this merits acclaim. Data are well presented.
However, the manuscript is too dense that renders its reading unease and contains unnecessary results (see below). Moreover, I have several concerns:
Concept:
Most experiments were carried out with keratinocyte cell lines ectopically rexpressing miR31 to mimic increased expression of miR31 in psoriatic epidermis. However, miR31 is increased in the spinous layer of the epidermis, so in differentiated keratinocytes. Authors carry out experiments in proliferating control cells, whose metabolism is totally different from than in differentiated cells. Thus, authors should repeat experiments with keratinocytes treated with calcium for 24h to mimic early stage of differentiation, similar to spinous cells.
In the abstract and in the introduction, authors claim that skin immune cells might uptake glucose from the microenvironment to fulfill their metabolic requirement i.e. increase glycolysis to sustain activation and that this decreases glucose availability for keratinocytes that should, in turn, survive in a glucose-limited environment. They refer to what occurs in solid tumor where cancer cells uptake most glucose, which reduces its availability for immune cells, hence leading to immune cell anergy. However, in solid tumor, immune cells are in or surrounding the tumor and all tumor cells increase the glycolytic pathway. In psoriasis, immune cells do not surround keratinocytes - they are in the dermis - and proliferating keratinocytes using glucose are only the basal keratinocytes (<10 % of epidermal cells). Moreover, basal keratinocytes are supplied with glucose via the blood stream. Thus, it is likely that increased use of glucose by immune cells in psoriatic skin is not capable to significantly limit glucose availability for keratinocytes. Moreover, the proportion of immune cells is increased in psoriatic skin but probably not to an extent

able to deprive keratinocytes in glucose.
A previous work from Zhang et al. (2018) et al showed that glycolysis is increased in psoriatic skin and associated with increased expression of GLUT1. Current work shows that miR31 is upregulated in psoriasis and decreases GLTU1 expression and glycolysis in proliferating cells (see additional comment for Fig 5). Can authors explain this discrepancy?
Results:
miR31 cells would upregulate glutamine metabolism to provide the TCA with metabolic intermediates. However, in these cells, the glycolysis is reduced as well as the pyruvate uptake. Where is the acetyl-coA coming from?
Anaplerosis is used when cells have increased TCA turn over. Which data (metabolimic, proteomic, expression) demonstrate increased TCA turn over in miR31 cells?
Fig. 1d: ATP and ADP are reduced. What does it mean energetically for cells?
Fig. 1d: glucose is increased in cells despite reduced GLUT1 and reduced glycolysis (Fig. 2). It is very surprising.
Fig.1e: authors should precise which genes are down versus up-regulated to facilitate readability and analysis of the results.
Authors write: "Our SILAC data indicate that miR-31 expression also reduced Pyruvate dehydrogenase complex member X (PDHX) and Pyruvate Carboxylase (PC) expression (Fig. 1c) and this was confirmed by western blotting (Fig. 2f). Both enzymes regulate entry of pyruvate into the mitochondria and therefore decreased expression may divert pyruvate flux towards lactate". Is LDHA activity increased in miR31 cells?
Seahorse experiments:
The ratio OCR/ECAR should be calculated for all experiments to give an indication on the relative utilization of mitochondrial (oxidative) versus glycolytic pathways for energy production. Similar remark for the mitochondrial function: the quantification of basal mitochondrial respiration, non-mitochondrial respiration, spare respiration, proton leak and ATP linked respiration is necessary to better understand changes in mitochondrial function in miR31cells.
Expression and activity of OXPHOS complex should be measured to support authors´ statements.
Western blot analysis should be quantified.
Fig 4a: the experimental conditions are not physiopathologically relevant.
Fig. 4e: expression of cytokines is not enough. Results should be supported by ELISA assay for most relevant cytokines.
Fig. 4f: the comparison with TNF-a effects is a bit far-fetched.
Fig. 4g: what is the connection between the experiments that have been carried out and the secretome of miR31 cells? Treatment of naive T cells with various cytokine cocktails have been already extensively studied. Authors should treat native T cells with miR31 cell secretome instead.
Fig. 5a-b: this has been extensively published and thus are unnecessary data.
Fig. 5d: staining for GLUT1 is unusual as GLUT1 is mainly expressed in plasma membrane of basal keratinocytes. When intracellular, GLUT1 is mainly in the ER. The uniformed staining speaks for technical problems.
Authors write: "Nutrient availability is likely not homogenous in the skin, with only the basal layer being adjacent to blood vessels and upper layers therefore relatively devoid of nutrients". This is not correct. Without nutrients, cells dye. Fatty acids, amino acids and pyruvate are available in suprabasal epidermal layers, as probably a bit of glucose as well.
Fig. 5: GLUT1 is increased in psoriasis as well as miR31 but ectopic expression of miR31 in proliferating keratinocytes reduces GLUT1. The use of cells treated with calcium would facilitate the translation of in vitro to in vivo data. It is important because it is likely that the metabolism in proliferating versus differentiated cells is different. Otherwise, two many parameters are different including pO2.
Discussion:
Authors write: "We present evidence that miR-31 expression induces metabolic rewiring through a combined deregulation of enzymes involved in glycolysis, glutaminolysis and lipid metabolism." Lipid metabolism has not been studied.
Authors write: "We observed that miR-31 expression limits glucose flux towards mitochondrial metabolism whereas it upholds lactate production and increases mitochondrial respiration." As authors write this sentence after their statement on psoriasis, this might mislead the readership. This has only been studied in cell lines, not in psoriatic skin.
Authors write: "Nutrients and O2 are provided by vasculature present below the basal layer and as such a gradient of nutrients upwards will emerge. Similar to glucose there will be limited O2 available and skin hypoxia along the same line of reasoning may be an important consequence of psoriasis. Hypoxia inhibits the mitochondrial electron transport chain and thereby aspartate synthesis (Garcia-Bermudez et al., 2018)." In their work, authors show that miR31 might decrease glycolysis and increase mitochondrial function. However, in the discussion, they state that in suprabasal epidermal layers low PO2 decreases mitochondrial function. How do suprabasal epidermal cell survive in psoriasis if they are not relying on either glycolysis due to high mir31 or OXPHOS due to low PO2. ATP has to be produced via one of this pathway. The amino acid pathway is only an ancillary pathways for TCA.
The discussion contains several overstatements (e.g. role of stem cells in psoriasis).

Referee #3 (Remarks for Author):

Comments to the manuscript by Wang et al. "Hsa-miR-31-1 5p controls a metabolic switch in psoriatic keratinocytes that identifies therapeutic intervention".
This study unravels a new functional role for hsa-microRNA-31-5p (miR-31), which has been previously shown to be one of the top upregulated miRNAs in psoriasis. The authors elegantly demonstrate that upregulated by limited glucose availability, miR-31 induces glutamine metabolism, enabling the survival and proliferation of keratinocytes. Moreover, miR-31 induces glutamine-dependent secretion of metabolites and cytokines, which in hand can activate Th17 cell differentiation, a hallmark of psoriasis.

Inhibiting the crosstalk between T-cells and keratinocytes by blocking glutaminase (GLS) alleviated psoriasis pathology in a mouse model. This study suggests that disconnecting the metabolic interaction could effectively treat psoriasis.
The text is well written, and the results are clear. It should be considered for publication after addressing the points listed below:
- Chapter "MiR-31 induced secretome induces Th17 differentiation"; Please add a reference to Figure 4g. Also, it is not clear why TGFb1 together with IL-6 and IL-21 were selected to stimulate naïve T-cells, as there were many significantly induced cytokines in the miR-31 induced secretome (Figure 4e and f). More experimental evidence is needed to conclude that 'miR-31-induced secretory phenotype is sufficient to induce Th17 cell differentiation (line 48-49). For example, will Th17 cell differentiation be induced by the secretome of keratinocytes with miR-31 overexpression? If so, will such effects be alleviated by blocking some crucial cytokines?
- Line 380; ...in psoriatic lesions GS expression was decreased in the spinous and granular layer concordant with miR-31 regulating GS and expression of miR-31 being low in the basal layer compared to an increase in the spinous and granular layer." Based on the immunostaining in Fig. 5d, GS expression seems low (or absent) in normal human skin but clearly detectable in cells of the basal layer of the skin in human psoriatic lesions. Please replace decreased (decreased from what?) with a more appropriate term.
- Chapter "Role for miR-31-induced metabolic regulation in psoriasis". Here, the authors tested the effect of CB-839 treatment in the IMQ mouse model. How is miR-31 expressed in the CB-839 treated skin lesions? Is there any reduction after treatment compared to non-treated skin? How would the authors speculate if a topical miR-31 inhibitor would be used in the IMQ-model? Could this potentially result in similar effects seen with the CB-893 inhibitor?
- Line 436; minor typos "...when measuring flux undr glucos limiting conditions...".

Point by point response

Referee #1 (Comments on Novelty/Model System for Author):
This study uses a comination of elegant techniques, including metabolomics and SILEC, to demonstrate metabolic rewiring and crosstalk between keratinocytes and T cells. My main concerns is that most experimenst were performed in vitro in human keratinocyte cell line (HACAT) and the in vivo experiments (mouse model of psoriasis) are scarce and should provide more mechanistic information.

(Remarks for Author):
This study elegantly demostrates that miR-31 increases glutamin metabolism in keratinocytes (HACAT cells) under limited glucose availability conditions. Increased glutamine metabolism results in secretion of aspartate, glutamate, malate, oxalacetate and fumarate. In vitro studies also show that aspartate, but not glutamate, facilitates Th17 differentiation. Finally, pharmacological inhibition of glutaminase (GLS) alleviates pathology in a mouse model of psoriasis. The experiments are well performed and the manuscript very well written. The figures are especially good. I have several minor suggestions and 2 major requests, since the in vivo experiments failed to demostrate the miR-31-mediated metabolic rewiring and keratinocytes/Th17 cells crosstalk in vivo.

Major points:
1. Fig 4g: this results of Th17 polarization is important, as the relevance of miR-31-induced secretome in the crosstalk between keratinocytes and T cells in psoriasis is one of the main conclusion of the study. I think much more work is required here:

1a: Which concentration of Asp and Glu were used? They are not indicated in the legend.

This is corrected. The concentrations are now provided in the figures.

More importnatly, how were they established? Several concentrations should be used.

In our initial report (Fig. 4G), we used a concentration of 25µM of aspartate. We made this choice on the basis of the following
(-) The formula for RPMI 1640 medium, which was originally developed for culturing human leukemia cells, contains 150µM of L-Aspartate and 136µM of L-Glutamate, of note both of these amino acids are not present in DMEM medium. Thus, these concentrations of aspartate and glutamate in RPMI 1640 medium served as our main reference of maximum concentration for culturing non-adherent immune cells.
(-) Following culturing of HaCat cells in DMEM (non-treated, miRNA-mimic control and miR31 expressing) we measured amino acids in the conditioned medium (see e.g. results presented in figure 3I) and calculated the mean concentration of aspartate secreted in the cell medium. These were found to be approximately 10 µM in non-treated control and scramble miRNA mimic overexpression cells and 25-30 µM in miR-31 mimic overexpression cells. As these concentrations did not exceed that of RPMI medium, these served as our reference for minimal concentration to test on Th17 differentiation.
In response to the reviewer’s suggestion, we now measured the effect of a range of aspartate and glutamate concentrations on induction of Th17 differentiation and observed that only aspartate in a dose-dependent manner can significantly enhance Th17 differentiation (novel Fig. EV 4G in manuscript)

The formula of RPMI1640 can be found here: https://www.thermofisher.cn/cn/zh/home/technical-resources/media-formulation.114.html
The formula of DMEM can be found here: https://www.thermofisher.cn/cn/zh/home/technical-resources/media-formulation.9.html.

1b:In Fig 3g. it is shown that miR-321 promotes release of Glu, Asp and oxalacetate, but also at lower levels of fumarate and malate. Therefore, oxalacetate, fumarate and malate need to be tested and their combinations (this is the expected situation in vivo). Fumarate through succination is a good candidate to promote Th17 polarization.

We agree with the reviewer and actually, we did initially consider this possibility, however a recent publication (Xu et al. reference 1 here) showed that neither 0.5mM succinate, 50μM fumarate, 0.5mM malate, 0.5mM citrate, and 0.5mM α-KG could enhance the proportion of Th17 cells induced by administration of a cytokine cocktail (IL-1β, IL-6, TGFβ, IL-23, IFN-γ, IL-4) similar to the cocktail we use. Thus, we did not consider these metabolites further.
In addition, the same study implies a crucial role of GOT1, which metabolizes aspartate and produces oxalacetate, in the differentiation of Th17 cells, also suggesting that aspartate as input is more relevant than oxalacetate in the process of Th17 differentiation. This is why we focus on aspartate rather than oxalacetate. We refer now to this study to explain our approach and choice for aspartate specifically

Figure for reviewers removed

2. Most of the experiments were performed in vitro. Only data presented in Fig 5 and Extended Data Fig 5 are in vivo. I think more work is required in the in vivo models:

2a: Only T cells (CD4+) and neutrophil (MPO+) infiltration is analyzed upon GLS inhibition with CB-839. At least Th17 infiltration need to be tested to confim the in vitro results. A cytokine profiling may also strenghten the conclusions.

We have done this for IL17 and IL10 (novel figure 5H and EV 6K)

2b: In this model, the crosstalk between keratinocytes and T cells promoted by miR-31-induced secretome is not investigated. In addition, the ability of GSL inhibition with CB-839 to alleviate psoriasis

in an IMQ mouse model was previously reported (Xia et al., 2020). Therefore, additional experiments to validate the in vitro results are required. For example, inhibition of glutamate/aspartate transporter, such SLC1A3 or SLC1A4, in the mouse model would demostrate the miR-31 regulated the crosstalk between keratinocytes and T cells in vivo via Glu/Asp release. Alternatively, Asp, or the best combination of metabolite able to promote Th17 differntiation in vitro (see my previous concern), could be topically applied and their effects in Th17 polarization and disease pathology evaluated.

Upon reviewer's suggestion, we performed the following experiments (novel Fig 4G and 4H):
Firstly, to strengthen the in vivo part, we performed additional mouse experiments in which we tested the effect of AOA (GOT1 inhibitor) and TFB-TBOA (SLC1A3 inhibitor) and compared these to CB839 (GLS inhibitor) in the IMQ mouse model.
Garcia-Bermudez et al. (ref. 2)) showed that 20 μM of TFB-TBOA inhibits cellular uptake of aspartate by cancer cells, indicating that indeed as suggested by the reviewer, we may consider effects of this compound to be indicative of blocking aspartate-mediated crosstalk between keratinocytes and naïve CD4+ T cells in the context of psoriatic lesioned skin. To demonstrate that this mechanism is indeed operational next to cancer cells also in T-cells, we tested this first in vitro. As shown, TFB-TBOA significantly inhibited in vitro Th17 differentiation in the presence of aspartate. In addition, aspartate taken up by the naïve CD4+ cells is metabolized to oxalacetate by GOT1, a process described to be essential for the differentiation of Th17 cells (ref. 1). We therefore also intervened using inhibitors of GOT1, including AOA and iGOT1, and found that both of them significantly inhibited in vitro Th17 differentiation (Fig. 4G and 4H).
Next, we used these inhibitors in vivo, and we observed that the SLC3A1 and GOT1 inhibitors can alleviate psoriatic skin damage (novel Fig 5C, 5D, 5E, 5F, 5G), and induce consequent histopathological changes. The disease parameters we analyzed, PASI score, spleen index, epidermal thickness, and baker score, were all suppressed by AOA, TFB-TBOA, and comparable to CB-839 (5E to 5F). In addition, we collected the splenic lymphocytes from these mice and stained them with CD3, CD4, and RORγt antibodies for Th17 cells. As shown in figure 3g, AOA, TFB-TBOA, and CB-839 displayed a significant inhibitory effect on Th17 differentiation in vivo as well (novel Fig 5H).
Finally, we also measured the serum level of IL-17A in the mice treated with different inhibitors by ELISA and found that all there three inhibitors suppress IL-17A induced by IMQ (novel Fig 5H). No significant difference was observed in the serum level of IL-10 (novel Fig. EV 6K). Unfortunately, despite testing a multitude of different antibodies and IHC protocols we were unable to obtain reliable (non-specific, high background etc.) Th17 staining in mouse skin.
Taken together, we believe these data reinforce the validity of our model in the in vivo setting as requested by the reviewer.

Minor points:

1. Extended data Fig 2C: miR-31 reduced glucose uptake under basal condition and after insulin stimulation. Do you have evidences of the regulation of GLUT4 by miR-31?

Zhang et al. (Nat Med *24*, 617-627, 2018) show that keratinocytes almost exclusively express GLUT-1 and no detectable GLUT-4. Indeed, using qPCR to detect mRNA expression, we do not/hardly observe mRNA expression of GLUT-4 in HaCaT cells. Also, in our proteomic data, GLUT4 is not identified as regulated by miR-31 and when measured by western blot we do not observe clear/detectable GLUT-4 expression. It should be noted that whilst GLUT-4 is the relevant glucose transporter in insulin sensitive tissue, GLUT-1 is also responding to insulin/growth factors see for example ref 3.

2. Fig. 1C: ALDOC levels should be confirmed by RT-qPCR or WB

Actually, we have measured ALDOC by western blot and saw a slight decrease after miR-31 overexpression in HaCat cells. However, at least the antibodies we used were apparently not of high quality. Measuring RNA expression by qPCR shows a reduction in RNA expression following ectopic miR-31 expression
(RT-PCR result added to Fig. 2C).

3. Fig. 2e & 2G: a clear impact of miR-31 and inhbition of MPC1 in lactate production is only observed after oligomycin addition. Any explanation?

Cells produce ATP primarily from mitochondrial respiration and glycolysis; therefore, when ATP from mitochondrial respiration is fully inhibited by oligomycin, glycolysis is significantly increased to compensate for the ATP deficiency. In this case, blocking MPC1 (UK5009) or PC (miR-31) would even further reduce the amount of pyruvate entering the mitochondria, thus promoting lactate production to maintain glycolysis and ATP production.

4. Fig. 2F: the reduction of PDHX by miR-31 is not concincing. Better WB or quantification are required.

We have measured PDHX expression by western blot multiple times in independent experiments and always observed a small but significant reduction. We provide now a better WB (updated Fig 2F) as well as RNA expression (new Fig EV 2F). Both show a miR-31-induced decrease in expression. We do like to note that in our approach, we argued that the biological relevance of expressing a miRNA, results from regulating multiple different RNAs/encoding proteins that are operational within one process. This to us indicates that miRNAs do not necessarily downregulate RNA expression always strongly, as multiple small effects can be as efficient in regulating a biological process. Irrespective, miR-31 overexpression in HaCat cells reduces PDHX both at protein and RNA level.

5. Fig. 3C: "Inhibition of GLS with CB-839 reduced glutamine uptake but did not inhibit miR-31 induced increase, but fully inhibited glutamate secretion in the presence or absence of miR-31". This sentence is confusing and should be rewritten. In any case, how can this result be explained?

This part has been rewritten.
These results show the total consumption of glutamine in cells under different conditions over 2 days. We think our results are best explained by the fact that the dose of CB-839 we used (10 μM) was not sufficient to completely block glutaminase-mediated metabolism, cells treated with CB839 still took up about 1.8 μmol of glutamine. The fact that glutamate secretion is fully blocked we attribute to the fact that either the small amount of glutamate still formed is not in excess and fully needed for further metabolism (converted to aKG) therefore glutamate is not secreted. Alternatively, glutamine maybe used for metabolism other than conversion to glutamate.

6. Extended data Fig. 3a. The involvement of the cysteine-glutamate antiporter in glutamate release may be clarified by by measuring GSH levels.

We thank the reviewer for this suggestion. We have looked back at our targeted metabolomics data and observed increased extracellular GSH/GSSG in the medium of HaCat cells that overexpressed miR-31 (see figure panel A for review purpose). As shown by Hayashima and Katoh (ref 4), secreted glutathione can be converted to cystine by the action of GGT1 and this can result in cystine import in case media lack sufficient supplemented cystine. Increased glutamate release is known to inhibit the cystine-glutamate antiporter and according to above referenced paper, consequently the conversion of GSH/GSGG to cystine (by means of product inhibition), Hence, we conclude that the increase observed in glutathione after miR-31 expression corroborates the involvement of the cystine-glutamate antiporter as suggested by the reviewer. Of note, relating to the issue of proliferating versus differentiated cells, we observe a similar increase in GSH/GSSG in both proliferating HaCat and CaCl2 treated (differentiated) HaCaT cells. Although supporting evidence for Xct antiporter involvement we considered this argument not improving readability so we choose to not incorporate these data.

7. Fig. 3d: miR-31 expression increased mitochondrial oxygen consumption under low glucose conditions and this was dependent on extracellular glutamine. However, this effect was only observed for maximal, but no basal,respiration. Any explanation?

We think this is due to the experimental set-up. In figure 3d, before we start the Seahorse measurement, cells are cultured in basal Seahorse medium supplemented with 1mM glucose and 2mM pyruvate but without glutamine for at least 30 minutes. We think this reduces the difference in the basal state. Indeed, we observed basal OCR increased significantly in miR-31 overexpression HaCat cells in figure 2E and 3E. In these two experiments, cells were precultured in a basal medium supplemented with 2mM glutamine and 1mM glucose.

8. Fig 3f: not cited in the text.

Thank you for this and this has been corrected.

9. Fig 3h: the effect of miR-312 in lacttae derived from glutamin is very weak.

The main source of lactate is likely still glucose and not glutamine amongst others because we see that glucose-derived pyruvate entry is inhibited by miR-31. In this study, through tracing the metabolism of C13-glutamine, we found that glutamine can be metabolized to produce M3+ lactate. And we did see that the miR-31 overexpression HaCat secreted more M3+ lactate than the control in figure 3h. This finding is supporting evidence that miR-31 improves glutaminolysis, which serves multiple purposes including contributing (to some extend) to lactate secretion.

10. Fig. 4e: Why il21, il8, il6, vegf and S100A8 are induced by CB-839? This is not obvious and no explanantion is given.

Initially we generated a list of cytokines that (-) in literature are more or less implicated in psoriasis (-) we thought might be interesting to measure. Subsequently, the level of these cytokines was measured in samples of cells ectopically expressing miR-31 and compared to control and CB-839 treated samples. We used TNF treatment as a control for possible NFkB mediated cytokine induction, also because miR-31 has been suggested to activate NFkB. We did not specifically intend to look for cytokines induced by CB-839, but in retrospect one could argue that if expression of certain cytokines is negatively regulated by CG-839

the opposite may also be the case. However, and also based on the suggestion of reviewer # 2 who considers TNF "far-fetched" we have decided to present our data in a different manner and consequently to take these data (on CB-839) out (now new Figure 4E).

10. Fig 4f: has this experiment performed in the absence of glucose? If not, would you expect different results or TNFa favors glutamine use over glucose?

This experiment was performed in the presence of 1mM glucose. It could very well be that TNF favors glutamine over glucose and although an interesting research question we have not studied this yet. Irrespective, considering the comments made by reviewer #2 also related to this figure panel, we have reorganized these data and now only show the effect of miR-31 expression on cytokines (fig 3F in the revised version).

11: Fig 5 and extended data Fig 5: GLS is incued in the IMQ-induced psoriasis model but not in human samples. Why GS is not analyzed by IHC in the mouse model?

GS IHC is shown in Fig EV6H and quantification shown in EV 6K

12: Extended Data Fig. 2: GLUT1 may be also quantified by FC.

We have quantified GLUT1 expression by RT-PCR this is now shown in Fig EV 2E

.

13: Fig. 5i: this model is confusign and does not include most results.

We agree this model is confusing and does not help the reader, so we removed it as we also have included an improved graphical abstract.

Referee #2 (Comments on Novelty/Model System for Author):

The work from Wang et al. studied the role of miR 31 in energy metabolism in keratinocytes and with regard to psoriasis. The topic of the study is new, interesting and relevant. Moreover, the team has generated a lot of data and this merits acclaim. Data are well presented. However, the manuscript is too dense that renders its reading unease and contains unnecessary results. Moreover, I have several concerns (please see my answer to authors).

Beside some technical flaws, my major concern is conceptual. Most experiments were carried out with keratinocyte cell lines overexpressing miR31 to mimic increased expression of miR31 in psoriatic epidermis. However, miR31 is increased in the spinous layer of the epidermis, so in differentiated keratinocytes. Authors carry out experiments in proliferating control cells, whose metabolism is totally different from than in differentiated (psoriatic) cells. Moreover, authors show that miR31 might decrease glycolysis and increase mitochondrial function in keratinocytes. However, in the discussion, they state that in suprabasal epidermal layers low PO2 decreases mitochondrial function. How do suprabasal epidermal cell survive in psoriasis if they are not relying on glycolysis due to high miR31 or on OXPHOS due to low PO2. ATP has to be produced via one of this pathway. Increased anaplerosis is just providing the TCA with substrates to produce NADH for OXPHOS. It is an ancillary pathway for TCA.

The abundance and quality of experiments are not able to cover the weakness of the concept. It is

difficult to conceptually reconcile in vitro and in vivo data. This work might require another approach to demonstrate authors´ hypothesis.

Referee #2 (Remarks for Author):

The work from Wang et al. studied the role of miR 31 in energy metabolism in keratinocytes and with regard to psoriasis.
The topic of the study is new, interesting and relevant. Moreover, the team has generated a lot of data and this merits acclaim. Data are well presented. However, the manuscript is too dense that renders its reading unease and contains unnecessary results (see below). Moreover, I have several concerns:

Concept:
Most experiments were carried out with keratinocyte cell lines ectopically rexpressing miR31 to mimic increased expression of miR31 in psoriatic epidermis. However, miR31 is increased in the spinous layer of the epidermis, so in differentiated keratinocytes. Authors carry out experiments in proliferating control cells, whose metabolism is totally different from than in differentiated cells. Thus, authors should repeat experiments with keratinocytes treated with calcium for 24h to mimic early stage of differentiation, similar to spinous cells.

We agree with the reviewer that there may be metabolic differences between proliferating and differentiated keratinocytes/cells. However, we would like to note that this does not appear to be always the case. For example, for fibroblasts it has been shown that quiescent fibroblasts to do not significantly differ in glucose flux activity compared to their proliferating counterparts (ref 4). Likely more important in the context of our findings, it has been shown that with respect to glutamine metabolism quiescent epithelial cells do differ from proliferating epithelial cells, where quiescent cells display altered glutamate metabolism (ref 6). Therefore, as suggested by the reviewer we have repeated several key experiments but now using HaCaT cells treated with 2 mM $CaCl_2$, which induces differentiation as indicated by the upregulation of Keratin 10 a marker for the spinous layer (ref 7, see also Fig EV 4A). We observed that $CaCl_2$ treated differentiated HaCaT cells ectopically expressing miR-31,
(-) reduced expression of identified miR-31 targets (GS (Fig EV 4B), PDHX (Fig. EV 2F)), but also GLUT1 (Fig. EV 2E)
(-) Increased lactate production upon glucose re-addition (Fig EV4C), including increased LDH activity (Fig. EV 2G)
(-) increased mitochondrial oxygen consumption under low glucose conditions dependent on extracellular glutamine (Fig EV 4D)
(-) displayed increased secretion of glutamate and aspartate (Fig EV 4F), the latter supporting Th17 differentiation.
Taken together, we believe that these data allow to conclude that our main findings also apply to differentiated (CaCl2 treated) HaCaT cells.

In the abstract and in the introduction, authors claim that skin immune cells might uptake glucose from the microenvironment to fulfill their metabolic requirement i.e. increase glycolysis to sustain activation and that this decreases glucose availability for keratinocytes that should, in turn, survive in a glucose-limited environment. They refer to what occurs in solid tumor where cancer cells uptake most glucose, which reduces its availability for immune cells, hence leading to immune cell anergy. However, in solid tumor, immune cells are in or surrounding the tumor and all tumor cells increase the glycolytic pathway. In psoriasis, immune cells do not surround keratinocytes - they are in the dermis - and proliferating

keratinocytes using glucose are only the basal keratinocytes (<10 % of epidermal cells). Moreover, basal keratinocytes are supplied with glucose via the blood stream. Thus, it is likely that increased use of glucose by immune cells in psoriatic skin is not capable to significantly limit glucose availability for keratinocytes. Moreover, the proportion of immune cells is increased in psoriatic skin but probably not to an extent able to deprive keratinocytes in glucose.

The concept of metabolic competition, or for that matter metabolic cooperation, has been developed by and large in the context of cancer models. We agree that a skin disease like psoriasis is different, but we think to some extend also alike. Similar, but of course not identical to cancer, it is typified by increased proliferation and inflammation. To generalize our data and come to a model using the data we present, we used the cancer model for analogy. It was/is not our intention to state that this model would be absolute, in the sense that competition is as fierce in cancer, however we do think that it is a valid frame of thinking to develop a model to understand this disease. In this respect, we appreciate the reviewer's questioning of our model because it helped to sharpen our model. Also, because the questioning states some assumptions that in all fairness we simply do not know at present. For example, "it is likely that increased use of glucose by immune cells in psoriatic skin is not capable to significantly limit glucose availability". This may indeed be likely (or not), but without proof we just do not know for sure. As the reviewer rightfully points out the the majority of proliferating (glycolytic) keratinocytes in general and in psoriasis is located in the basal layer. Thus, it could be possible that a combination of increased proliferation in the basal layer and activated immune cells, rather than only immune cells as we originally suggested, result in reduced availability of glucose in the spinous layer. The experimental evidence we provide for this possibility is that we observed miR-31 expression to increase when cells are grown under low glucose conditions or when treated with 2-DG. Combined this may explain why miR-31 expression is increased in the spinous layer. We do hope that the reviewer agrees that our model proposed provides a mean to understand and consequently is also subject to discussion. However, we have removed all reference to the cancer model throughout the manuscript and have toned down our discussion as to not provide the suggestion that the cancer model applies one on one with psoriasis. By taking into account the concerns expressed by the reviewer regarding the comparison to cancer we provide hopefully now a more balanced viewpoint.

A previous work from Zhang et al. (2018) et al showed that glycolysis is increased in psoriatic skin and associated with increased expression of GLUT1. Current work shows that miR31 is upregulated in psoriasis and decreases GLTU1 expression and glycolysis in proliferating cells (see additional comment for Fig 5). Can authors explain this discrepancy?

We thank the reviewer for pointing out the distinction between the basal layer and spinous layer, also with respect to the above. When taking this distinction into account we believe that the data of Zhang et al. are quite complimentary to ours and not discrepant. As mentioned above we (fig ...) and others show that in psoriasis miR-31 is highly expressed in the spinous layer (mostly differentiated keratinocytes), but not in the basal layer (mostly proliferating) keratinocytes. Thus, as in vivo miR-31 is not significantly expressed in the basal layer there will also be no effect on GLUT-1/glycolysis within the basal layer (at least not miR-31 mediated). We also observe reduced expression of GLUT-1 in the spinous layer (as observed in our in vitro analysis), thus, in essence this compartmentalization (basal versus spinous layer) within the skin further supports our hypothesis of glutamine dependency within the spinous layer that is enabled by miR-31 expression. So, our data combined with the data from Zhang et al. clearly suggest that the basal layer metabolically differs from the spinous layer and that either metabolic targeting of the basal layer (by GLUT-1 inhibition) or the spinous layer (by GLS1 inhibition) shows similar efficacy in

treatment of psoriasis. Interestingly, Zhang et al. also showed a distinction between applying topically a GLUT-1 inhibitor versus genetic deletion of GLUT-1 in the skin.

"Thus, while genetic Glut1 deletion rescued only the epidermal acanthosis and proliferation, the topical, chemical inhibition of glucose transport rescued both keratinocyte proliferation and prevented inflammation in animal and organoid models of psoriasiform inflammation." (copied from Zhang et al. 2018; conclusion part written at the end of result section)

This indicates that GLUT-1 is not as relevant for the cells in the spinous layer as for the cells in the basal layer, but also that CB-839 (and for that matter also (TFB- AOA) similar to the GLUT-1 inhibitor targets both keratinocytes and immune cells albeit through different mechanisms. So taken together we believe our data are rather complementary and not as much contradicting to the data presented by Zhang et al..

Results:
miR31 cells would upregulate glutamine metabolism to provide the TCA with metabolic intermediates. However, in these cells, the glycolysis is reduced as well as the pyruvate uptake. Where is the acetyl-coA coming from?

We consider reductive carboxylation (ref 5) the most likely pathway to maintain Acetyl-Co A levels. We have however not studied this.

Anaplerosis is used when cells have increased TCA turn over. Which data (metabolimic, proteomic, expression) demonstrate increased TCA turn over in miR31 cells?
Our explanation is two-fold. First, we think anaplerosis can also result from limited availability of glucose derived pyruvate to feed the TCA cycle so not necessarily increased turnover. Second, the increase in OCR following miR-31 expression indirectly suggests that there is increased TCA, as this feeds the OXPHOS. So, we think it is a combination of both processes.

Fig. 1d: ATP and ADP are reduced. What does it mean energetically for cells?

To address this question, we measured the effect of miR-31 expression on AMPK activity. Phosphorylation of AMPK as well as phosphorylation of its major substrate (ACC2) did not significantly change upon expression of miR-31 mimic or inhibitor (see panel B Figure for reviewing purpose). We conclude from this that miR-31 expressing cells do not significantly differ in this aspect of energy metabolism.

Fig. 1d: glucose is increased in cells despite reduced GLUT1 and reduced glycolysis (Fig. 2). It is very surprising.

This may indeed seem surprising, but our explanation is that in 1d we look at steady state level and GLUT1 is a passive transporter so if glucose taken up by cells will not, or inefficiently, be further metabolized the level will increase (as long as sufficient external glucose is present). We show that glycolytic enzymes are repressed (fig 2) and HK2 responsible for converting glucose into glucose-6-phosphate (that can no longer be exported by GLUT1) is also reduced. So, despite the reduced GLUT1 expression, we can still see increased substrate levels for glycolysis (at steady state). One could consider this similar to substrate increase upon inhibition of an enzyme that converts this substrate.

Fig.1e: authors should precise which genes are down versus up-regulated to facilitate readability and analysis of the results.

We added a list of genes up or down regulated (Extended table 1)

Authors write: "Our SILAC data indicate that miR-31 expression also reduced Pyruvate dehydrogenase complex member X (PDHX) and Pyruvate Carboxylase (PC) expression (Fig. 1c) and this was confirmed by western blotting (Fig. 2f). Both enzymes regulate entry of pyruvate into the mitochondria and therefore decreased expression may divert pyruvate flux towards lactate". Is LDHA activity increased in miR31 cells?

We measured LDH activity and observed an increase upon miR-31 expression and a slight decrease upon anti-miR-31 expression (Fig EV 2G).

Seahorse experiments:
The ratio OCR/ECAR should be calculated for all experiments to give an indication on the relative utilization of mitochondrial (oxidative) versus glycolytic pathways for energy production. Similar remark for the mitochondrial function: the quantification of basal mitochondrial respiration, non-mitochondrial respiration, spare respiration, proton leak and ATP linked respiration is necessary to better understand changes in mitochondrial function in miR31cells.

We have generated a supplementary table providing for all Seahorse experiments the requested parameters (now Extended table 6)

Expression and activity of OXPHOS complex should be measured to support authors´ statements.

We analyzed the expression of different OXHPOS complex proteins and did not observe significant changes after miR31 mimic or miR31 inhibitor expression (see panel C figure for reviewing purpose). Because the Seahorse experiments measures OXPHOS activity, we were not sure what additional experiments the reviewer requests. We choose not to add these data to our manuscript.

Fig 4a: the experimental conditions are not physiopathologically relevant.

This is correct, absolute glucose or glutamine starvation conditions likely do not exist under physiological or maybe even pathological conditions. However, such an experimental setup does help us to understand the viability of miR-31 cells in different metabolic environments. We have moved Fig4a to the extended figures.

Fig. 4e: expression of cytokines is not enough. Results should be supported by ELISA assay for most relevant cytokines.

We do not understand this remark/question. Luminex is a multiplexed ELISA assay.

Fig. 4f: the comparison with TNF-a effects is a bit far-fetched.

We agree and also based on remarks made by reviewer #1, we removed Figure 4f and replaced in with a new figure (4E)

Fig. 4g: what is the connection between the experiments that have been carried out and the secretome of miR31 cells? Treatment of naive T cells with various cytokine cocktails have been already extensively studied. Authors should treat native T cells with miR31 cell secretome instead.

We have cultured naïve T cells with different dilutions of conditioned medium derived from HaCaT cells either expressing miR-31 or negative control. We observed a consistent and significant 2-3 fold induction of Th17 cell differentiation by conditioned medium derived from miR-31 expressing HaCaT cells (new Fig 4F). This corroborates our suggestion that the miR-31 induced secretome supports Th17 cell differentiation.

Fig. 5a-b: this has been extensively published and thus are unnecessary data.
Indeed, similar data have been published, yet we think it is still relevant, if not to reassure only ourselves, to have independently validated these results from literature (reproducibility). So, these data have been re-allocated to supplementary data.

Fig. 5d: staining for GLUT1 is unusual as GLUT1 is mainly expressed in plasma membrane of basal keratinocytes. When intracellular, GLUT1 is mainly in the ER. The uniformed staining speaks for technical problems.

We do not fully agree with the conclusion that the staining is uniform, however we have supported the conclusion of this figure by performing RT-PCR as requested by the other reviewer and this shows that GLUT-1 RNA is down regulated in HaCaT cells after miR-31 expression both in proliferating and differentiated condition.

Authors write: "Nutrient availability is likely not homogenous in the skin, with only the basal layer being adjacent to blood vessels and upper layers therefore relatively devoid of nutrients". This is not correct. Without nutrients, cells dye. Fatty acids, amino acids and pyruvate are available in suprabasal epidermal layers, as probably a bit of glucose as well.

We tried to be cautious in our writing here and therefore we wrote "relatively devoid of nutrients" so we did not claim complete absence of nutrients. As reviewer rightfully states cells would then die. In any case we rewrote this part of the discussion as to avoid this confusion (see also discussion above).

Fig. 5: GLUT1 is increased in psoriasis as well as miR31 but ectopic expression of miR31 in proliferating keratinocytes reduces GLUT1. The use of cells treated with calcium would facilitate the translation of in vitro to in vivo data. It is important because it is likely that the metabolism in proliferating versus differentiated cells is different. Otherwise, two many parameters are different including pO2.

In response to reviewer's suggestion, we used CaCl2 to induce HaCaT cell differentiation and repeated several key experiments, the results show that in the differentiated state the response to miR-31 expression is by-and-large similar if not identical. (see response to reviewer enhance the glutaminolysis pathway and secrete large amounts of aspartate (see for details response above)

Discussion:
Authors write: "We present evidence that miR-31 expression induces metabolic rewiring through a

combined deregulation of enzymes involved in glycolysis, glutaminolysis and lipid metabolism." Lipid metabolism has not been studied.

Correct, we have not studied lipid metabolism, this is subject of further future studies and we corrected the writing.

Authors write: "We observed that miR-31 expression limits glucose flux towards mitochondrial metabolism whereas it upholds lactate production and increases mitochondrial respiration." As authors write this sentence after their statement on psoriasis, this might mislead the readership. This has only been studied in cell lines, not in psoriatic skin.

We have rewritten this and now state that our findings are in cell lines.

Authors write: "Nutrients and O2 are provided by vasculature present below the basal layer and as such a gradient of nutrients upwards will emerge. Similar to glucose there will be limited O2 available and skin hypoxia along the same line of reasoning may be an important consequence of psoriasis. Hypoxia inhibits the mitochondrial electron transport chain and thereby aspartate synthesis (Garcia-Bermudez et al., 2018)." In their work, authors show that miR31 might decrease glycolysis and increase mitochondrial function. However, in the discussion, they state that in suprabasal epidermal layers low PO2 decreases mitochondrial function. How do suprabasal epidermal cell survive in psoriasis if they are not relying on either glycolysis due to high mir31 or OXPHOS due to low PO2. ATP has to be produced via one of this pathway. The amino acid pathway is only an ancillary pathways for TCA.

Apparently, we were not clear in our writing and also based on the previous questions/remarks by this and other reviewer, we have removed this discussion part.

The discussion contains several overstatements (e.g. role of stem cells in psoriasis).

Agreed, we removed the stem cell part and rewrote the discussion as to remove or downtune several of the statements.

Referee #3 (Remarks for Author):

Comments to the manuscript by Wang et al. "Hsa-miR-31-1 5p controls a metabolic switch in psoriatic keratinocytes that identifies therapeutic intervention".
This study unravels a new functional role for hsa-microRNA-31-5p (miR-31), which has been previously shown to be one of the top upregulated miRNAs in psoriasis. The authors elegantly demonstrate that upregulated by limited glucose availability, miR-31 induces glutamine metabolism, enabling the survival and proliferation of keratinocytes. Moreover, miR-31 induces glutamine-dependent secretion of metabolites and cytokines, which in hand can activate Th17 cell differentiation, a hallmark of psoriasis. Inhibiting the crosstalk between T-cells and keratinocytes by blocking glutaminase (GLS) alleviated psoriasis pathology in a mouse model. This study suggests that disconnecting the metabolic interaction could effectively treat psoriasis.
The text is well written, and the results are clear. It should be considered for publication after

addressing the points listed below:
- Chapter "MiR-31 induced secretome induces Th17 differentiation"; Please add a reference to Figure 4g.

Also, it is not clear why TGFb1 together with IL-6 and IL-21 were selected to stimulate naïve T-cells, as there were many significantly induced cytokines in the miR-31 induced secretome (Figure 4e and f).

The secretome of keratinocytes with miR-31 overexpression was measured by Luminex for cytokines and targeted metabolomics for metabolites. As compared to control, miR-31 secretome contains abundant level of TGFb, IL-6, IL-21, aspartate, and glutamate. A role for TGFb, IL-6, IL-21 was already suggested by literature (see e.g ref 6 and 7) therefore our choice for these cytokines. However, a possible role for aspartate and/or glutamate on Th17 cell differentiation is still unknown and therefore tested in combination with these cytokines.

More experimental evidence is needed to conclude that 'miR-31-induced secretory phenotype is sufficient to induce Th17 cell differentiation (line 48-49). For example, will Th17 cell differentiation be induced by the secretome of keratinocytes with miR-31 overexpression? If so, will such effects be alleviated by blocking some crucial cytokines?

We have extensively changed this part (see answers to reviewer 1 and 2) and have now added data that conditioned medium of miR-31 expressing HaCaT cells can induce Th17 differentiation, that aspartate enhances cytokine induced Th17 differentiation and that this is relevant in vivo. We did not perform experiments using e.g. blocking antibodies to IL-21 as these experiments have already been done by others (see also ref 6 and 7)

- Line 380; ...in psoriatic lesions GS expression was decreased in the spinous and granular layer concordant with miR-31 regulating GS and expression of miR-31 being low in the basal layer compared to an increase in the spinous and granular layer." Based on the immunostaining in Fig. 5d, GS expression seems low (or absent) in normal human skin but clearly detectable in cells of the basal layer of the skin in human psoriatic lesions. Please replace decreased (decreased from what?) with a more appropriate term.

The sentence is replaced with:
GS expression was low in normal skin but increased in cells of the basal layer of the skin in psoriatic lesions. However, in the spinous and granular layer of psoriatic lesions GS expression remained low compared to basal layer (for quantification see Fig 5F).

- Chapter "Role for miR-31-induced metabolic regulation in psoriasis". Here, the authors tested the effect of CB-839 treatment in the IMQ mouse model. How is miR-31 expressed in the CB-839 treated skin lesions?

We have measured miR-31 expression following treatment (new Fig 5J) and expression is lowered by treatment with CB-839.

Is there any reduction after treatment compared to non-treated skin? How would the authors speculate if a topical miR-31 inhibitor would be used in the IMQ-model? Could this potentially result in similar effects seen with the CB-893 inhibitor?

The reviewer is correct in his/her conclusion. Indeed, mice with knockout of miR-31 show a reduction in IMQ-induced psoriasis-like skin changes (ref 7). It is thus clear that eliminating miR-31 can be an effective mean to improve psoriasis symptoms. However, miRNAs are usually involved in regulating the translation of hundreds and thousands of target genes (as we show here as well), so a knockdown/out of miR-31 may lead to unwanted side-effects. In addition, targeted delivery of anti-MiRs may be a challenge. Therefore, we hope to reveal the specific processes and molecular targets of miR-31 involved in psoriasis pathology, and then block the corresponding processes or inhibit specific targets to identify new therapeutic strategies, such as Aspartate metabolism and targets such as GLS, SLC1A3 and GOT1 identified in this study.

- Line 436; minor typos "...when measuring flux under glucos limiting conditions...".

Thanks. The sentence has been corrected.

References

1: Xu, T., et al. (2017). "Metabolic control of TH17 and induced Treg cell balance by an epigenetic mechanism." Nature 548(7666): 228-233.

2: Garcia-Bermudez J, Baudrier L, La K, et al. Aspartate is a limiting metabolite for cancer cell proliferation under hypoxia and in tumours [published correction appears in Nat Cell Biol. 2018 Oct;20(10):1228

3. Samih N, Hovsepian S, Aouani A, Lombardo D, Fayet G. Endocrinology. 2000 Nov;141(11):4146-55. Glut-1 translocation in FRTL-5 thyroid cells: role of phosphatidylinositol 3-kinase and N-glycosylation.

4: Hayashima K and Katoh H, Expression of gamma-glutamyltransferase 1 in glioblastoma cells confers resistance to cystine deprivation-induced ferroptosis J Biol Chem 2022 Mar;298(3):101703

5: Metallo CM, Gameiro PA, Bell EL, et al. Reductive glutamine metabolism by IDH1 mediates lipogenesis under hypoxia. Nature. 2011 Nov 20;481(7381):380-4.

6: van den Berg WB, McInnes IB (2013) Th17 cells and IL-17 a—focus on immunopathogenesis and immunotherapeutics. Semin Arthritis Rheum 43: 158-170

7: Zhou L, Lopes JE, Chong MM, et al. TGF-beta-induced Foxp3 inhibits T(H)17 cell differentiation by antagonizing RORgammat function. Nature. 2008;453(7192):236-240.

8: Yan S, Xu Z, Lou F, et al. NF-κB-induced microRNA-31 promotes epidermal hyperplasia by repressing protein phosphatase 6 in psoriasis. Nat Commun. 2015;6:7652.

.

Figures for review only

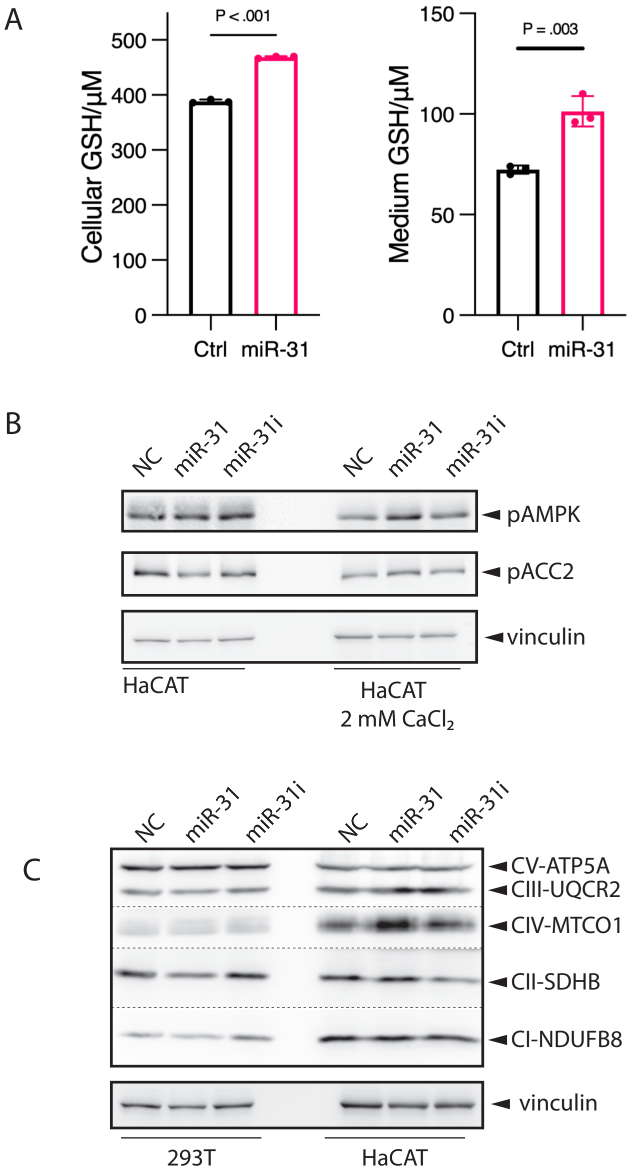

4th Oct 2022

Dear Prof. Burgering,

Thank you for the submission of your manuscript to EMBO Molecular Medicine, and please accept my apologies for the delay in getting back to you, as I sought further advice on your manuscript. We have indeed received the enclosed reports from the original three referees. As you will see, while two referees are satisfied with the revisions, referee #2 still finds the work unsuited for publication, mainly because of the use of HaCaT cells as a model system. As mentioned above, given these contradictory reports, I consulted with an external expert advisor, who stated:

"I agree that HaCaT cells are not an ideal model for this purpose, because they have a p53 mutation that may affect their metabolism. However, I am not quite as critical as the reviewer, because HaCaT cells are not transformed (they are just immortalized) and they differentiate normally in 3D cultures. Many highly relevant studies have been performed with HaCaT cells. However, they are not suitable for differentiation in 2D, in particular when they are already maintained in DMEM/10% FCS (and thus under relatively high Ca2+ concentrations).

I suggest that the authors perform a few experiments with primary human keratinocytes (which are commercially available). They could transfect these cells with miR-31 mimics as they did with HaCaT cells and then check the expression of a few selected genes/proteins (e.g. RPIA, GS, HK1 and IL6). This should be done under normal culture conditions (low calcium) and in response to Ca-induced differentiation. This is not difficult and should not take too much time, but it would show that the general effect of miR-31 is reproducible in primary human keratinocytes. In addition, they should address the other comments of the reviewer (at least by modification of the text), since they are relevant.

We would therefore like to invite you to revise the manuscript further following this advisor's recommendations. As EMBO Press usually encourages one single round of revisions, please be aware that this will be the last chance for you to address these concerns.

Moreover, please address the following editorial issues:

- All corresponding authors are required to supply an ORCID ID for their name upon submission of a revised manuscript. An ORCID identifier is currently missing for C. Lu and R. Huang.
- Please remember to deposit your primary datasets in an appropriate public database, and to list the accession numbers and database under 'Data Availability'.
- Please provide legends in the files for the Datasets EV1 and 2, as well as for EV tables.
- We would also encourage you to include the source data for figure panels that show essential data. Numerical data should be provided as individual .xls or .csv files (including a tab describing the data). For blots or microscopy, uncropped images should be submitted (using a zip archive if multiple images need to be supplied for one panel). Additional information on source data and instruction on how to label the files are available at
.
- We usually allow up to 5 EV figures, and we note that your manuscript currently has 6. Would it be possible to reduce the number of EV figures to 5? For the figures that you do not wish to display as Expanded View figures, they should be bundled together with their legends in a single PDF file called *Appendix*, which should start with a short Table of Content. Appendix figures should be referred to in the main text as: "Appendix Figure S1, Appendix Figure S2" etc.
- As part of the EMBO Publications transparent editorial process initiative (see our Editorial at http://embomolmed.embopress.org/content/2/9/329), EMBO Molecular Medicine will publish online a Review Process File (RPF) to accompany accepted manuscripts.
In the event of acceptance, this file will be published in conjunction with your paper and will include the anonymous referee reports, your point-by-point response and all pertinent correspondence relating to the manuscript. Let us know whether you agree with the publication of the RPF and as here, if you want to remove or not any figures from it prior to publication.
Please note that the Authors checklist will be published at the end of the RPF.

I look forward to receiving your revised manuscript.

Yours sincerely,

Lise Roth

Lise Roth, PhD
Senior Editor

***** Reviewer's comments *****

Referee #1 (Comments on Novelty/Model System for Author):

All concerns were satisfactorily addressed. This reviewer acknoledge the inclusion of the requested in vivo experiments. The new data strenghten the conclusions and relevance of this study.

Referee #2 (Comments on Novelty/Model System for Author):

Authors should have worked with primary cells, patients' cells, organotypic cultures or transgenic mice and dedicated only a limited number of specific experiments to HaCaT cells.

Referee #2 (Remarks for Author):

Although authors carried out additional experiments, the conceptual problem remains. Authors chose to utilize HaCaT cells for their study, a model, which is progressively abandoned in dermatological research because HaCaT cells are transformed aneuploid immortal keratinocyte cell lines with limited physio-pathological relevance. Several studies have demonstrated that HaCaT cells are not a suitable alternative to primary keratinocytes (e.g. PMID: 24116291, PMID: 34909717). Authors should have worked with primary cells, patients' cells, organotypic cultures or transgenic mice and dedicated only a limited number of specific experiments to HaCaT cells.
Authors invested a lot of efforts and probably money in carrying very elegant techniques but unfortunately applied to a weak experimental model. Thus, the whole set of results generated with HaCaT cells is unfortunately highly questionable because of the flaw on physio-pathological relevance.
The title of the manuscript is: "Metabolic rewiring in psoriatic keratinocytes by Hsa-miR-31-5p identifies therapeutic intervention". A bit of temperance in the title would better fit with the experimental model, which is far from clinical relevance.
The readability of the manuscript has not been improved because authors were reluctant to remove some results. The figures are a jumble of results whose abundance does not improve physio-pathological relevance. This is a pity because the impact of their work is dampened. Submerging the readership with unnecessary results does not allow to accurately analyzing data.
Moreover, authors did not satisfactorily explained several discrepancies in their work. They never question the relevance of their cell line, which displays altered metabolic features when compared to primary cells.
Response to Fig. 1d is not satisfactory.
Authors: "We agree with the reviewer that there may be metabolic differences between proliferating and differentiated keratinocytes/cells. However, we would like to note that this does not appear to be always the case. For example, for fibroblasts it has been shown that quiescent fibroblasts to do not significantly differ in glucose flux activity compared to their proliferating counterparts (ref 4). Likely more important in the context of our findings, it has been shown that with respect to glutamine metabolism quiescent epithelial cells do differ from proliferating epithelial cells, where quiescent cells display altered glutamate metabolism (ref 6)."
Fibroblasts are very different cells from keratinocytes. Keratinocytes undergo a complex process of differentiation in the epidermis. It is not the case for fibroblasts in the dermis. Results cannot be justified by comparing apples and bananas.
OCR/ECAR ratio is not provided and interpreted.
Authors: "Seahorse experiments measures OXPHOS activity".
.. in relation to substrate uptake. When substrate uptake is altered (due to GLUT1 down-regulation for example), Seahorse technology does not allow discriminating the alterations of mitochondrial function due to changes in substrate uptake or in OXPHOS activity. It is actually one of the main pitfalls of Seahorse technology.

Referee #3 (Remarks for Author):

Is suitable for publication

response of external expert advisor:

"I agree that HaCaT cells are not an ideal model for this purpose, because they have a p53 mutation that may affect their metabolism. However, I am not quite as critical as the reviewer, because HaCaT cells are not transformed (they are just immortalized) and they differentiate normally in 3D cultures. Many highly relevant studies have been performed with HaCaT cells. However, they are not suitable for differentiation in 2D, in particular when they are already maintained in DMEM/10% FCS (and thus under relatively high Ca2+ concentrations).
I suggest that the authors perform a few experiments with primary human keratinocytes (which are commercially available). They could transfect these cells with miR-31 mimics as they did with HaCaT cells and then check the expression of a few selected genes/proteins (e.g. RPIA, GS, HK1 and IL6). This should be done under normal culture conditions (low calcium) and in response to Ca-induced differentiation. This is not difficult and should not take too much time, but it would show that the general effect of miR-31 is reproducible in primary human keratinocytes. In addition, they should address the other comments of the reviewer (at least by modification of the text), since they are relevant.

HaCaT cells are not an ideal model for this purpose, because they have a p53 mutation that may affect their metabolism.
The discussion initiated by reviewer #2 and as stated here by the advisor concerns the suitability of HaCaT cells as a model system.
First of all, we wish to be clear that we agree that HaCaT cells do not consitute a model system for psoriasis. However, here we employed HaCaT cells for discovery and understanding of the cellular consequences of miR-31 expression. We used unbiased proteomics to determine what protein changes are induced by miR-31 expression and secondly informed by the identified protein changes of metabolic enzymes, how this may translate into redirecting metabolism.
(-) With respect to the first we identified miR31 targets by performing SILAC with two commonly used cell lines (a keratinocyte line (HaCaT) and a non-keratinocyte line (HEK293T). The fact that we observe a strong concordance between results from HaCaT and 293T cells confirms the notion that the mechanism by which miRNAs control RNA expression is not cell-type specific and in this respect any cell line is suitable.
(-) With respect to the metabolic analysis, we stayed as close to the keratinocyte as possible by using HaCat cells. Culture medium requirements etc. essentialy preclude e.g. use of primary keratinocytes for experiments like metabolic tracing and Seahorse analysis as we have performed in our study.
Thus, undoubtedly as for any model, there are limitations in the use of a model like HaCaT cells also for discovery. The HaCaT cell line is a spontaneously immortalized aneuploid human keratinocyte cell line that exhibits normal keratinization [1] and has a p53 inactivating mutation [2]. The latter may have consequences for metabolism, but we like to note that p53 is predominantly expressed in keratinocytes of the basal layer of psoriasis affected skin, while largely absent in keratinocytes of suprabasal layer [3], suggesting that the HaCaT cell line actually has a p53 background quite similar to the keratinocytes in the suprabasal layer and thus in retrospect may even be considered quite a suitable model cell line to study metabolic consequences of miR-31 expression since miR-31 is also mainly expressed in suprabasal layer in psoriasis, where p53 is absent.

In summary, we never considered HaCaT cells a model system for psoriasis we used HaCaT cells to study effects of miR-31 expression. We consider this a valid approach for discovery and HaCaT cells are as a model in this respect as good (or as bad) as any other cell model. The "patho-physiological" model for psoriasis we used are the mice treated with IMQ and we translated our findings/discoveries to this model

system. The outcome of this shows that what we discovered in HaCaT cells apparently translates to large extend to this model system.
As this expert states ". Many highly relevant studies have been performed with HaCaT cells." and apparently that holds for our study as well.

However, we agree with reviewer #2 that the original title indeed intrinsically suggested, that we considered HaCaT cells as a model for psoriasis and as indicated above that was not our intention nor our perception. Consequently we changed the title to avoid this confusion and it now reads as "Metabolic rewiring in keratinocytes by miR-31-5p identifies therapeutic intervention for psoriasis"

I suggest that the authors perform a few experiments with primary human keratinocytes (which are commercially available).
we have performed additional experiments withprimary keratinocyte cultures (low and high CaCl2) and we have added these results to our manuscript (data of NHEK in Fig EV2C,EV2E, and EV2F). In short also in this cell system miR-31 regulates the same metabolic enzymes as in all the other cell lines we used in this study.

they should address the other comments of the reviewer (at least by modification of the text), since they are relevant**.**
We have added to the discussion a brief paragraph on the limitations of our study i.e. the use of HaCaT cells and Seahorse

2nd Feb 2023

Dear Prof. Burgering,

Thank you for the submission of your revised manuscript to EMBO Molecular Medicine.
We consulted the expert that had advised on your manuscript after the initial round of revisions. This advisor is now supportive of publication of your manuscript and stated:
"The authors performed the experiment that I requested, and the data obtained with primary human keratinocytes are consistent with those obtained with HaCaT cells. In addition, they performed additional experiments that have further improved the manuscript. I am fine with this revision."

We will therefore be able to accept your manuscript once the following editorial points will be addressed:

1/ Main manuscript text:
- Please remove the yellow highlights and only keep in track changes mode any new modification.
- Materials and Methods:
o Clinical specimens: Please include a sentence that the experiments conformed to the principles set out in the WMA Declaration of Helsinki and the Department of Health and Human Services Belmont Report.
o Animal experiments: please provide information on housing and husbandry conditions.
- Data Availability: Thank you for depositing your datasets in public repositories. Please also provide the corresponding URL and note that these datasets must be publicly available before acceptance of the manuscript. Kindly remove the reference to Table EV2 as this section is only for newly generated datasets deposited in public repositories.

2/ Figures:
Thank you for providing legends for the EV tables. Please add them in a separate tab.

3/ Please provide a complete author checklist, which you can download from our author guidelines (https://www.embopress.org/page/journal/17574684/authorguide#submissionofrevisions). Please insert information in the checklist that is also reflected in the manuscript. The completed author checklist will also be part of the RPF.

4/ Thank you for providing The paper explained. I introduced minor modifications, please let me know if you agree with the following or amend as you see fit:
Problem
Psoriasis is a chronic immune-mediated disorder of the skin that affects 0.1-0.4% of the total world population, and for which current treatment is still not optimal. Several skin diseases, including psoriasis, are characterized by a combination of keratinocyte hyperproliferation, consequent aberrant keratinocyte differentiation and immune cell activation. These diseases thus require metabolic adaptations that enable coexistence between hyperproliferative keratinocytes and activated immune cells.
Results
Here, we found that miR-31 expression in the spiny layer of psoriatic skin induced a metabolic shift toward glutamine utilization, leading to a massive production of aspartate. Aspartate in turn became a key mediator of metabolic crosstalk between keratinocytes and naive CD4+ T cells, promoting their differentiation toward Th17 cells. Importantly, targeting miR-31-induced metabolic changes and blocking intercellular metabolic crosstalk could alleviate psoriatic pathology.
Impact
Collectively, our data illustrate an emerging concept of metabolic interaction across cell compartments that characterizes disease development. This can be exploited to design effective treatment options for disease, as shown here for psoriasis.

5/ Thank you for providing a synopsis text and image. Please upload the figure as an independent PNG/TIF/JPEG file 550 px wide x 300-600 px high, and make sure that the text remains legible.

6/ We note that you agree with the publication of the Review Process File.

I look forward to receiving your revised manuscript.

Yours sincerely,

Lise Roth

Lise Roth, PhD
Senior Editor

The authors addressed the remaining editorial issues.

7th Feb 2023

Dear Prof. Burgering,

I am pleased to inform you that your manuscript is accepted for publication and is now being sent to our publisher to be included in the next available issue of EMBO Molecular Medicine.

We would like to remind you that as part of the EMBO Publications transparent editorial process initiative, EMBO Molecular Medicine will publish a Review Process File online to accompany accepted manuscripts. If you do NOT want the file to be published or would like to exclude figures, please immediately inform the editorial office via e-mail.

Please read below for additional IMPORTANT information regarding your article, its publication and the production process.

Congratulations on your interesting work!

With kind regards,

Lise Roth

Lise Roth, Ph.D
Senior Editor
EMBO Molecular Medicine

Follow us on Twitter @EmboMolMed
Sign up for eTOCs at embopress.org/alertsfeeds

*** *** *** IMPORTANT INFORMATION *** *** ***

SPEED OF PUBLICATION
The journal aims for rapid publication of papers, using using the advance online publication "Early View" to expedite the process: A properly copy-edited and formatted version will be published as "Early View" after the proofs have been corrected. Please help the Editors and publisher avoid delays by providing e-mail address(es), telephone and fax numbers at which author(s) can be contacted.

Should you be planning a Press Release on your article, please get in contact with embomolmed@wiley.com as early as possible, in order to coordinate publication and release dates.

LICENSE AND PAYMENT:

All articles published in EMBO Molecular Medicine are fully open access: immediately and freely available to read, download and share.

EMBO Molecular Medicine charges an article processing charge (APC) to cover the publication costs. You, as the corresponding author for this manuscript, should have already received a quote with the article processing fee separately. Please let us know in case this quote has not been received.

Once your article is at Wiley for editorial production you will receive an email from Wiley's Author Services system, which will ask you to log in and will present you with the publication license form for completion. Within the same system the publication fee can be paid by credit card, an invoice, pro forma invoice or purchase order can be requested.

Payment of the publication charge and the signed Open Access Agreement form must be received before the article can be published online.

PROOFS

You will receive the proofs by e-mail approximately 2 weeks after all relevant files have been sent o our Production Office. Please return them within 48 hours and if there should be any problems, please contact the production office at embopressproduction@wiley.com.

Please inform us if there is likely to be any difficulty in reaching you at the above address at that time. Failure to meet our deadlines may result in a delay of publication.

All further communications concerning your paper proofs should quote reference number EMM-2022-15674-V4 and be directed to the production office at embopressproduction@wiley.com.

EMBO Press Author Checklist

Corresponding Author Name: Boudewijn MT Burgering
Journal Submitted to: EMBO Molecular Medicine
Manuscript Number: EMM-2022-15674

USEFUL LINKS FOR COMPLETING THIS FORM
The EMBO Journal - Author Guidelines
EMBO Reports - Author Guidelines
Molecular Systems Biology - Author Guidelines
EMBO Molecular Medicine - Author Guidelines

Reporting Checklist for Life Science Articles (updated January 2022)
This checklist is adapted from Materials Design Analysis Reporting (MDAR) Checklist for Authors. MDAR establishes a minimum set of requirements in transparent reporting in the life sciences (see Statement of Task: 10.31222/osf.io/9sm4x). Please follow the journal's guidelines in preparing your manuscript.
Please note that a copy of this checklist will be published alongside your article.

Abridged guidelines for figures
1. Data
The data shown in figures should satisfy the following conditions:
- the data were obtained and processed according to the field's best practice and are presented to reflect the results of the experiments in an accurate and unbiased manner.
- ideally, figure panels should include only measurements that are directly comparable to each other and obtained with the same assay.
- plots include clearly labeled error bars for independent experiments and sample sizes. Unless justified, error bars should not be shown for technical replicates.
- if n<5, the individual data points from each experiment should be plotted. Any statistical test employed should be justified.
- Source Data should be included to report the data underlying figures according to the guidelines set out in the authorship guidelines on Data Presentation.

2. Captions
Each figure caption should contain the following information, for each panel where they are relevant:
- a specification of the experimental system investigated (eg cell line, species name).
- the assay(s) and method(s) used to carry out the reported observations and measurements.
- an explicit mention of the biological and chemical entity(ies) that are being measured.
- an explicit mention of the biological and chemical entity(ies) that are altered/varied/perturbed in a controlled manner.
- the exact sample size (n) for each experimental group/condition, given as a number, not a range;
- a description of the sample collection allowing the reader to understand whether the samples represent technical or biological replicates (including how many animals, litters, cultures, etc.).
- a statement of how many times the experiment shown was independently replicated in the laboratory.
- definitions of statistical methods and measures:
  - common tests, such as t-test (please specify whether paired vs. unpaired), simple χ2 tests, Wilcoxon and Mann-Whitney tests, can be unambiguously identified by name only, but more complex techniques should be described in the methods section;
  - are tests one-sided or two-sided?
  - are there adjustments for multiple comparisons?
  - exact statistical test results, e.g., P values = x but not P values < x;
  - definition of 'center values' as median or average;
  - definition of error bars as s.d. or s.e.m.

Please complete ALL of the questions below.
Select "Not Applicable" only when the requested information is not relevant for your study.

Materials

Newly Created Materials	Information included in the manuscript?	In which section is the information available? (Reagents and Tools Table, Materials and Methods, Figures, Data Availability Section)
New materials and reagents need to be available; do any restrictions apply?	Not Applicable	

Antibodies	Information included in the manuscript?	In which section is the information available? (Reagents and Tools Table, Materials and Methods, Figures, Data Availability Section)
For **antibodies** provide the following information:<br>- Commercial antibodies: RRID (if possible) or supplier name, catalogue number and or/clone number<br>- Non-commercial: RRID or citation	Yes	Table EV6

DNA and RNA sequences	Information included in the manuscript?	In which section is the information available? (Reagents and Tools Table, Materials and Methods, Figures, Data Availability Section)
**Short novel DNA or RNA including primers, probes**: provide the sequences.	Yes	Table EV5

Cell materials	Information included in the manuscript?	In which section is the information available? (Reagents and Tools Table, Materials and Methods, Figures, Data Availability Section)
**Cell lines:** Provide species information, strain. Provide accession number in repository **OR** supplier name, catalog number, clone number, and/**OR** RRID.	Yes	Materials and Methods
**Primary cultures:** Provide species, strain, sex of origin, genetic modification status.	Not Applicable	
Report if the cell lines were recently **authenticated** (e.g., by STR profiling) and tested for mycoplasma contamination.	Yes	Materials and Methods

Experimental animals	Information included in the manuscript?	In which section is the information available? (Reagents and Tools Table, Materials and Methods, Figures, Data Availability Section)
**Laboratory animals or Model organisms:** Provide species, strain, sex, age, genetic modification status. Provide accession number in repository **OR** supplier name, catalog number, clone number, **OR** RRID.	Yes	Materials and Methods
**Animal observed in or captured from the field:** Provide species, sex, and age where possible.	Not Applicable	
Please detail **housing and husbandry conditions**.	Yes	Materials and Methods

Plants and microbes	Information included in the manuscript?	In which section is the information available? (Reagents and Tools Table, Materials and Methods, Figures, Data Availability Section)
**Plants:** provide species and strain, ecotype and cultivar where relevant, unique accession number if available, and source (including location for collected wild specimens).	Not Applicable	
**Microbes:** provide species and strain, unique accession number if available, and source.	Not Applicable	

Human research participants	Information included in the manuscript?	In which section is the information available? (Reagents and Tools Table, Materials and Methods, Figures, Data Availability Section)
If collected and within the bounds of privacy constraints report on age, sex and gender or ethnicity for all study participants.	Yes	Materials and Methods

Core facilities	Information included in the manuscript?	In which section is the information available? (Reagents and Tools Table, Materials and Methods, Figures, Data Availability Section)
If your work benefited from core facilities, was their service mentioned in the acknowledgments section?	Yes	Acknowledgments

Design

Study protocol	Information included in the manuscript?	In which section is the information available? (Reagents and Tools Table, Materials and Methods, Figures, Data Availability Section)
If study protocol has been **pre-registered, provide DOI in the manuscript**. For clinical trials, provide the trial registration number **OR** cite DOI.	Not Applicable	
Report the **clinical trial registration number** (at ClinicalTrials.gov or equivalent), where applicable.	Not Applicable	

Laboratory protocol	Information included in the manuscript?	In which section is the information available? (Reagents and Tools Table, Materials and Methods, Figures, Data Availability Section)
Provide DOI OR other citation details if **external detailed step-by-step protocols** are available.	Yes	Materials and Methods and Figures

Experimental study design and statistics	Information included in the manuscript?	In which section is the information available? (Reagents and Tools Table, Materials and Methods, Figures, Data Availability Section)
Include a statement about **sample size** estimate even if no statistical methods were used.	Yes	Materials and Methods and Figures
Were any steps taken to minimize the effects of subjective bias when allocating animals/samples to treatment (e.g. **randomization procedure**)? If yes, have they been described?	Yes	Materials and Methods
Include a statement about **blinding** even if no blinding was done.	Yes	Materials and Methods
Describe **inclusion/exclusion criteria** if samples or animals were excluded from the analysis. Were the criteria pre-established?<br><br>If sample or data points were omitted from analysis, report if this was due to attrition or intentional exclusion and provide justification.	Yes	Materials and Methods
For every figure, are **statistical tests** justified as appropriate? Do the data meet the assumptions of the tests (e.g., normal distribution)? Describe any methods used to assess it. Is there an estimate of variation within each group of data? Is the variance similar between the groups that are being statistically compared?	Yes	Figure legends

Sample definition and in-laboratory replication	Information included in the manuscript?	In which section is the information available? (Reagents and Tools Table, Materials and Methods, Figures, Data Availability Section)
In the figure legends: state number of times the experiment was **replicated** in laboratory.	Yes	Figure legends
In the figure legends: define whether data describe **technical or biological replicates**.	Yes	Figure legends

Ethics

Ethics	Information included in the manuscript?	In which section is the information available? (Reagents and Tools Table, Materials and Methods, Figures, Data Availability Section)
Studies involving **human participants**: State details of **authority granting ethics approval** (IRB or equivalent committee(s), provide reference number for approval.	Yes	Materials and Methods
Studies involving **human participants**: Include a statement confirming that **informed consent** was obtained from all subjects and that the experiments conformed to the principles set out in the WMA Declaration of Helsinki and the Department of Health and Human Services Belmont Report.	Yes	Materials and Methods
Studies involving **human participants:** For publication of **patient photos**, include a statement confirming that consent to publish was obtained.	Not Applicable	
Studies involving experimental **animals**: State details of **authority granting ethics approva**l (IRB or equivalent committee(s), provide reference number for approval. Include a statement of compliance with ethical regulations.	Yes	Materials and Methods
Studies involving **specimen and field samples:** State if relevant **permits** obtained, provide details of authority approving study; if none were required, explain why.	Not Applicable	

Dual Use Research of Concern (DURC)	Information included in the manuscript?	In which section is the information available? (Reagents and Tools Table, Materials and Methods, Figures, Data Availability Section)
Could your study fall under dual use research restrictions? Please check biosecurity documents and list of **select agents and toxins** (CDC): https://www.selectagents.gov/sat/list.htm	Not Applicable	
If you used a select agent, is the security level of the lab appropriate and reported in the manuscript?	Not Applicable	
If a study is subject to dual use research of concern regulations, is the name of the **authority granting approval and reference number** for the regulatory approval provided in the manuscript?	Not Applicable	

Reporting

The MDAR framework recommends adoption of discipline-specific guidelines, established and endorsed through community initiatives. Journals have their own policy about requiring specific guidelines and recommendations to complement MDAR.

Adherence to community standards	Information included in the manuscript?	In which section is the information available? (Reagents and Tools Table, Materials and Methods, Figures, Data Availability Section)
State if relevant guidelines or checklists (e.g., **ICMJE, MIBBI, ARRIVE, PRISMA**) have been followed or provided.	Not Applicable	
For **tumor marker prognostic studies**, we recommend that you follow the **REMARK** reporting guidelines (see link list at top right). See author guidelines, under 'Reporting Guidelines'. Please confirm you have followed these guidelines.	Not Applicable	
For **phase II and III randomized controlled trials**, please refer to the **CONSORT** flow diagram (see link list at top right) and submit the CONSORT checklist (see link list at top right) with your submission. See author guidelines, under 'Reporting Guidelines'. Please confirm you have submitted this list.	Not Applicable	

Data Availability

Data availability	Information included in the manuscript?	In which section is the information available? (Reagents and Tools Table, Materials and Methods, Figures, Data Availability Section)
Have **primary datasets** been deposited according to the journal's guidelines (see 'Data Deposition' section) and the respective accession numbers provided in the Data Availability Section?	Yes	Materials and Methods
Were **human clinical and genomic datasets** deposited in a public access-controlled repository in accordance to ethical obligations to the patients and to the applicable consent agreement?	Not Applicable	
Are **computational models** that are central and integral to a study available without restrictions in a machine-readable form? Were the relevant accession numbers or links provided?	Not Applicable	
If publicly available data were reused, provide the respective **data citations in the reference list**.	Yes	Materials and Methods
